# Supplementary material for: Design, Synthesis, and Antitumor Activity Evaluation of Proteolysis-Targeting Chimeras as Degraders of Extracellular Signal-Regulated Kinases 1/2
Source: Int J Mol Sci. 2023 Nov 14;24(22):16290. doi: 10.3390/ijms242216290 (PMC10671693; doi:10.3390/ijms242216290)
Supplement: Supplementary file 1 [file ijms-24-16290-s001.zip › ijms-2705159-supplementary.pdf]

Supporting information

Design, Synthesis, and Antitumor Activity Evaluation of Proteolysis-Targeting Chimeras as Degraders of Extracellular Signal-Regulated Kinases 1/2

Pengming Pan, Yichao He, Tongtong Geng, Zhongtang Li, Zhongjun Li and Xiangbao Meng *

State Key Laboratory of Natural and Biomimetic Drugs, School of Pharmaceutical Sciences, Peking University, Beijing 100191, China
* Correspondence: xbmeng@bjmu.edu.cn

Contents

1. Docking result……………………………………………………………2-3

2. Determination of DC_{50} for **B1-10J**………………………………………….....3

3. Supplementary figure for xenograft experiment…………………………………...4

4. Synthesis of compouds……………………………………………………………4-30

5. ^{1}H NMR and ^{13}C NMR spectrum………………………………………….….30-72

6. HRMS spectrum………………………………………………………… 73-87

7. HPLC spectrum…………………………………..……………………..87-103

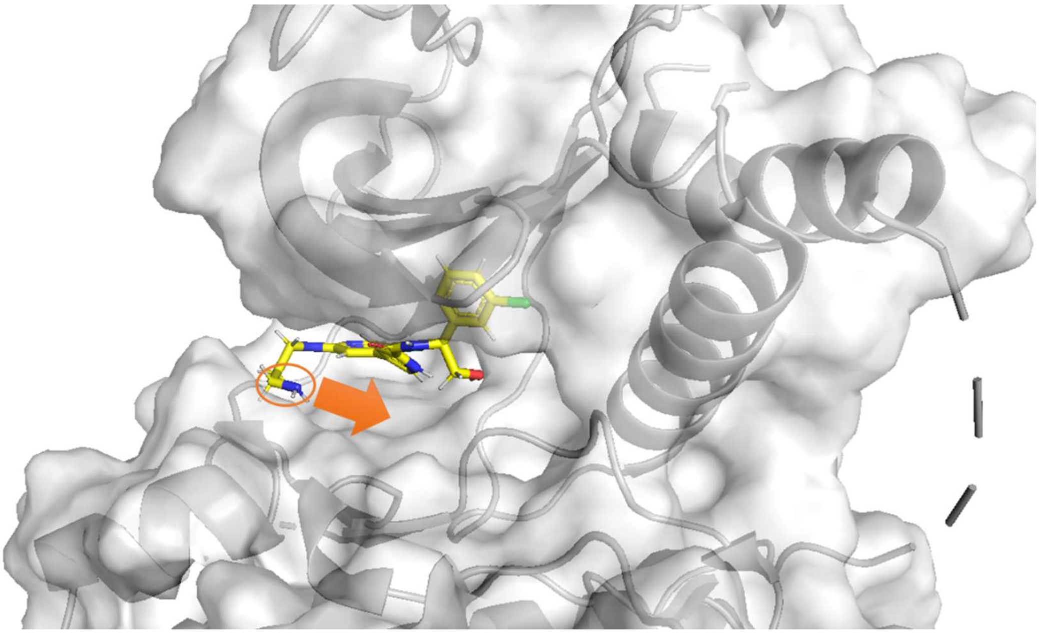

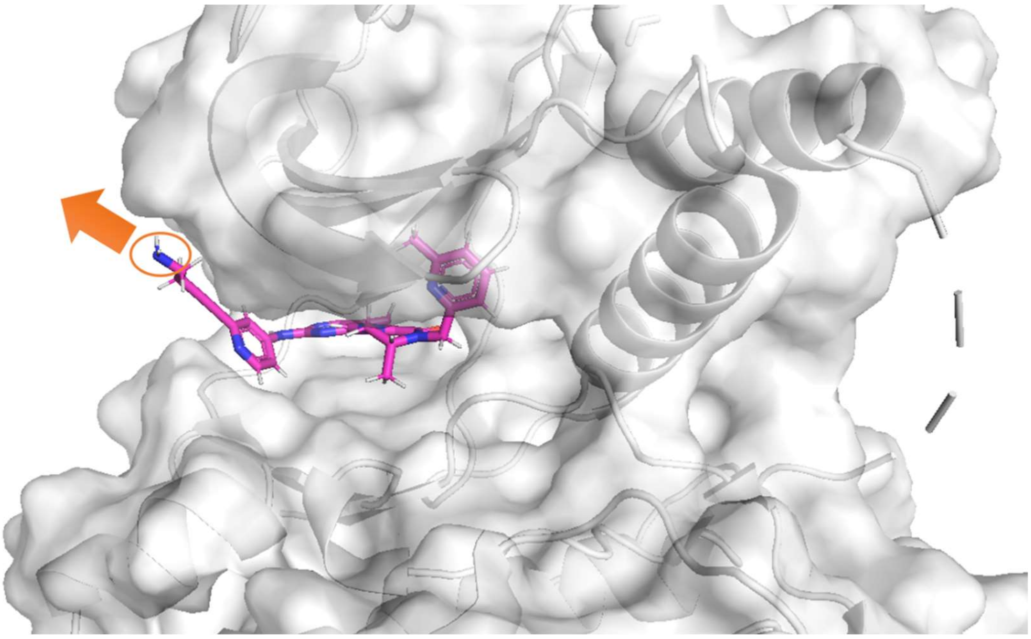

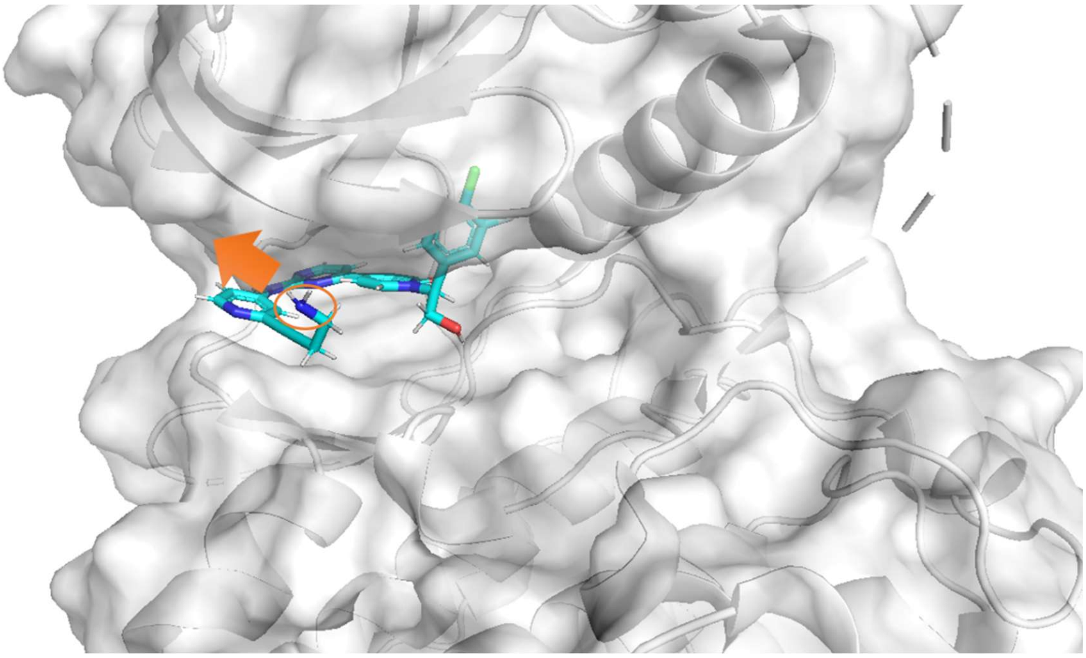

Figure S1. Docking model of the modified inhibitors to ERK1/2 (PDB: 6gdq).

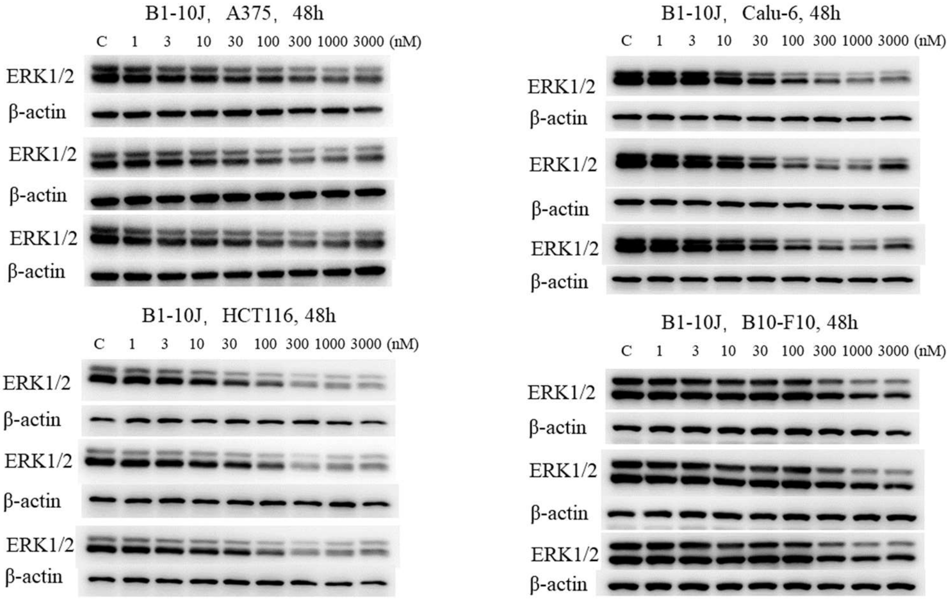

Figure S2. Wide applicability of BI-10J in various human tumor cells. Cells were treated with the indicated concentrations of BI-10J for 48 h before western blot.

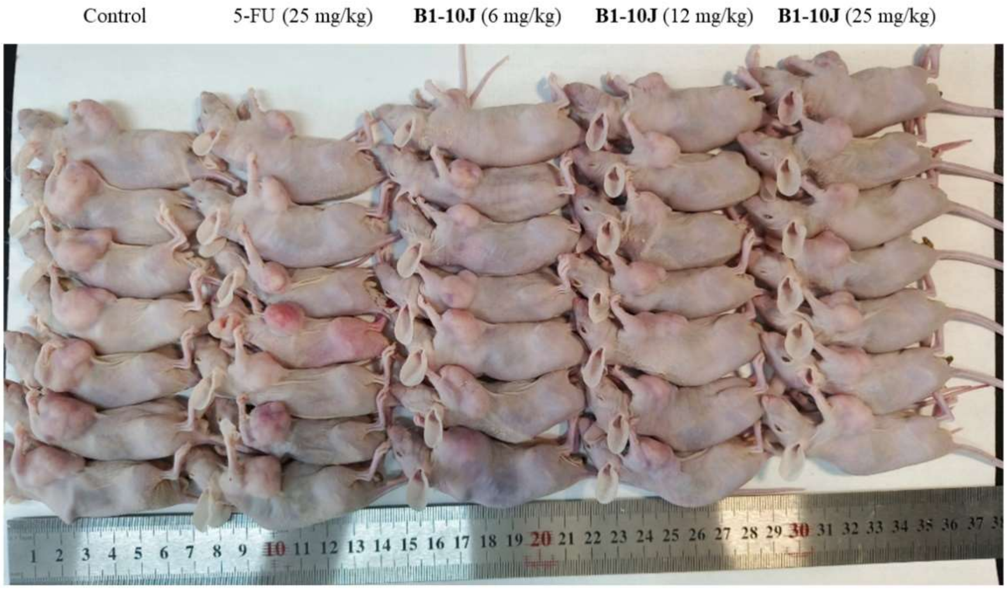

Figure S3. The antitumor activity of B1-10J in a HCT116 subcutaneous xenograft model.

4. Synthesis of compouds

4.1. Chemistry

4.1.1. Synthesis of intermediates.

4.1.1.1. (E)-7-(2,6-dimethoxy-4-(3-oxo-3-(6-oxo-3,6-dihydropyridin-1(2H)-yl)prop-1-en-1-yl)phenoxy)heptanoic acid (7)

Using a method similar to the one reported in literature[21]. White solid; m.p. 97-98℃; ^{1}H NMR (400 MHz, $CDCl_3$) δ 9.29 (s, 1H), 7.68 (d, J = 15.5 Hz, 1H), 7.41 (d, J = 15.5 Hz, 1H), 6.94 (dt, J = 9.3, 4.2 Hz, 1H), 6.80 (s, 2H), 6.05 (dt, J = 9.7, 1.7 Hz, 1H), 4.06–3.96 (m, 4H), 3.87 (s, 6H), 2.52–2.43 (m, 2H), 2.36 (t, J = 7.5 Hz, 2H), 1.79–1.72 (m, 2H), 1.72–1.62 (m, 2H), 1.54–1.44 (m, 2H), 1.43–1.33 (m, 2H). ^{13}C NMR (101 MHz, $CDCl_3$) δ 179.46, 168.95, 165.91, 153.55, 145.62, 143.97, 139.29, 130.39, 125.75, 120.85, 105.59, 73.35, 56.16, 41.67, 33.95, 29.85, 28.80, 25.47, 24.79, 24.63. HRMS (m/z): calculated for $C_{23}H_{29}NO_7$ $[M+Na]^+$: 454.1836, found 454.1858.

*4.1.1.2 tert-butyl (3-((5-chloro-4-iodopyridin-2-yl)amino)propyl)carbamate (**9**)*

A mixture of 5-chloro-2-fluoro-4-iodopyridine **8** (1 g, 3.89 mmol), tert-butyl (3-

aminopropyl)carbamate (677 mg, 3.89 mmol), DIEA (1.02 mL, 5.84 mmol) and DMF (15mL) was stirred at 70°C for 12 hours. The mixture was dissolved in EA and washed with brine, dried over Na_2SO_4, filtered and concentrated. The crude product was purified by column chromatography (PE:EA = 2:1). The intermediate **9** (735 mg, 46%) was obtained as a white solid; m.p. 135-136°C; ^{1}H NMR (400 MHz, $CDCl_3$) δ 8.01 (s, 1H), 6.94 (s, 1H), 5.05 (s, 1H), 4.84 (s, 1H), 3.34 (q, J = 6.3 Hz, 2H), 3.22 (q, J = 6.4 Hz, 2H), 1.78 – 1.69 (m, 2H), 1.46 (s, 9H). ^{13}C NMR (101 MHz, $CDCl_3$) δ 156.97, 156.48, 145.67, 123.65, 118.07, 109.63, 79.44, 38.59, 37.46, 29.88, 28.41. HRMS (m/z): calculated for $C_{13}H_{19}ClIN_3O_2$ $[M+H]^+$: 412.0283, found 412.0302.

*4.1.1.3. 4-(2-((3-((tert-butoxycarbonyl)amino)propyl)amino)-5-chloropyridin-4-yl)-1H-pyrrole-2-carboxylic acid (**14**)*

A mixture of **9** (735 mg, 1.79 mmol), 1-(tert-butyl) 2-methyl 4-(4,4,5,5-tetramethyl-1,3,2-dioxaborolan-2-yl)-1H-pyrrole-1,2-dicarboxylate **12** (628 mg, 1.79 mmol), $Pd(PPh)_3$ (104 mg, 0.09 mmol), K_2CO_3 (618 mg, 4.48 mmol), and dioxane: H_2O (= 4:1, 10 mL: 2.5 mL) was stirred at 70°C for 8 hours under argon. The mixture was dissolved in EA and washed with brine, dried over Na_2SO_4, filtered and concentrated. The crude product is directly put into the next step without further purification. A mixture of the crude product **13**, 2N LiOH (5 mL) and THF (5 mL) was stirred at room temperature for 12h. Then, add 0.5N HCl to adjust the pH to 4-5. After stirring for 2h, white solids precipitate. Filter and wash with methanol for 3 times to obtain the intermediate **14** (247 mg, 35% over 2 steps). White solid; m.p. 137-138℃; 1H NMR (400 MHz, DMSO-*d*6) δ 12.55 (s, 1H), 12.14 (s, 1H), 7.96 (s, 1H), 7.45 (s, 1H), 7.10 (s, 1H), 6.82 (s, 1H), 6.62 (s, 1H), 6.51 (s, 1H), 3.21 (q, J = 6.5 Hz, 2H), 2.98 (q, J = 6.5 Hz, 2H), 1.68–1.56 (m, 2H), 1.36 (s, 9H). ^{13}C NMR (101 MHz, DMSO-*d*6) δ 162.12, 158.47, 156.08, 147.82, 140.96, 124.04, 120.71, 115.65, 114.60, 107.02, 77.87, 39.03, 38.27, 29.83, 28.70. HRMS (m/z): calculated for $C_{18}H_{23}ClN_4O_4$ $[M+H]^+$: 395.1481, found 395.1504.

*4.1.1.4. (S)-4-(2-((3-aminopropyl)amino)-5-chloropyridin-4-yl)-N-(1-(3-chlorophenyl)-2-hydroxyethyl)-1H-pyrrole-2-carboxamide (**A**)*

A mixture of **14** (247 mg, 0.63 mmol), (S)-2-amino-2-(3-chlorophenyl)ethan-1-ol (107 mg, 0.63 mmol), HATU (289 mg, 0.76 mmol), DIEA (156 ul, 0.95 mmol) and DMF was stirred at room temperature for 12 hours. The mixture was dissolved in EA and washed with brine, dried over Na_2SO_4, filtered and concentrated. The crude product was then added to TFA and stirred at room temperature for 2h and concentrated to remove TFA. The crude product was purified by column chromatography (DCM:MeOH = 10:1, 0.3% ammonia). The intermediate **A** (200 mg, 71%) was obtained. White solid; m.p. 123-124℃; ^{1}H NMR (400 MHz, CD_3OD) δ 7.94 (s, 1H), 7.50 (s, 1H), 7.46 (s, 1H), 7.40 (s, 1H), 7.38–7.24 (m, 3H), 6.67 (s, 1H), 5.16 (t, J = 6.4 Hz, 1H), 3.87 (d, J = 6.4 Hz, 2H), 3.37 (s, 5H), 2.79 (t, J = 7.0 Hz, 2H), 1.85–1.75 (m, 2H). ^{13}C NMR (101 MHz, CD_3OD) δ 161.65, 158.11, 146.68, 142.73, 142.33, 133.94, 129.58, 127.02, 126.80, 125.96, 125.17, 122.63, 120.59, 116.63, 110.71, 106.81, 64.38, 55.26, 38.58, 38.35, 31.47. HRMS (m/z): calculated for $C_{21}H_{23}Cl_2N_5O_2$ $[M+H]^+$: 448.1302, found 448.1331.

4.1.1.5. (R)-7-(2-((2-(4-aminobut-1-yn-1-yl)pyridin-4-yl)amino)-5-methylpyrimidin-4-yl)-3-methyl-2-((6-methylpyridin-2-yl)methyl)-3,4-dihydropyrrolo[1,2-a]pyrazin-1(2H)-one (**B**)

Using a method similar to the one reported in literature[15]. A mixture of **19** (2.0 g, 5.25 mmol), tert-butyl (4-(4-aminopyridin-2-yl)but-3-yn-1-yl)carbamate (1.37g, 5.25 mmol), BrettPhos third-generation precatalyst (CAS: 1470372-59-8, 238 mg, 0.26 mmol), Cs_2CO_3 (2.42 g, 10.5 mmol) and *t*-BuOH: dioxane (1: 1, 20mL) was stirred at 85°C for 6 hours under argon. The mixture was dissolved in EA and washed with brine, dried over Na_2SO_4, filtered and concentrated. The crude product was then added to TFA and stirred at room temperature for 2h and concentrated to remove TFA. The crude product was purified by column chromatography (DCM:MeOH = 8:1, 0.3% ammonia). The intermediate **B** (1.2 g, 45%) was obtained. Brown oil; ^{1}H NMR (400 MHz, CD_3OD) δ 8.38 (s, 1H), 8.32 (s, 1H), 8.29 (d, J = 6.5 Hz, 1H), 7.83 (dd, J = 6.6, 2.3 Hz, 1H), 7.76 (t, J = 7.8 Hz, 1H), 7.69 (d, J = 1.7 Hz, 1H), 7.58 (d, J = 1.6 Hz, 1H), 7.32 (d, J = 7.8

Hz, 1H), 7.25 (d, J = 7.7 Hz, 1H), 5.33 (d, J = 15.8 Hz, 1H), 4.49–4.39 (m, 2H), 4.22 (dd, J = 13.1, 2.2 Hz, 1H), 4.11–4.01 (m, 1H), 3.30 (t, J = 6.6 Hz, 2H), 3.00 (t, J = 6.6 Hz, 2H), 2.58 (s, 3H), 2.42 (s, 3H), 1.29 (d, J = 6.6 Hz, 3H). ^{13}C NMR (101 MHz, CD_3OD) δ 159.45, 159.21, 158.97, 157.87, 157.02, 156.28, 151.69, 144.94, 138.22, 137.98, 126.85, 124.00, 123.53, 122.40, 120.02, 119.13, 115.43, 113.68, 111.65, 78.48, 52.43, 48.88, 48.71, 37.78, 22.41, 17.52, 16.55, 15.89. HRMS (m/z): calculated for $C_{29}H_{30}N_8O$ $[M+H]^+$: 507.2615, found 507.2612.

*4.1.1.6. (R)-3-methyl-7-(5-methyl-2-((2-(4-(piperazin-1-yl)but-1-yn-1-yl)pyridin-4-yl)amino)pyrimidin-4-yl)-2-((6-methylpyridin-2-yl)methyl)-3,4-dihydropyrrolo[1,2-a]pyrazin-1(2H)-one (**B-P**)*

This compound was prepared using a procedure similar to that used for **B**. Brown oil; ^{1}H NMR (400 MHz, CD_3OD) δ 8.33 (s, 1H), 8.30 – 8.21 (m, 2H), 8.07 (d, J = 6.9 Hz, 1H), 7.80 (t, J = 7.8 Hz, 1H), 7.69 (d, J = 1.7 Hz, 1H), 7.51 (d, J = 1.7 Hz, 1H), 7.34 (d, J = 7.8 Hz, 1H), 7.28 (d, J = 7.7 Hz, 1H), 5.30 (d, J = 15.9 Hz, 1H), 4.49–4.40 (m, 2H), 4.27–4.18 (m, 1H), 4.12–4.01 (m, 1H), 3.28–3.20 (m, 4H), 2.87–2.77 (m, 8H), 2.58 (s, 3H), 2.41 (s, 3H), 1.28 (d, J = 6.7 Hz, 3H). ^{13}C NMR (101 MHz, CD_3OD) δ 162.15, 161.81, 161.47, 161.13, 159.41, 159.16, 157.56, 156.52, 156.00, 153.87, 141.67, 138.84, 135.28, 126.97, 124.07, 122.67, 120.99, 119.33, 118.28, 115.36, 113.63, 111.26, 97.99, 73.85, 55.10, 52.50, 49.01, 43.33, 22.09, 17.03, 16.58, 15.94. HRMS (m/z): calculated for $C_{33}H_{37}N_9O$ $[M+H]^+$: 576.3194, found 576.3199.

*4.1.1.7. (R)-3-methyl-7-(5-methyl-2-((2-(4-(methylamino)but-1-yn-1-yl)pyridin-4-yl)amino)pyrimidin-4-yl)-2-((6-methylpyridin-2-yl)methyl)-3,4-dihydropyrrolo[1,2-a]pyrazin-1(2H)-one (**B-J**)*

This compound was prepared using a procedure similar to that used for **B**. Brown oil; ^{1}H NMR (400 MHz, CD_3OD) δ 8.26–8.19 (m, 2H), 8.16 (s, 1H), 7.73 (t, J = 7.8 Hz, 1H), 7.66 (d, J = 8.5 Hz, 2H), 7.54 (s, 1H), 7.28 (d, J = 7.8 Hz, 1H), 7.21 (d, J = 7.7 Hz, 1H), 5.30 (d, J = 15.8 Hz, 1H), 4.47–4.37 (m, 2H), 4.22 (d, J = 13.0 Hz, 1H), 4.10–4.00

(m, 1H), 3.36 (t, J = 6.5 Hz, 2H), 3.01 (t, J = 6.5 Hz, 2H), 2.80 (s, 3H), 2.55 (s, 3H), 2.36 (s, 3H), 1.29 (d, J = 6.6 Hz, 3H). ^{13}C NMR (101 MHz, CD_3OD) δ 159.31, 159.23, 158.73, 158.01, 157.43, 156.51, 149.32, 148.23, 141.12, 137.84, 126.73, 123.90, 123.74, 122.17, 118.89, 118.85, 115.27, 113.69, 111.69, 84.87, 81.79, 52.44, 48.91, 47.25, 32.43, 22.65, 16.58, 16.38, 15.96. HRMS (m/z): calculated for $C_{30}H_{32}N_8O$ $[M+H]^+$: 521.2772, found 521.2782.

*4.1.1.8. (R)-7-(2-((2-((3-aminopropyl)amino)pyridin-4-yl)amino)-5-methylpyrimidin-4-yl)-3-methyl-2-((6-methylpyridin-2-yl)methyl)-3,4-dihydropyrrolo[1,2-a]pyrazin-1(2H)-one (**B-N**)*

This compound was prepared using a procedure similar to that used for **B**. White oil; ^{1}H NMR (400 MHz, CD_3OD) δ 8.26 (s, 1H), 8.21 (t, J = 7.9 Hz, 1H), 7.76 (s, 1H), 7.72 –7.66 (m, 2H), 7.63 (d, J = 7.9 Hz, 1H), 7.58 (d, J = 7.2 Hz, 1H), 7.53 (d, J = 1.6 Hz, 1H), 6.86 (d, J = 8.0 Hz, 1H), 5.42 (d, J = 16.4 Hz, 1H), 4.65 (d, J = 16.4 Hz, 1H), 4.57 –4.48 (m, 1H), 4.27 (d, J = 13.2 Hz, 1H), 4.22–4.11 (m, 1H), 3.43 (t, J = 6.6 Hz, 2H), 3.13 (t, J = 7.6 Hz, 2H), 2.76 (s, 3H), 2.37 (s, 3H), 2.16–2.06 (m, 2H), 1.34 (d, J = 6.6 Hz, 3H). ^{13}C NMR (101 MHz, CD_3OD) δ 161.93, 161.58, 159.56, 158.77, 157.04, 155.58, 154.07, 153.50, 152.85, 143.37, 134.94, 127.11, 124.84, 123.56, 121.39, 120.09, 118.23, 115.33, 114.04, 104.64, 92.50, 53.16, 39.10, 36.96, 26.04, 19.82, 16.58, 16.00. HRMS (m/z): calculated for $C_{28}H_{33}N_9O$ $[M+H]^+$: 512.2881, found 512.2897.

*4.1.1.9. (R)-7-(2-((3-(4-aminobut-1-yn-1-yl)phenyl)amino)-5-methylpyrimidin-4-yl)-3-methyl-2-((6-methylpyridin-2-yl)methyl)-3,4-dihydropyrrolo[1,2-a]pyrazin-1(2H)-one (**B-B**)*

This compound was prepared using a procedure similar to that used for **B**. White oil; ^{1}H NMR (400 MHz, CD_3OD) δ 8.20 (s, 1H), 8.15 (s, 1H), 7.71 (t, J = 7.8 Hz, 1H), 7.60 (d, J = 11.5 Hz, 2H), 7.50 (d, J = 8.3 Hz, 1H), 7.29–7.17 (m, 3H), 7.03 (d, J = 7.6 Hz, 1H), 5.30 (d, J = 15.7 Hz, 1H), 4.43–4.30 (m, 2H), 4.15 (d, J = 13.0 Hz, 1H), 3.99 (s, 1H), 3.23 (t, J = 6.4 Hz, 2H), 2.90 (t, J = 6.9 Hz, 2H), 2.55 (s, 3H), 2.34 (s, 3H), 1.25

(d, J = 6.6 Hz, 3H). ^{13}C NMR (101 MHz, CD_3OD) δ 159.28, 159.12, 158.77, 158.27, 158.01, 156.51, 140.88, 137.80, 128.27, 126.54, 124.17, 124.05, 123.72, 122.96, 122.19, 121.48, 118.89, 118.41, 116.92, 113.85, 83.41, 83.04, 52.35, 48.84, 38.42, 22.64, 17.80, 16.44, 15.88. HRMS (m/z): calculated for $C_{30}H_{31}N_7O$ $[M+H]^+$: 506.2663, found 506.2691.

*4.1.1.10. (R)-7-(2-((1-(4-aminobutyl)-1H-pyrazol-3-yl)amino)-5-methylpyrimidin-4-yl)-3-methyl-2-((6-methylpyridin-2-yl)methyl)-3,4-dihydropyrrolo[1,2-a]pyrazin-1(2H)-one (**B-BZ**)*

This compound was prepared using a procedure similar to that used for **B**. White solid; m.p. 92-93°C; ^1H NMR (400 MHz, CD_3OD) δ 8.18 (s, 1H), 7.66 (t, J = 7.7 Hz, 1H), 7.56 (d, J = 18.3 Hz, 2H), 7.50 (d, J = 2.3 Hz, 1H), 7.22 (d, J = 7.8 Hz, 1H), 7.16 (d, J = 7.7 Hz, 1H), 6.77 (d, J = 2.3 Hz, 1H), 5.26 (d, J = 15.8 Hz, 1H), 4.41–4.31 (m, 2H), 4.14 (d, J = 13.0 Hz, 1H), 4.07 (t, J = 6.9 Hz, 2H), 4.02–3.93 (m, 1H), 2.64 (t, J = 7.3 Hz, 2H), 2.52 (s, 3H), 2.34 (s, 3H), 1.86 (p, J = 7.0 Hz, 2H), 1.46 (p, J = 7.6 Hz, 2H), 1.23 (d, J = 6.6 Hz, 3H). ^{13}C NMR (101 MHz, CD_3OD) δ 159.25, 159.22, 159.18, 157.99, 157.91, 156.56, 148.96, 137.75, 130.22, 126.49, 124.14, 123.76, 122.11, 118.82, 116.57, 113.73, 96.27, 52.35, 51.01, 48.85, 40.54, 29.08, 27.33, 22.66, 16.36, 15.92. HRMS (m/z): calculated for $C_{27}H_{33}N_9O$ $[M+H]^+$: 500.2881, found 500.2902.

*4.1.1.11. (S)-4-(2-((2-(4-aminobut-1-yn-1-yl)pyridin-4-yl)amino)pyrimidin-4-yl)-1-(1-(4-chloro-3-fluorophenyl)-2-hydroxyethyl)pyridin-2(1H)-one (**C**)*

Using a method similar to the one reported in literature[32]. White solid; m.p. 138-139°C; ^1H NMR (400 MHz, CD_3OD) δ 8.68 (s, 1H), 8.25 (s, 1H), 7.96 (s, 1H), 7.93–7.88 (m, 1H), 7.78 (s, 1H), 7.55–7.43 (m, 2H), 7.39–7.30 (m, 2H), 7.21 (d, J = 8.6 Hz, 1H), 7.09 (s, 1H), 6.15 (t, J = 6.2 Hz, 1H), 4.38–4.28 (m, 1H), 4.28–4.18 (m, 1H), 2.91 (t, J = 6.3 Hz, 2H), 2.63 (t, J = 6.3 Hz, 2H). HRMS (m/z): calculated for $C_{26}H_{22}ClFN_6O_2$ $[M+H]^+$: 505.1550, found 505.1547.

4.1.2. General Procedure for A1 ~ A4, C1 ~ C4, B1-4 ~ B1-12, B1-3P ~ B1-5P, B1-10N (B, BZ), B2-4 ~ B2-14 and B2-12N, B3, B4.

A mixture of ligands of ERK1/2 (1 eq), complex of E3 ligand and linker (1 eq), HATU (1.2 eq), DIEA (1.5 eq) and DMF was stirred at room temperature for 12 hours. The mixture was dissolved in EA and washed with brine, dried over Na_2SO_4, filtered and concentrated. The crude product was purified by column chromatography (DCM:MeOH = 10:1, 0.3% ammonia). The corresponding Amide compound (31–85%) was obtained.

*4.1.2.1. 4-(5-chloro-2-((3-(8-((2-(2,6-dioxopiperidin-3-yl)-1,3-dioxoisoindolin-4-yl)amino)octanamido)propyl)amino)pyridin-4-yl)-N-((S)-1-(3-chlorophenyl)-2-hydroxyethyl)-1H-pyrrole-2-carboxamide (**A1**)*

Yellow solid; m.p. 101-102℃; ^{1}H NMR (400 MHz, DMSO-*d6*) δ 11.86 (s, 1H), 11.09 (s, 1H), 8.45 (d, J = 8.2 Hz, 1H), 7.98 (s, 1H), 7.78 (t, J = 5.1 Hz, 1H), 7.57 (t, J = 7.8 Hz, 1H), 7.46 (s, 1H), 7.43–7.34 (m, 4H), 7.33–7.28 (m, 1H), 7.06 (d, J = 8.6 Hz, 1H), 7.01 (d, J = 7.0 Hz, 1H), 6.62 (s, 1H), 6.56 (t, J = 5.8 Hz, 1H), 6.50 (t, J = 5.9 Hz, 1H), 5.12–4.96 (m, 3H), 3.73–3.65 (m, 2H), 3.33–3.16 (m, 5H), 3.11 (q, J = 6.5 Hz, 2H), 2.95–2.81 (m, 1H), 2.64–2.55 (m, 1H), 2.10–1.97 (m, 3H), 1.69–1.60 (m, 2H), 1.59–1.44 (m, 4H), 1.36–1.20 (m, 6H). ^{13}C NMR (101 MHz, DMSO-*d6*) δ 173.25, 172.58, 170.54, 169.41, 167.75, 160.45, 158.47, 147.84, 146.88, 144.58, 141.45, 136.72, 133.28, 132.64, 130.42, 127.28, 127.24, 127.07, 126.39, 122.49, 120.15, 117.59, 115.73, 110.83, 110.72, 109.47, 106.92, 64.67, 55.08, 49.01, 42.29, 39.13, 36.80, 35.90, 31.45, 29.55, 29.13, 29.09, 28.97, 26.69, 25.75, 22.62. HRMS (m/z): calculated for $C_{42}H_{46}Cl_2N_8O_7$ $[M+H]^+$: 845.2939, found 845.2990. Purity: 97.2%.

*4.1.2.2. N1-(3-((5-chloro-4-(5-(((S)-1-(3-chlorophenyl)-2-hydroxyethyl)carbamoyl)-1H-pyrrol-3-yl)pyridin-2-yl)amino)propyl)-N10-((S)-1-((2S,4R)-4-hydroxy-2-(((S)-1-(4-(4-methylthiazol-5-yl)phenyl)ethyl)carbamoyl)pyrrolidin-1-yl)-3,3-dimethyl-1-oxobutan-2-yl)decanediamide (**A2**)*

White solid; m.p. 140-141℃; ^{1}H NMR (400 MHz, CD_3OD) δ 8.85 (s, 1H), 7.93 (s, 1H), 7.52 (s, 1H), 7.45 (s, 1H), 7.43–7.37 (m, 5H), 7.36–7.21 (m, 3H), 6.66 (s, 1H), 5.18 (t, J = 6.3 Hz, 1H), 5.00 (q, J = 6.9 Hz, 1H), 4.68–4.59 (m, 2H), 4.44 (s, 1H), 3.95–3.83 (m, 3H), 3.76 (dd, J = 11.0, 3.9 Hz, 1H), 3.33–3.25 (m, 4H), 2.45 (s, 3H), 2.29–2.12 (m, 5H), 2.05–1.92 (m, 1H), 1.86–1.74 (m, 2H), 1.62–1.50 (m, 4H), 1.47 (d, J = 7.0 Hz, 3H), 1.30 –1.20 (m, 8H), 1.05 (s, 9H). ^{13}C NMR (101 MHz, CD_3OD) δ 174.97, 174.67, 171.88, 171.01, 161.61, 157.95, 151.40, 147.62, 146.81, 144.19, 142.79, 142.25, 133.93, 131.93, 130.08, 129.61, 129.06, 127.05, 126.85, 126.19, 125.94, 125.24, 122.82, 120.55, 116.63, 110.73, 106.79, 69.57, 64.46, 59.25, 57.62, 56.65, 55.32, 48.79, 38.86, 37.40, 36.64, 35.86, 35.20, 35.10, 28.86, 28.83, 28.81, 28.76, 25.76, 25.69, 25.57, 21.09, 14.50. HRMS (m/z): calculated for $C_{54}H_{69}Cl_2N_9O_7S$ $[M+H]^+$: 1058.4490, found 1058.4498. Purity: 99.3%.

*4.1.2.3. (S,E)-4-(5-chloro-2-((3-(7-(2,6-dimethoxy-4-(3-oxo-3-(6-oxo-3,6-dihydropyridin-1(2H)-yl)prop-1-en-1-yl)phenoxy)heptanamido)propyl)amino)pyridin-4-yl)-N-(1-(3-chlorophenyl)-2-hydroxyethyl)-1H-pyrrole-2-carboxamide (**A3**)*

White solid; m.p. 103-104℃; ^{1}H NMR (400 MHz, $CDCl_3$) δ 10.94 (s, 1H), 8.11 (s, 1H), 7.90 (s, 1H), 7.64 (d, J = 15.5 Hz, 1H), 7.42–7.34 (m, 2H), 7.28–7.18 (m, 2H), 7.13 (s, 2H), 7.05 (s, 1H), 6.92 (dt, 1H), 6.84 (s, 1H), 6.74 (s, 2H), 6.25 (s, 1H), 6.01 (d, J = 9.7 Hz, 1H), 5.29 (s, 1H), 5.22 (s, 1H), 4.73 (s, 1H), 4.06 – 3.87 (m, 6H), 3.79 (s, 6H), 3.36 –3.17 (m, 4H), 2.45 (d, J = 5.7 Hz, 2H), 2.19 (t, J = 7.7 Hz, 2H), 1.72–1.55 (m, 6H), 1.48–1.28 (m, 4H). ^{13}C NMR (101 MHz, $CDCl_3$) δ 174.51, 168.99, 165.94, 161.59, 157.46, 153.47, 147.47, 145.77, 143.82, 142.05, 141.58, 139.18, 134.32, 130.39, 129.84, 127.60, 126.88, 125.83, 125.65, 125.20, 123.27, 120.95, 120.68, 117.06, 110.47, 106.63, 105.59, 73.38, 65.29, 56.12, 55.87, 41.71, 38.48, 36.66, 29.85, 29.30, 28.96, 25.80, 25.48, 24.77. HRMS (m/z): calculated for $C_{44}H_{50}Cl_2N_6O_8$ $[M+H]^+$: 861.3140, found 861.3131. Purity: 98.0%.

4.1.2.4. (S)-N1-(adamantan-1-yl)-N10-(3-((5-chloro-4-(5-((1-(3-chlorophenyl)-2-

*hydroxyethyl)carbamoyl)-1H-pyrrol-3-yl)pyridin-2-yl)amino)propyl)decanediamide (**A4**)*

White solid; m.p. 124-125°C; ^{1}H NMR (400 MHz, CD_3OD) δ 7.92 (s, 1H), 7.49 (d, J = 1.5 Hz, 1H), 7.46 (s, 1H), 7.40 (d, J = 1.6 Hz, 1H), 7.37–7.28 (m, 2H), 7.27–7.23 (m, 1H), 6.65 (s, 1H), 5.18 (t, J = 6.4 Hz, 1H), 3.88 (d, J = 6.4 Hz, 2H), 3.37 (s, 2H), 3.33 –3.25 (m, 4H), 2.18 (t, J = 7.5 Hz, 2H), 2.07 (t, J = 7.5 Hz, 2H), 1.99 (s, 9H), 1.85–1.73 (m, 2H), 1.67 (s, 6H), 1.63–1.47 (m, 4H), 1.27 (s, 8H). ^{13}C NMR (101 MHz, CD_3OD) δ 174.98, 174.13, 161.63, 157.92, 146.72, 142.72, 142.29, 133.96, 129.60, 127.06, 126.85, 125.95, 125.22, 122.72, 120.59, 116.65, 110.76, 106.84, 64.43, 55.29, 51.28, 40.97, 38.81, 36.61, 36.12, 35.84, 29.49, 28.90, 28.85, 28.80, 28.75, 25.78, 25.66. HRMS (m/z): calculated for $C_{41}H_{54}Cl_2N_6O_4$ $[M+H]^+$: 764.3656, found 765.3654. Purity: 99.2%.

*4.1.2.5. N-(4-(4-((4-(1-((S)-1-(4-chloro-3-fluorophenyl)-2-hydroxyethyl)-2-oxo-1,2-dihydropyridin-4-yl)pyrimidin-2-yl)amino)pyridin-2-yl)but-3-yn-1-yl)-8-((2-(2,6-dioxopiperidin-3-yl)-1,3-dioxoisoindolin-4-yl)amino)octanamide (**C1**)*

Yellow solid; m.p. 140-141°C; ^{1}H NMR (400 MHz, DMSO-*d*6) δ 11.10 (s, 1H), 10.38 (s, 1H), 8.73 (d, J = 5.2 Hz, 1H), 8.33 (d, J = 5.7 Hz, 1H), 8.06 (t, J = 5.7 Hz, 1H), 8.02 –7.95 (m, 2H), 7.76–7.69 (m, 1H), 7.64–7.40 (m, 4H), 7.25 (d, J = 2.0 Hz, 1H), 7.19 (d, J = 8.4 Hz, 1H), 7.03–6.93 (m, 3H), 6.43 (t, J = 5.9 Hz, 1H), 6.02 (t, J = 6.4 Hz, 1H), 5.37 (s, 1H), 5.05 (dd, J = 12.9, 5.4 Hz, 1H), 4.26–4.15 (m, 1H), 4.13–4.05 (m, 1H), 3.29 (q, J = 6.6 Hz, 2H), 3.19 (q, J = 6.7 Hz, 2H), 2.96 – 2.81 (m, 1H), 2.65–2.51 (m, 4H), 2.10–2.00 (m, 3H), 1.48 (t, J = 7.0 Hz, 4H), 1.22 (s, 6H). 13C NMR (101 MHz, DMSO-*d*6) δ 173.24, 172.87, 170.54, 169.39, 167.73, 162.21, 161.55, 160.45, 159.98, 158.82, 156.37, 150.37, 147.77, 146.98, 146.81, 143.38, 140.08, 140.01, 137.59, 136.66, 132.60, 131.20, 125.56, 125.53, 119.39, 119.22, 118.06, 117.48, 116.74, 116.52, 116.07, 112.30, 111.01, 110.80, 109.46, 103.41, 87.98, 82.00, 61.24, 59.35, 49.02, 42.27, 38.02, 35.85, 31.45, 29.12, 29.05, 29.00, 26.65, 25.73, 22.62, 19.93. HRMS (m/z): calculated for $C_{47}H_{45}ClFN_9O_7$ $[M+H]^+$: 902.3187, found 902.3187. Purity:

100.0%.

*4.1.2.6. N1-(4-(4-((4-(1-((S)-1-(4-chloro-3-fluorophenyl)-2-hydroxyethyl)-2-oxo-1,2-dihydropyridin-4-yl)pyrimidin-2-yl)amino)pyridin-2-yl)but-3-yn-1-yl)-N10-((S)-1-((2S,4R)-4-hydroxy-2-(((S)-1-(4-(4-methylthiazol-5-yl)phenyl)ethyl)carbamoyl) pyrrolidin-1-yl)-3,3-dimethyl-1-oxobutan-2-yl)decanediamide (**C2**)*

White solid; m.p. 143-144°C; ^{1}H NMR (400 MHz, CD_3OD) δ 8.83 (s, 1H), 8.60 (d, J = 5.1 Hz, 1H), 8.21 (d, J = 5.9 Hz, 1H), 8.00 (d, J = 2.1 Hz, 1H), 7.90 (d, J = 7.3 Hz, 1H), 7.71–7.62 (m, 1H), 7.45 (t, J = 8.0 Hz, 1H), 7.41–7.30 (m, 7H), 7.21–7.14 (m, 1H), 7.04 –6.99 (m, 1H), 6.19–6.11 (m, 1H), 5.01 (q, J = 6.9 Hz, 1H), 4.66–4.59 (m, 2H), 4.45 (s, 1H), 4.38–4.30 (m, 1H), 4.27–4.19 (m, 1H), 3.91 (d, J = 11.0 Hz, 1H), 3.80–3.71 (m, 1H), 3.43 (t, J = 6.5 Hz, 2H), 2.67 (t, J = 6.5 Hz, 2H), 2.44 (s, 3H), 2.27–2.09 (m, J = 7.5 Hz, 5H), 2.04–1.93 (m, 1H), 1.61–1.43 (m, 7H), 1.32–1.14 (m, 8H), 1.03 (s, 9H). ^{13}C NMR (101 MHz, CD_3OD) δ 175.04, 174.58, 174.50, 171.79, 170.94, 163.39, 161.08, 159.70, 159.62, 159.17, 156.70, 151.38, 149.04, 148.14, 147.76, 147.60, 144.16, 142.94, 138.70, 138.64, 136.64, 131.89, 130.73, 130.06, 129.01, 126.22, 124.58, 124.54, 120.22, 120.04, 117.47, 116.17, 115.73, 111.89, 110.28, 104.50, 87.82, 80.76, 69.59, 61.20, 59.71, 59.23, 57.69, 57.60, 56.66, 37.85, 37.44, 35.78, 35.31, 35.27, 35.10, 28.97, 28.89, 28.87, 28.85, 25.76, 25.57, 21.07, 19.34, 14.56. HRMS (m/z): calculated for $C_{59}H_{68}ClFN_{10}O_7S$ $[M+H]^+$: 1115.4738, found 1115.4748. Purity: 99.3%.

*4.1.2.7. (S,E)-N-(4-(4-((4-(1-(1-(4-chloro-3-fluorophenyl)-2-hydroxyethyl)-2-oxo-1,2-dihydropyridin-4-yl)pyrimidin-2-yl)amino)pyridin-2-yl)but-3-yn-1-yl)-7-(2,6-dimethoxy-4-(3-oxo-3-(6-oxo-3,6-dihydropyridin-1(2H)-yl)prop-1-en-1-yl)phenoxy) heptanamide (**C3**)*

White solid; m.p. 114-115°C; ^{1}H NMR (400 MHz, $CDCl_3$) δ 8.64 (s, 1H), 8.42 (d, J = 5.1 Hz, 1H), 8.09 (d, J = 5.7 Hz, 1H), 7.70 (s, 1H), 7.61 (d, J = 15.6 Hz, 1H), 7.49 – 7.32 (m, 5H), 7.28 (d, J = 10.1 Hz, 1H), 7.16 (d, J = 8.5 Hz, 1H), 7.11–7.02 (m, 2H), 6.98 – 6.89 (m, 1H), 6.72 (s, 2H), 6.56 (d, J = 7.2 Hz, 1H), 6.18 (t, J = 4.5 Hz, 1H), 6.03

(d, J = 9.7 Hz, 1H), 4.39 (s, 2H), 4.01 (t, J = 6.5 Hz, 2H), 3.91 (t, J = 6.6 Hz, 2H), 3.78 (s, 6H), 3.61–3.51 (m, 1H), 3.49–3.37 (m, 1H), 2.68–2.60 (m, 2H), 2.46 (q, J = 4.1 Hz, 2H), 2.25 (t, J = 7.5 Hz, 2H), 1.73–1.60 (m, 4H), 1.48–1.28 (m, 4H). ^{13}C NMR (101 MHz, $CDCl_3$) δ 174.03, 168.88, 165.90, 162.71, 161.15, 159.36, 159.31, 159.16, 156.88, 153.46, 149.73, 147.16, 146.49, 145.72, 143.72, 143.16, 139.24, 138.19, 138.13, 136.77, 131.03, 130.35, 125.68, 124.92, 120.95, 120.78, 117.90, 116.91, 116.70, 115.80, 111.71, 110.02, 105.57, 103.59, 88.45, 81.48, 73.39, 61.45, 59.30, 56.10, 41.68, 38.15, 36.40, 29.92, 29.00, 25.81, 25.53, 24.78, 20.39. HRMS (m/z): calculated for $C_{49}H_{49}ClFN_7O_8$ $[M+H]^+$: 918.3388, found 918.3387. Purity: 99.7%.

*4.1.2.8. (S)-N1-(adamantan-1-yl)-N10-(4-(4-((4-(1-(1-(4-chloro-3-fluorophenyl)-2-hydroxyethyl)-2-oxo-1,2-dihydropyridin-4-yl)pyrimidin-2-yl)amino)pyridin-2-yl)but-3-yn-1-yl)decanediamide (**C4**)*

White solid; m.p. 134-135°C; ^{1}H NMR (400 MHz, CD_3OD) δ 8.61 (d, J = 5.1 Hz, 1H), 8.20 (d, J = 5.9 Hz, 1H), 7.96 (d, J = 2.0 Hz, 1H), 7.89 (d, J = 7.3 Hz, 1H), 7.72–7.65 (m, 1H), 7.46 (t, J = 8.0 Hz, 1H), 7.39 (d, J = 5.1 Hz, 1H), 7.36–7.29 (m, 2H), 7.20 (s, 1H), 7.06–6.99 (m, 1H), 6.19–6.10 (m, 1H), 4.39–4.29 (m, 1H), 4.28–4.19 (m, 1H), 3.43 (t, J = 6.6 Hz, 2H), 2.66 (t, J = 6.5 Hz, 2H), 2.19 (t, J = 7.4 Hz, 2H), 2.06–1.94 (m, 11H), 1.65 (d, J = 2.8 Hz, 6H), 1.62–1.53 (m, 2H), 1.52–1.41 (m, 2H), 1.32–1.16 (m, 8H). ^{13}C NMR (101 MHz, CD_3OD) δ 175.07, 174.12, 174.04, 163.40, 161.08, 159.73, 159.57, 159.18, 156.71, 148.98, 148.16, 147.80, 142.91, 138.69, 138.62, 136.57, 130.72, 124.54, 124.51, 120.23, 120.06, 117.42, 116.15, 115.93, 115.69, 111.80, 110.22, 104.51, 87.73, 80.69, 61.18, 59.69, 51.33, 47.64, 47.42, 47.21, 47.00, 40.98, 37.85, 36.63, 36.57, 36.11, 35.74, 29.48, 28.95, 28.88, 28.83, 28.76, 25.75, 25.70, 19.34. HRMS (m/z): calculated for $C_{46}H_{53}ClFN_7O_4$ $[M+H]^+$: 822.3904, found 822.3913. Purity: 99.7%.

4.1.2.9. 4-((2-(2,6-dioxopiperidin-3-yl)-1,3-dioxoisoindolin-4-yl)amino)-N-(4-(4-((5-methyl-4-((R)-3-methyl-2-((6-methylpyridin-2-yl)methyl)-1-oxo-1,2,3,4-

*tetrahydropyrrolo[1,2-a]pyrazin-7-yl)pyrimidin-2-yl)amino)pyridin-2-yl)but-3-yn-1-yl)butanamide (**B1-4**)*

Yellow solid; m.p. 156-157°C; ^{1}H NMR (400 MHz, DMSO-*d6*) δ 11.11 (s, 1H), 10.02 (s, 1H), 8.37 (s, 1H), 8.27 (d, J = 5.8 Hz, 1H), 8.14 (t, J = 5.8 Hz, 1H), 8.04 (s, 1H), 7.75 (s, 1H), 7.72–7.62 (m, 2H), 7.55–7.47 (m, 1H), 7.39 (s, 1H), 7.21–7.11 (m, 2H), 7.04 (d, J = 8.7 Hz, 1H), 6.98 (d, J = 7.0 Hz, 1H), 6.57 (t, J = 6.1 Hz, 1H), 5.17 (d, J = 16.0 Hz, 1H), 5.09–5.00 (m, 1H), 4.46–4.37 (m, 1H), 4.31 (d, J = 16.0 Hz, 1H), 4.24 (d, J = 10.8 Hz, 1H), 4.03–3.96 (m, 1H), 3.32–3.23 (m, 4H), 2.95–2.81 (m, 1H), 2.63 (t, J = 7.0 Hz, 4H), 2.46 (s, 3H), 2.37 (s, 3H), 2.19 (t, J=7.1 Hz, 2H), 2.07–1.98 (m, 1H), 1.86–1.75 (m, 2H), 1.19 (d, J = 6.6 Hz, 3H). ^{13}C NMR (101 MHz, DMSO-*d6*) δ 173.27, 172.29, 170.56, 169.26, 167.73, 160.01, 159.02, 158.06, 158.01, 157.86, 157.37, 149.77, 148.64, 146.72, 142.72, 137.66, 136.59, 132.60, 126.87, 124.93, 123.12, 122.05, 118.75, 118.72, 117.50, 115.53, 112.93, 111.79, 110.80, 109.50, 88.26, 81.72, 52.10, 49.38, 49.21, 48.99, 41.95, 38.09, 32.94, 31.45, 25.19, 24.47, 22.64, 19.97, 17.62, 17.48. HRMS (m/z): calculated for $C_{46}H_{45}N_{11}O_6$ $[M+H]^+$: 848.3627, found 848.3628. Purity: 95.8%.

*4.1.2.10. 6-((2-(2,6-dioxopiperidin-3-yl)-1,3-dioxoisoindolin-4-yl)amino)-N-(4-(4-((5-methyl-4-((R)-3-methyl-2-((6-methylpyridin-2-yl)methyl)-1-oxo-1,2,3,4-tetrahydropyrrolo[1,2-a]pyrazin-7-yl)pyrimidin-2-yl)amino)pyridin-2-yl)but-3-yn-1-yl)hexanamide (**B1-6**)*

Yellow solid; m.p. 149-150°C; ^{1}H NMR (400 MHz, DMSO-*d6*) δ 11.12 (s, 1H), 9.94 (s, 1H), 8.35 (s, 1H), 8.27 (d, J = 5.7 Hz, 1H), 8.10–8.02 (m, 2H), 7.74 (s, 1H), 7.71–7.60 (m, 2H), 7.52 (t, J = 7.8 Hz, 1H), 7.40 (s, 1H), 7.21–7.10 (m, 2H), 7.01–6.93 (m, 2H), 6.43 (t, J = 5.8 Hz, 1H), 5.17 (d, J = 16.0 Hz, 1H), 5.10–5.00 (m, 1H), 4.46–4.36 (m, 1H), 4.36–4.19 (m, 2H), 4.05–3.93 (m, 1H), 3.29 (q, J = 6.7 Hz, 2H), 3.19 (q, J = 6.7 Hz, 2H), 2.95–2.81 (m, 1H), 2.66–2.51 (m, 4H), 2.46 (s, 3H), 2.36 (s, 3H), 2.13–1.99 (m, 3H), 1.60–1.45 (m, 4H), 1.36–1.25 (m, 2H), 1.19 (d, J = 6.6 Hz, 3H). ^{13}C NMR (101 MHz, DMSO-*d6*) δ 173.27, 172.68, 170.57, 169.37, 167.74, 160.02, 158.96,

158.17, 158.03, 157.88, 157.38, 150.37, 148.20, 146.77, 143.48, 137.63, 136.62, 132.57, 126.84, 124.90, 123.18, 122.04, 118.74, 118.49, 117.46, 115.53, 112.95, 111.79, 110.78, 109.42, 87.39, 82.30, 52.11, 49.38, 49.22, 49.01, 42.17, 38.09, 35.77, 31.46, 28.93, 26.37, 25.52, 24.48, 22.64, 19.93, 17.62, 17.47. HRMS (m/z): calculated for $C_{48}H_{49}N_{11}O_6$ $[M+H]^+$: 876.3940, found 876.3935. Purity: 95.3%.

*4.1.2.11. 8-((2-(2,6-dioxopiperidin-3-yl)-1,3-dioxoisoindolin-4-yl)amino)-N-(4-(4-((5-methyl-4-((R)-3-methyl-2-((6-methylpyridin-2-yl)methyl)-1-oxo-1,2,3,4-tetrahydropyrrolo[1,2-a]pyrazin-7-yl)pyrimidin-2-yl)amino)pyridin-2-yl)but-3-yn-1-yl)octanamide (**B1-8**)*

Yellow solid; m.p. 159-160℃; ^{1}H NMR (400 MHz, DMSO-*d*6) δ 11.11 (s, 1H), 9.95 (s, 1H), 8.36 (s, 1H), 8.27 (d, J = 5.7 Hz, 1H), 8.08–7.98 (m, 2H), 7.75 (d, J = 1.5 Hz, 1H), 7.71–7.61 (m, 2H), 7.54 (t, J = 7.9 Hz, 1H), 7.40 (d, J = 1.7 Hz, 1H), 7.21–7.11 (m, 2H), 7.04–6.96 (m, 2H), 6.44 (t, J = 5.9 Hz, 1H), 5.17 (d, J = 16.0 Hz, 1H), 5.10–5.01 (m, 1H), 4.45– .36 (m, 1H), 4.31 (d, J = 16.0 Hz, 1H), 4.23 (d, J = 10.8 Hz, 1H), 4.05–3.93 (m, 1H), 3.28 (q, J = 6.6 Hz, 2H), 3.19 (q, J = 6.7 Hz, 2H), 2.96–2.81 (m, 1H), 2.67–2.51 (m, 4H), 2.46 (s, 3H), 2.37 (s, 3H), 2.10–1.96 (m, 3H), 1.53–1.41 (m, 4H), 1.29–1.14 (m, 9H). ^{13}C NMR (101 MHz, DMSO-*d*6) δ 173.25, 172.75, 170.55, 169.39, 167.74, 160.03, 158.97, 158.19, 158.02, 157.87, 157.38, 150.35, 148.20, 146.81, 143.49, 137.62, 136.66, 132.60, 126.84, 124.90, 123.18, 122.03, 118.75, 118.48, 117.49, 115.54, 112.93, 111.78, 110.78, 109.44, 87.37, 82.30, 52.09, 49.37, 49.22, 49.01, 42.27, 38.05, 35.87, 31.45, 29.13, 29.08, 29.01, 26.66, 25.75, 24.48, 22.62, 19.91, 17.61, 17.46. HRMS (m/z): calculated for $C_{50}H_{53}N_{11}O_6$ $[M+H]^+$: 904.4253, found 904.4238. Purity: 99.0%.

*4.1.2.12. 10-((2-(2,6-dioxopiperidin-3-yl)-1,3-dioxoisoindolin-4-yl)amino)-N-(4-(4-((5-methyl-4-((R)-3-methyl-2-((6-methylpyridin-2-yl)methyl)-1-oxo-1,2,3,4-tetrahydropyrrolo[1,2-a]pyrazin-7-yl)pyrimidin-2-yl)amino)pyridin-2-yl)but-3-yn-1-yl)decanamide (**B1-10**)*

Yellow solid; m.p. 140-141°C; ^{1}H NMR (400 MHz, DMSO-**d**6) δ 11.12 (s, 1H), 9.95 (s, 1H), 8.36 (s, 1H), 8.27 (d, J = 5.7 Hz, 1H), 8.05 (s, 1H), 8.00 (t, J = 5.7 Hz, 1H), 7.75 (s, 1H), 7.71–7.61 (m, 2H), 7.59–7.50 (m, 1H), 7.41 (s, 1H), 7.21–7.11 (m, 2H), 7.01 (t, J = 7.8 Hz, 2H), 6.46 (t, J = 5.9 Hz, 1H), 5.17 (d, J = 16.0 Hz, 1H), 5.11–5.01 (m, 1H), 4.45–4.36 (m, 1H), 4.31 (d, J = 16.0 Hz, 1H), 4.23 (d, J = 10.7 Hz, 1H), 4.03 –3.96 (m, 1H), 3.33–3.16 (m, 4H), 2.96–2.82 (m, 1H), 2.65–2.55 (m, 4H), 2.46 (s, 3H), 2.37 (s, 3H), 2.04 (t, J = 7.4 Hz, 3H), 1.53–1.41 (m, 4H), 1.27–1.10 (m, 13H). ^{13}C NMR (101 MHz, DMSO-*d*6) δ 173.26, 172.77, 170.55, 169.40, 167.75, 160.04, 158.97, 158.21, 158.02, 157.87, 157.38, 150.35, 148.19, 146.83, 143.51, 137.62, 136.68, 132.61, 126.84, 124.90, 123.20, 122.03, 118.75, 118.46, 117.53, 115.54, 112.95, 111.78, 110.80, 109.44, 87.34, 82.32, 52.10, 49.37, 49.22, 49.01, 42.29, 38.04, 35.93, 31.46, 29.35, 29.27, 29.23, 29.14, 29.11, 26.77, 25.82, 24.48, 22.63, 19.91, 17.62, 17.46. HRMS (m/z): calculated for $C_{52}H_{57}N_{11}O_6$ $[M+H]^+$: 932.4566, found 932.4567. Purity: 95.2%.

*4.1.2.13. 12-((2-(2,6-dioxopiperidin-3-yl)-1,3-dioxoisoindolin-4-yl)amino)-N-(4-(4-((5-methyl-4-((R)-3-methyl-2-((6-methylpyridin-2-yl)methyl)-1-oxo-1,2,3,4-tetrahydropyrrolo[1,2-a]pyrazin-7-yl)pyrimidin-2-yl)amino)pyridin-2-yl)but-3-yn-1-yl)dodecanamide (**B1-12**)*

Yellow solid; m.p. =146-147°C; ^{1}H NMR (400 MHz, DMSO-*d*6) δ 11.12 (s, 1H), 10.01 (s, 1H), 8.38 (s, 1H), 8.27 (d, J = 5.8 Hz, 1H), 8.07 (s, 1H), 8.01 (t, J = 5.7 Hz, 1H), 7.76 (s, 1H), 7.72–7.61 (m, 2H), 7.60–7.51 (m, 1H), 7.41 (d, J = 1.7 Hz, 1H), 7.21–7.11 (m, 2H), 7.07–6.97 (m, 2H), 6.48 (t, J = 5.9 Hz, 1H), 5.17 (d, J = 16.0 Hz, 1H), 5.11–5.01 (m, 1H), 4.45–4.36 (m, 1H), 4.31 (d, J = 16.0 Hz, 1H), 4.23 (d, J = 13.1 Hz, 1H), 4.04–3.93 (m, 1H), 3.30–3.20 (m, 4H), 2.96–2.82 (m, 1H), 2.66–2.51 (m, 4H), 2.46 (s, 3H), 2.38 (s, 3H), 2.04 (t, J = 7.4 Hz, 3H), 1.57–1.39 (m, 4H), 1.27–1.12 (m, 17H). ^{13}C NMR (101 MHz, DMSO-*d*6) δ 173.26, 172.79, 170.55, 169.41, 167.75, 160.04, 158.99, 158.16, 158.02, 157.86, 157.37, 149.98, 148.46, 146.85, 143.10, 137.64, 136.69, 132.62, 126.86, 124.91, 123.17, 122.04, 118.76, 118.58, 117.56, 115.55,

112.96, 111.79, 110.81, 109.44, 87.87, 81.96, 52.11, 49.38, 49.22, 49.01, 42.30, 38.01, 35.93, 31.45, 29.46, 29.37, 29.31, 29.24, 29.14, 29.12, 26.79, 25.82, 24.47, 22.63, 19.92, 17.63, 17.45. HRMS (m/z): calculated for $C_{54}H_{61}N_{11}O_6$ $[M+H]^+$: 960.4879, found 960.4887. Purity: 95.0%.

*4.1.2.14. 2-(2-(2-(2-((2-(2,6-dioxopiperidin-3-yl)-1,3-dioxoisoindolin-4-yl)amino)ethoxy)ethoxy)ethoxy)-N-(4-(4-((5-methyl-4-((R)-3-methyl-2-((6-methylpyridin-2-yl)methyl)-1-oxo-1,2,3,4-tetrahydropyrrolo[1,2-a]pyrazin-7-yl)pyrimidin-2-yl)amino)pyridin-2-yl)but-3-yn-1-yl)acetamide (**B1-3P**)*

Yellow solid; m.p. 133-134°C; ^{1}H NMR (400 MHz, CD_3OD) δ 8.24 (s, 1H), 8.19 (d, J = 5.9 Hz, 1H), 7.98 (s, 1H), 7.76–7.67 (m, 2H), 7.64 (s, 1H), 7.56 (s, 1H), 7.41 (t, J = 8.5 Hz, 1H), 7.29 (d, J = 7.8 Hz, 1H), 7.21 (d, J = 7.7 Hz, 1H), 6.95–6.87 (m, 2H), 5.30 (d, J = 15.8 Hz, 1H), 5.04–4.99 (m, 1H), 4.61 (s, 1H), 4.46–4.35 (m, 2H), 4.18 (d, J = 11.4 Hz, 1H), 4.07–3.97 (m, 3H), 3.71–3.57 (m, 9H), 3.52 (t, J = 6.7 Hz, 2H), 3.42–3.35 (m, 2H), 2.91–2.64 (m, 5H), 2.55 (s, 3H), 2.38 (s, 3H), 2.15–2.06 (m, 1H), 1.28 (d, J = 6.6 Hz, 3H). ^{13}C NMR (101 MHz, CD_3OD) δ 173.26, 171.62, 170.29, 169.16, 167.78, 159.27, 159.20, 158.87, 157.94, 157.58, 156.60, 148.91, 148.71, 146.54, 142.66, 137.81, 135.65, 132.28, 126.65, 124.00, 123.78, 122.15, 118.93, 118.68, 116.64, 115.39, 113.70, 111.36, 110.52, 109.99, 109.73, 87.21, 80.96, 70.70, 70.18, 70.05, 69.79, 69.07, 52.40, 48.92, 48.78, 41.73, 37.40, 30.81, 22.60, 22.44, 19.27, 16.43, 15.96. HRMS (m/z): calculated for $C_{50}H_{53}N_{11}O_9$ $[M+Na]^+$: 974.3920, found 974.3928. Purity: 96.0%.

*4.1.2.15. 14-((2-(2,6-dioxopiperidin-3-yl)-1,3-dioxoisoindolin-4-yl)amino)-N-(4-(4-((5-methyl-4-((R)-3-methyl-2-((6-methylpyridin-2-yl)methyl)-1-oxo-1,2,3,4-tetrahydropyrrolo[1,2-a]pyrazin-7-yl)pyrimidin-2-yl)amino)pyridin-2-yl)but-3-yn-1-yl)-3,6,9,12-tetraoxatetradecanamide (**B1-4P**)*

Yellow solid; m.p. 122-123°C; ^{1}H NMR (400 MHz, CD_3OD) δ 8.27 (s, 1H), 8.21 (d, J = 6.0 Hz, 1H), 8.00 (s, 1H), 7.78–7.63 (m, 3H), 7.56 (d, J = 1.7 Hz, 1H), 7.43 (t, J = 7.8

Hz, 1H), 7.29 (d, J = 7.7 Hz, 1H), 7.21 (d, J = 7.7 Hz, 1H), 6.98 – 6.90 (m, 2H), 5.30 (d, J = 15.8 Hz, 1H), 5.06–4.96 (m, 1H), 4.47–4.36 (m, 2H), 4.19 (d, J = 13.1 Hz, 1H), 4.04 (s, 1H), 3.99 (s, 2H), 3.68–3.51 (m, 15H), 3.43–3.37 (m, 2H), 2.87–2.61 (m, 5H), 2.56 (s, 3H), 2.40 (s, 3H), 2.14–2.05 (m, 1H), 1.28 (d, J = 6.6 Hz, 3H). ^{13}C NMR (101 MHz, CD_3OD) δ 173.22, 171.61, 170.24, 169.16, 167.77, 159.31, 159.31, 159.19, 158.92, 157.94, 157.55, 156.60, 149.07, 148.47, 146.60, 142.20, 137.79, 135.66, 132.32, 126.66, 124.03, 123.75, 122.14, 118.93, 118.85, 116.67, 115.39, 113.69, 111.34, 110.52, 109.75, 70.64, 70.22, 70.18, 70.13, 69.90, 69.78, 69.13, 52.40, 48.92, 48.78, 41.79, 37.37, 30.81, 22.60, 22.43, 19.30, 16.42, 15.95. HRMS (m/z): calculated for $C_{52}H_{57}N_{11}O_{10}$ $[M+H]^+$: 996.4363, found 996.4363. Purity: 98.2%.

*4.1.2.16. 17-((2-(2,6-dioxopiperidin-3-yl)-1,3-dioxoisoindolin-4-yl)amino)-N-(4-(4-((5-methyl-4-((R)-3-methyl-2-((6-methylpyridin-2-yl)methyl)-1-oxo-1,2,3,4-tetrahydropyrrolo[1,2-a]pyrazin-7-yl)pyrimidin-2-yl)amino)pyridin-2-yl)but-3-yn-1-yl)-3,6,9,12,15-pentaoxaheptadecanamide (**B1-5P**)*

Yellow solid; m.p. 116-117°C; ^{1}H NMR (400 MHz, CD_3OD) δ 8.26 (s, 1H), 8.21 (d, J = 5.9 Hz, 1H), 7.99 (s, 1H), 7.76–7.63 (m, 3H), 7.56 (s, 1H), 7.45 (t, J = 7.8 Hz, 1H), 7.28 (d, J = 7.8 Hz, 1H), 7.20 (d, J = 7.7 Hz, 1H), 6.95 (d, J = 7.5 Hz, 2H), 5.30 (d, J = 15.8 Hz, 1H), 5.07–4.96 (m, 1H), 4.47–4.35 (m, 2H), 4.19 (d, J = 13.1 Hz, 1H), 4.02 (d, J = 18.2 Hz, 3H), 3.70–3.47 (m, 19H), 3.40 (t, J = 5.7 Hz, 2H), 2.90–2.65 (m, 5H), 2.55 (s, 3H), 2.39 (s, 3H), 2.14–2.05 (m, 1H), 1.28 (d, J = 6.6 Hz, 3H). ^{13}C NMR (101 MHz, CD_3OD) δ 173.24, 171.55, 170.21, 169.16, 167.78, 159.29, 159.18, 158.91, 157.94, 157.61, 156.61, 148.87, 148.78, 146.61, 142.61, 137.80, 135.68, 132.33, 126.65, 124.02, 123.78, 122.14, 118.93, 118.71, 116.70, 115.40, 113.69, 111.38, 110.54, 109.76, 87.32, 80.89, 70.61, 70.21, 70.15, 70.05, 69.88, 69.78, 69.13, 52.39, 48.91, 48.79, 41.81, 37.41, 30.82, 22.61, 22.45, 19.30, 16.43, 15.97. HRMS (m/z): calculated for $C_{54}H_{61}N_{11}O_{11}$ $[M+H]^+$: 1040.4625, found 1040.4645. Purity: 96.6%.

4.1.2.17. 10-((2-(2,6-dioxopiperidin-3-yl)-1,3-dioxoisoindolin-4-yl)amino)-N-(3-((4-

*((5-methyl-4-((R)-3-methyl-2-((6-methylpyridin-2-yl)methyl)-1-oxo-1,2,3,4-tetrahydropyrrolo[1,2-a]pyrazin-7-yl)pyrimidin-2-yl)amino)pyridin-2-yl)amino)propyl)decanamide (**B1-10N**)*

Yellow solid; m.p. 110-111°C; ^{1}H NMR (400 MHz, DMSO-*d6*) δ 11.14 (s, 1H), 9.73 (s, 1H), 8.33 (s, 1H), 7.85 (s, 1H), 7.78 (s, 2H), 7.71–7.50 (m, 2H), 7.38 (s, 1H), 7.26 (s, 1H), 7.20–7.10 (m, 2H), 7.07–6.94 (m, 2H), 6.91 (d, J = 6.1 Hz, 1H), 6.76 (s, 1H), 6.47 (t, J = 5.9 Hz, 1H), 5.17 (d, J = 16.0 Hz, 1H), 5.11–5.01 (m, 1H), 4.45–4.21 (m, 3H), 4.04–3.93 (m, 1H), 3.33–3.07 (m, 7H), 2.96–2.83 (m, 1H), 2.65–2.54 (m, 1H), 2.46 (s, 3H), 2.37 (s, 3H), 2.03 (t, J = 7.4 Hz, 3H), 1.69 (t, J = 6.7 Hz, 2H), 1.58–1.39 (m, 4H), 1.28–1.15 (m, 13H). ^{13}C NMR (101 MHz, DMSO-d6) δ 173.27, 172.63, 170.55, 169.41, 167.75, 159.92, 158.97, 158.38, 158.06, 157.87, 157.38, 149.61, 146.84, 137.62, 136.68, 132.60, 126.88, 124.83, 123.20, 122.03, 118.71, 118.26, 117.53, 112.99, 110.81, 109.44, 103.94, 94.19, 52.09, 49.33, 49.18, 49.02, 42.30, 36.70, 35.97, 31.45, 29.36, 29.28, 29.21, 29.13, 26.77, 25.80, 24.47, 22.64, 17.59, 17.44. HRMS (m/z): calculated for $C_{51}H_{60}N_{12}O_6$ $[M+H]^+$: 937.4832, found 937.4848. Purity: 99.1%.

*4.1.2.18. 10-((2-(2,6-dioxopiperidin-3-yl)-1,3-dioxoisoindolin-4-yl)amino)-N-(4-(3-((5-methyl-4-((R)-3-methyl-2-((6-methylpyridin-2-yl)methyl)-1-oxo-1,2,3,4-tetrahydropyrrolo[1,2-a]pyrazin-7-yl)pyrimidin-2-yl)amino)phenyl)but-3-yn-1-yl)decanamide (**B1-10B**)*

Yellow solid; m.p. 88-89°C; ^{1}H NMR (400 MHz, DMSO-*d6*) δ 11.13 (s, 1H), 9.45 (s, 1H), 8.28 (s, 1H), 8.07 (s, 1H), 7.99 (t, J = 5.8 Hz, 1H), 7.79–7.69 (m, 2H), 7.65 (t, J = 7.7 Hz, 1H), 7.55 (t, J = 7.8 Hz, 1H), 7.42 (s, 1H), 7.24 (t, J = 7.9 Hz, 1H), 7.21–7.11 (m, 2H), 7.05–6.97 (m, 2H), 6.94 (d, J = 7.5 Hz, 1H), 6.46 (t, J = 5.9 Hz, 1H), 5.18 (d, J = 15.9 Hz, 1H), 5.12–5.02 (m, 1H), 4.45–4.36 (m, 1H), 4.31 (d, J = 16.0 Hz, 1H), 4.22 (d, J = 13.1 Hz, 1H), 4.04–3.93 (m, 1H), 3.32–3.18 (m, 4H), 2.97–2.83 (m, 1H), 2.67–2.52 (m, 4H), 2.47 (s, 3H), 2.34 (s, 3H), 2.05 (t, J = 7.4 Hz, 3H), 1.56–1.41 (m, 4H), 1.27–1.17 (m, 13H). ^{13}C NMR (101 MHz, DMSO-*d6*) δ 173.26, 172.79, 170.55, 169.42, 167.76, 159.96, 158.75, 158.69, 158.09, 157.84, 157.36, 146.84, 141.75, 137.67,

136.67, 132.60, 129.03, 126.61, 124.74, 123.95, 123.62, 123.58, 122.06, 121.17, 118.77, 118.33, 117.51, 116.95, 113.03, 110.80, 109.45, 88.04, 82.11, 54.07, 52.11, 49.35, 49.18, 49.02, 42.31, 38.27, 35.97, 31.47, 29.36, 29.28, 29.24, 29.15, 29.12, 26.79, 25.86, 24.43, 22.65, 20.14, 18.52, 17.58, 17.43, 17.15. HRMS (m/z): calculated for $C_{53}H_{58}N_{10}O_6$ $[M+Na]^+$: 953.4433, found 953.4450. Purity: 97.7%.

*4.1.2.19. 10-((2-(2,6-dioxopiperidin-3-yl)-1,3-dioxoisoindolin-4-yl)amino)-N-(4-(3-((5-methyl-4-((R)-3-methyl-2-((6-methylpyridin-2-yl)methyl)-1-oxo-1,2,3,4-tetrahydropyrrolo[1,2-a]pyrazin-7-yl)pyrimidin-2-yl)amino)-1H-pyrazol-1-yl)butyl)decanamide (**B1-10BZ**)*

Yellow solid; m.p. 96-97°C; ^{1}H NMR (400 MHz, DMSO-*d*6) δ 11.13 (s, 1H), 10.38 (s, 1H), 8.21 (s, 1H), 7.89–7.72 (m, 3H), 7.66 (t, J = 7.7 Hz, 1H), 7.56 (t, J = 7.7 Hz, 1H), 7.42 (s, 1H), 7.17 (t, J = 8.8 Hz, 2H), 7.06 (d, J = 8.6 Hz, 1H), 7.01 (d, J = 7.1 Hz, 1H), 6.50 (t, J = 6.0 Hz, 1H), 6.40 (s, 1H), 5.17 (d, J = 16.0 Hz, 1H), 5.11–5.02 (m, 1H), 4.41 (d, J = 9.2 Hz, 1H), 4.36–4.21 (m, 2H), 4.16 (s, 2H), 4.05–3.93 (m, 1H), 3.26 (q, J = 6.1, 5.5 Hz, 2H), 3.06 (q, J = 5.8, 5.0 Hz, 2H), 2.89 (t, J = 16.0 Hz, 1H), 2.65–2.53 (m, 2H), 2.47 (s, 3H), 2.39 (s, 3H), 2.03 (t, J = 7.4 Hz, 3H), 1.89–1.80 (m, 2H), 1.60–1.41 (m, 4H), 1.39–1.17 (m, 15H). ^{13}C NMR (101 MHz, DMSO-*d*6) δ 173.29, 172.46, 170.58, 169.41, 167.76, 157.91, 157.88, 157.36, 148.58, 146.85, 137.66, 136.71, 132.62, 125.06, 122.06, 118.71, 117.58, 113.26, 110.81, 109.43, 95.76, 52.09, 51.30, 49.40, 49.20, 49.00, 42.30, 40.59, 40.38, 40.17, 39.96, 39.75, 39.54, 39.33, 38.23, 35.91, 31.46, 29.37, 29.20, 29.13, 27.56, 26.77, 26.67, 25.76, 24.49, 22.64, 17.49. HRMS (m/z): calculated for $C_{50}H_{60}N_{12}O_6$ $[M+Na]^+$: 947.4651, found 947.4680. Purity: 99.3%.

*4.1.2.20. N1-((S)-1-((2S,4R)-4-hydroxy-2-(((S)-1-(4-(4-methylthiazol-5-yl)phenyl) ethyl)carbamoyl)pyrrolidin-1-yl)-3,3-dimethyl-1-oxobutan-2-yl)-N4-(4-(4-((5-methyl-4-((R)-3-methyl-2-((6-methylpyridin-2-yl)methyl)-1-oxo-1,2,3,4-tetrahydropyrrolo [1,2-a]pyrazin-7-yl)pyrimidin-2-yl)amino)pyridin-2-yl)but-3-yn-1-yl)succinimide (**B2-4**)*

White solid; m.p. 160-161°C; ^{1}H NMR (400 MHz, CD_3OD) δ 8.85 (s, 1H), 8.23 (s, 1H), 8.20 (d, J = 5.9 Hz, 1H), 7.96 (d, J = 2.2 Hz, 1H), 7.78–7.73 (m, 1H), 7.69 (t, J = 7.7 Hz, 1H), 7.62 (s, 1H), 7.55 (d, J = 1.6 Hz, 1H), 7.40 (s, 4H), 7.26 (d, J = 7.8 Hz, 1H), 7.19 (d, J = 7.6 Hz, 1H), 5.28 (d, J = 15.8 Hz, 1H), 5.00 (q, J = 6.9 Hz, 1H), 4.64 – 4.56 (m, 2H), 4.47–4.35 (m, 3H), 4.17 (d, J = 13.0 Hz, 1H), 4.08–3.97 (m, 1H), 3.87 (d, J = 11.1 Hz, 1H), 3.77–3.68 (m, 1H), 3.49–3.40 (m, 2H), 2.70–2.59 (m, 3H), 2.58–2.49 (m, 6H), 2.46 (s, 3H), 2.37 (s, 3H), 2.25–2.15 (m, 1H), 2.02–1.90 (m, 1H), 1.49 (d, J = 7.0 Hz, 3H), 1.26 (d, J = 6.6 Hz, 3H), 1.03 (s, 9H). ^{13}C NMR (101 MHz, CD_3OD) δ 173.31, 172.95, 171.82, 170.79, 159.27, 159.21, 158.88, 157.94, 157.61, 156.57, 151.39, 148.89, 148.71, 147.61, 144.16, 142.49, 137.79, 131.90, 130.06, 129.03, 126.61, 126.22, 123.96, 123.81, 122.14, 118.91, 118.70, 115.28, 113.74, 111.31, 87.55, 80.62, 69.59, 59.18, 57.71, 56.51, 52.39, 48.93, 48.69, 38.02, 37.40, 35.19, 30.88, 30.66, 25.69, 22.66, 21.01, 19.34, 16.49, 15.99, 14.47. HRMS (m/z): calculated for $C_{56}H_{64}N_{12}O_6S$ $[M+Na]^+$: 1055.4685, found 1055.4702. Purity: 100.0%.

4.1.2.21. N1-((S)-1-((2S,4R)-4-hydroxy-2-(((S)-1-(4-(4-methylthiazol-5-yl)phenyl) ethyl)carbamoyl)pyrrolidin-1-yl)-3,3-dimethyl-1-oxobutan-2-yl)-N6-(4-(4-((5-methyl-4-((R)-3-methyl-2-((6-methylpyridin-2-yl)methyl)-1-oxo-1,2,3,4-tetrahydropyrrolo [1,2-a]pyrazin-7-yl)pyrimidin-2-yl)amino)pyridin-2-yl)but-3-yn-1-yl)adipamide ***(B2-6)***

White solid; m.p. 153-154°C; ^{1}H NMR (400 MHz, CD_3OD) δ 8.85 (s, 1H), 8.26–8.18 (m, 2H), 7.98 (s, 1H), 7.74 (d, J = 6.1 Hz, 1H), 7.69 (t, J = 7.7 Hz, 1H), 7.63 (s, 1H), 7.56 (s, 1H), 7.44–7.35 (m, 4H), 7.26 (d, J = 7.8 Hz, 1H), 7.18 (d, J = 7.7 Hz, 1H), 5.28 (d, J = 15.7 Hz, 1H), 5.00 (q, J = 6.9 Hz, 1H), 4.65–4.55 (m, 2H), 4.46–4.35 (m, 3H), 4.17 (d, J = 13.2 Hz, 1H), 4.07–3.99 (m, 1H), 3.88 (d, J = 11.0 Hz, 1H), 3.78–3.69 (m, 1H), 3.49–3.39 (m, 2H), 2.68 (t, J = 6.6 Hz, 2H), 2.54 (s, 3H), 2.46 (s, 3H), 2.38 (s, 3H), 2.32–2.15 (m, 5H), 1.97 (ddd, J = 13.3, 9.0, 4.5 Hz, 1H), 1.70–1.57 (m, 4H), 1.49 (d, J = 7.0 Hz, 3H), 1.26 (d, J = 6.6 Hz, 3H), 1.03 (s, 9H). ^{13}C NMR (101 MHz, CD_3OD) δ 174.57, 174.21, 174.13, 171.79, 170.91, 170.89, 159.30, 159.21, 158.91, 157.95, 157.65, 156.56, 151.37, 148.89, 148.80, 147.63, 144.15, 142.68, 137.77, 131.90,

130.08, 129.03, 126.61, 126.21, 123.98, 123.83, 122.14, 118.92, 118.69, 115.28, 113.76, 111.35, 87.41, 80.76, 69.58, 59.19, 57.76, 57.66, 56.58, 52.41, 48.96, 48.70, 37.94, 37.39, 35.34, 35.04, 34.86, 25.70, 25.17, 25.10, 22.67, 20.99, 19.33, 16.48, 16.00, 14.47. HRMS (m/z): calculated for $C_{58}H_{68}N_{12}O_6S$ $[M+Na]^+$: 1083.4998, found 1083.5025. Purity: 100.0%.

4.1.2.22. N1-((S)-1-((2S,4R)-4-hydroxy-2-(((S)-1-(4-(4-methylthiazol-5-yl)phenyl) ethyl)carbamoyl)pyrrolidin-1-yl)-3,3-dimethyl-1-oxobutan-2-yl)-N8-(4-(4-((5-methyl-4-((R)-3-methyl-2-((6-methylpyridin-2-yl)methyl)-1-oxo-1,2,3,4-tetrahydropyrrolo [1,2-a]pyrazin-7-yl)pyrimidin-2-yl)amino)pyridin-2-yl)but-3-yn-1-yl)octanediamide ***(B2-8)***

White solid; m.p. 155-156°C; ^{1}H NMR (400 MHz, CD_3OD) δ 8.86 (s, 1H), 8.27 (s, 1H), 8.22 (d, J = 5.9 Hz, 1H), 8.06 (d, J = 2.2 Hz, 1H), 7.76–7.64 (m, 3H), 7.60 (d, J = 1.7 Hz, 1H), 7.46–7.37 (m, 4H), 7.27 (d, J = 7.8 Hz, 1H), 7.20 (d, J = 7.7 Hz, 1H), 5.29 (d, J = 15.8 Hz, 1H), 5.00 (q, J = 7.0 Hz, 1H), 4.65–4.55 (m, 2H), 4.47–4.36 (m, 3H), 4.24 –4.16 (m, 1H), 4.08–4.00 (m, 1H), 3.89 (d, J = 11.1 Hz, 1H), 3.78–3.70 (m, 1H), 3.49 –3.37 (m, 2H), 2.69 (t, J = 6.6 Hz, 2H), 2.54 (s, 3H), 2.47 (s, 3H), 2.40 (s, 3H), 2.28–2.10 (m, 5H), 2.02–1.90 (m, 1H), 1.65–1.46 (m, 7H), 1.35–1.22 (m, 7H), 1.03 (s, 9H). ^{13}C NMR (101 MHz, CD_3OD) δ 174.97, 174.46, 171.82, 170.89, 159.35, 159.23, 158.93, 157.96, 157.68, 156.56, 151.40, 148.88, 148.78, 147.62, 144.19, 142.64, 137.78, 131.91, 130.07, 129.04, 126.65, 126.21, 123.99, 123.84, 122.15, 118.92, 118.72, 115.27, 113.76, 111.38, 87.54, 80.67, 69.57, 59.20, 57.60, 56.59, 52.44, 48.97, 48.71, 37.84, 37.40, 35.68, 35.12, 35.03, 28.54, 25.67, 25.58, 25.39, 22.64, 21.00, 19.29, 16.47, 15.97, 14.44. HRMS (m/z): calculated for $C_{60}H_{72}N_{12}O_6S$ $[M+Na]^+$: 1111.5311, found 1111.5338. Purity: 99.2%.

4.1.2.23. N1-((S)-1-((2S,4R)-4-hydroxy-2-(((S)-1-(4-(4-methylthiazol-5-yl)phenyl) ethyl)carbamoyl)pyrrolidin-1-yl)-3,3-dimethyl-1-oxobutan-2-yl)-N10-(4-(4-((5-methyl-4-((R)-3-methyl-2-((6-methylpyridin-2-yl)methyl)-1-oxo-1,2,3,4-

*tetrahydropyrrolo[1,2-a]pyrazin-7-yl)pyrimidin-2-yl)amino)pyridin-2-yl)but-3-yn-1-yl)decanediamide (**B2-10**)*

White solid; m.p. 144-145℃; ^{1}H NMR (400 MHz, CD_3OD) δ 8.86 (s, 1H), 8.25 (s, 1H), 8.21 (d, J = 5.9 Hz, 1H), 8.14 (d, J = 2.2 Hz, 1H), 7.73–7.63 (m, 3H), 7.59 (d, J = 1.7 Hz, 1H), 7.43–7.37 (m, 4H), 7.26 (d, J = 7.8 Hz, 1H), 7.18 (d, J = 7.7 Hz, 1H), 5.28 (d, J = 15.7 Hz, 1H), 5.01 (q, J = 7.1 Hz, 1H), 4.67–4.58 (m, 2H), 4.48–4.35 (m, 3H), 4.23–4.14 (m, 1H), 4.09–3.99 (m, 1H), 3.90 (d, J = 10.9 Hz, 1H), 3.80–3.71 (m, 1H), 3.44 (t, J = 6.5 Hz, 2H), 2.70 (t, J = 6.4 Hz, 2H), 2.54 (s, 3H), 2.46 (s, 3H), 2.39 (s, 3H), 2.27–2.10 (m, 5H), 2.05–1.91 (m, 1H), 1.61–1.45 (m, 7H), 1.30–1.14 (m, 11H), 1.05 (s, 9H). ^{13}C NMR (101 MHz, CD_3OD) δ 174.98, 174.53, 171.79, 170.95, 159.32, 159.19, 158.89, 157.94, 157.66, 156.58, 151.38, 148.80, 147.62, 144.17, 142.76, 137.75, 131.90, 130.08, 129.03, 126.63, 126.22, 124.01, 123.85, 122.13, 118.93, 118.65, 115.29, 113.80, 111.47, 87.47, 80.84, 69.57, 59.23, 57.62, 56.64, 52.45, 49.00, 48.93, 48.71, 37.82, 37.42, 35.83, 35.28, 35.09, 28.98, 28.89, 28.86, 25.73, 25.58, 22.68, 21.00, 19.25, 16.53, 16.03, 14.49. HRMS (m/z): calculated for $C_{62}H_{76}N_{12}O_6S$ $[M+Na]^+$: 1139.5624, found 1139.5639. Purity: 99.4%.

*4.1.2.24. N1-((S)-1-((2S,4R)-4-hydroxy-2-(((S)-1-(4-(4-methylthiazol-5-yl)phenyl)ethyl)carbamoyl)pyrrolidin-1-yl)-3,3-dimethyl-1-oxobutan-2-yl)-N12-(4-(4-((5-methyl-4-((R)-3-methyl-2-((6-methylpyridin-2-yl)methyl)-1-oxo-1,2,3,4-tetrahydropyrrolo[1,2-a]pyrazin-7-yl)pyrimidin-2-yl)amino)pyridin-2-yl)but-3-yn-1-yl)dodecanediamide (**B2-12**)*

White solid; m.p. 143-144°C; ^{1}H NMR (400 MHz, CD_3OD) δ 8.86 (s, 1H), 8.27 (s, 1H), 8.22 (d, J = 6.0 Hz, 1H), 8.17 (d, J = 2.2 Hz, 1H), 7.76–7.64 (m, 3H), 7.61 (d, J = 1.7 Hz, 1H), 7.46–7.37 (m, 4H), 7.27 (d, J = 7.7 Hz, 1H), 7.19 (d, J = 7.7 Hz, 1H), 5.29 (d, J = 15.7 Hz, 1H), 5.01 (q, J = 6.9 Hz, 1H), 4.69–4.57 (m, 2H), 4.49–4.35 (m, 3H), 4.25–4.16 (m, 1H), 4.08–4.01 (m, 1H), 3.94–3.86 (m, 1H), 3.81–3.72 (m, 1H), 3.44 (t, J = 6.4 Hz, 2H), 2.71 (t, J = 6.4 Hz, 2H), 2.54 (s, 3H), 2.47 (s, 3H), 2.41 (s, 3H), 2.29–2.10 (m, 5H), 2.05–1.92 (m, 1H), 1.63–1.44 (m, 7H), 1.31–1.10 (m, 15H), 1.05 (s, 9H). ^{13}C

NMR (101 MHz, CD_3OD) δ 175.05, 174.67, 174.59, 171.79, 170.97, 159.34, 159.21, 158.91, 157.94, 157.69, 156.57, 151.40, 148.82, 147.62, 144.17, 142.79, 137.76, 131.91, 130.08, 129.04, 126.66, 126.22, 124.01, 123.87, 122.15, 118.94, 118.65, 115.29, 113.82, 111.47, 87.45, 80.83, 69.56, 59.23, 57.62, 56.65, 52.47, 49.02, 48.72, 37.81, 37.41, 35.85, 35.27, 35.12, 29.15, 29.09, 28.94, 28.89, 28.86, 25.82, 25.73, 25.60, 22.66, 21.00, 19.21, 16.53, 16.02, 14.47. HRMS (m/z): calculated for $C_{64}H_{80}N_{12}O_6S$ $[M+Na]^+$: 1167.5937, found 1167.5964. Purity: 96.8%.

*4.1.2.25. N1-((S)-1-((2S,4R)-4-hydroxy-2-(((S)-1-(4-(4-methylthiazol-5-yl)phenyl) ethyl)carbamoyl)pyrrolidin-1-yl)-3,3-dimethyl-1-oxobutan-2-yl)-N14-(4-(4-((5-methyl-4-((R)-3-methyl-2-((6-methylpyridin-2-yl)methyl)-1-oxo-1,2,3,4-tetrahydropyrrolo[1,2-a]pyrazin-7-yl)pyrimidin-2-yl)amino)pyridin-2-yl)but-3-yn-1-yl)tetradecanediamide (**B2-14**)*

White solid; m.p. 128-129°C; ^1H NMR (400 MHz, CD_3OD) δ 8.87 (s, 1H), 8.32 (s, 1H), 8.23 (d, J = 6.0 Hz, 1H), 8.16 (s, 1H), 7.75–7.65 (m, 3H), 7.64 (d, J = 1.7 Hz, 1H), 7.47 –7.31 (m, 4H), 7.28 (d, J = 7.8 Hz, 1H), 7.20 (d, J = 7.6 Hz, 1H), 5.30 (d, J = 15.8 Hz, 1H), 5.00 (q, J = 6.9 Hz, 1H), 4.65–4.55 (m, 2H), 4.49–4.36 (m, 3H), 4.26–4.17 (m, 1H), 4.11–4.01 (m, 1H), 3.89 (d, J = 11.0 Hz, 1H), 3.80–3.71 (m, 1H), 3.48–3.39 (m, 2H), 2.70 (t, J = 6.4 Hz, 2H), 2.55 (s, 3H), 2.50–2.41 (m, 6H), 2.33–2.13 (m, 5H), 2.02 –1.90 (m, 1H), 1.63–1.46 (m, 7H), 1.31–1.10 (m, 19H), 1.04 (s, 9H). ^{13}C NMR (101 MHz, CD_3OD) δ 175.11, 174.59, 171.79, 170.94, 159.37, 159.24, 159.00, 157.96, 157.75, 156.56, 151.38, 148.93, 148.72, 147.64, 144.18, 142.75, 137.74, 131.90, 130.09, 129.04, 126.63, 126.19, 124.04, 123.89, 122.12, 118.94, 118.70, 115.25, 113.78, 111.40, 87.52, 80.68, 69.53, 59.19, 57.58, 56.05, 52.48, 49.01, 48.71, 37.75, 37.35, 35.80, 35.24, 35.09, 29.27, 29.23, 29.18, 29.15, 29.08, 28.96, 28.85, 28.82, 25.79, 25.66, 25.57, 22.59, 20.93, 19.15, 16.41, 15.94, 14.37. HRMS (m/z): calculated for $C_{66}H_{84}N_{12}O_6S$ $[M+H]^+$: 1173.6430, found 1173.6461. Purity: 96.3%.

4.1.2.26. N1-((S)-1-((2S,4R)-4-hydroxy-2-(((S)-1-(4-(4-methylthiazol-5-yl)phenyl)

*ethyl)carbamoyl)pyrrolidin-1-yl)-3,3-dimethyl-1-oxobutan-2-yl)-N12-(3-((4-((5-methyl-4-((R)-3-methyl-2-((6-methylpyridin-2-yl)methyl)-1-oxo-1,2,3,4-tetrahydropyrrolo[1,2-a]pyrazin-7-yl)pyrimidin-2-yl)amino)pyridin-2-yl)amino)propyl)dodecanediamide (**B2-12N**)*

White solid; m.p. 133-134°C; ^{1}H NMR (400 MHz, CD_3OD) δ 8.86 (s, 1H), 8.24 (s, 1H), 7.75–7.64 (m, 3H), 7.60 (s, 1H), 7.50 (s, 1H), 7.44–7.36 (m, 4H), 7.26 (d, J = 7.8 Hz, 1H), 7.19 (d, J = 7.7 Hz, 1H), 6.81 (d, J = 6.4 Hz, 1H), 5.30 (d, J = 15.8 Hz, 1H), 5.01 (q, J = 7.0 Hz, 1H), 4.67–4.57 (m, 2H), 4.49–4.35 (m, 3H), 4.20 (d, J = 13.1 Hz, 1H), 4.09–3.98 (m, 1H), 3.90 (d, J = 12.7 Hz, 1H), 3.81–3.72 (m, 1H), 3.37–3.30 (m, 4H), 2.54 (s, 3H), 2.46 (s, 3H), 2.38 (s, 3H), 2.33–2.09 (m, 5H), 2.03–1.93 (m, 1H), 1.90–1.81 (m, 2H), 1.63–1.46 (m, 7H), 1.30–1.17 (m, 15H), 1.05 (s, 9H). ^{13}C NMR (101 MHz, CD_3OD) δ 174.92, 174.56, 171.79, 170.94, 159.27, 158.89, 158.10, 157.97, 157.85, 156.52, 151.39, 150.09, 147.63, 144.18, 143.18, 137.76, 131.90, 130.08, 129.04, 126.66, 126.22, 123.85, 122.15, 118.89, 118.51, 113.86, 109.99, 103.94, 93.92, 69.56, 59.23, 57.60, 56.63, 52.42, 48.94, 48.72, 39.22, 37.43, 36.57, 35.85, 35.28, 35.13, 29.16, 29.01, 28.91, 28.52, 25.74, 25.69, 25.62, 22.71, 21.03, 16.55, 16.01, 14.50. HRMS (m/z): calculated for $C_{63}H_{83}N_{13}O_6S$ $[M+H]^+$: 1150.6383, found 1150.6415. Purity: 99.1%.

*4.1.2.27. (R,E)-7-(2,6-dimethoxy-4-(3-oxo-3-(6-oxo-3,6-dihydropyridin-1(2H)-yl)prop-1-en-1-yl)phenoxy)-N-(4-(4-((5-methyl-4-(3-methyl-2-((6-methylpyridin-2-yl)methyl)-1-oxo-1,2,3,4-tetrahydropyrrolo[1,2-a]pyrazin-7-yl)pyrimidin-2-yl)amino)pyridin-2-yl)but-3-yn-1-yl)heptanamide (**B3**)*

White solid; m.p. 105-106°C; ^{1}H NMR (400 MHz, $CDCl_3$) δ 8.27 (d, J = 5.7 Hz, 1H), 8.19 (s, 2H), 7.86 (s, 1H), 7.60 (d, J = 15.5 Hz, 1H), 7.56–7.45 (m, 3H), 7.42–7.31 (m, 2H), 7.14 (d, J = 7.7 Hz, 1H), 7.02 (d, J = 7.6 Hz, 1H), 6.95–6.86 (m, 1H), 6.75 (t, J = 6.0 Hz, 1H), 6.71 (s, 2H), 6.00 (d, J = 9.8 Hz, 1H), 5.39 (d, J = 15.4 Hz, 1H), 4.33–4.25 (m, 1H), 4.21 (d, J = 15.5 Hz, 1H), 4.02 – 3.86 (m, 6H), 3.78 (s, 6H), 3.51–3.40 (m, 2H), 2.64 (t, J = 6.3 Hz, 2H), 2.49 (s, 3H), 2.43 (q, J = 4.5 Hz, 2H), 2.35 (s, 3H), 2.14

(t, J = 7.6 Hz, 2H), 1.70–1.55 (m, 4H), 1.41–1.25 (m, 4H), 1.23 (d, J = 6.6 Hz, 3H). ^{13}C NMR (101 MHz, $CDCl_3$) δ 173.47, 168.86, 165.84, 159.58, 159.07, 158.27, 157.96, 157.60, 156.75, 153.48, 149.92, 147.52, 145.64, 143.75, 143.46, 139.29, 137.15, 130.29, 125.69, 125.56, 124.79, 123.89, 122.04, 120.89, 119.01, 118.69, 115.50, 113.93, 111.43, 105.53, 87.15, 81.88, 77.38, 73.35, 56.10, 51.68, 49.65, 49.42, 41.64, 38.12, 36.49, 29.88, 28.99, 25.73, 25.50, 24.77, 24.43, 20.29, 17.62, 17.24. HRMS (m/z): calculated for $C_{52}H_{57}N_9O_7$ $[M+Na]^+$: 942.4273, found 942.4268. Purity: 98.1%.

*4.1.2.28. (R)-N1-(adamantan-1-yl)-N10-(4-(4-((5-methyl-4-(3-methyl-2-((6-methylpyridin-2-yl)methyl)-1-oxo-1,2,3,4-tetrahydropyrrolo[1,2-a]pyrazin-7-yl)pyrimidin-2-yl)amino)pyridin-2-yl)but-3-yn-1-yl)decanediamide (**B4**)*

White solid; m.p. 130-131°C; ^{1}H NMR (400 MHz, CD_3OD) δ 8.23 (s, 1H), 8.20 (d, J = 5.9 Hz, 1H), 8.05 (d, J = 1.9 Hz, 1H), 7.74–7.65 (m, 2H), 7.63 (d, J = 1.6 Hz, 1H), 7.57 (d, J = 1.4 Hz, 1H), 7.26 (d, J = 7.7 Hz, 1H), 7.19 (d, J = 7.6 Hz, 1H), 5.28 (d, J = 15.7 Hz, 1H), 4.45–4.35 (m, 2H), 4.23–4.14 (m, 1H), 4.08–4.00 (m, 1H), 3.44 (t, J = 6.6 Hz, 2H), 2.69 (t, J = 6.5 Hz, 2H), 2.54 (s, 3H), 2.38 (s, 3H), 2.17 (t, J = 7.4 Hz, 2H), 2.07–1.95 (m, 11H), 1.67 (s, 6H), 1.62–1.53 (m, 2H), 1.51–1.42 (m, 2H), 1.30–1.16 (m, 11H). ^{13}C NMR (101 MHz, CD_3OD) δ 175.01, 174.04, 159.31, 159.18, 158.82, 157.94, 157.64, 156.57, 148.89, 148.65, 142.57, 137.74, 126.60, 123.96, 123.86, 122.13, 118.91, 118.64, 115.25, 113.78, 111.34, 87.57, 80.68, 52.42, 51.22, 48.95, 40.95, 37.85, 36.56, 36.12, 35.78, 29.48, 28.95, 28.88, 28.85, 28.76, 25.74, 25.72, 22.68, 19.29, 16.54, 16.00. HRMS (m/z): calculated for $C_{49}H_{61}N_9O_3$ $[M+Na]^+$: 846.4790, found 846.4786. Purity: 99.4%.

4.1.3. General Procedure for B1-10J, B1-10P, B2-12J and B2-12P.

A mixture of ligands of ERK1/2 (1 eq), complex of E3 ligand and linker (1 eq), K_2CO_3 (2 eq), DMF was stirred at 80°C for 8 hours. Then the mixture was dissolved in EA and washed with brine, dried over Na_2SO_4, filtered and concentrated. The crude product was purified by column chromatography (DCM:MeOH = 10:1, 0.3% ammonia). The

corresponding Amide compound (15–48%) was obtained.

*4.1.3.1. 2-(2,6-dioxopiperidin-3-yl)-4-((10-(methyl(4-(4-((5-methyl-4-((R)-3-methyl-2-((6-methylpyridin-2-yl)methyl)-1-oxo-1,2,3,4-tetrahydropyrrolo[1,2-a]pyrazin-7-yl)pyrimidin-2-yl)amino)pyridin-2-yl)but-3-yn-1-yl)amino)decyl)amino)isoindoline-1,3-dione (**B1-10J**)*

Yellow solid; m.p. 128-129°C; ^{1}H NMR (400 MHz, DMSO-*d*6) δ 11.12 (s, 1H), 9.93 (s, 1H), 8.36 (s, 1H), 8.26 (d, J = 5.7 Hz, 1H), 8.07 (s, 1H), 7.74 (s, 1H), 7.69 – 7.60 (m, 2H), 7.54 (t, J = 7.6 Hz, 1H), 7.41 (s, 1H), 7.20–7.10 (m, 2H), 7.01 (t, J = 7.7 Hz, 2H), 6.46 (t, J = 5.9 Hz, 1H), 5.18 (d, J = 16.0 Hz, 1H), 5.06 (t, J = 12.9, 5.4 Hz, 1H), 4.44–4.34 (m, 1H), 4.30 (d, J = 16.0 Hz, 1H), 4.22 (d, J = 13.1 Hz, 1H), 4.04–3.92 (m, 1H), 3.22 (q, J = 6.6 Hz, 2H), 2.96–2.82 (m, 1H), 2.68–2.54 (m, 5H), 2.46 (s, 3H), 2.36 (s, 5H), 2.20 (s, 3H), 2.09–1.98 (m, 1H), 1.55–1.46 (m, 2H), 1.40–1.32 (m, 2H), 1.30–1.12 (m, 16H). ^{13}C NMR (101 MHz, DMSO-*d*6) δ 173.24, 170.52, 169.41, 167.75, 160.02, 158.95, 158.21, 157.96, 157.85, 157.39, 150.32, 148.19, 146.84, 143.64, 137.60, 136.67, 132.59, 126.80, 124.92, 123.21, 122.02, 118.72, 118.44, 117.51, 115.40, 112.95, 111.71, 110.80, 109.45, 88.37, 82.17, 56.81, 55.79, 52.04, 49.38, 49.16, 49.02, 42.30, 41.84, 31.46, 29.49, 29.45, 29.23, 29.14, 27.26, 27.04, 26.78, 24.47, 22.65, 17.64, 17.45, 17.20. HRMS (m/z): calculated for $C_{53}H_{61}N_{11}O_5$ $[M+H]^+$: 932.4930, found 932.4938. Purity: 99.6%.

*4.1.3.2. 2-(2,6-dioxopiperidin-3-yl)-4-((8-(4-(4-(4-((5-methyl-4-((R)-3-methyl-2-((6-methylpyridin-2-yl)methyl)-1-oxo-1,2,3,4-tetrahydropyrrolo[1,2-a]pyrazin-7-yl)pyrimidin-2-yl)amino)pyridin-2-yl)but-3-yn-1-yl)piperazin-1-yl)octyl)amino) isoindoline-1,3-dione (**B1-10P**)*

Yellow solid; m.p. 134-135°C; ^{1}H NMR (400 MHz, DMSO-*d*6) δ 11.11 (s, 1H), 9.94 (s, 1H), 8.37 (s, 1H), 8.26 (d, J = 5.7 Hz, 1H), 8.06 (s, 1H), 7.75 (s, 1H), 7.69–7.61 (m, 2H), 7.57 (t, J = 7.9 Hz, 1H), 7.41 (s, 1H), 7.21–7.11 (m, 2H), 7.06 (d, J = 8.6 Hz, 1H), 7.01 (d, J = 7.0 Hz, 1H), 6.50 (t, J = 5.9 Hz, 1H), 5.17 (d, J = 16.0 Hz, 1H), 5.10–5.01

(m, 1H), 4.45–4.35 (m, 1H), 4.35–4.19 (m, 2H), 4.04–3.93 (m, 1H), 3.27 (q, J = 6.7 Hz, 4H), 2.96–2.82 (m, 1H), 2.67–2.53 (m, 6H), 2.49–2.35 (m, 11H), 2.28 (t, J = 6.7 Hz, 2H), 2.09–1.97 (m, 1H), 1.56 (t, J = 7.0 Hz, 2H), 1.44–1.15 (m, 14H). ^{13}C NMR (101 MHz, DMSO-*d6*) δ 173.26, 170.53, 169.42, 167.76, 160.07, 158.98, 158.21, 157.97, 157.86, 157.41, 150.37, 148.21, 146.88, 143.60, 137.64, 136.72, 132.62, 126.84, 124.93, 123.18, 122.03, 118.68, 118.49, 117.60, 115.42, 112.92, 111.74, 110.84, 109.46, 88.30, 82.13, 58.03, 56.61, 52.92, 52.35, 52.06, 49.39, 49.17, 49.02, 42.31, 31.46, 29.31, 29.16, 29.12, 27.23, 26.74, 26.33, 24.49, 22.65, 17.64, 17.49, 17.22. HRMS (m/z): calculated for $C_{54}H_{62}N_{12}O_5$ [M+H]$^+$: 959.5039, found 959.5038. Purity: 98.9%.

4.1.3.3. (2S,4R)-1-((S)-3,3-dimethyl-2-(12-(methyl(4-(4-((5-methyl-4-((R)-3-methyl-2-((6-methylpyridin-2-yl)methyl)-1-oxo-1,2,3,4-tetrahydropyrrolo[1,2-a]pyrazin-7-yl)pyrimidin-2-yl)amino)pyridin-2-yl)but-3-yn-1-yl)amino)dodecanamido)butanoyl)-4-hydroxy-N-((S)-1-(4-(4-methylthiazol-5-yl)phenyl)ethyl)pyrrolidine-2-carboxamide ***(B2-12J)***

White solid; m.p. 123-124°C; ^{1}H NMR (400 MHz, CD_3OD) δ 8.86 (s, 1H), 8.27 (s, 1H), 8.22 (d, J = 5.9 Hz, 1H), 8.08 (d, J = 2.2 Hz, 1H), 7.75–7.67 (m, 2H), 7.66 (d, J = 1.7 Hz, 1H), 7.60 (d, J = 1.7 Hz, 1H), 7.41 (s, 4H), 7.27 (d, J = 7.7 Hz, 1H), 7.20 (d, J = 7.6 Hz, 1H), 5.31 (d, J = 15.8 Hz, 1H), 5.01 (q, J = 7.0 Hz, 1H), 4.67–4.56 (m, 2H), 4.49–4.34 (m, 3H), 4.19 (d, J = 13.0 Hz, 1H), 4.09–3.97 (m, 1H), 3.94–3.86 (m, 1H), 3.81–3.72 (m, 1H), 2.88 (t, J = 7.3 Hz, 2H), 2.73 (t, J = 7.2 Hz, 2H), 2.61–2.52 (m, 5H), 2.47 (s, 3H), 2.43–2.36 (m, 6H), 2.32–2.14 (m, 3H), 2.04–1.92 (m, 1H), 1.62–1.45 (m, 7H), 1.31–1.20 (m, 17H), 1.05 (s, 9H). ^{13}C NMR (101 MHz, CD_3OD) δ 174.58, 171.78, 170.93, 159.29, 159.16, 158.95, 157.95, 157.70, 156.58, 151.39, 148.88, 148.82, 147.63, 144.18, 142.75, 137.78, 131.91, 130.08, 129.04, 126.61, 126.22, 124.01, 123.84, 122.15, 118.90, 118.66, 115.27, 113.78, 111.41, 87.59, 80.93, 69.56, 59.21, 57.59, 56.70, 56.62, 55.05, 52.32, 48.87, 48.71, 40.75, 37.39, 35.29, 35.12, 29.30, 29.24, 29.18, 29.01, 28.90, 27.08, 26.18, 25.73, 25.61, 22.68, 21.01, 16.52, 16.18, 15.99, 14.48. HRMS (m/z): calculated for $C_{65}H_{84}N_{12}O_5S$ [M+H]$^+$: 1145.6481, found 1145.6522.

Purity: 98.0%.

*4.1.3.4. (2S,4R)-1-((S)-3,3-dimethyl-2-(10-(4-(4-(4-((5-methyl-4-((R)-3-methyl-2-((6-methylpyridin-2-yl)methyl)-1-oxo-1,2,3,4-tetrahydropyrrolo[1,2-a]pyrazin-7-yl)pyrimidin-2-yl)amino)pyridin-2-yl)but-3-yn-1-yl)piperazin-1-yl)decanamido) butanoyl)-4-hydroxy-N-((S)-1-(4-(4-methylthiazol-5-yl)phenyl)ethyl)pyrrolidine-2-carboxamide (**B2-12P**)*

White solid; m.p. 145-146°C; ^{1}H NMR (400 MHz, CD_3OD) δ 8.87 (s, 1H), 8.27 (s, 1H), 8.22 (d, J = 5.9 Hz, 1H), 8.01 (s, 1H), 7.78–7.64 (m, 3H), 7.59 (s, 1H), 7.47–7.34 (m, 4H), 7.27 (d, J = 7.7 Hz, 1H), 7.20 (d, J = 7.7 Hz, 1H), 5.31 (d, J = 15.8 Hz, 1H), 5.01 (q, J = 7.0 Hz, 1H), 4.65 (s, 1H), 4.60 (t, J = 8.3 Hz, 1H), 4.48–4.35 (m, 3H), 4.19 (d, J = 13.1 Hz, 1H), 4.08 – 3.98 (m, 1H), 3.90 (d, J = 11.1 Hz, 1H), 3.80–3.73 (m, 1H), 2.81 –2.59 (m, 11H), 2.57–2.45 (m, 9H), 2.41 (s, 3H), 2.33–2.18 (m, 3H), 2.04–1.93 (m, 1H), 1.66–1.48 (m, 7H), 1.34–1.26 (m, 13H), 1.06 (s, 9H). ^{13}C NMR (101 MHz, CD_3OD) δ 174.58, 171.79, 170.90, 159.28, 159.19, 158.97, 157.95, 157.71, 156.58, 151.40, 148.87, 148.82, 147.63, 144.20, 142.81, 137.80, 131.91, 130.09, 129.05, 126.62, 126.21, 123.99, 123.82, 122.15, 118.86, 118.69, 115.25, 113.74, 111.30, 87.87, 80.66, 69.55, 59.19, 57.88, 57.57, 56.59, 56.03, 52.34, 52.24, 51.42, 48.87, 48.71, 37.39, 35.26, 35.11, 29.04, 28.95, 28.88, 26.97, 25.69, 25.59, 25.47, 22.67, 21.00, 16.67, 16.48, 15.96, 14.46. HRMS (m/z): calculated for $C_{66}H_{85}N_{13}O_5S$ $[M+H]^+$: 1172.6590, found 1172.6618. Purity: 95.3%.

5. ^{1}H NMR and ^{13}C NMR spectrum

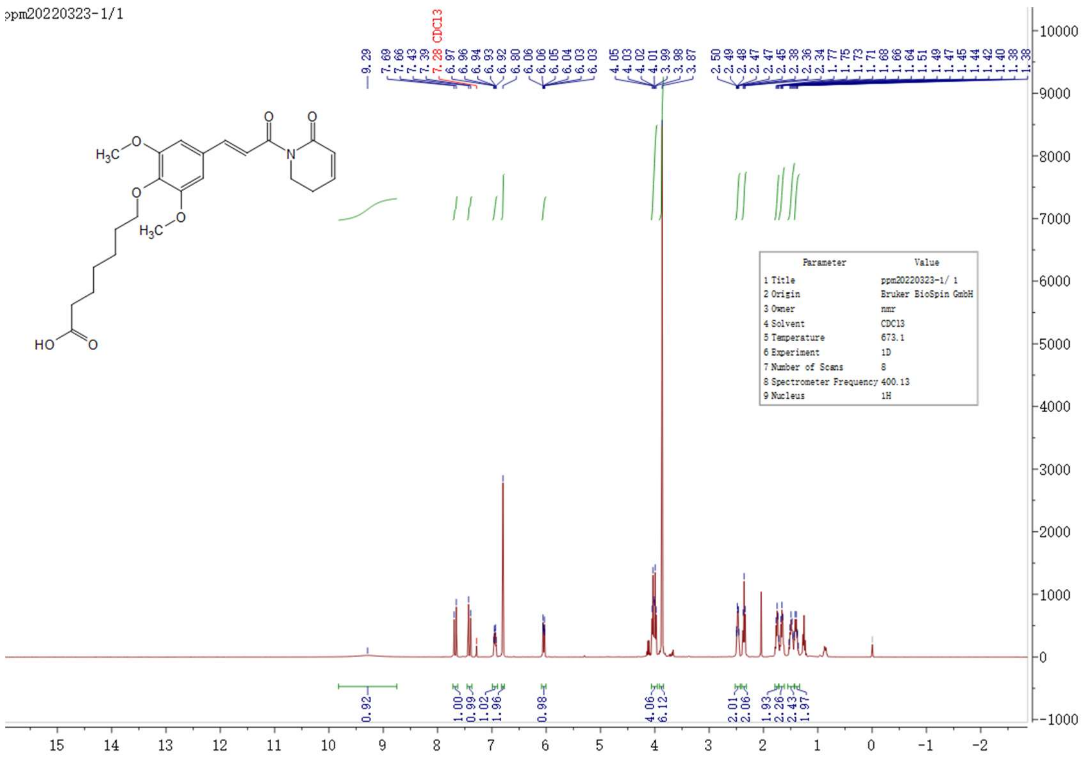

^{1}H NMR of (E)-7-(2,6-dimethoxy-4-(3-oxo-3-(6-oxo-3,6-dihydropyridin-1(2H)-yl)prop-1-en-1-yl)phenoxy)heptanoic acid (**7**)

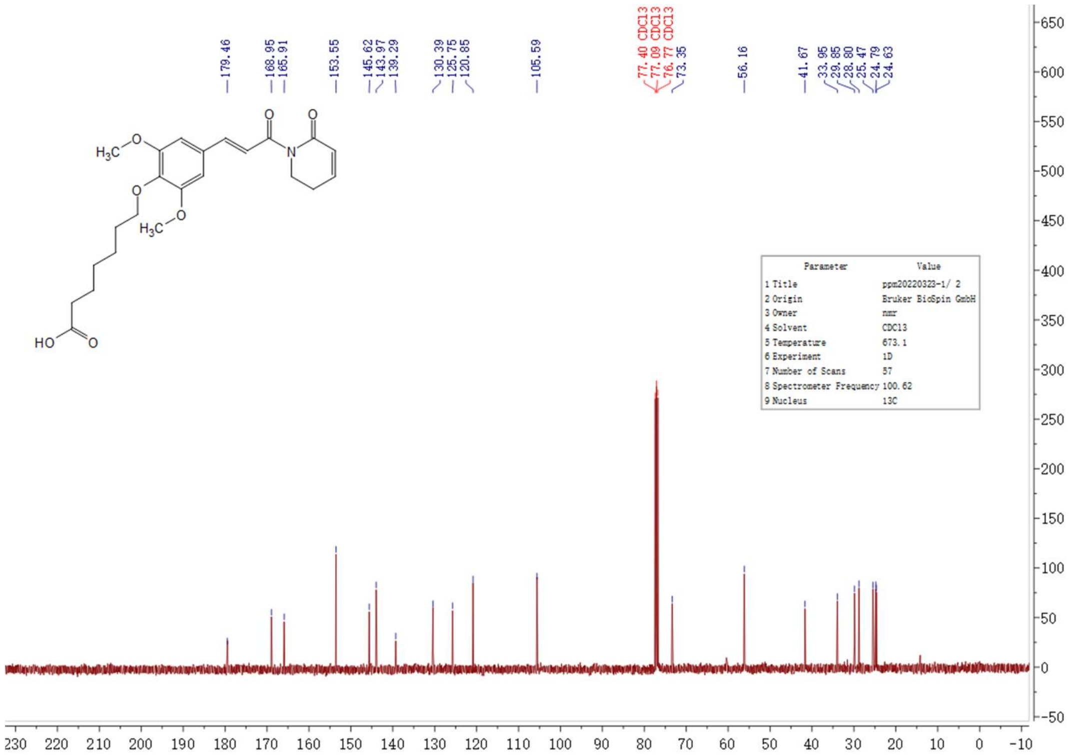

^{13}C NMR of (E)-7-(2,6-dimethoxy-4-(3-oxo-3-(6-oxo-3,6-dihydropyridin-1(2H)-yl)prop-1-en-1-yl)phenoxy)heptanoic acid (**7**)

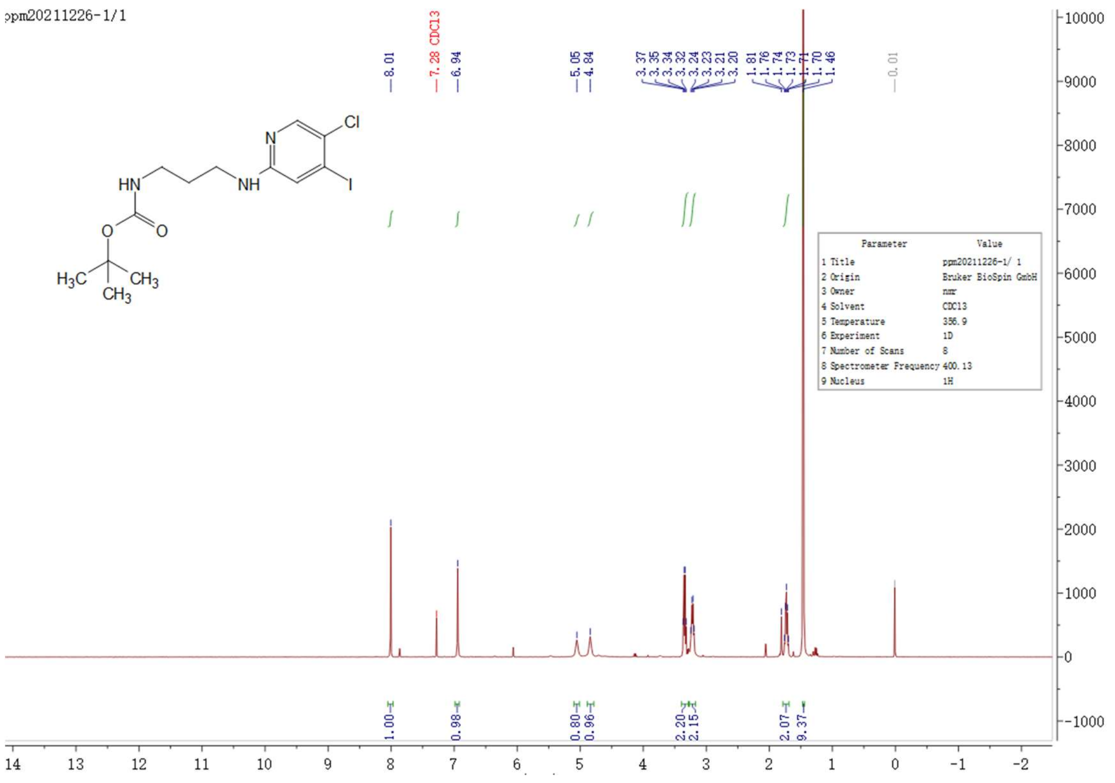

^{1}H NMR of tert-butyl (3-((5-chloro-4-iodopyridin-2-yl)amino)propyl)carbamate (**9**)

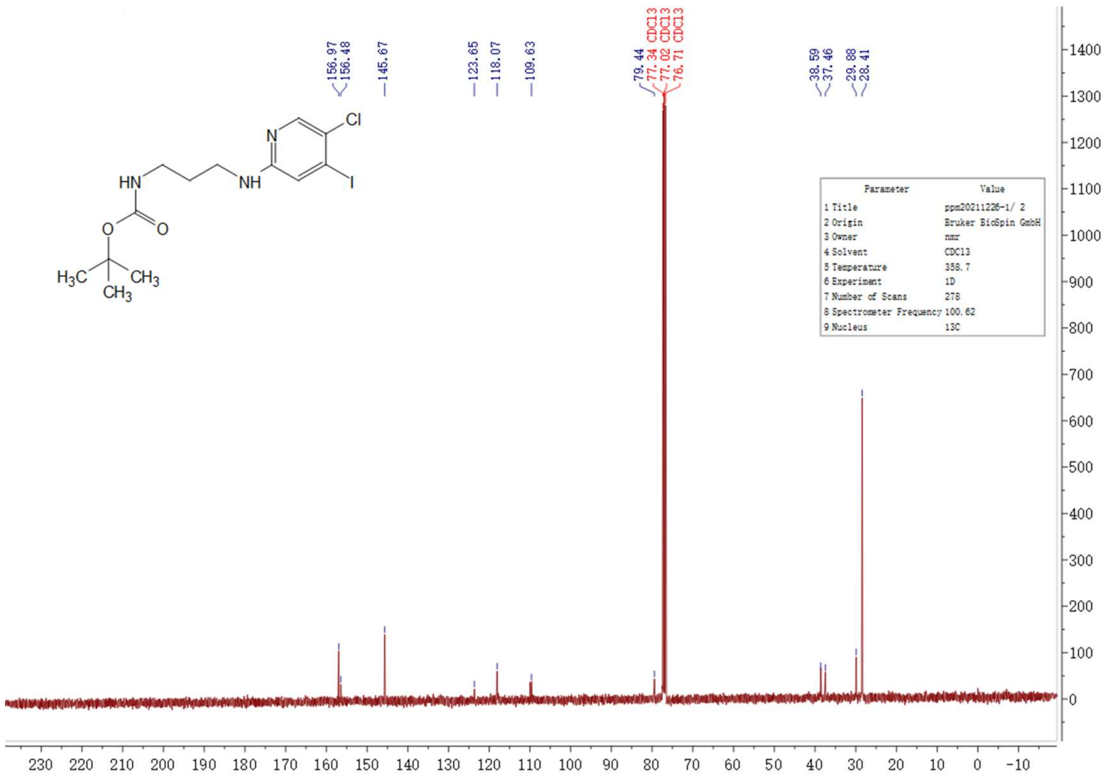

^{13}C NMR of of tert-butyl (3-((5-chloro-4-iodopyridin-2-yl)amino)propyl)carbamate (**9**)

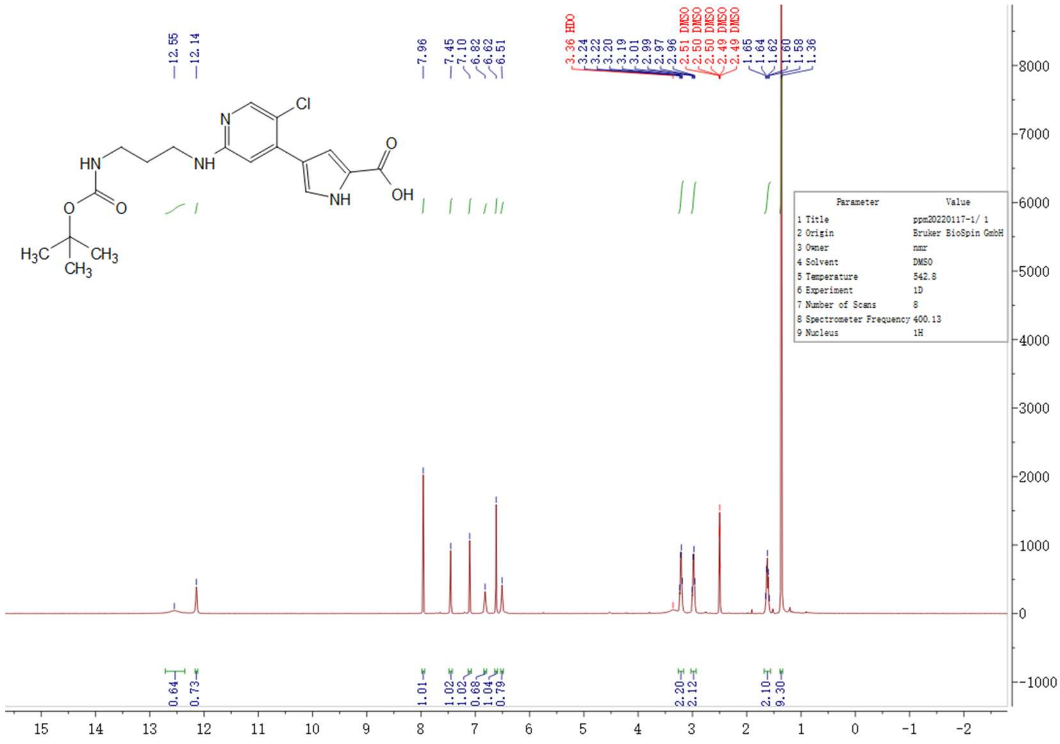

^{1}H NMR of 4-(2-((3-((tert-butoxycarbonyl)amino)propyl)amino)-5-chloropyridin-4-yl)-1H-pyrrole-2-carboxylic acid (**14**)

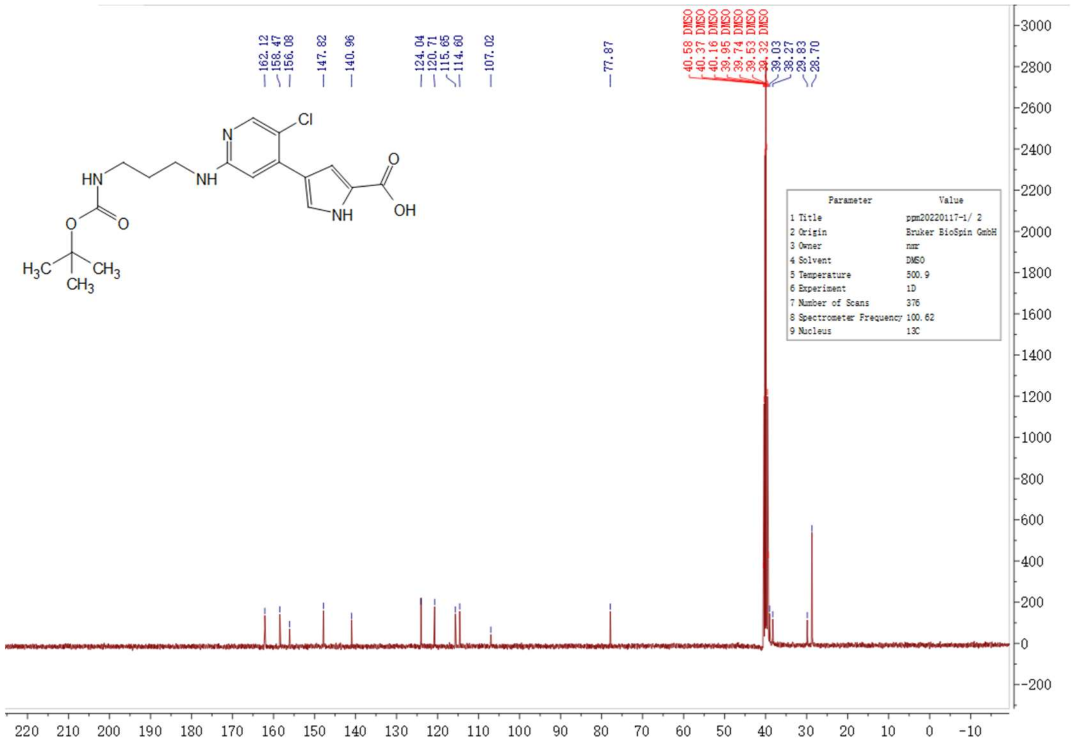

^{13}C NMR of 4-(2-((3-((tert-butoxycarbonyl)amino)propyl)amino)-5-chloropyridin-4-yl)-1H-pyrrole-2-carboxylic acid (**14**)

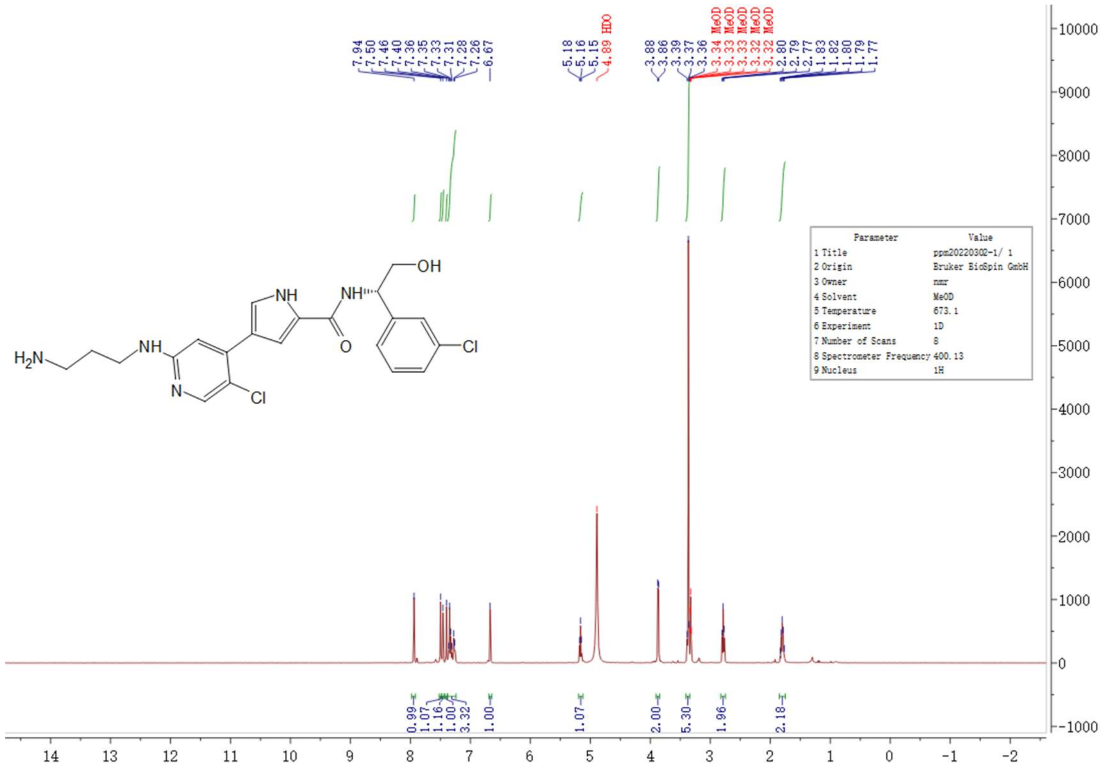

^{1}H NMR of **A**

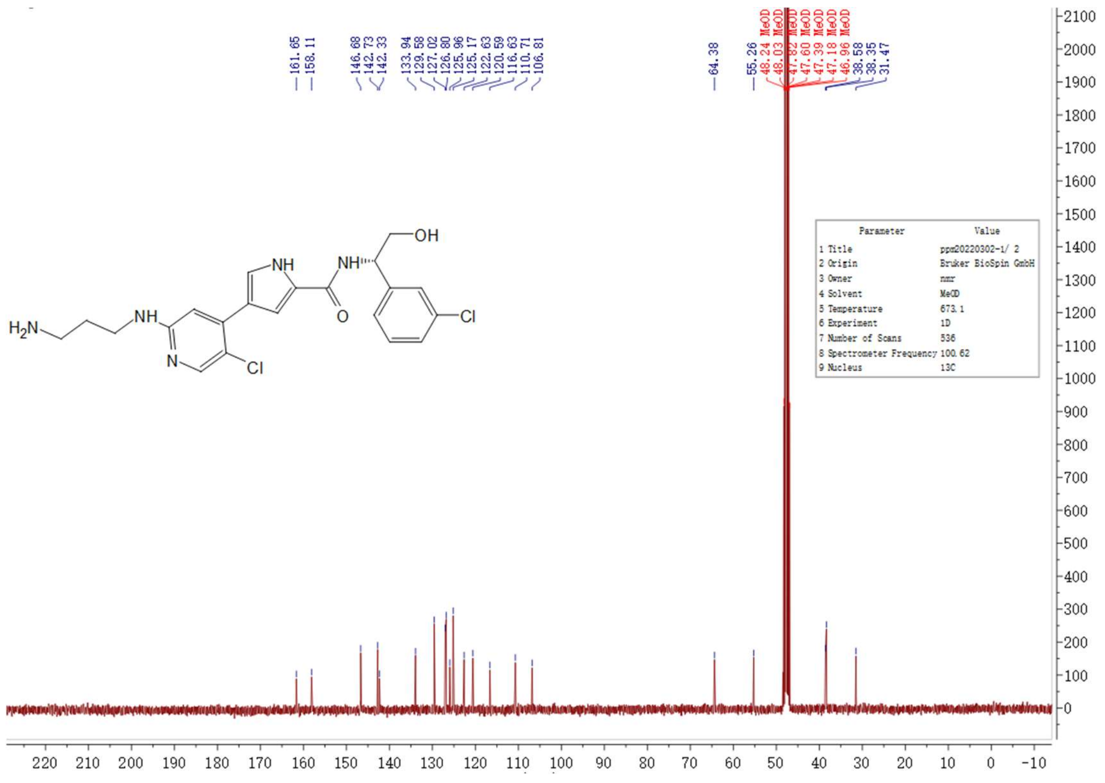

^{13}C NMR of **A**

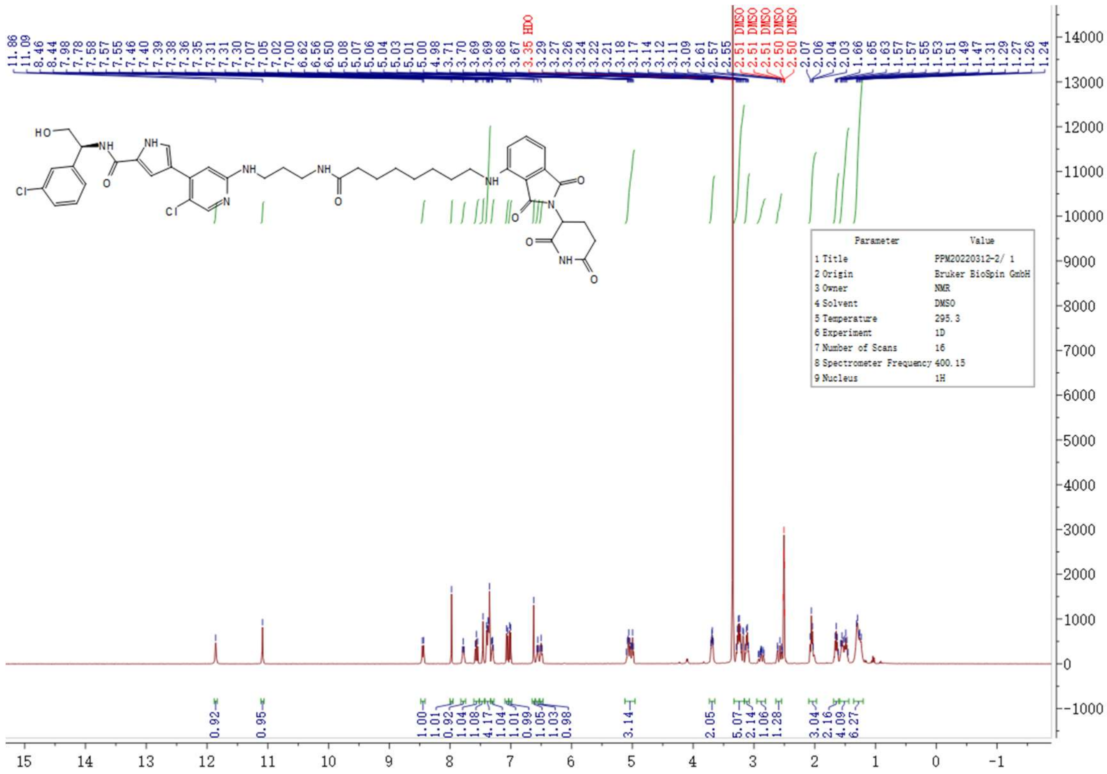

^{1}H NMR of **A1**

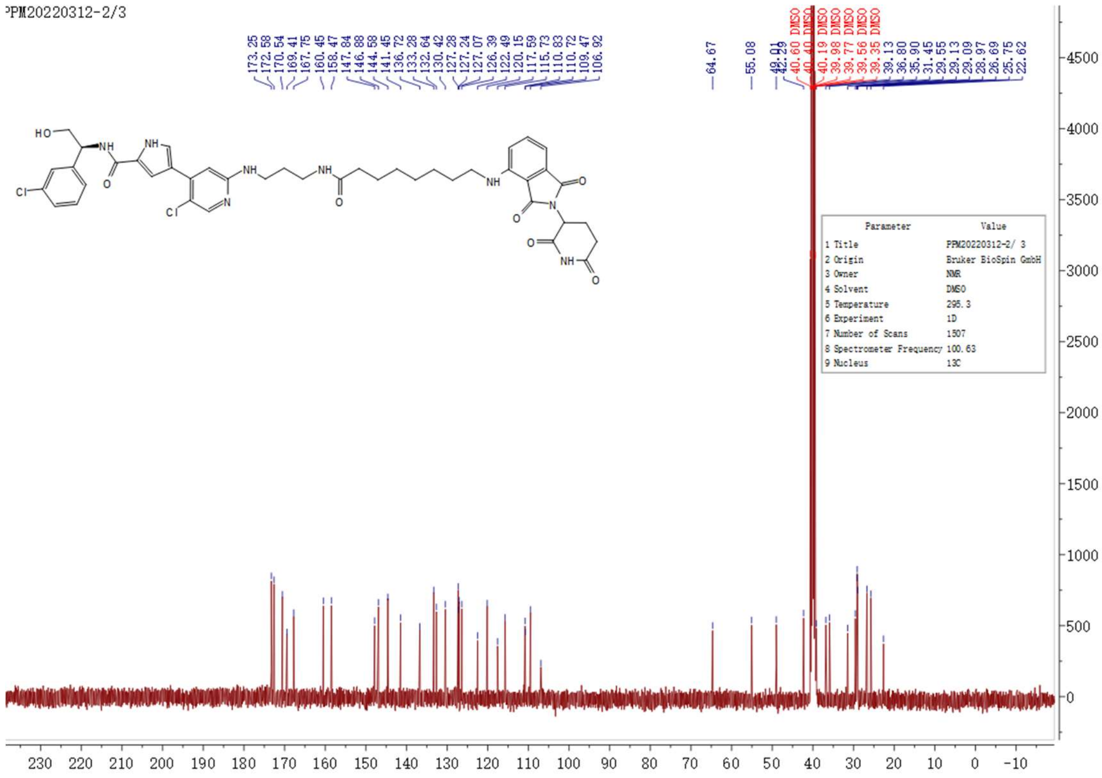

^{13}C NMR of **A1**

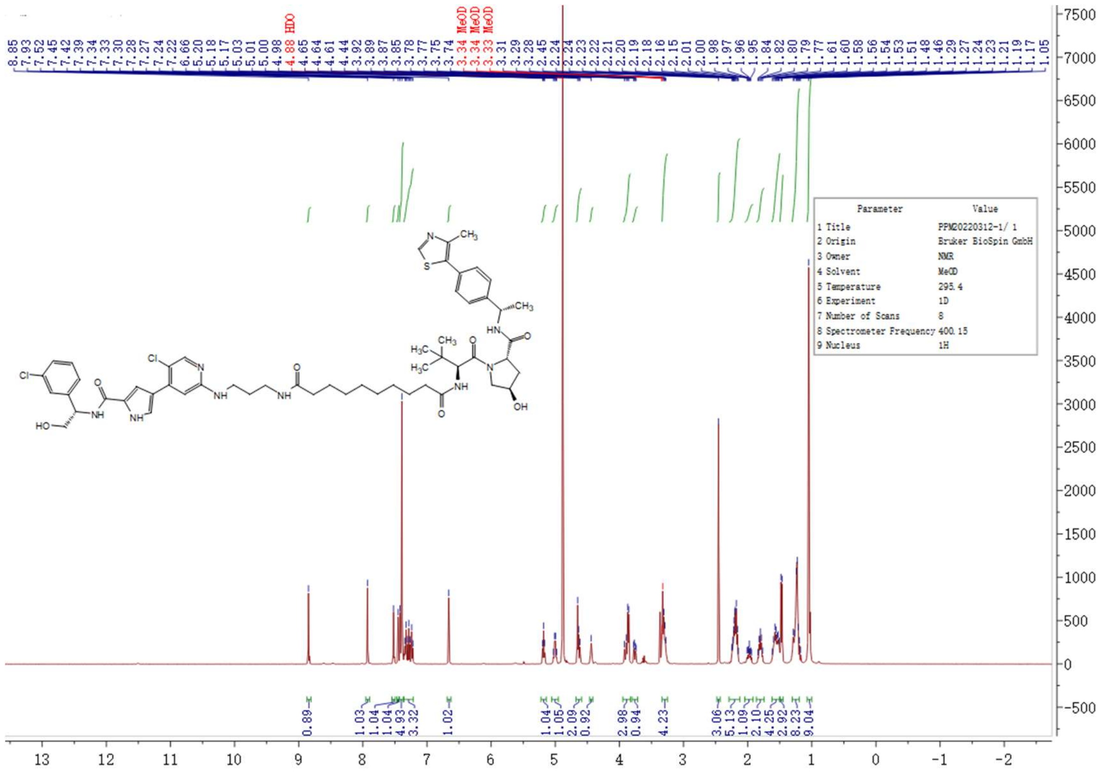

^{1}H NMR of **A2**

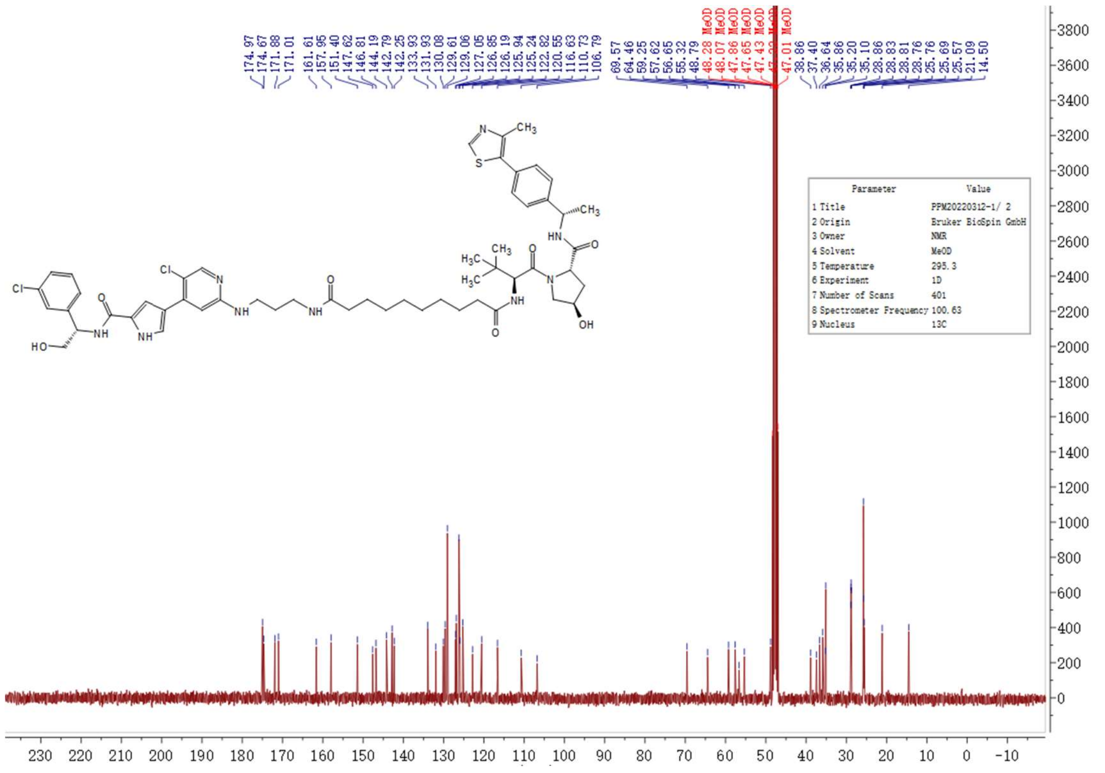

^{13}C NMR of **A2**

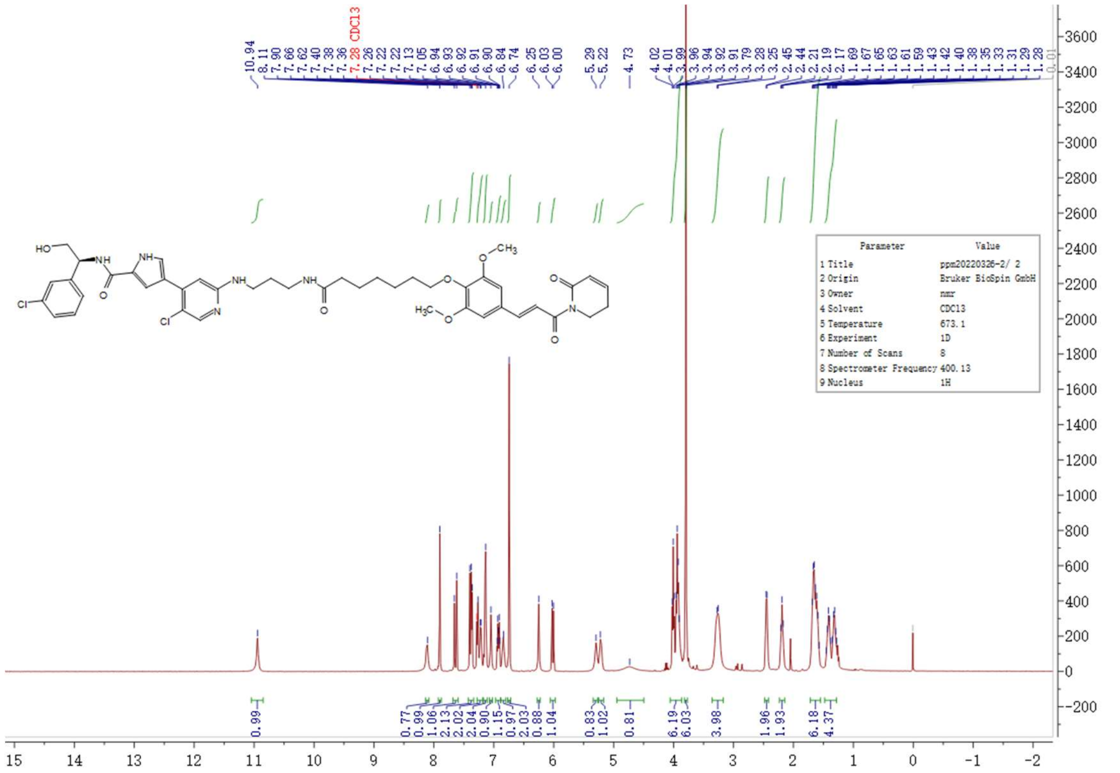

^{1}H NMR of **A3**

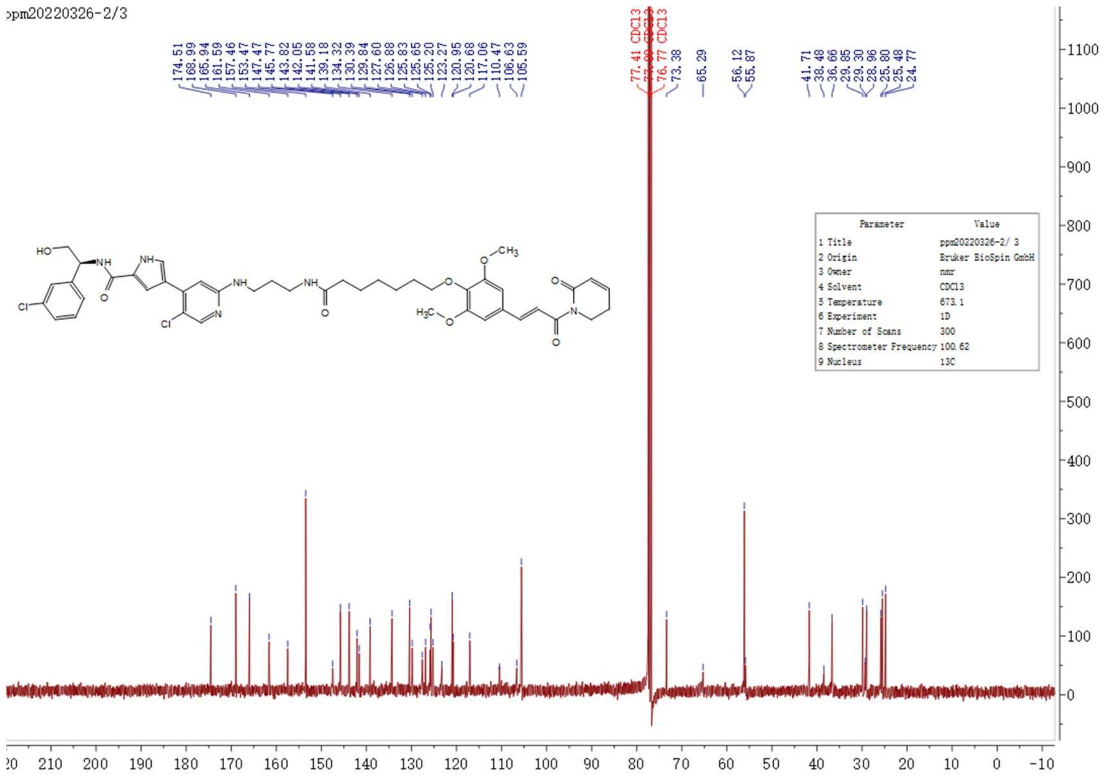

^{13}C NMR of **A3**

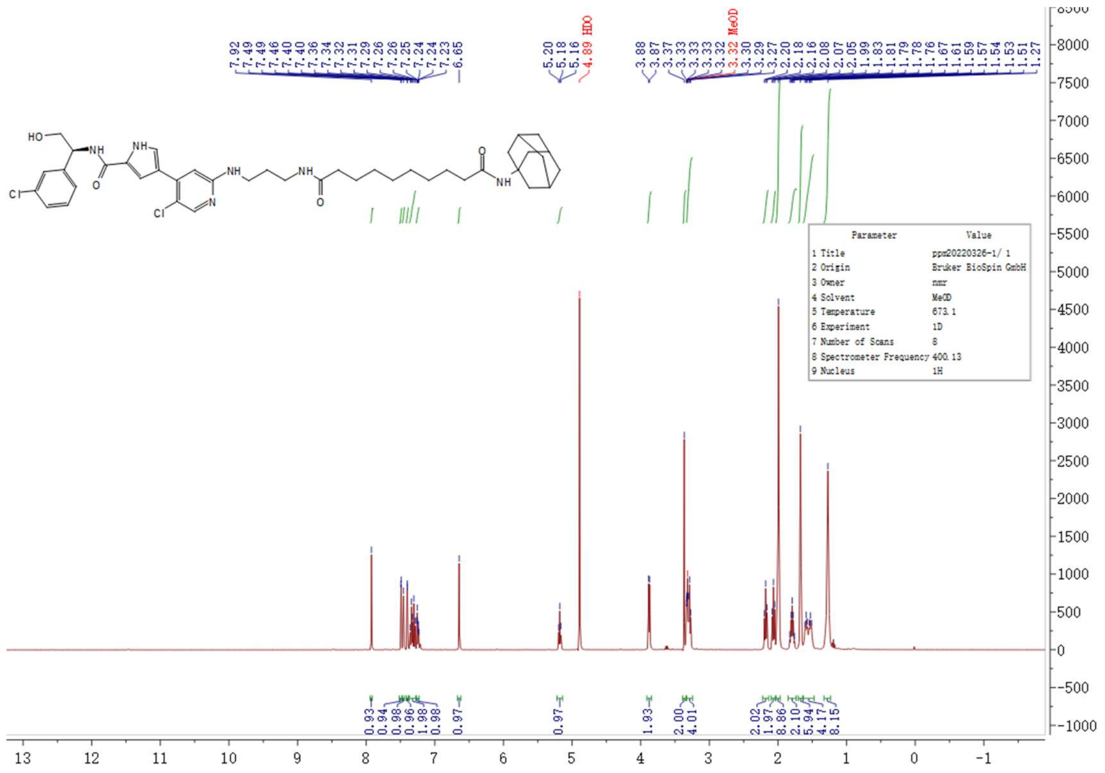

^{1}H NMR of **A4**

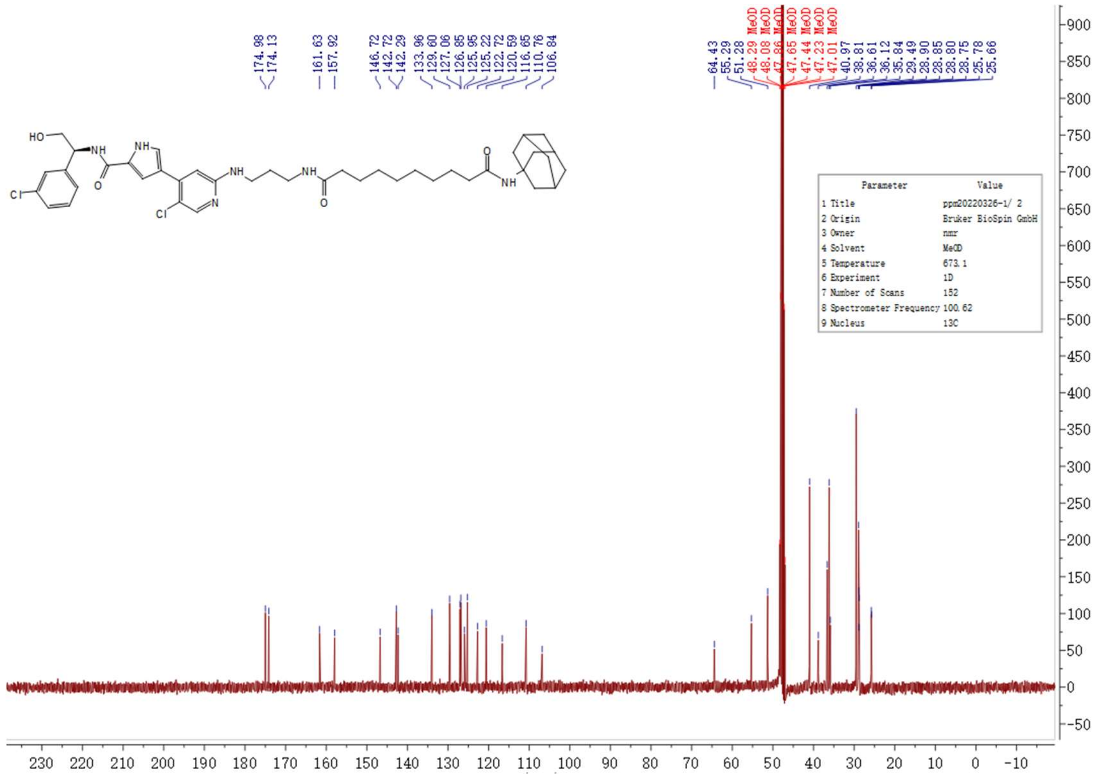

^{13}C NMR of **A4**

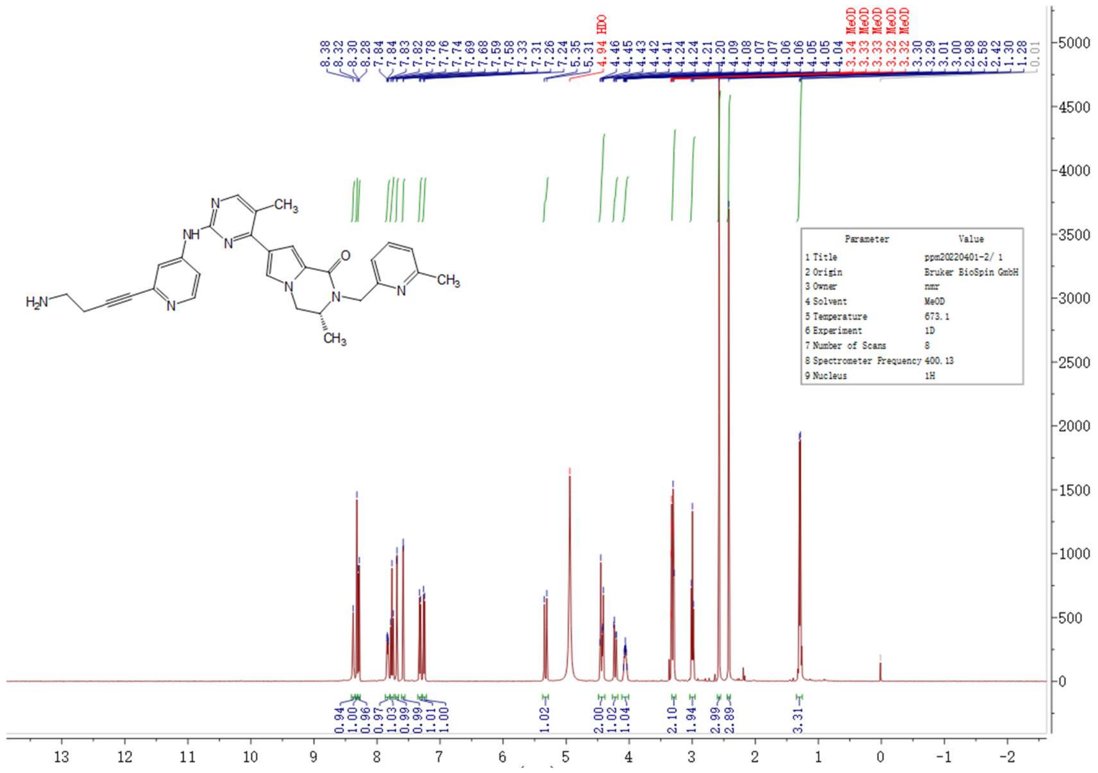

^{1}H NMR of **B**

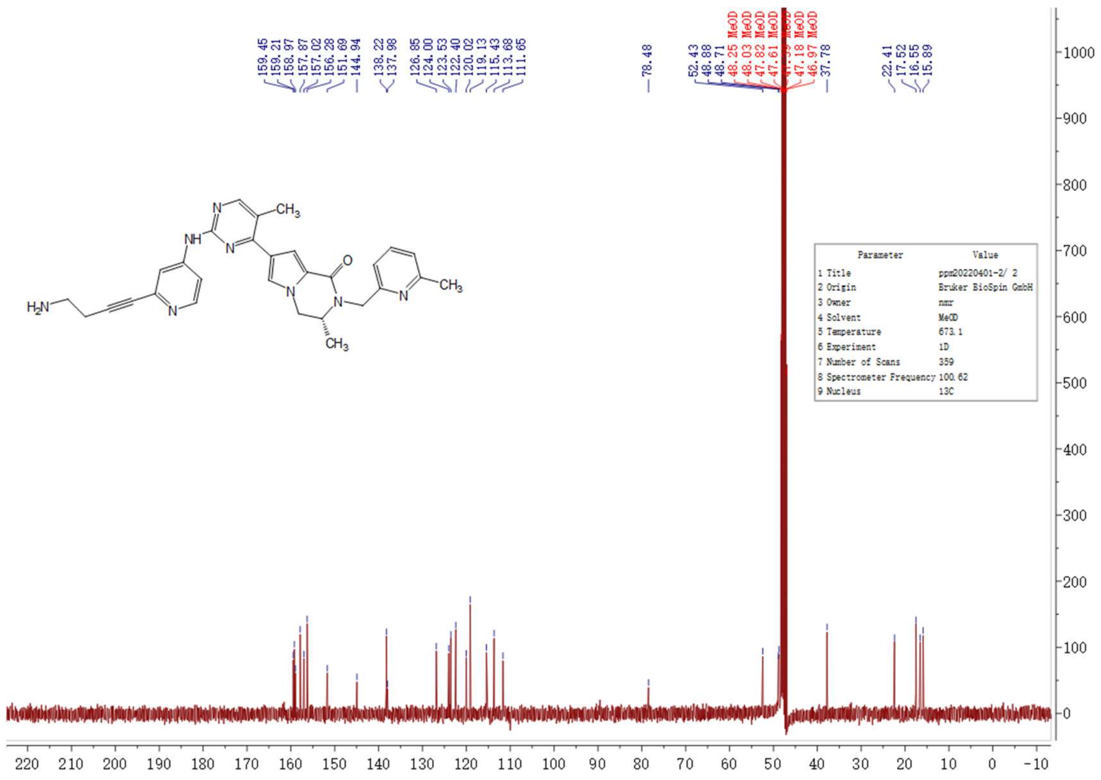

^{13}C NMR of **B**

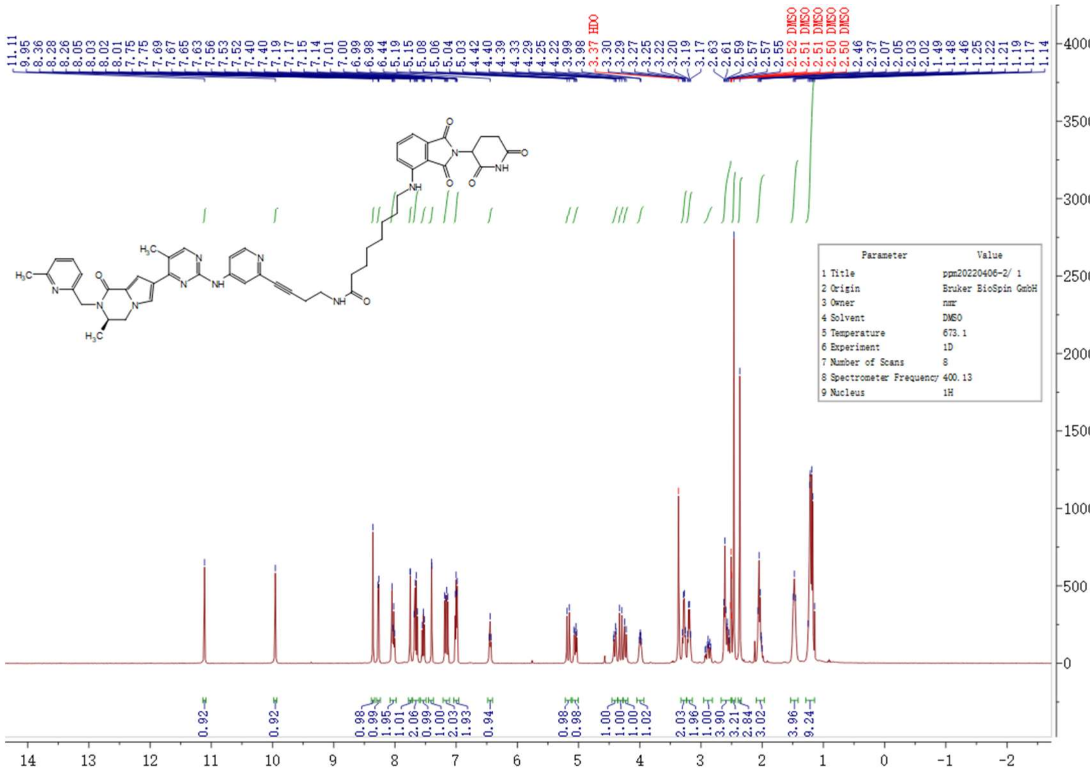

^{1}H NMR of **B1-8**

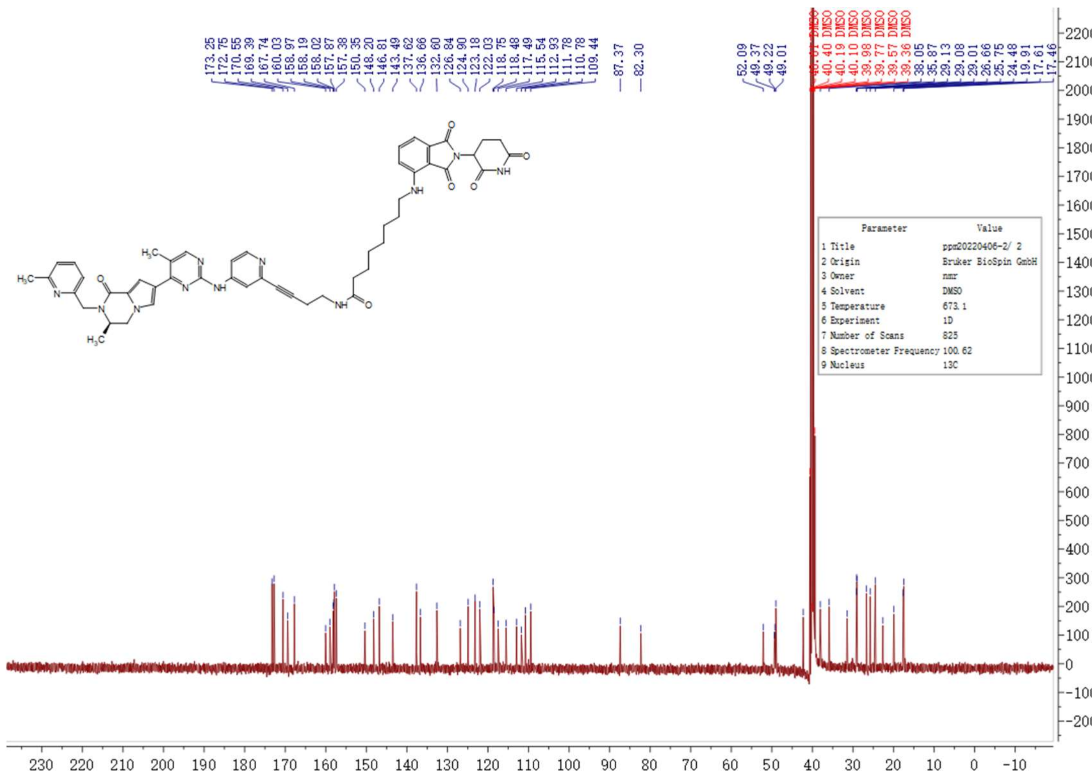

^{13}C NMR of **B1-8**

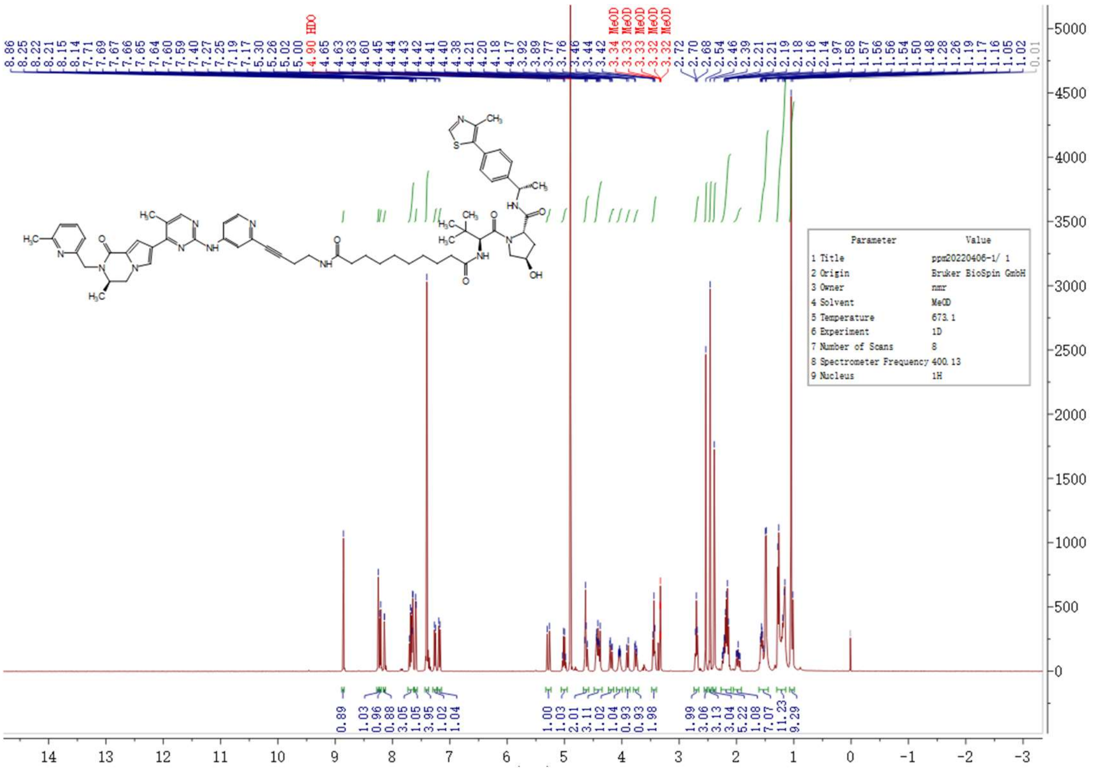

^{1}H NMR of **B2-10**

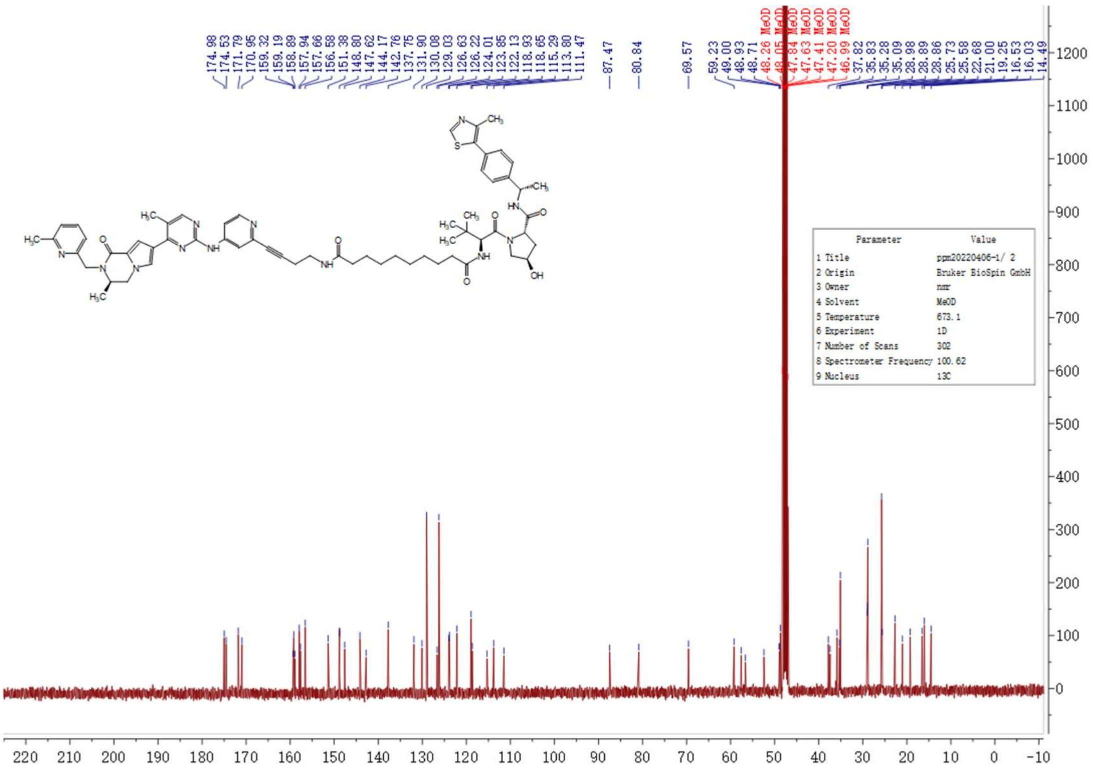

^{13}C NMR of **B2-10**

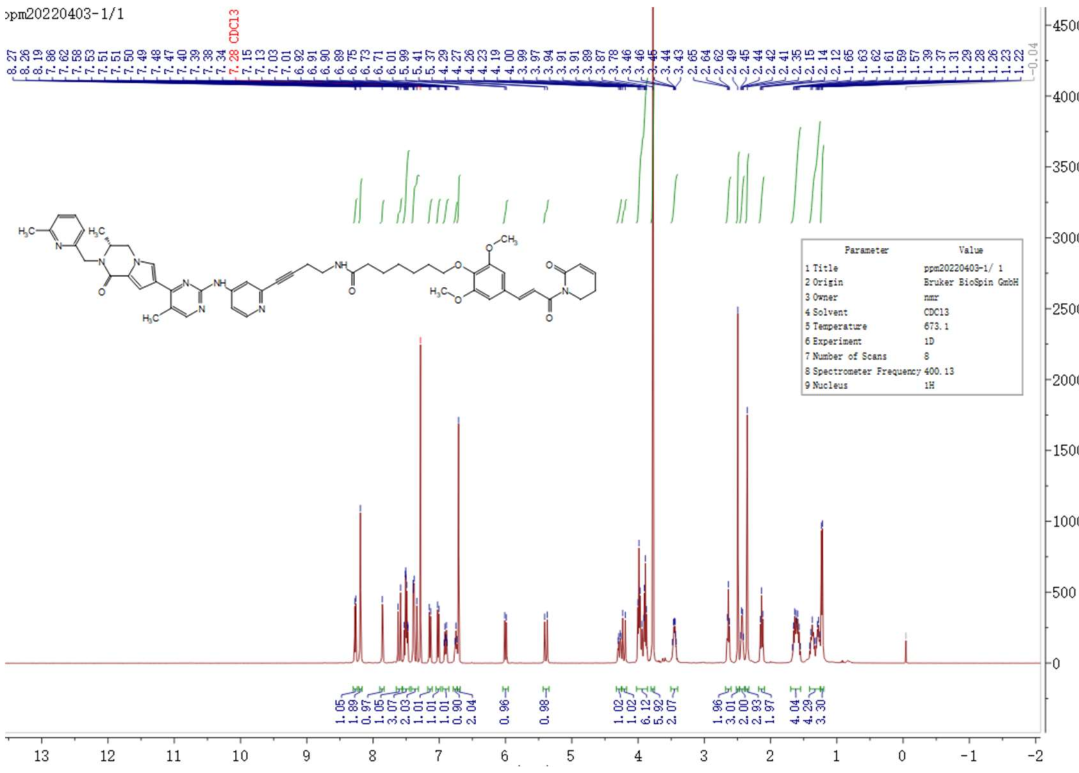

^{1}H NMR of **B3**

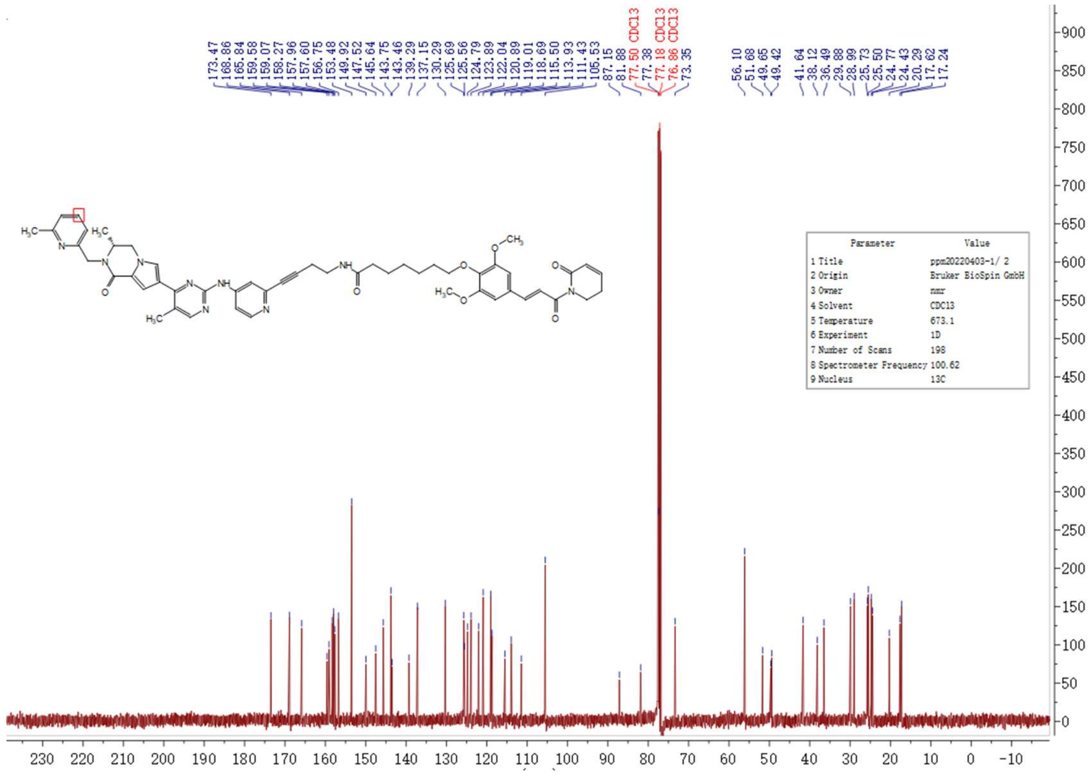

^{13}C NMR of **B3**

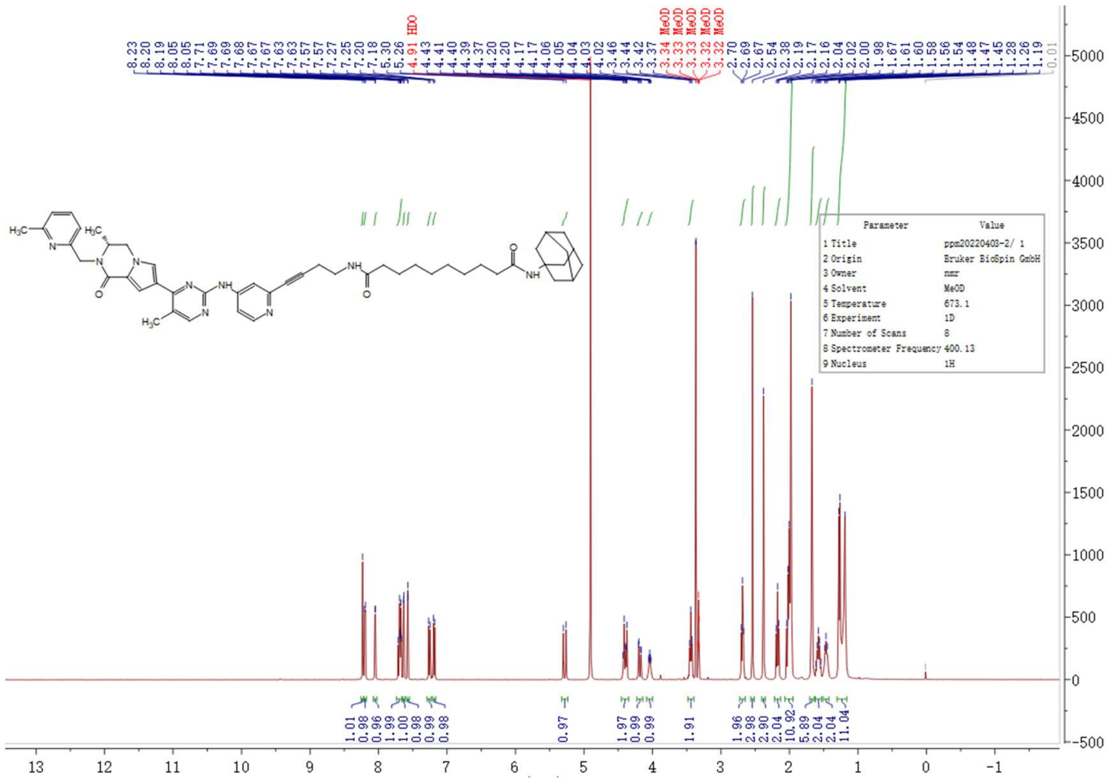

^{1}H NMR of **B4**

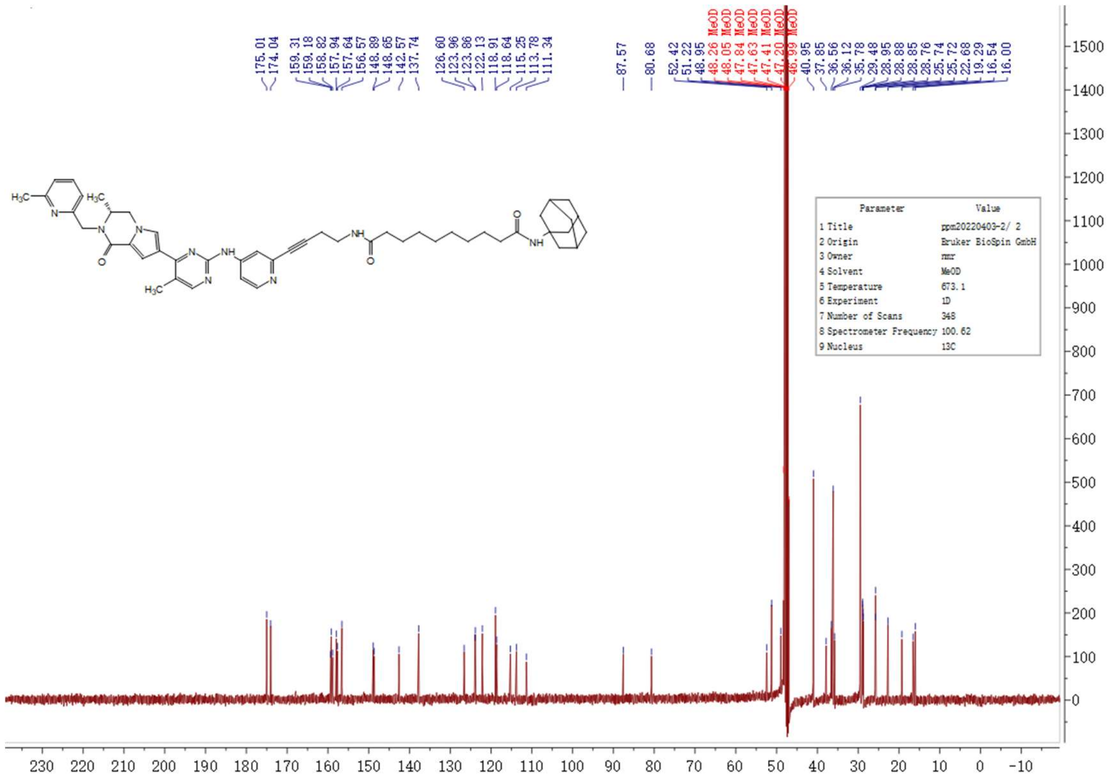

^{13}C NMR of **B4**

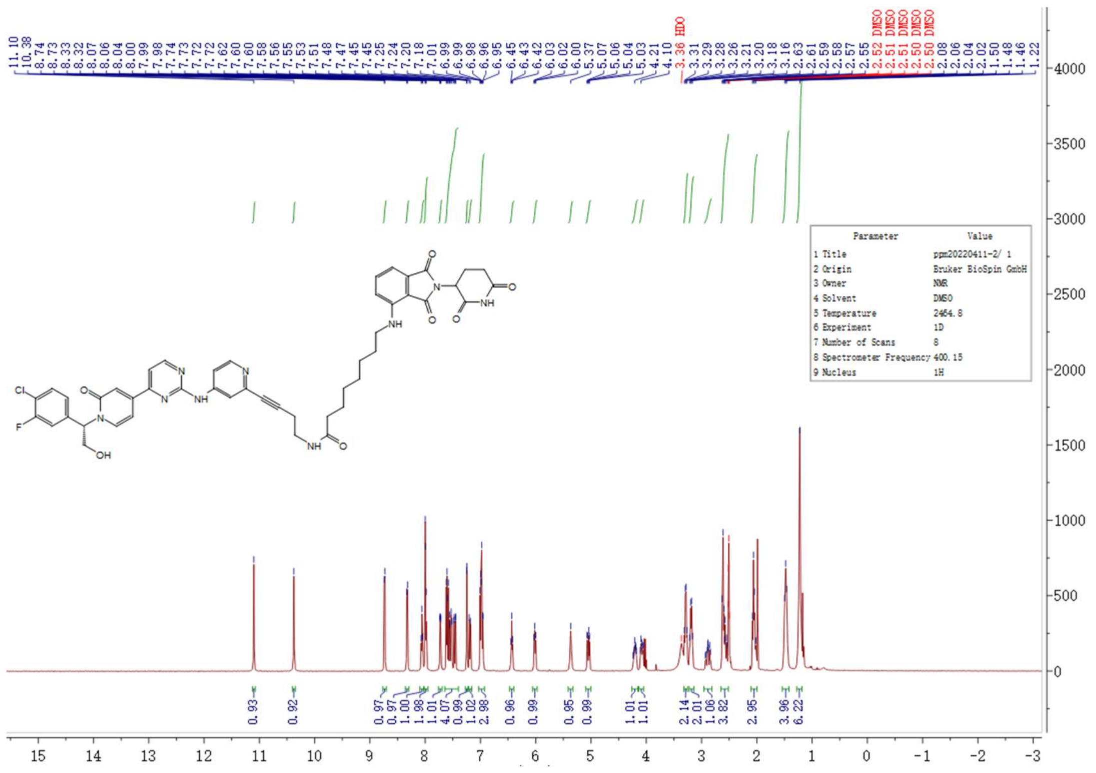

^{1}H NMR of **C1**

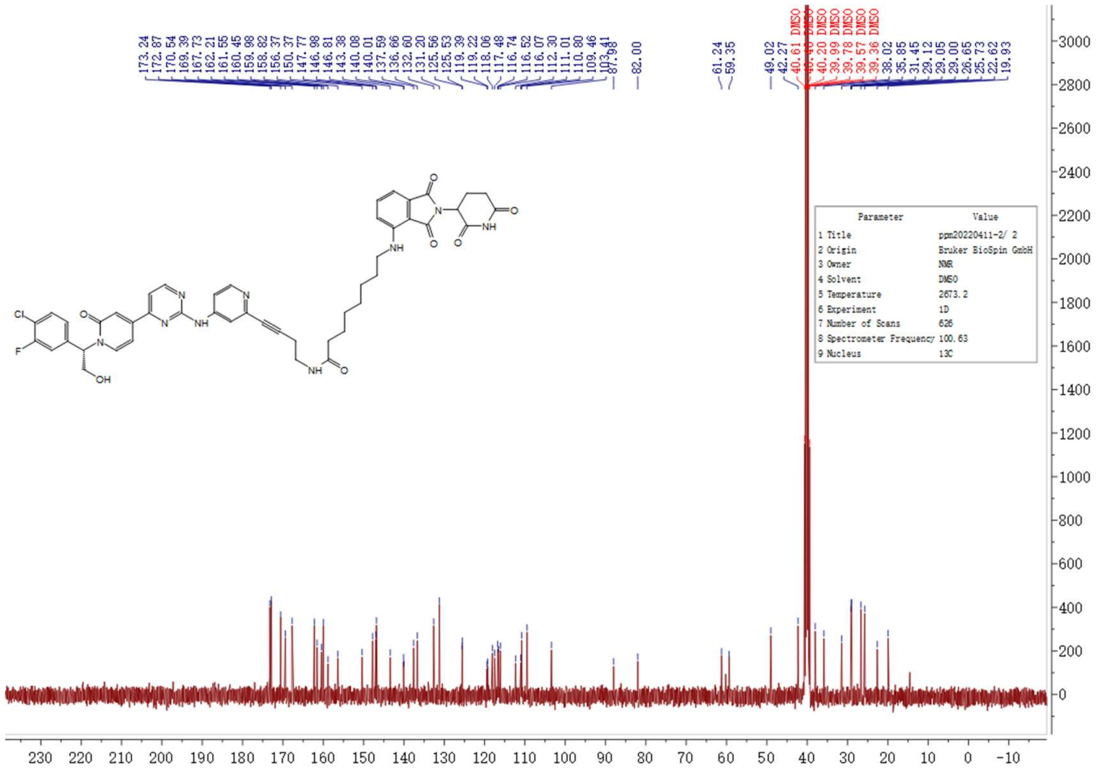

^{13}C NMR of **C1**

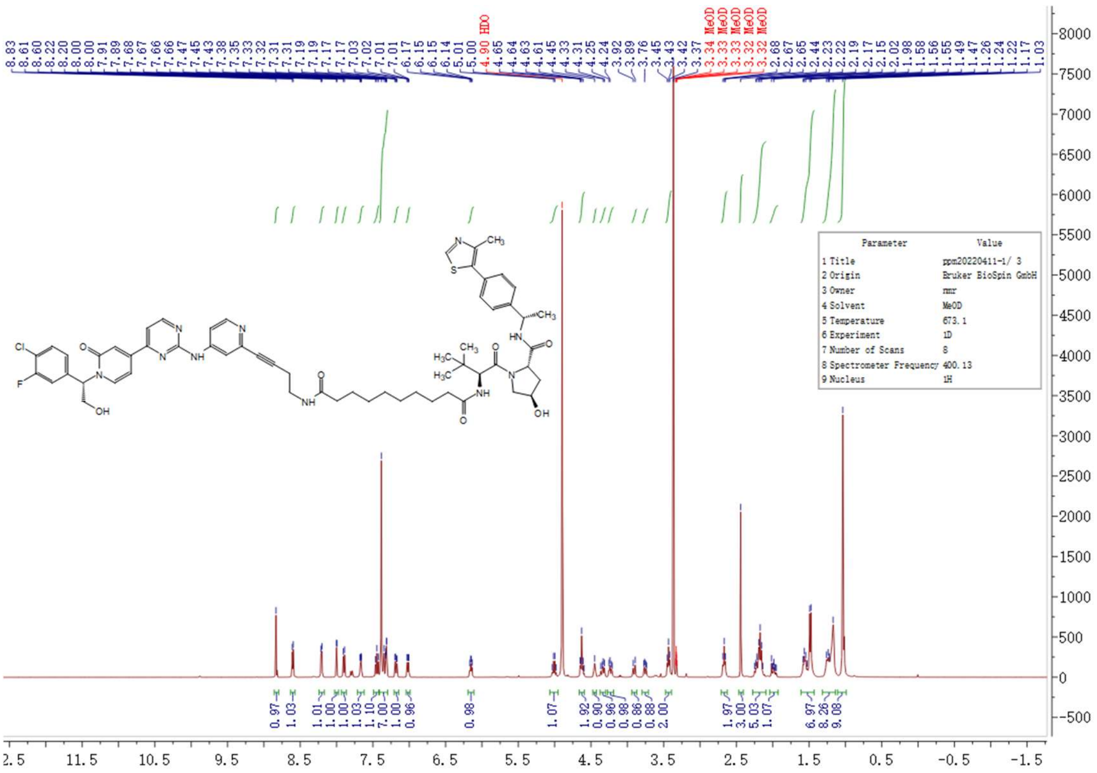

^{1}H NMR of **C2**

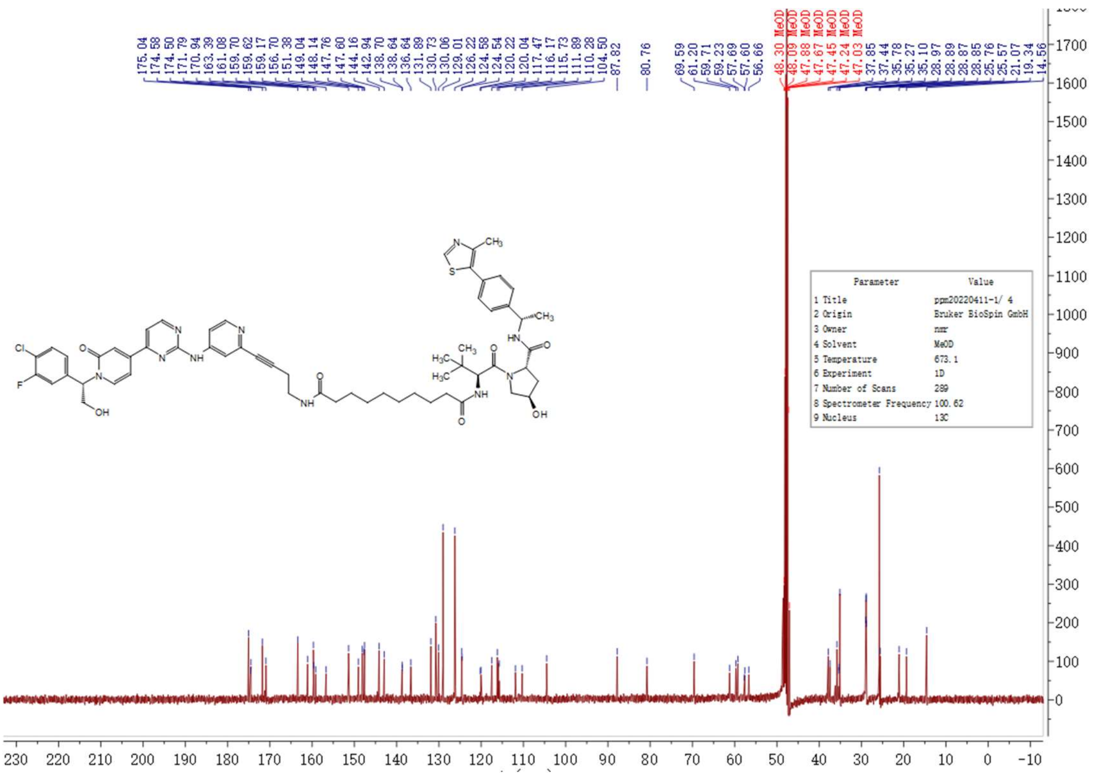

^{13}C NMR of **C2**

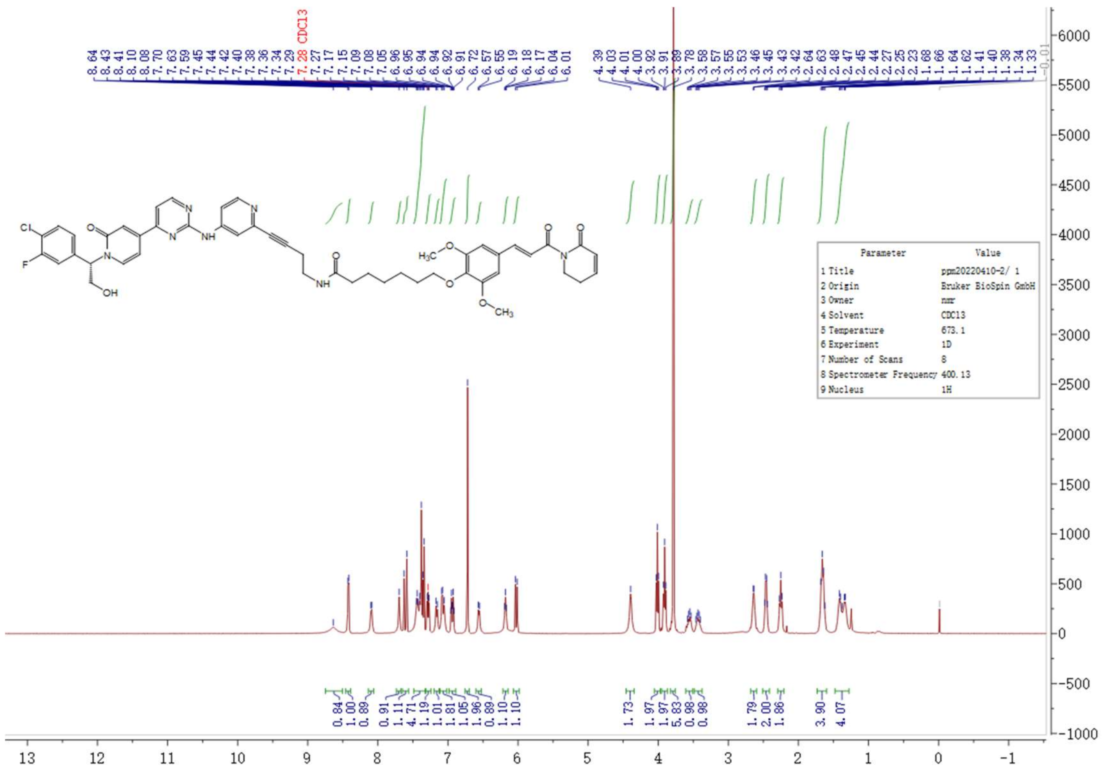

^{1}H NMR of **C3**

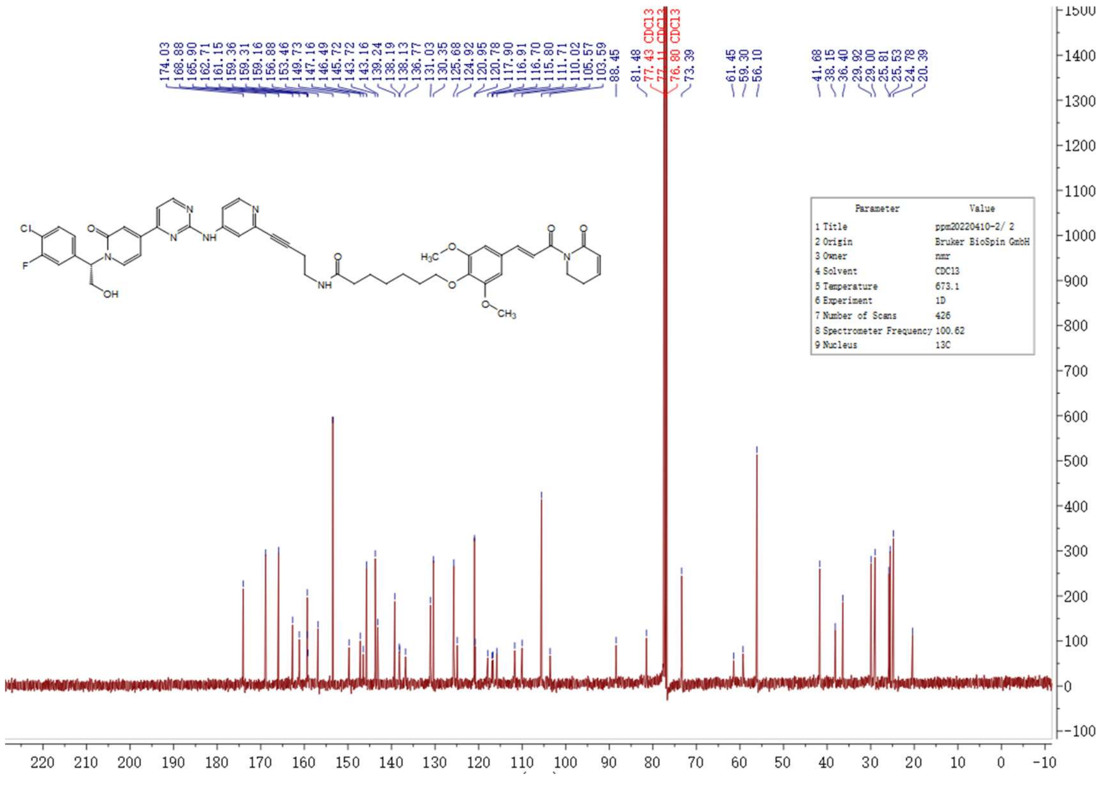

^{13}C NMR of **C3**

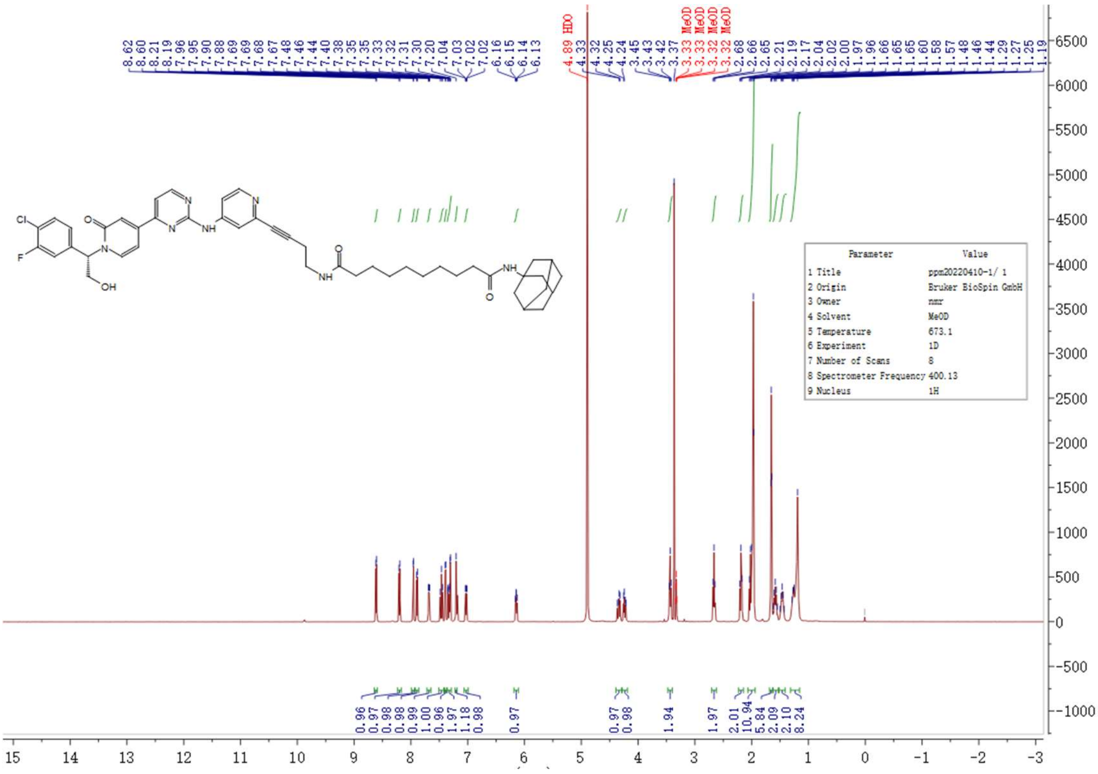

^{1}H NMR of **C4**

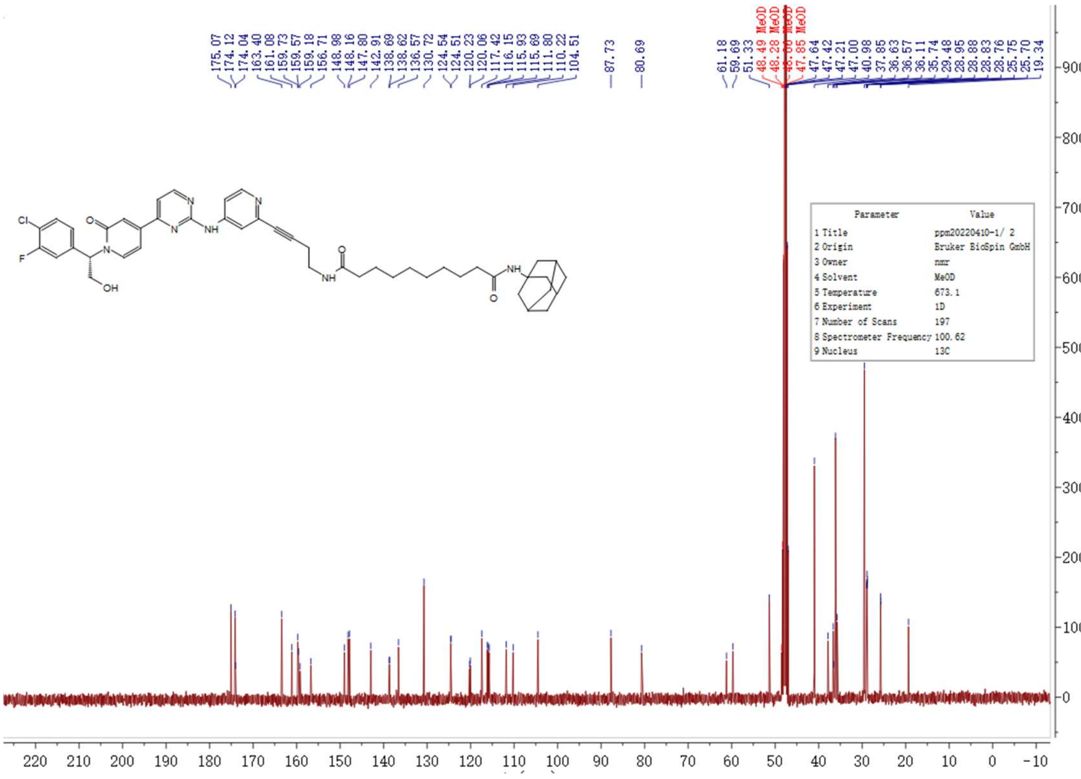

^{13}C NMR of **C4**

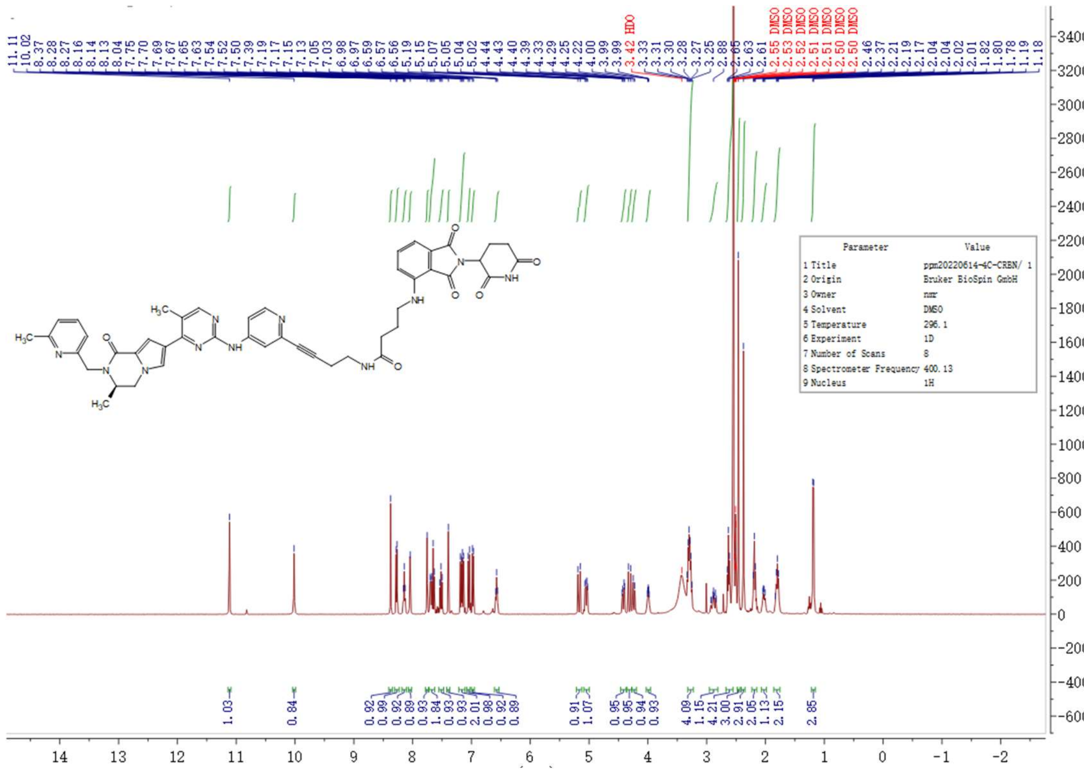

^{1}H NMR of **B1-4**

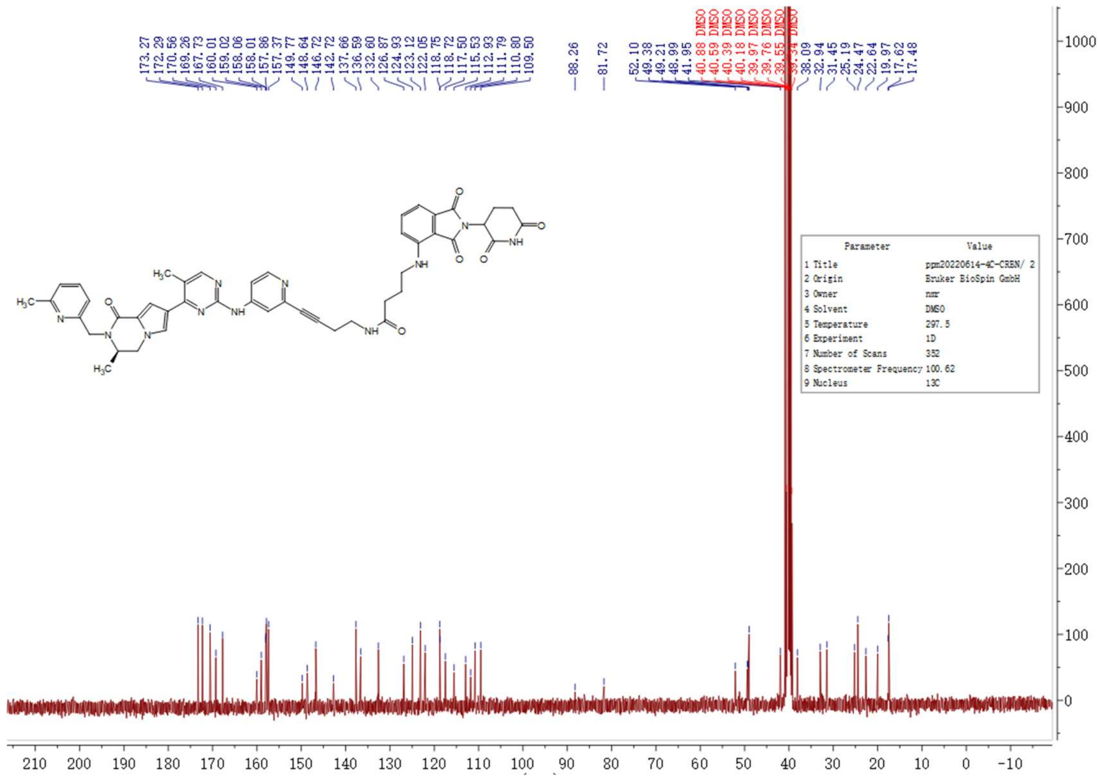

^{13}C NMR of **B1-4**

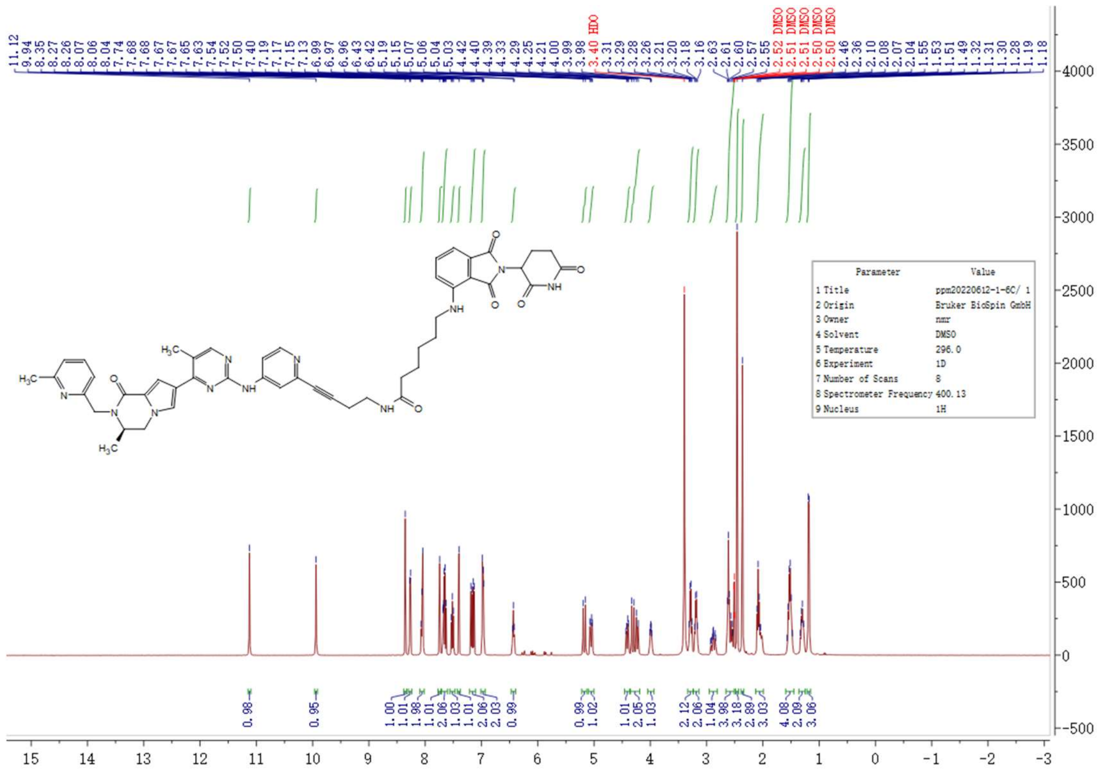

^{1}H NMR of **B1-6**

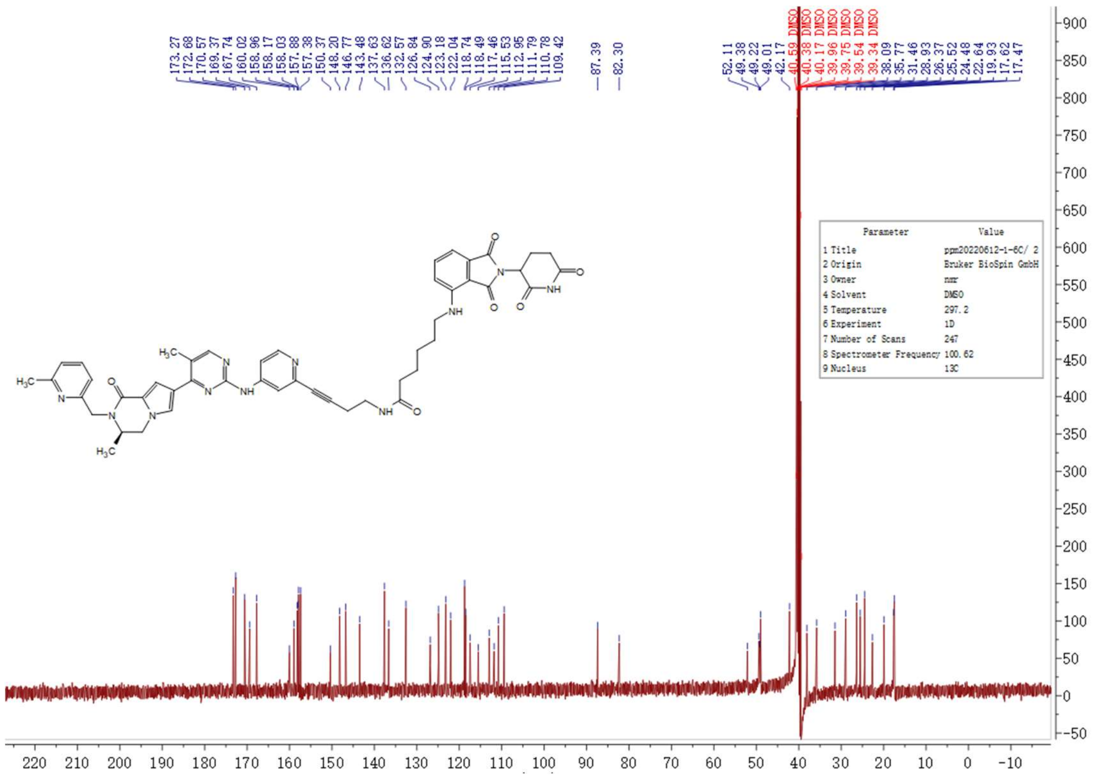

^{13}C NMR of **B1-6**

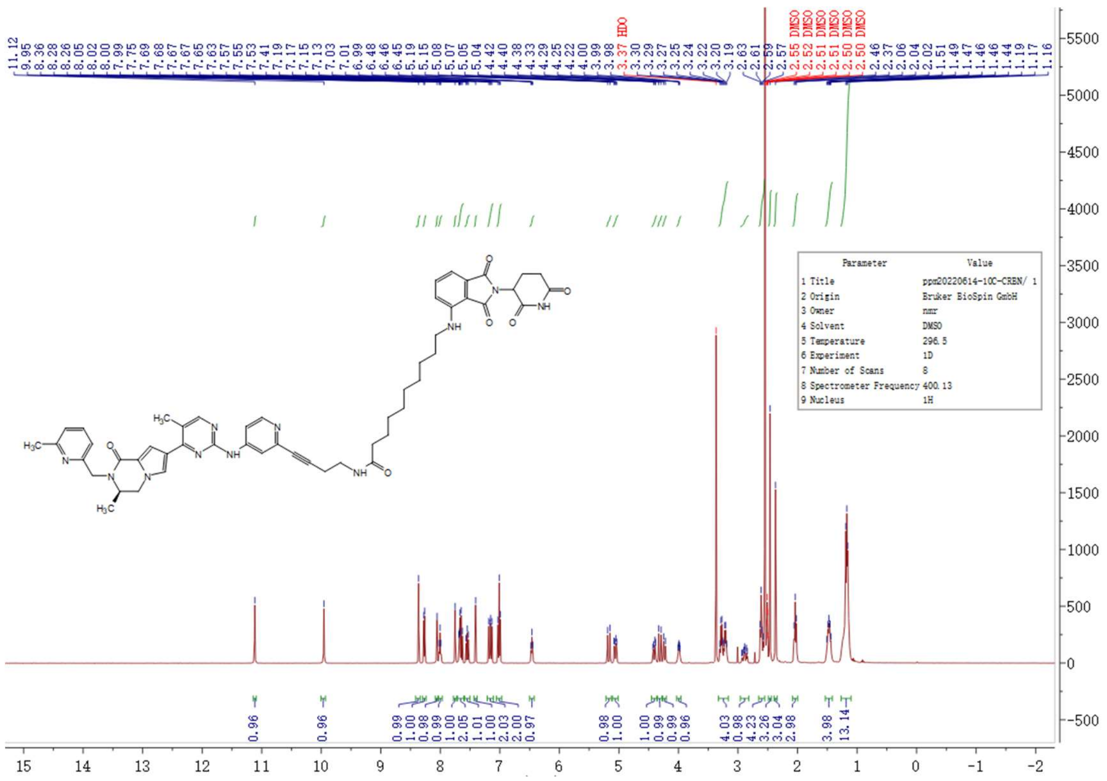

^{1}H NMR of **B1-10**

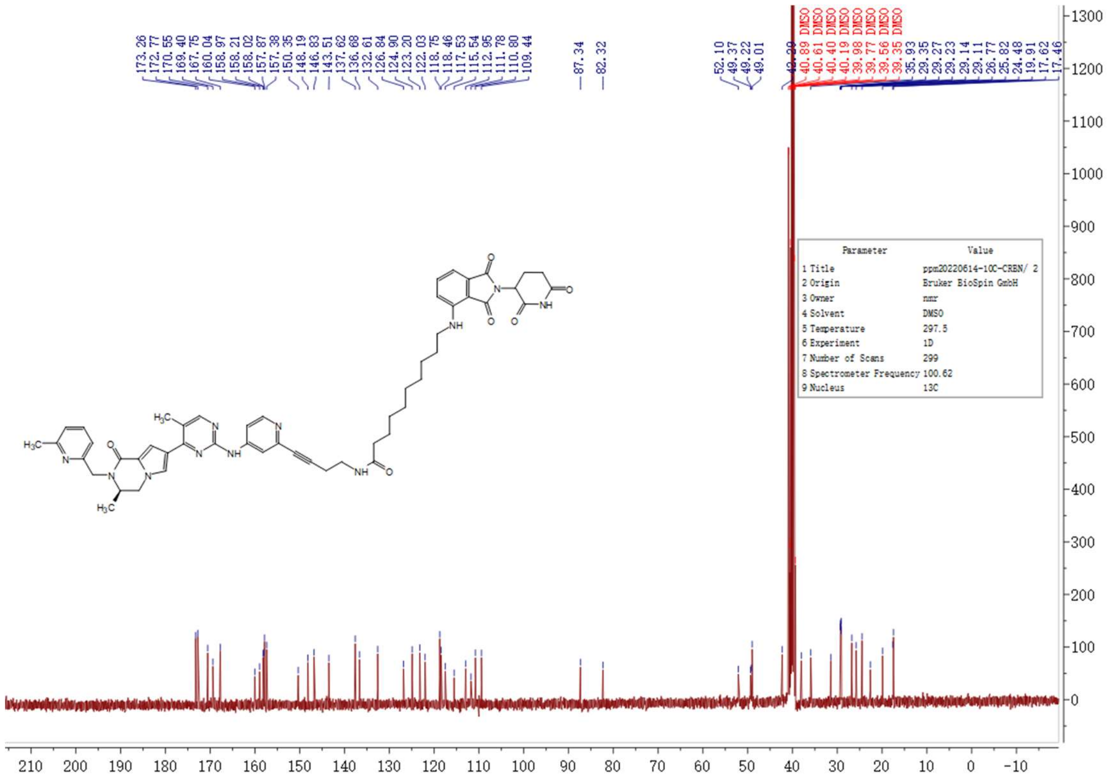

^{13}C NMR of **B1-10**

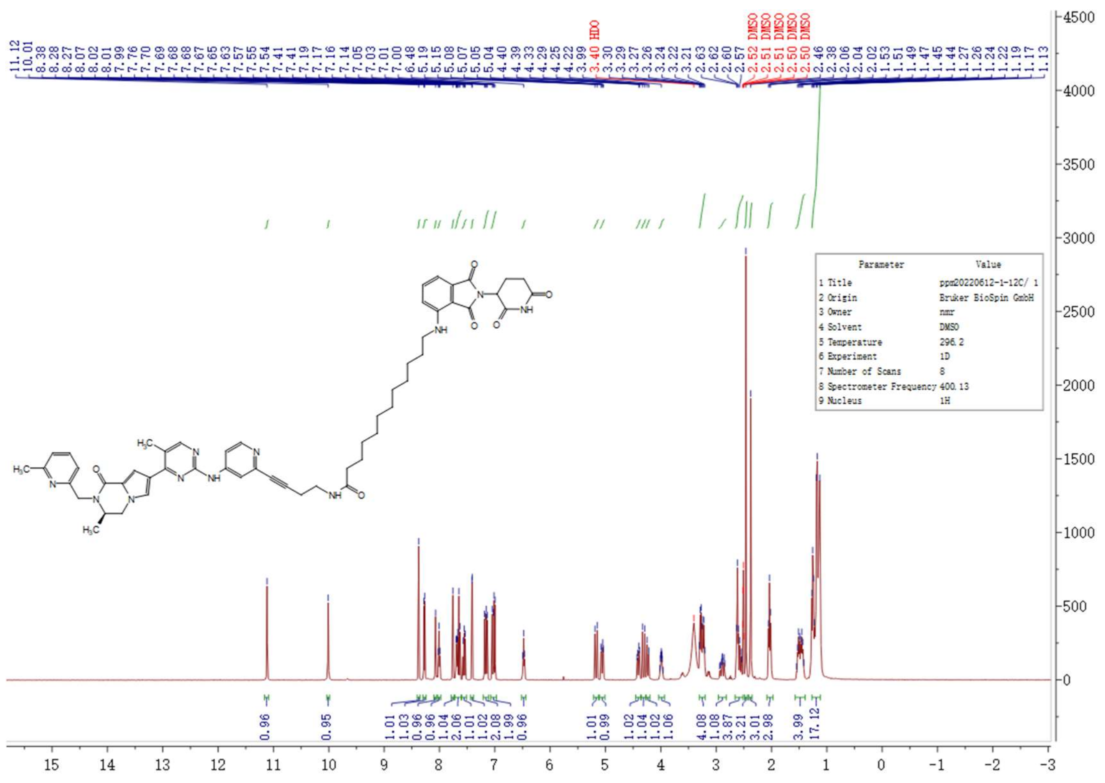

^{1}H NMR of **B1-12**

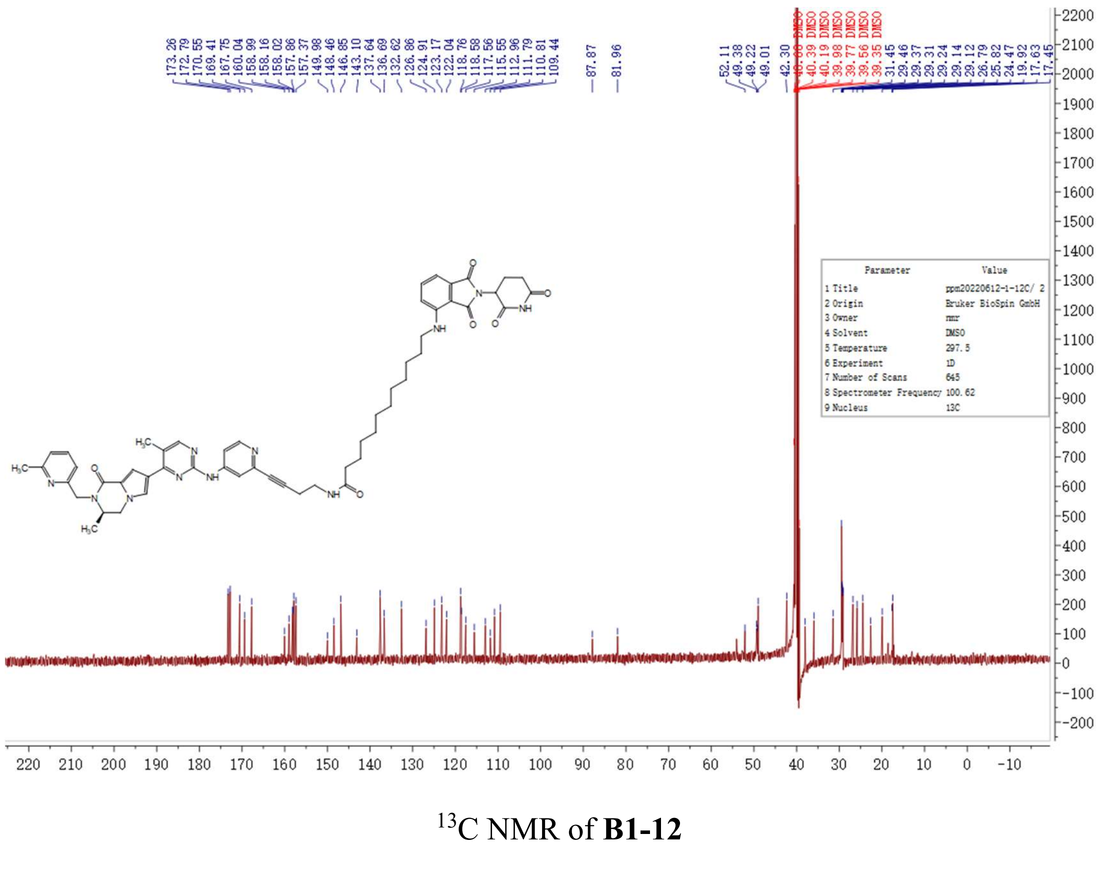

^{13}C NMR of **B1-12**

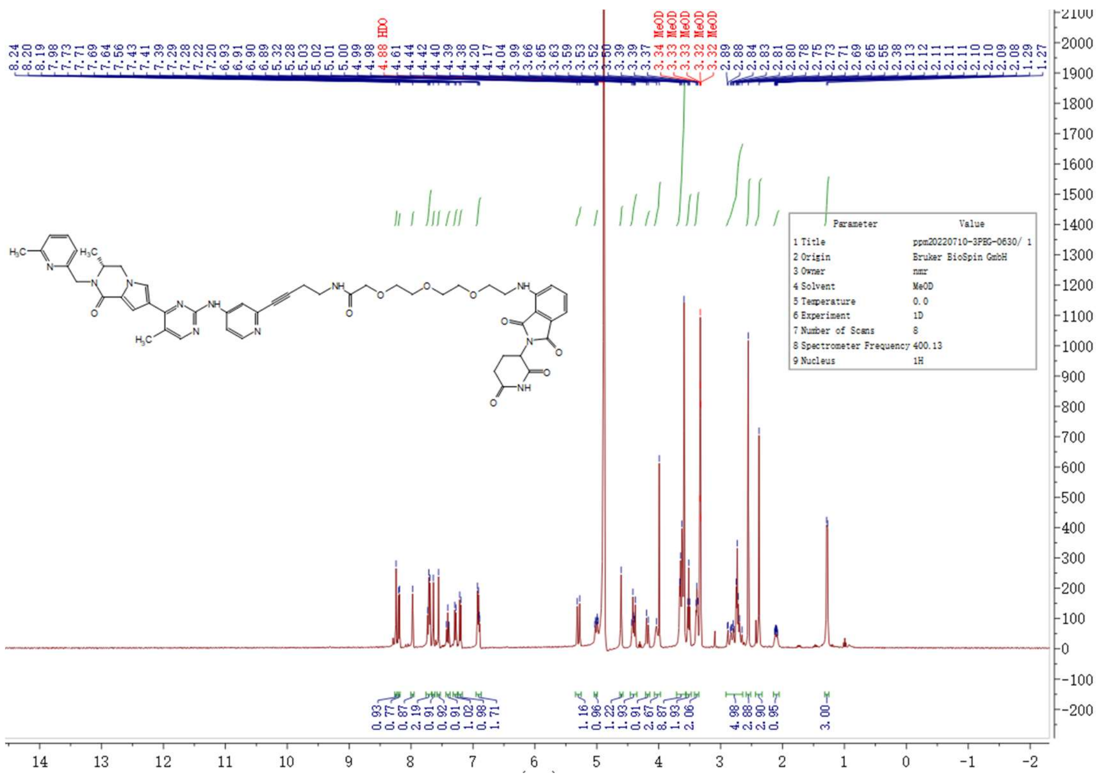

^{1}H NMR of **B1-3P**

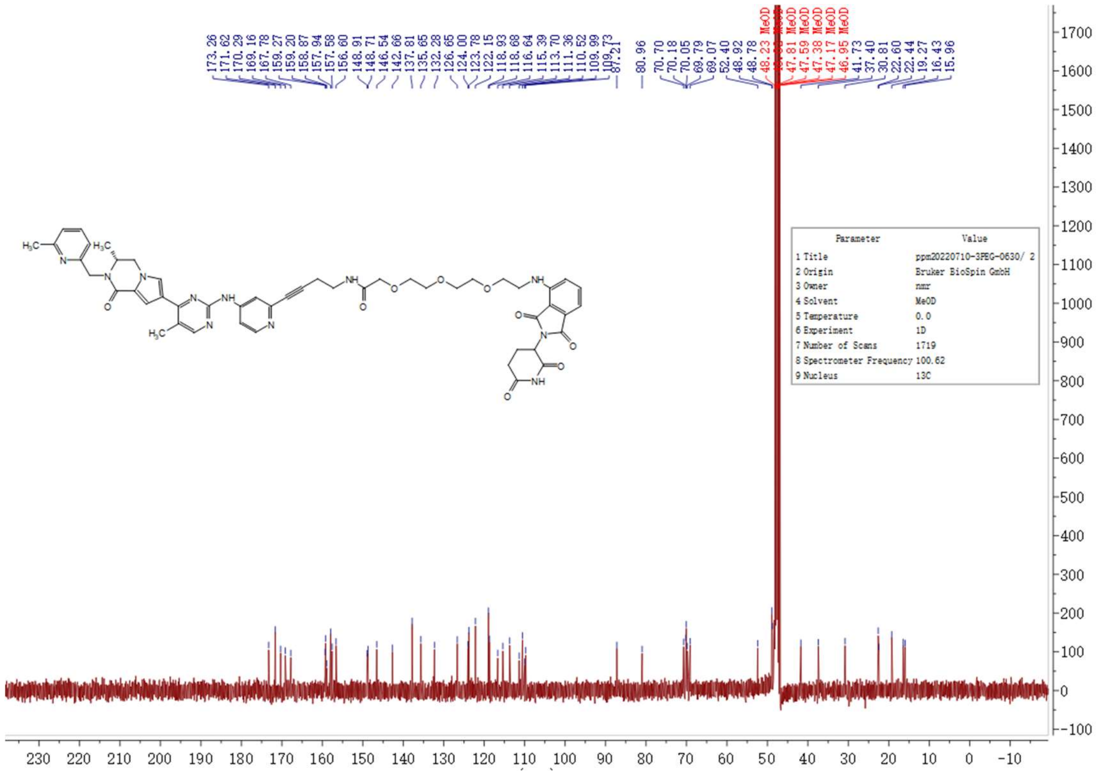

^{13}C NMR of **B1-3P**

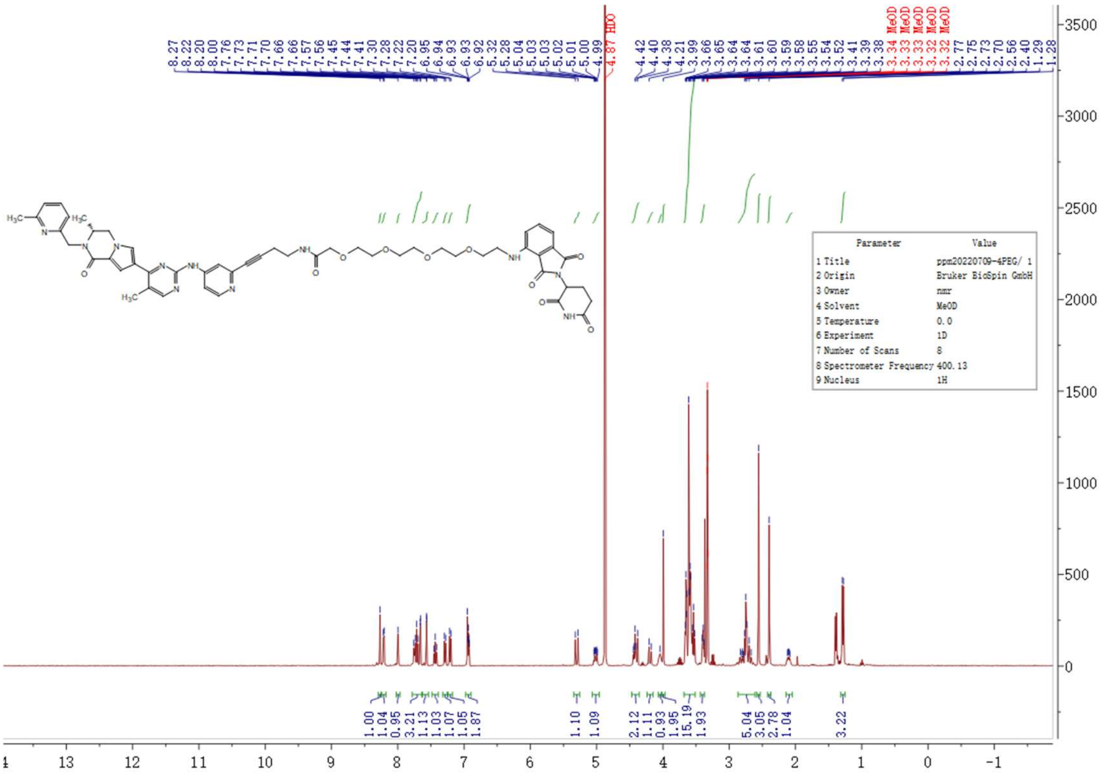

^{1}H NMR of **B1-4P**

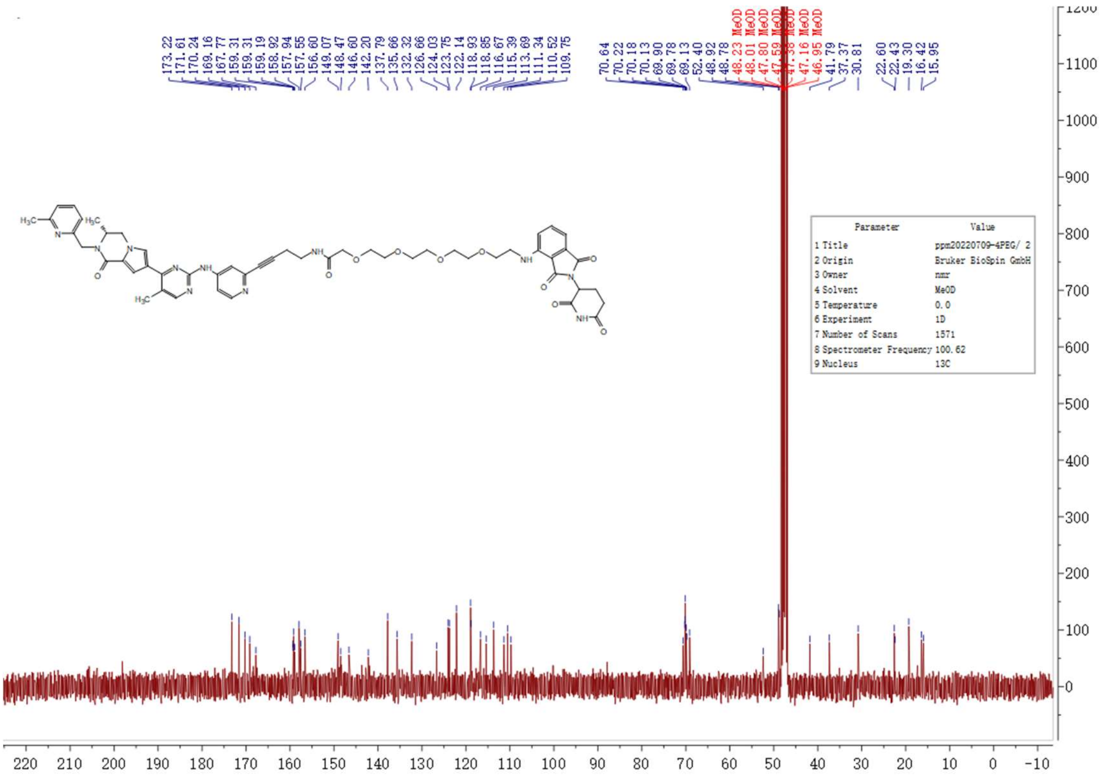

^{13}C NMR of **B1-4P**

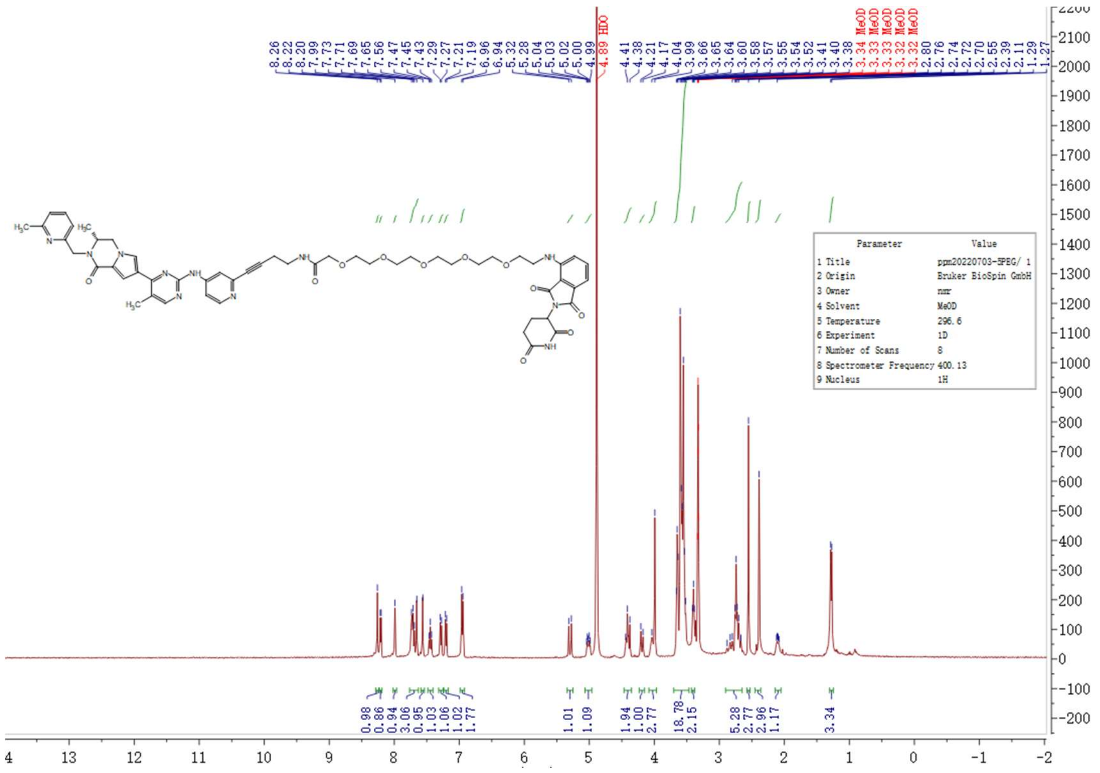

^{1}H NMR of **B1-5P**

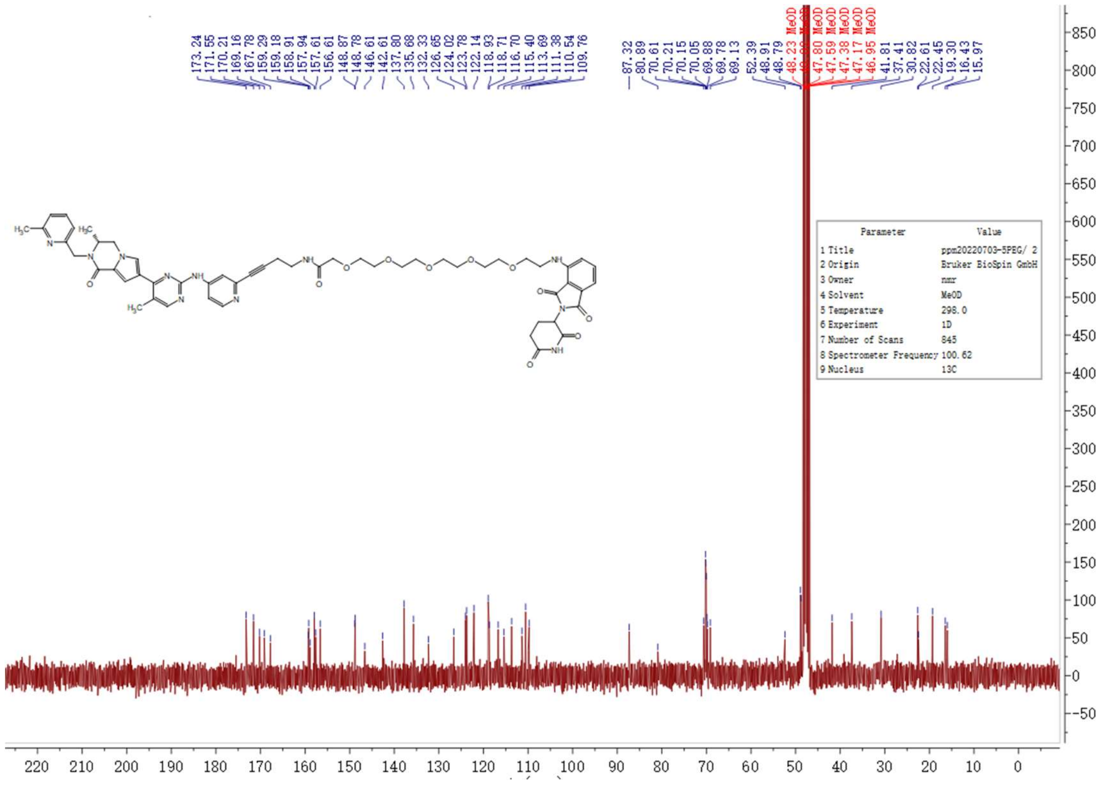

^{13}C NMR of **B1-5P**

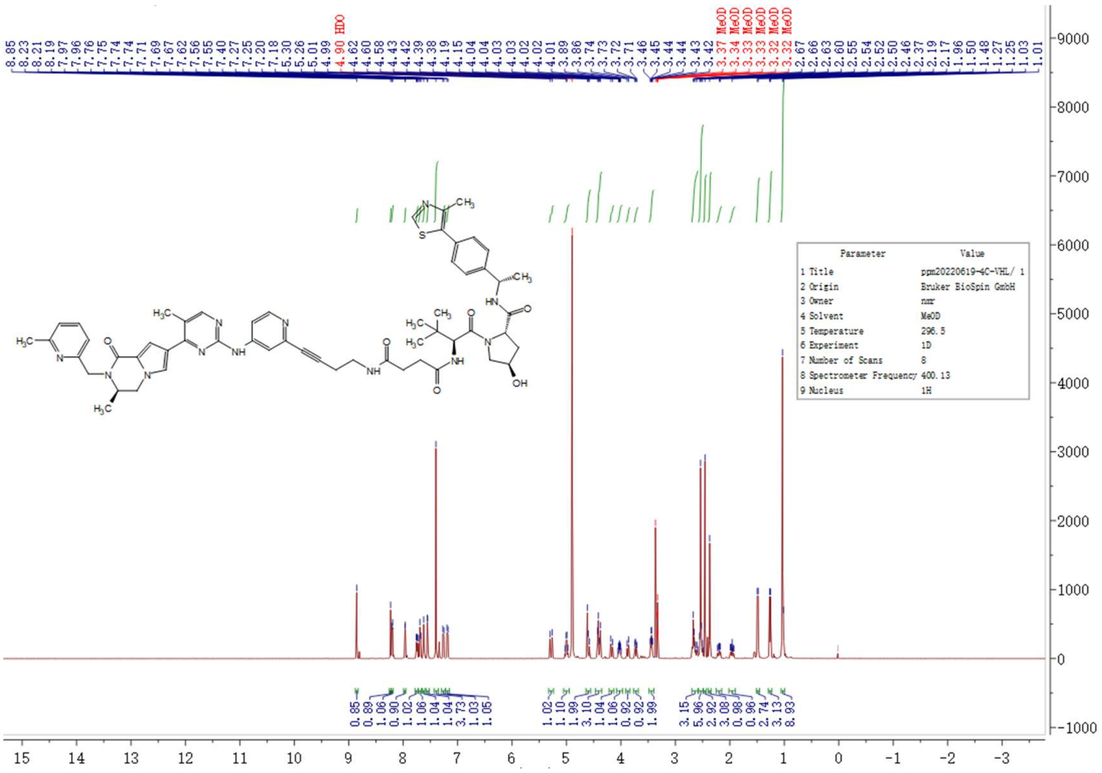

^{1}H NMR of **B2-4**

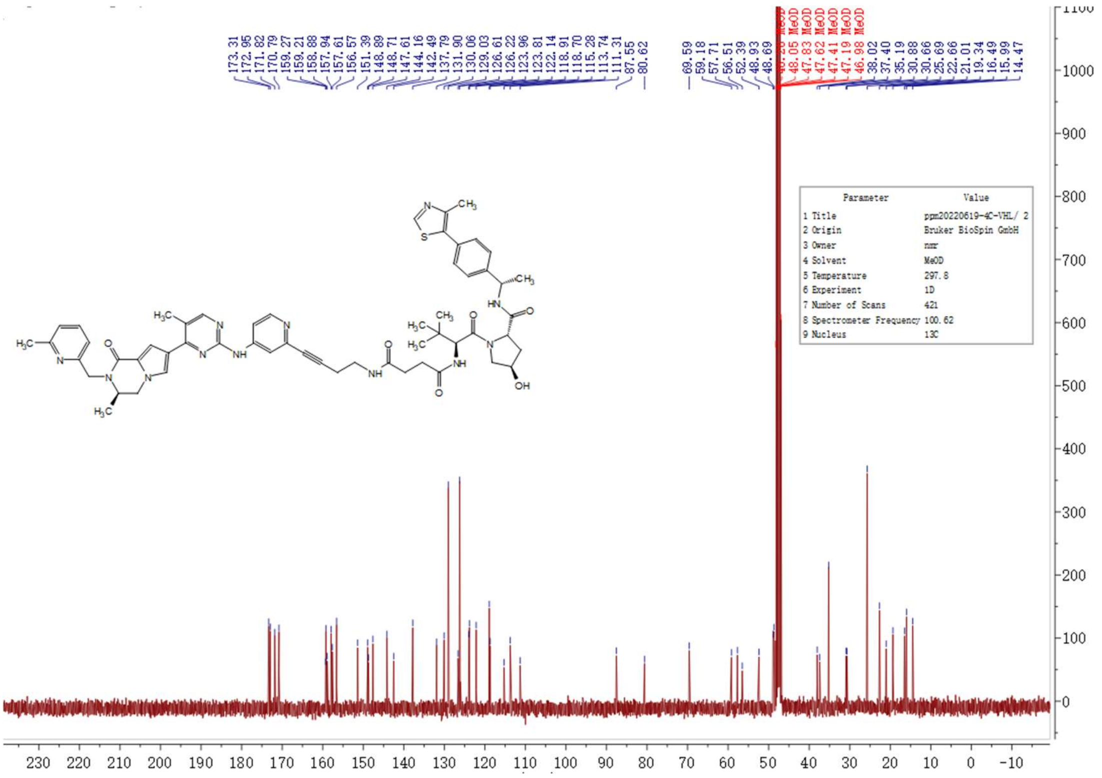

^{13}C NMR of **B2-4**

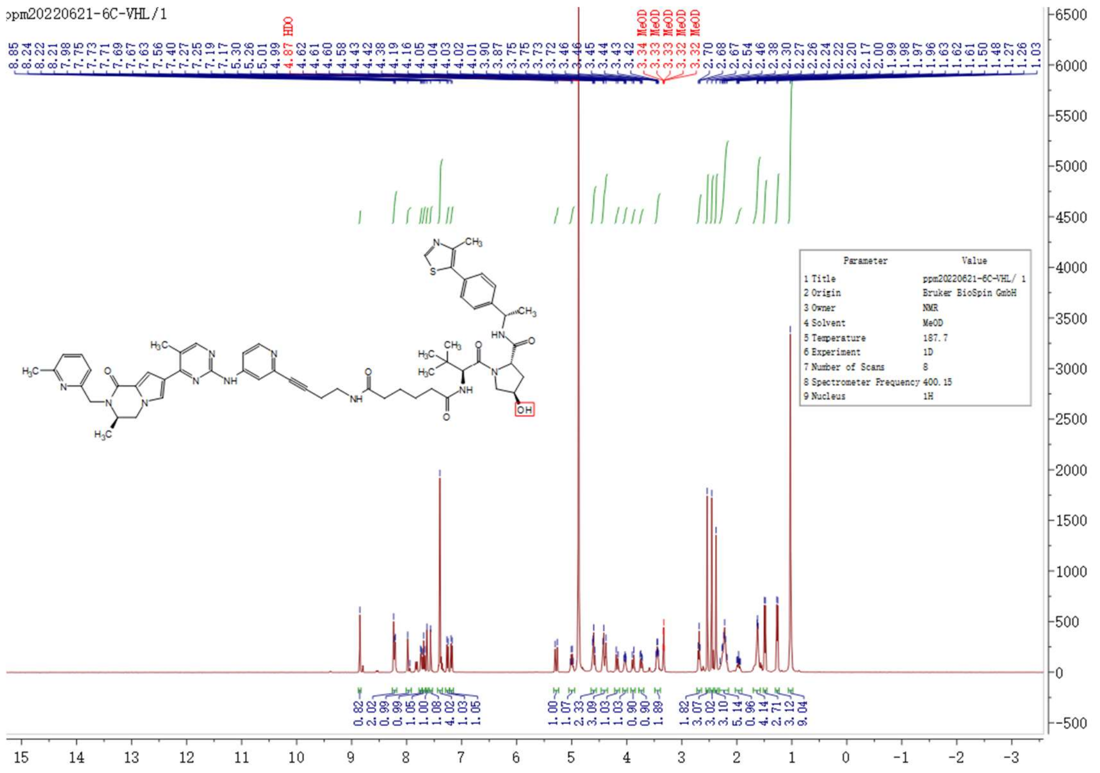

^{1}H NMR of **B2-6**

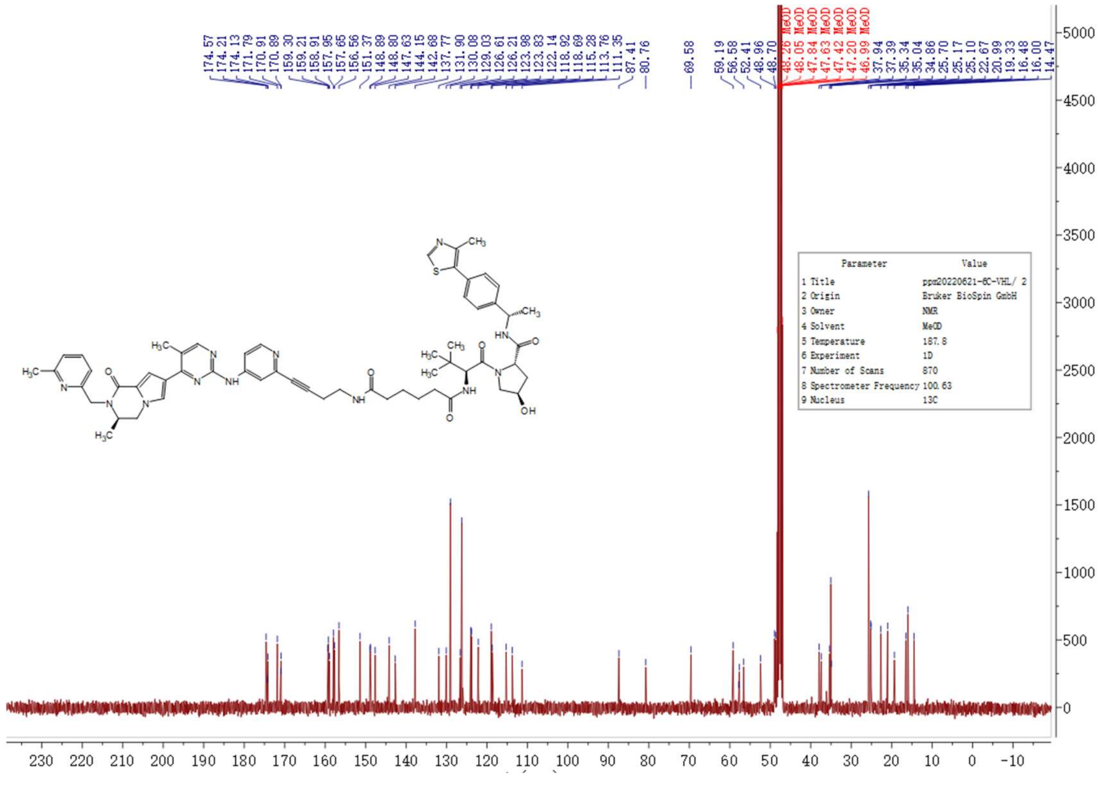

^{13}C NMR of **B2-6**

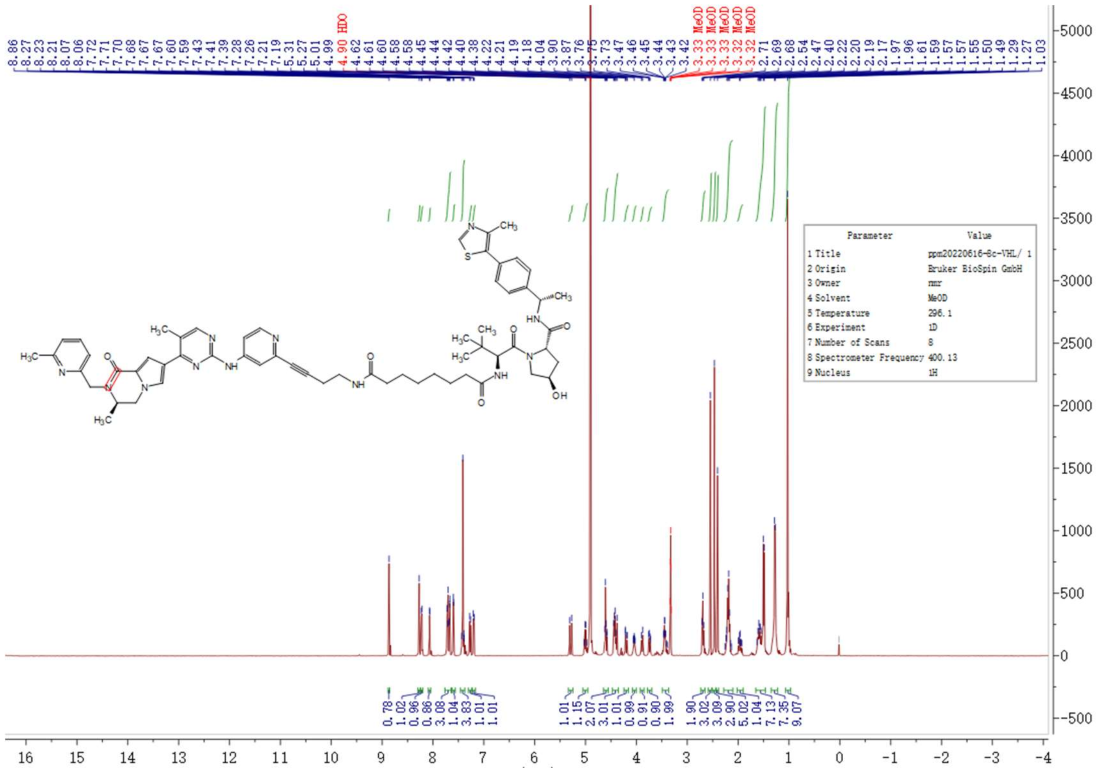

^{1}H NMR of **B2-8**

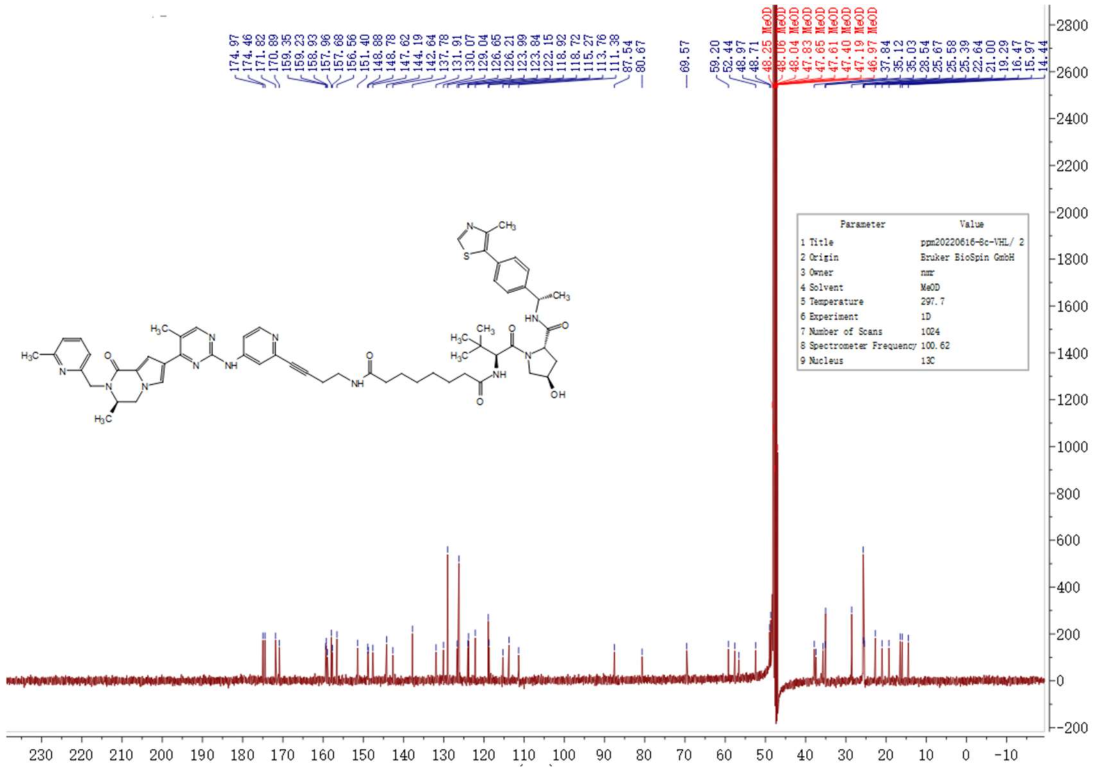

^{13}C NMR of **B2-8**

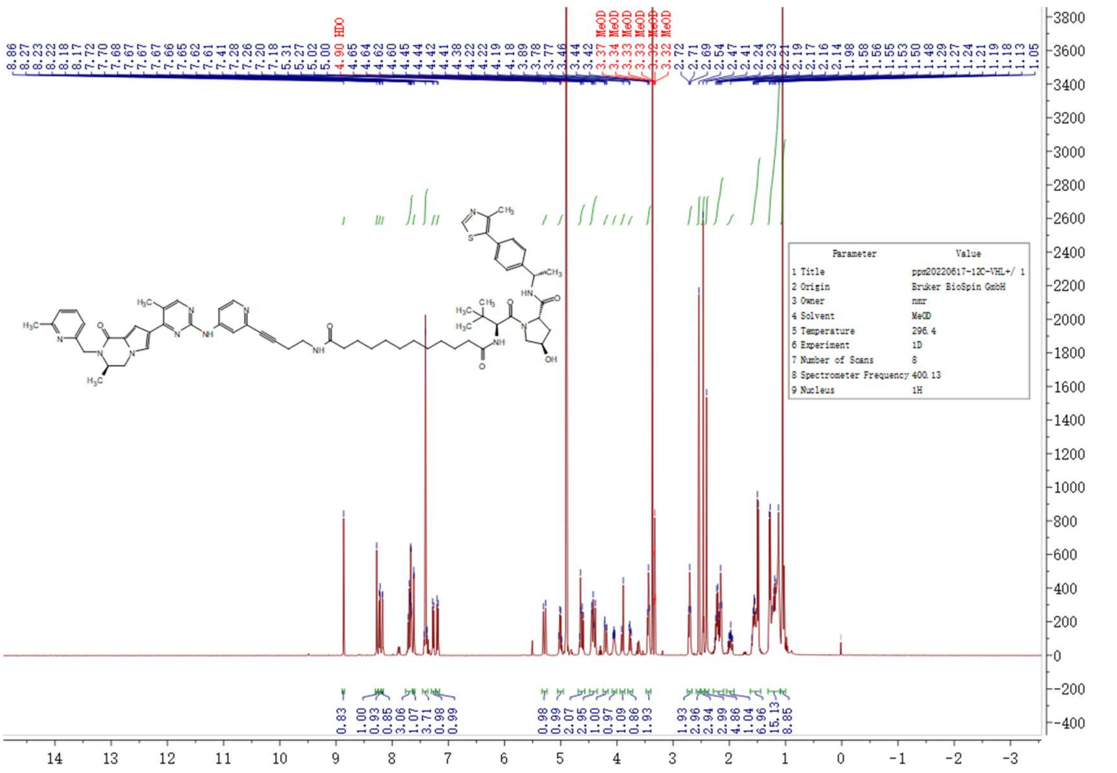

^{1}H NMR of **B2-12**

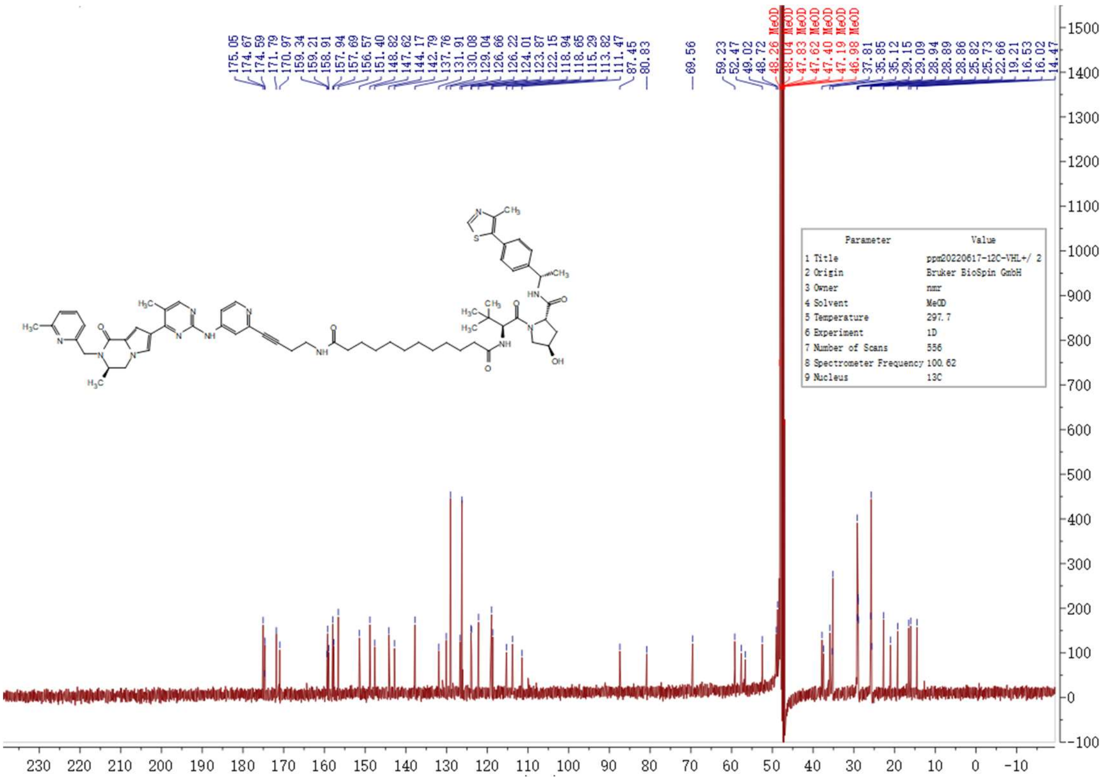

^{13}C NMR of **B2-12**

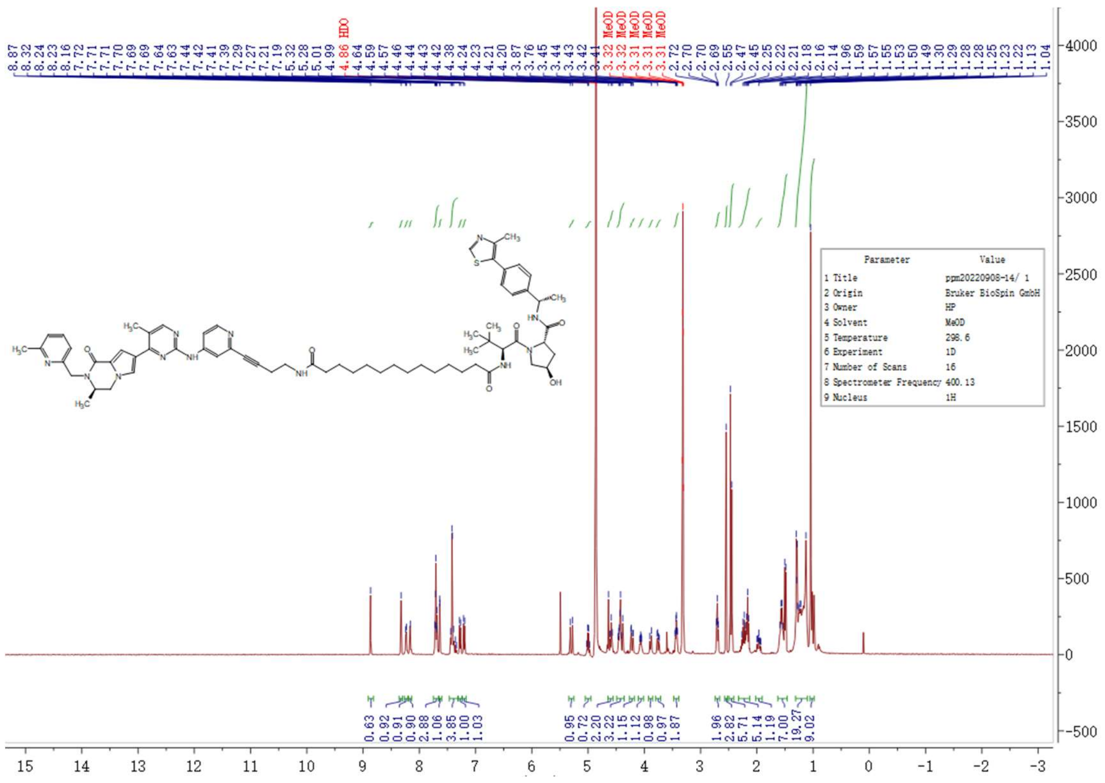

^{1}H NMR of **B2-14**

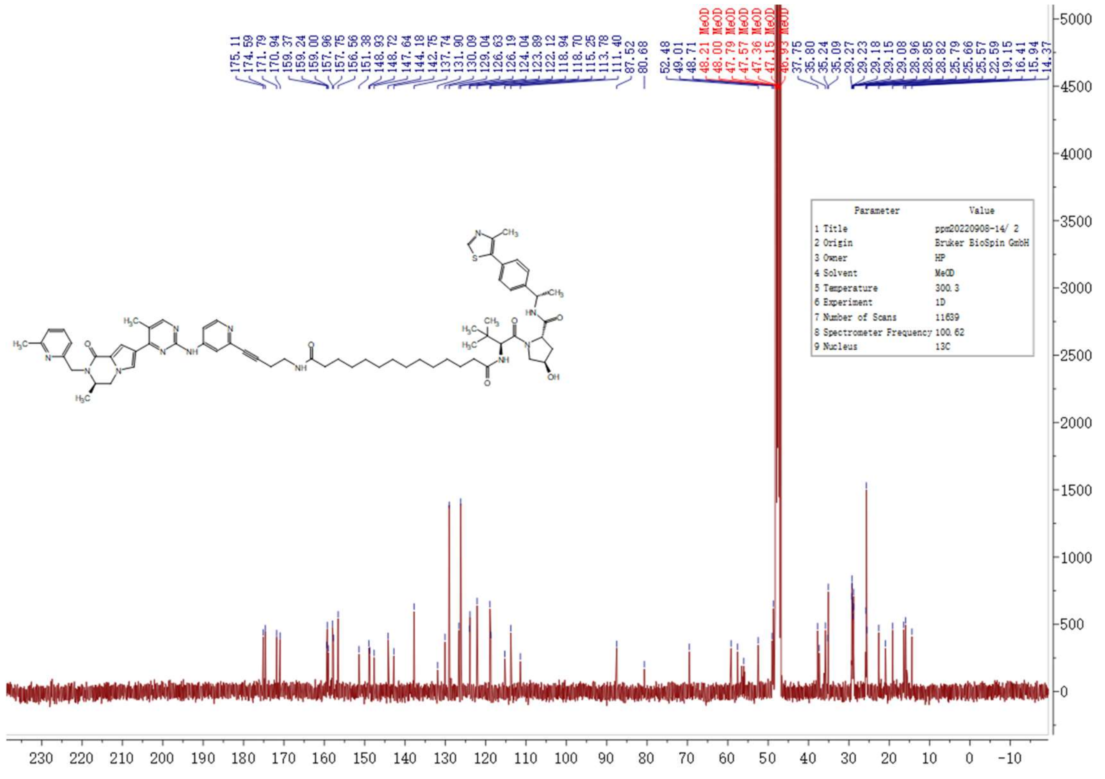

^{13}C NMR of **B2-14**

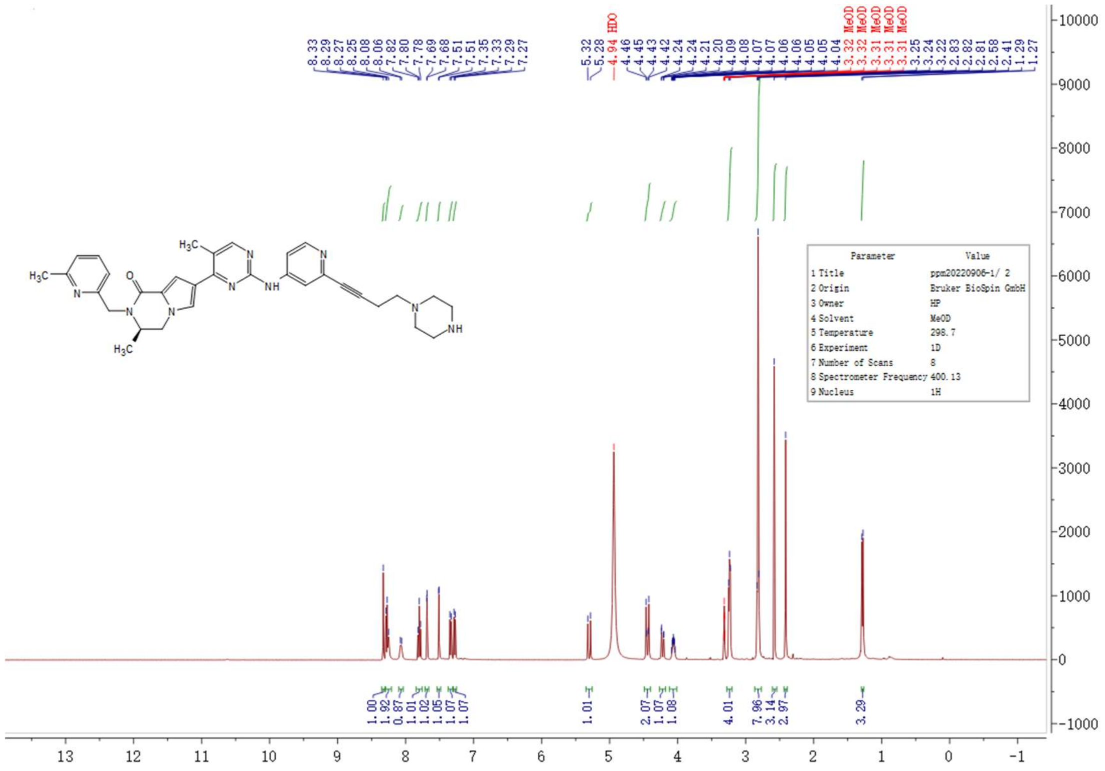

^{1}H NMR of **B-P**

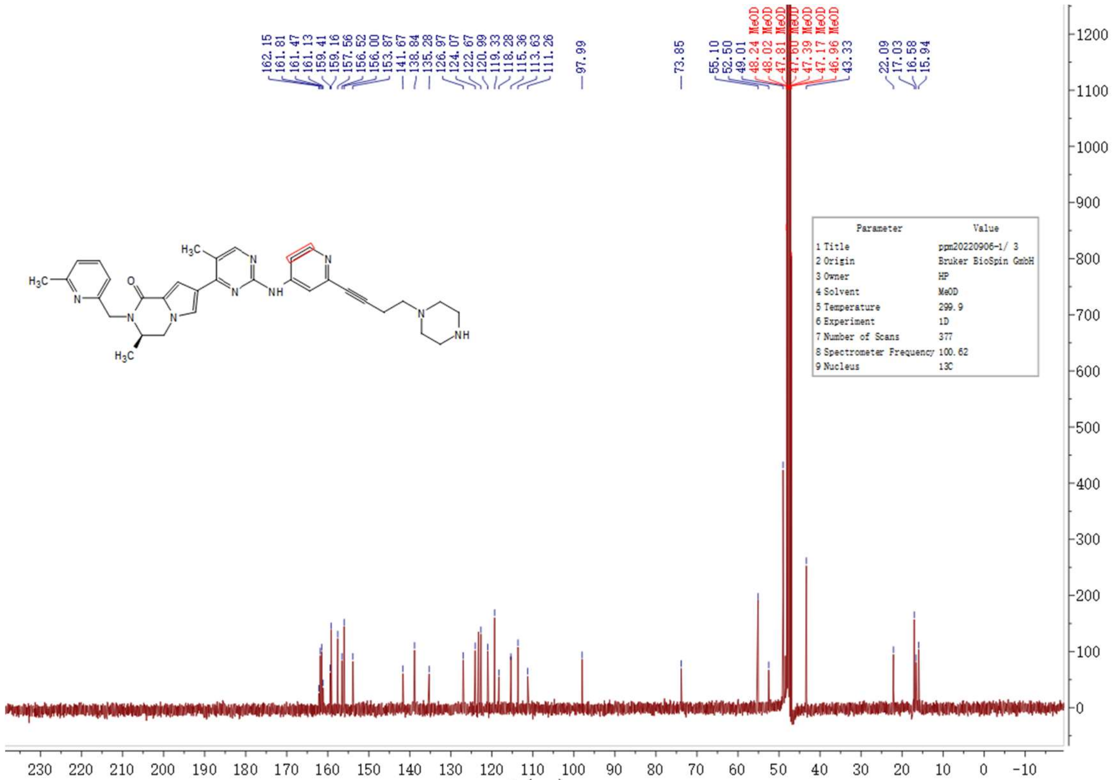

^{13}C NMR of **B-P**

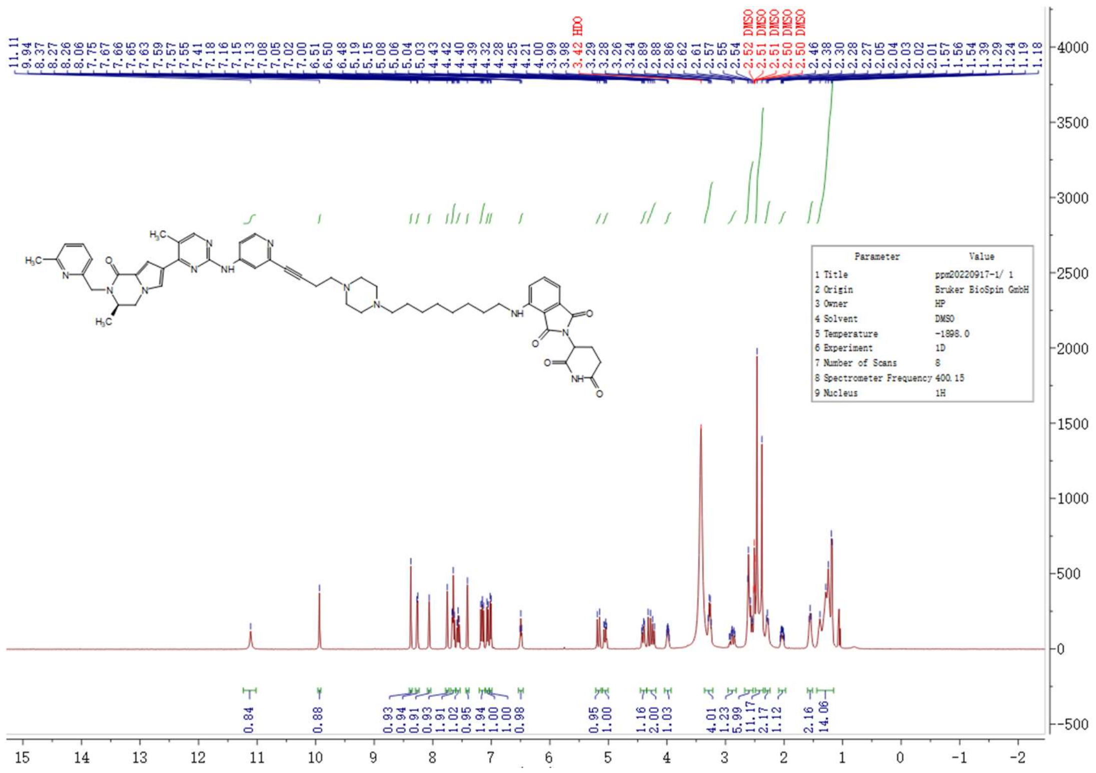

^{1}H NMR of **B1-10P**

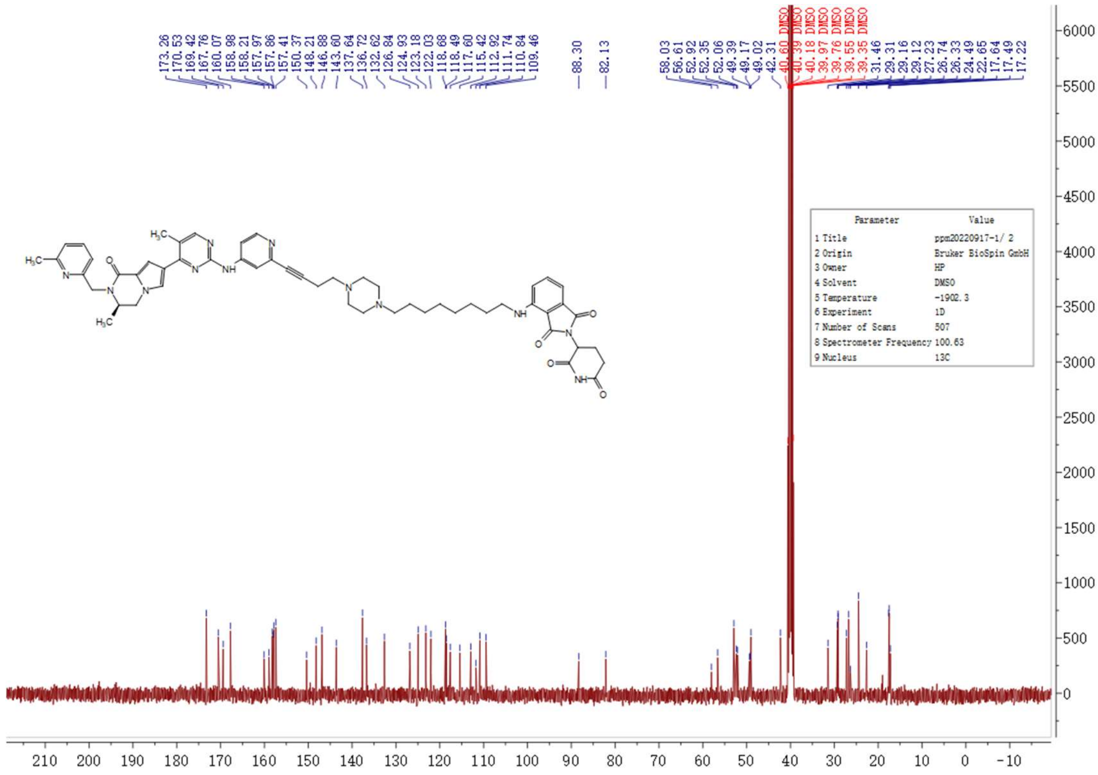

^{13}C NMR of **B1-10P**

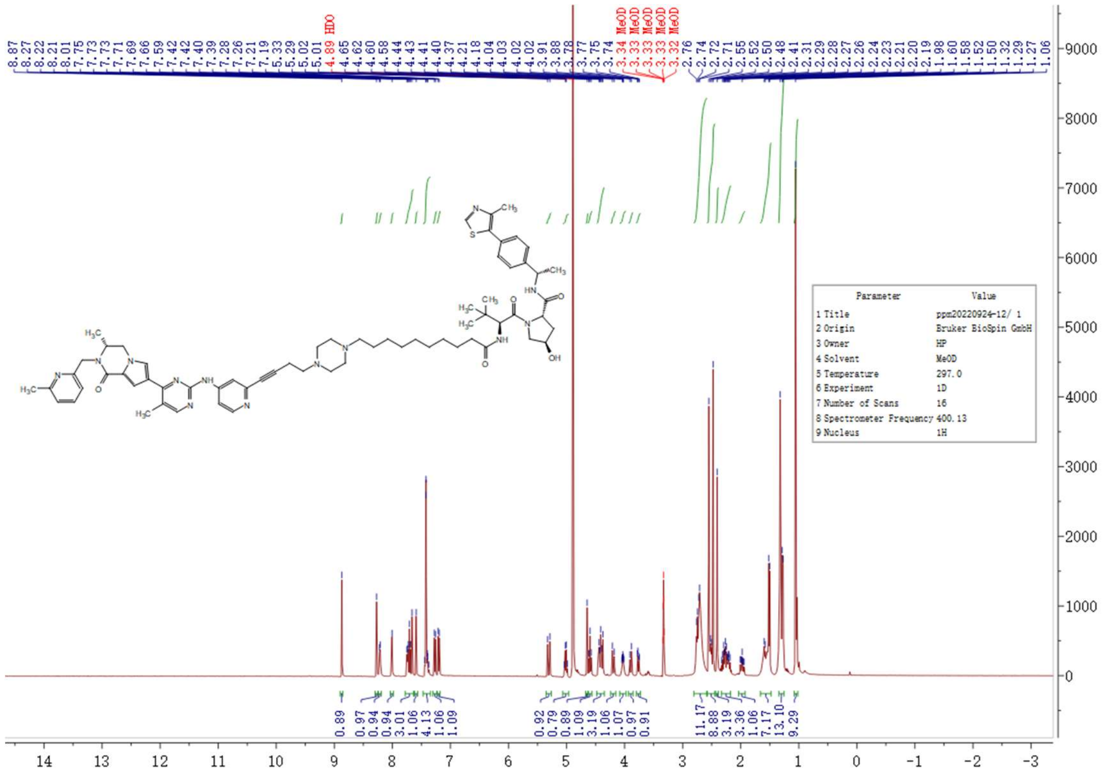

^{1}H NMR of **B2-12P**

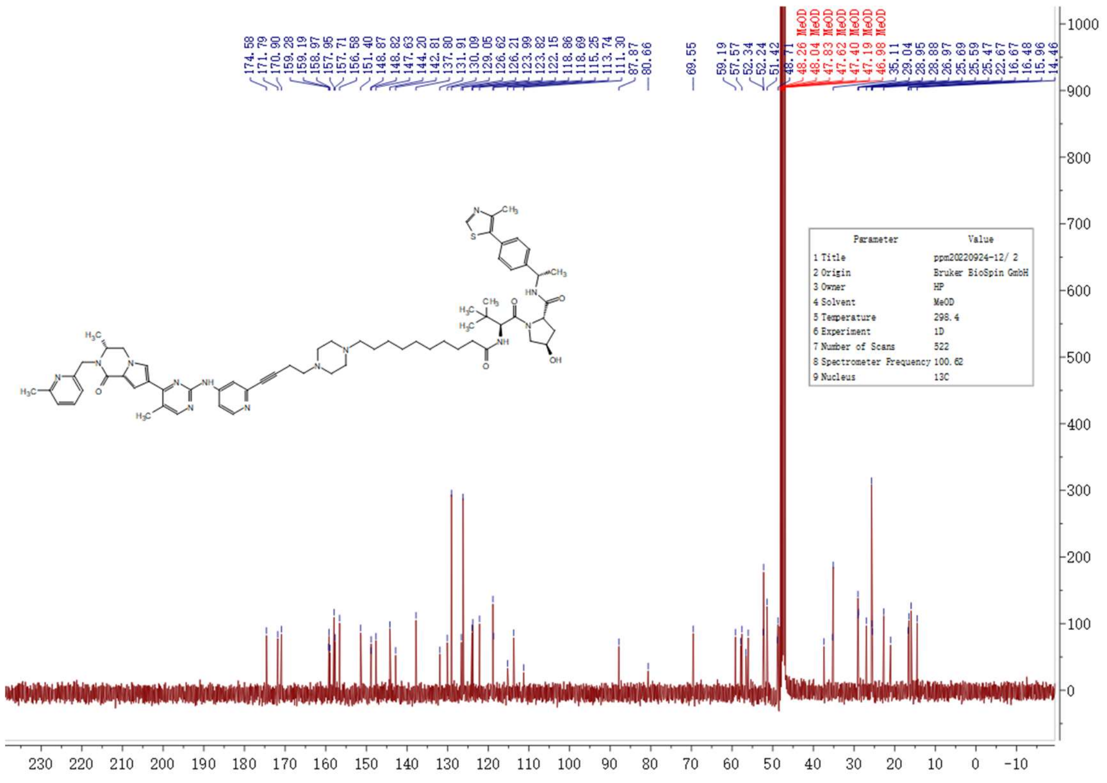

^{13}C NMR of **B2-12P**

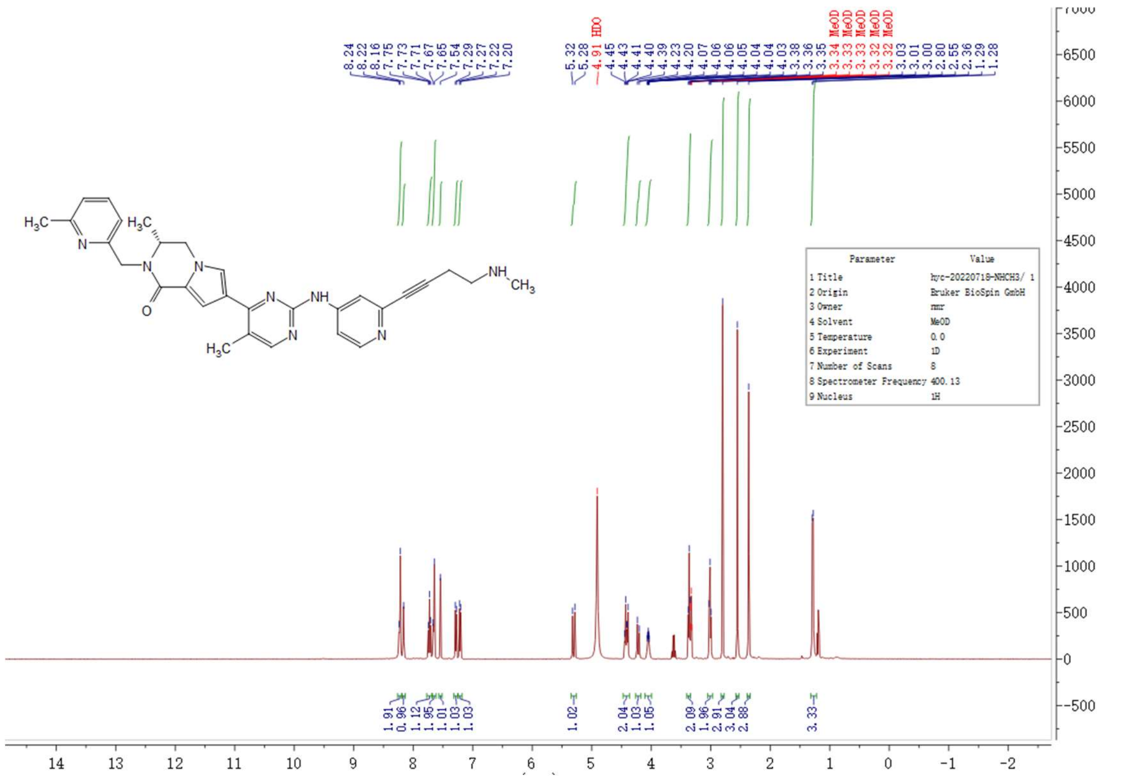

^{1}H NMR of **B-J**

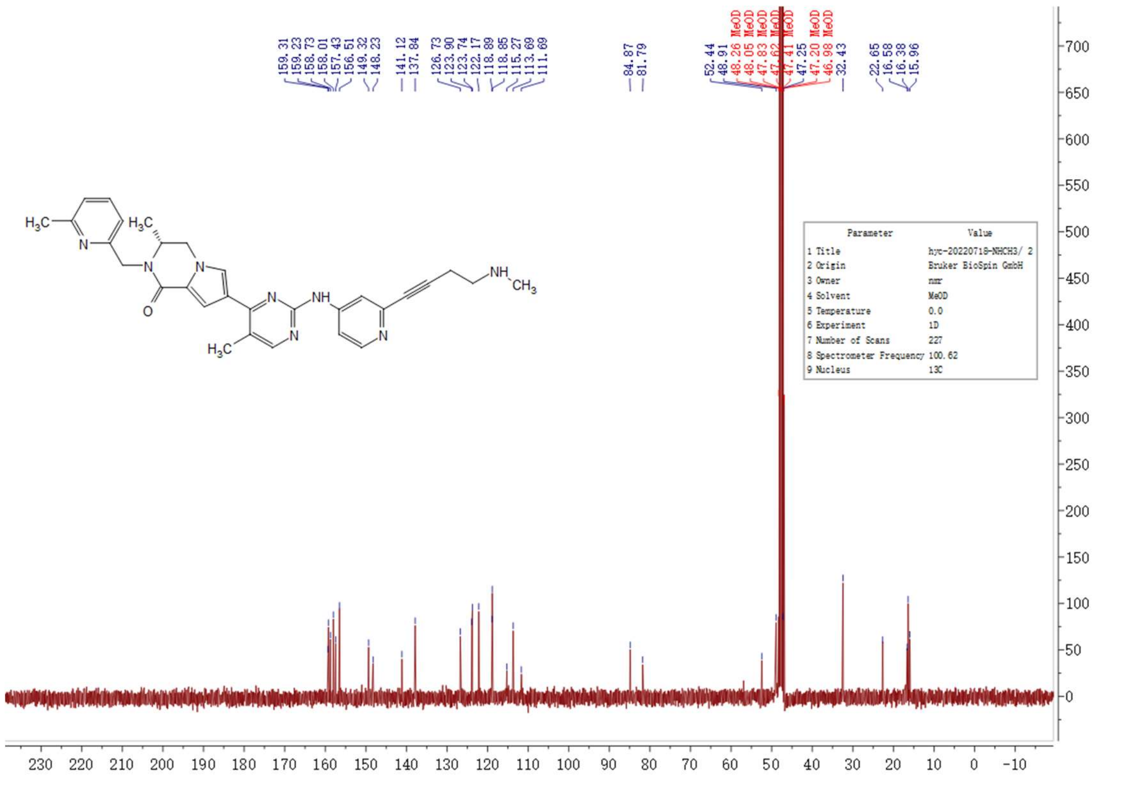

^{13}C NMR of **B-J**

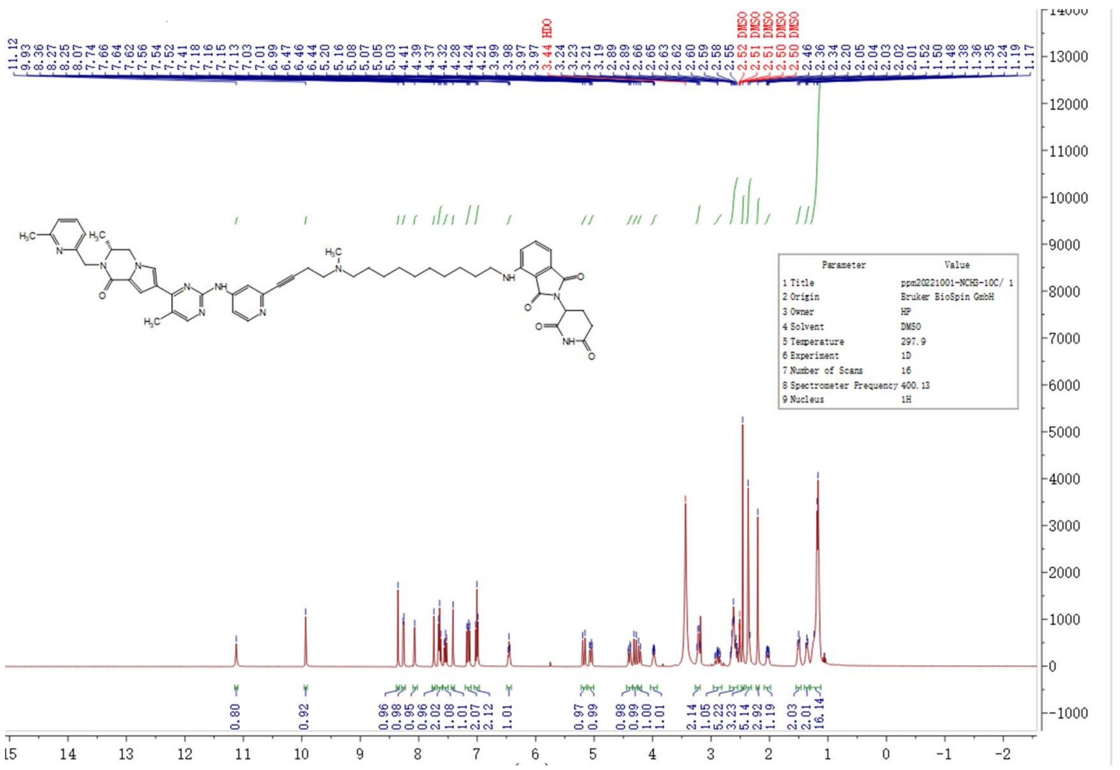

^{1}H NMR of **B1-10J**

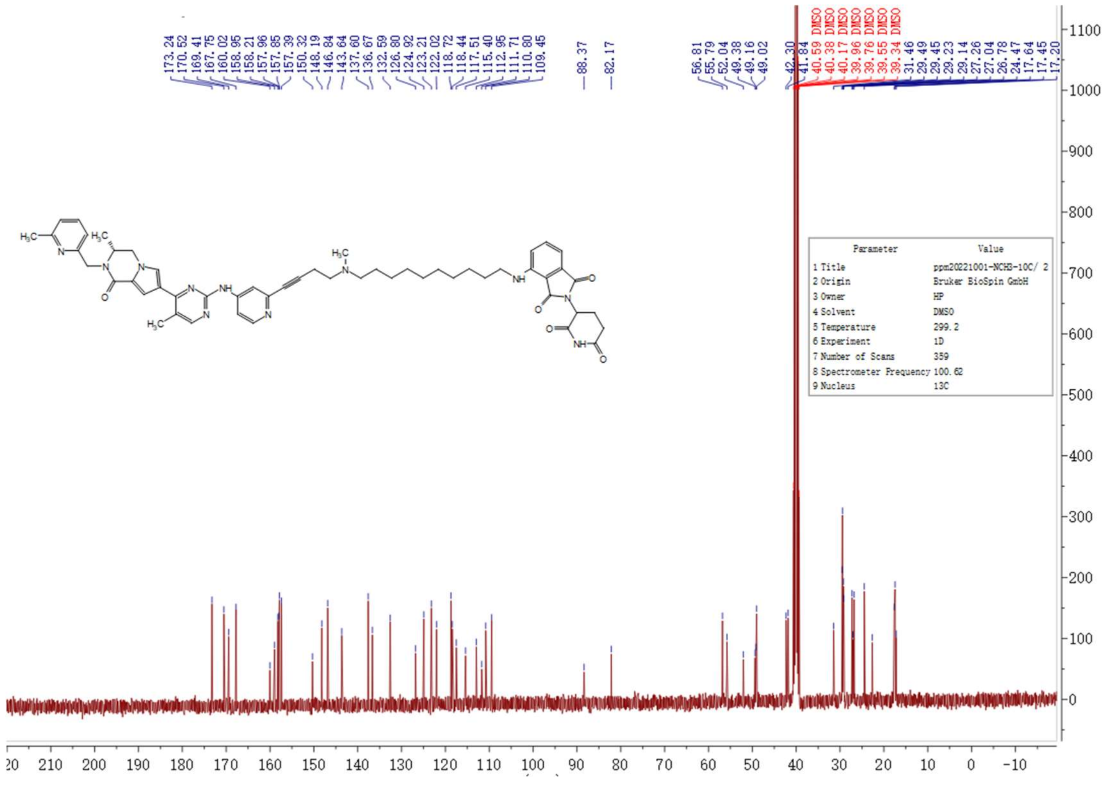

^{13}C NMR of **B1-10J**

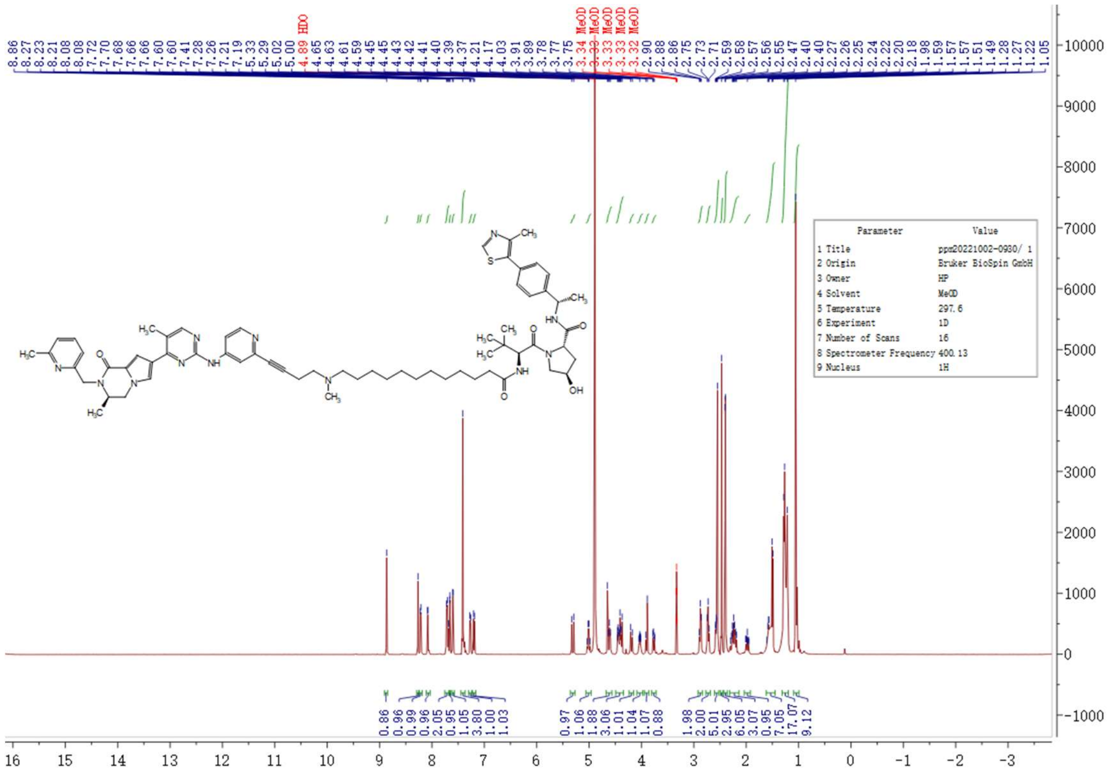

^{1}H NMR of **B2-12J**

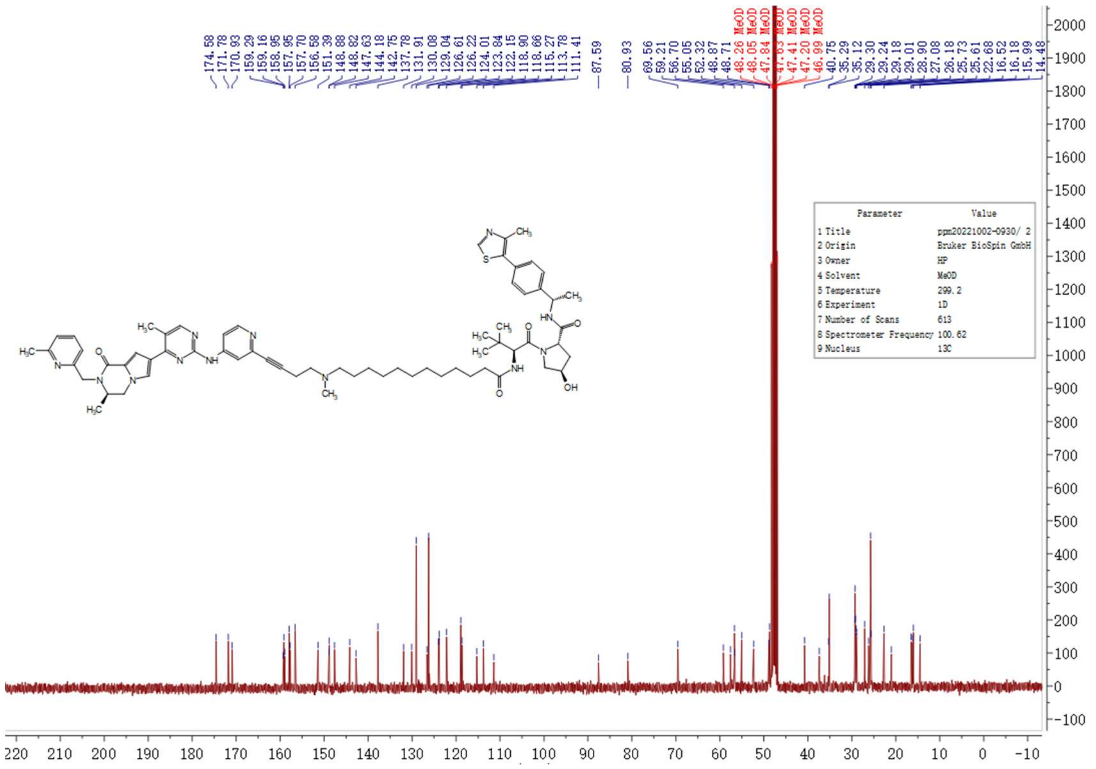

^{13}C NMR of **B2-12J**

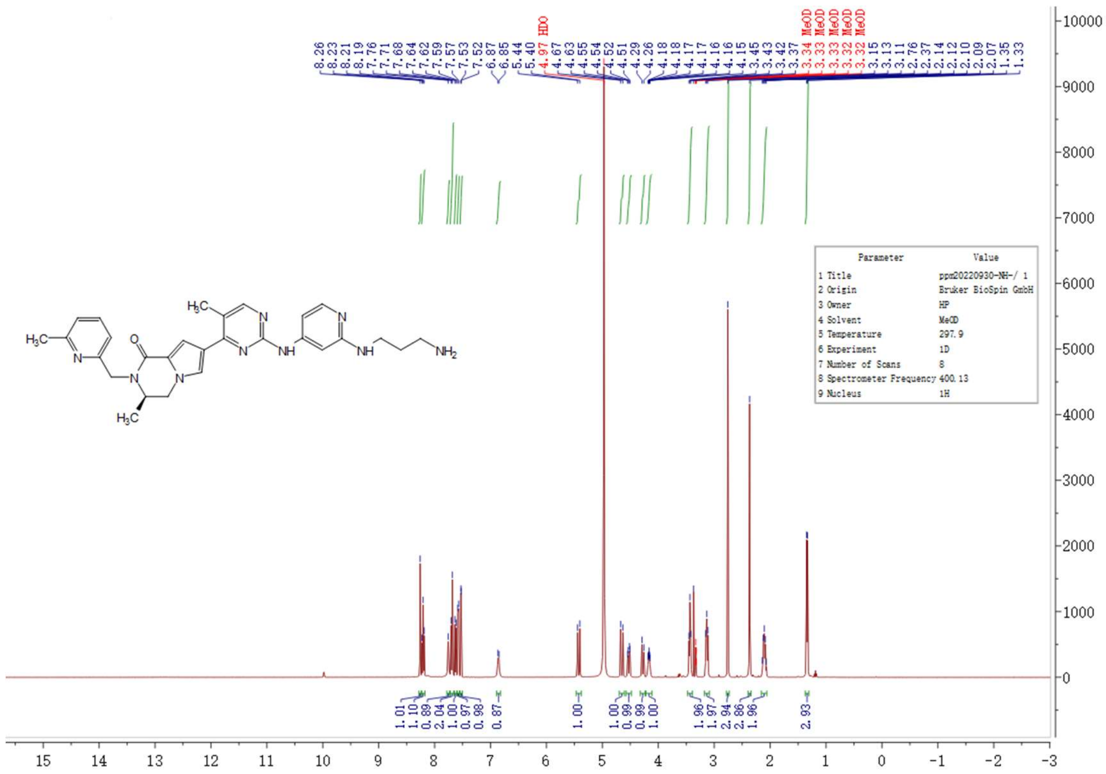

^{1}H NMR of **B-N**

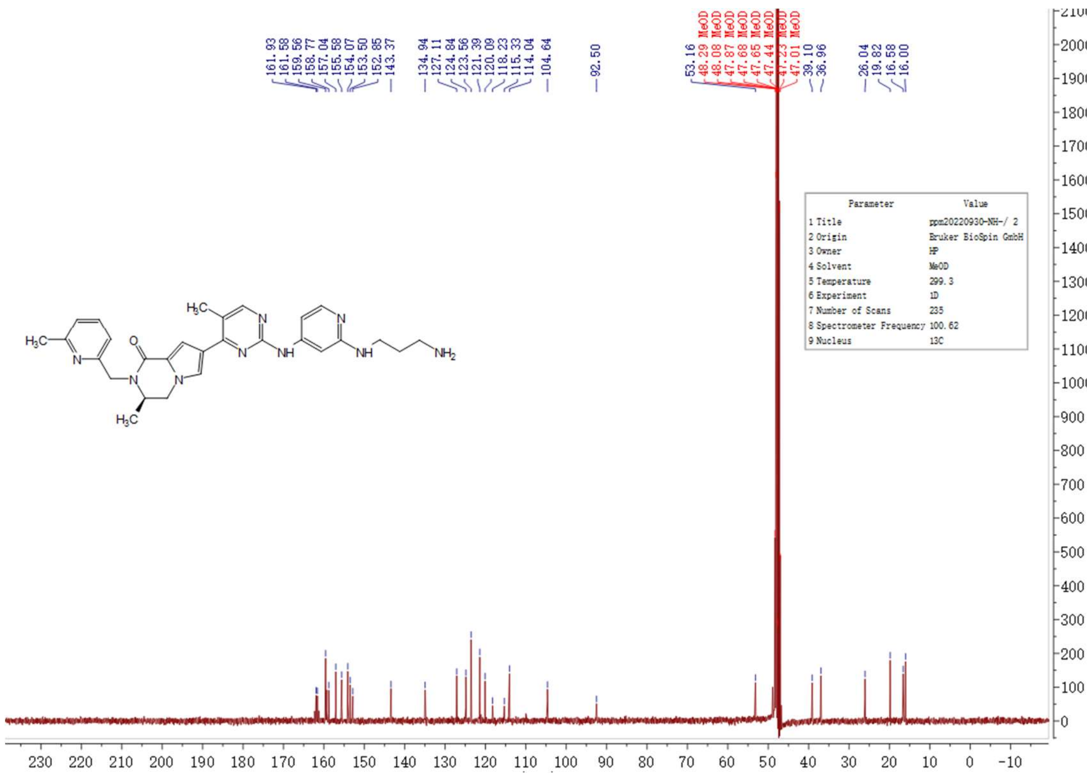

^{13}C NMR of **B-N**

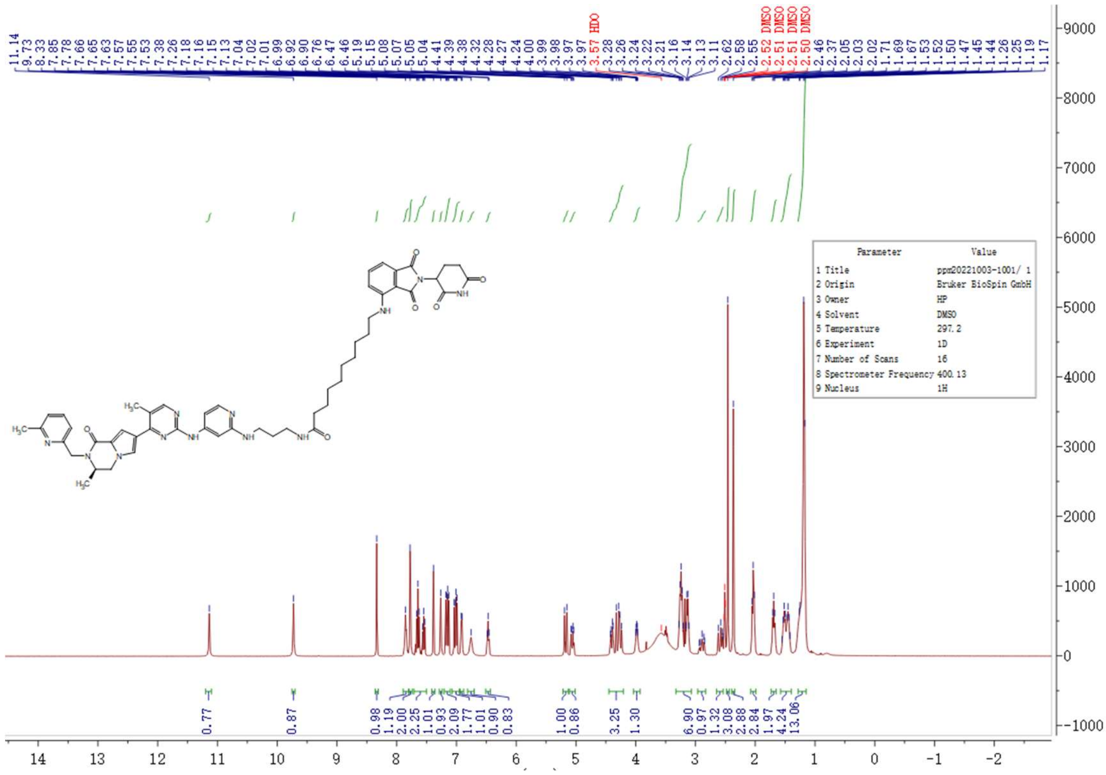

^{1}H NMR of **B1-10N**

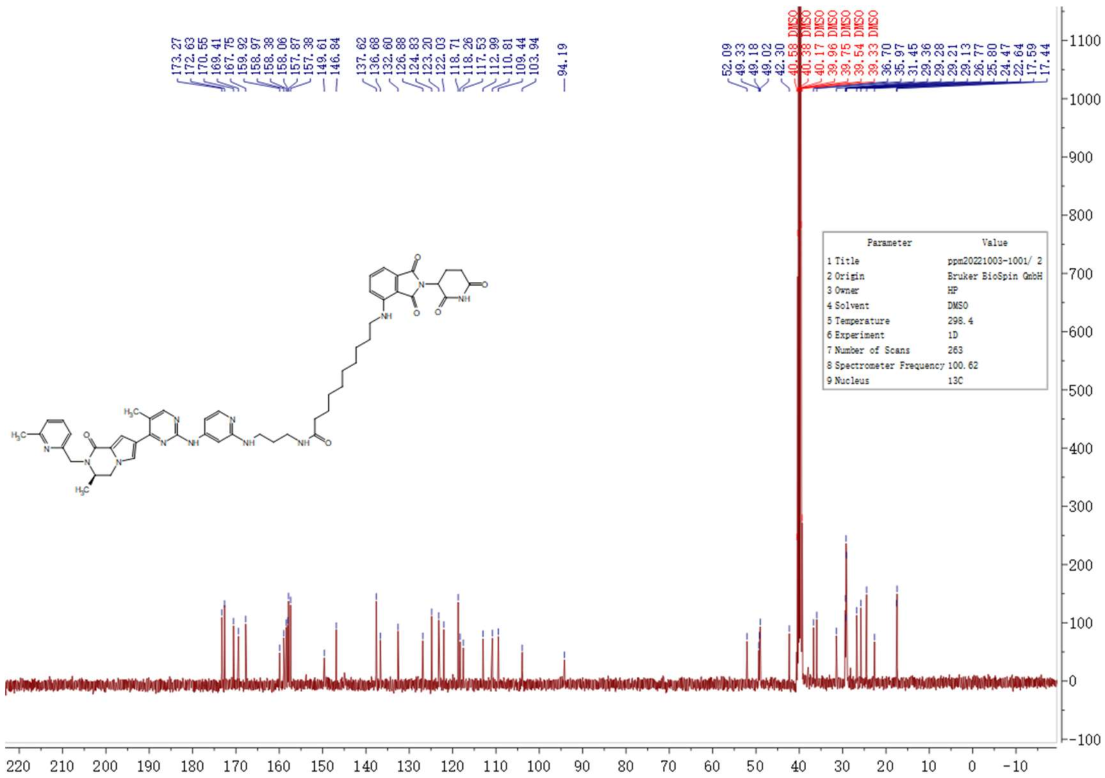

^{13}C NMR of **B1-10N**

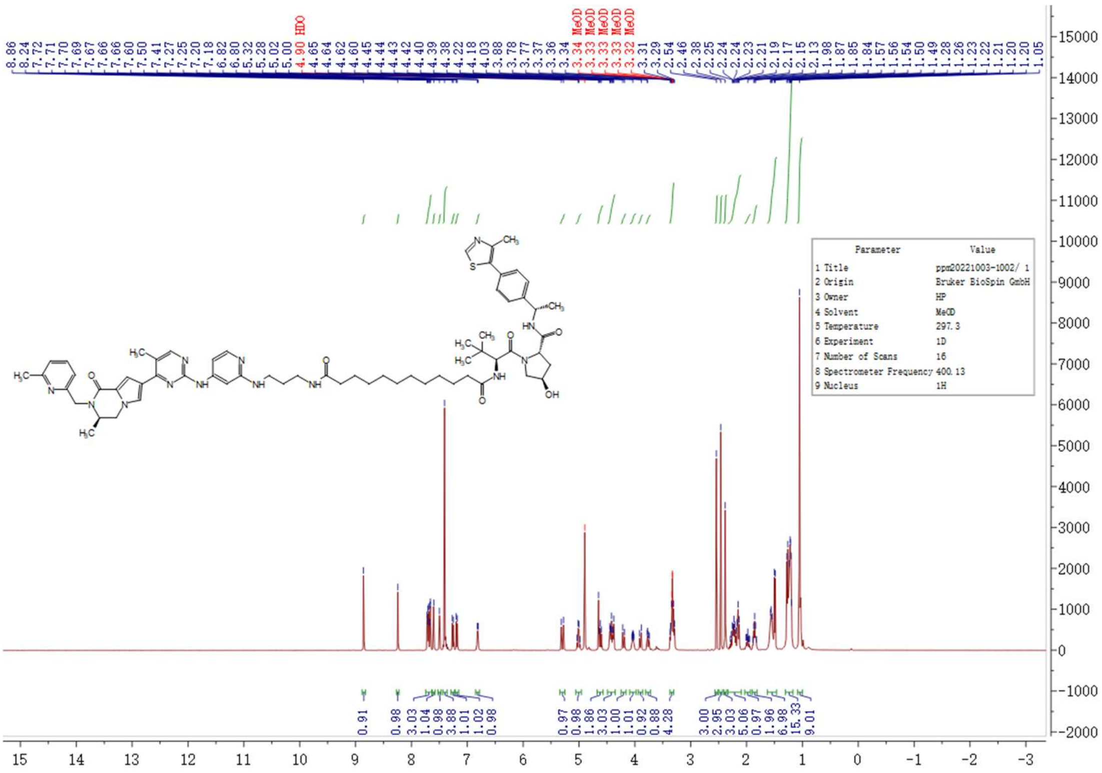

^{1}H NMR of **B2-12N**

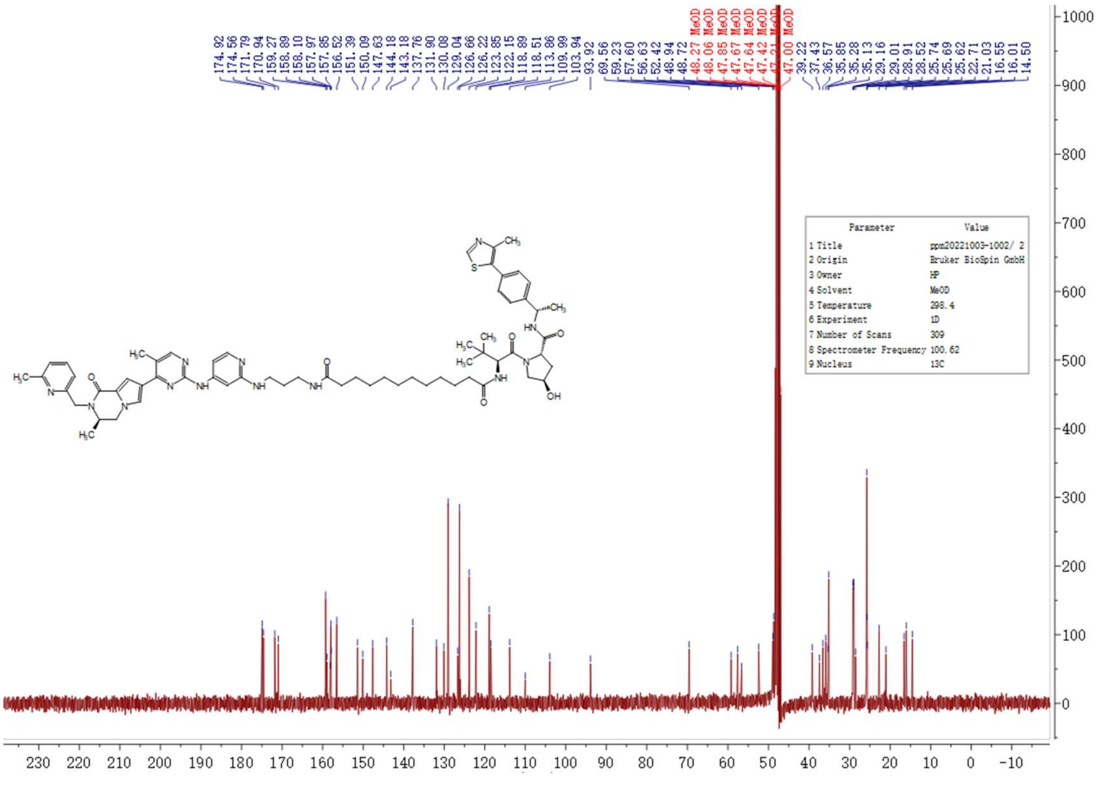

^{13}C NMR of **B2-12N**

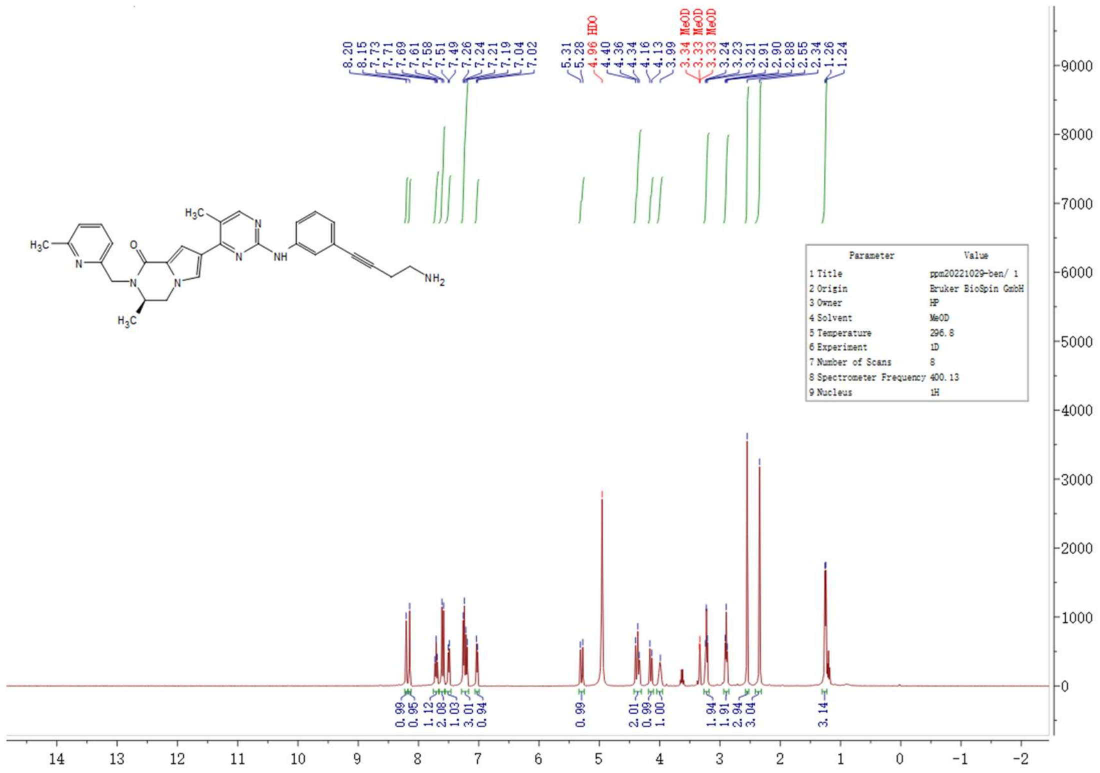

^{1}H NMR of **B-B**

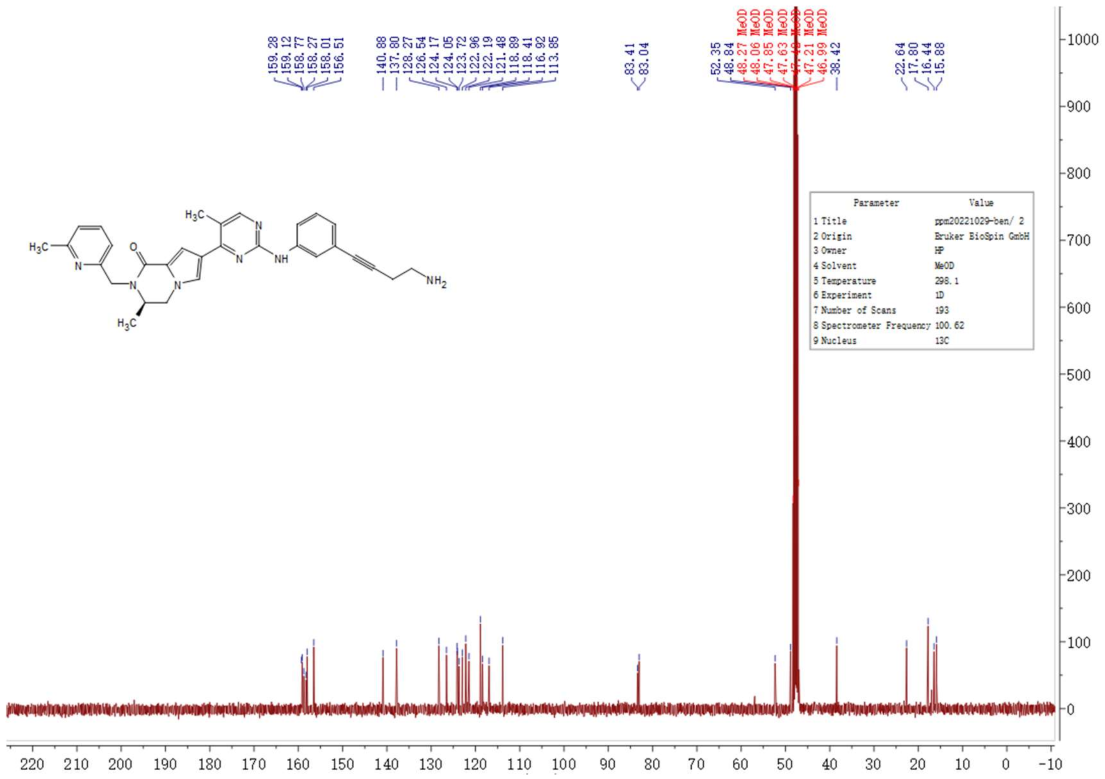

^{13}C NMR of **B-B**

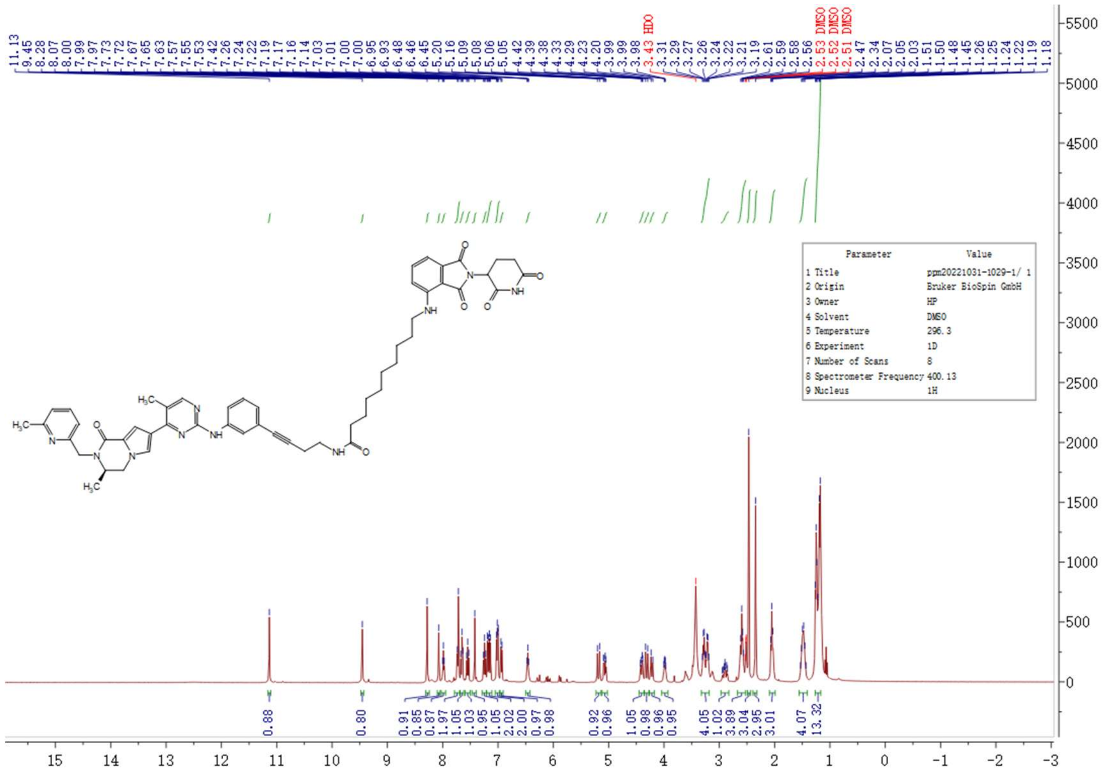

^{1}H NMR of **B1-10B**

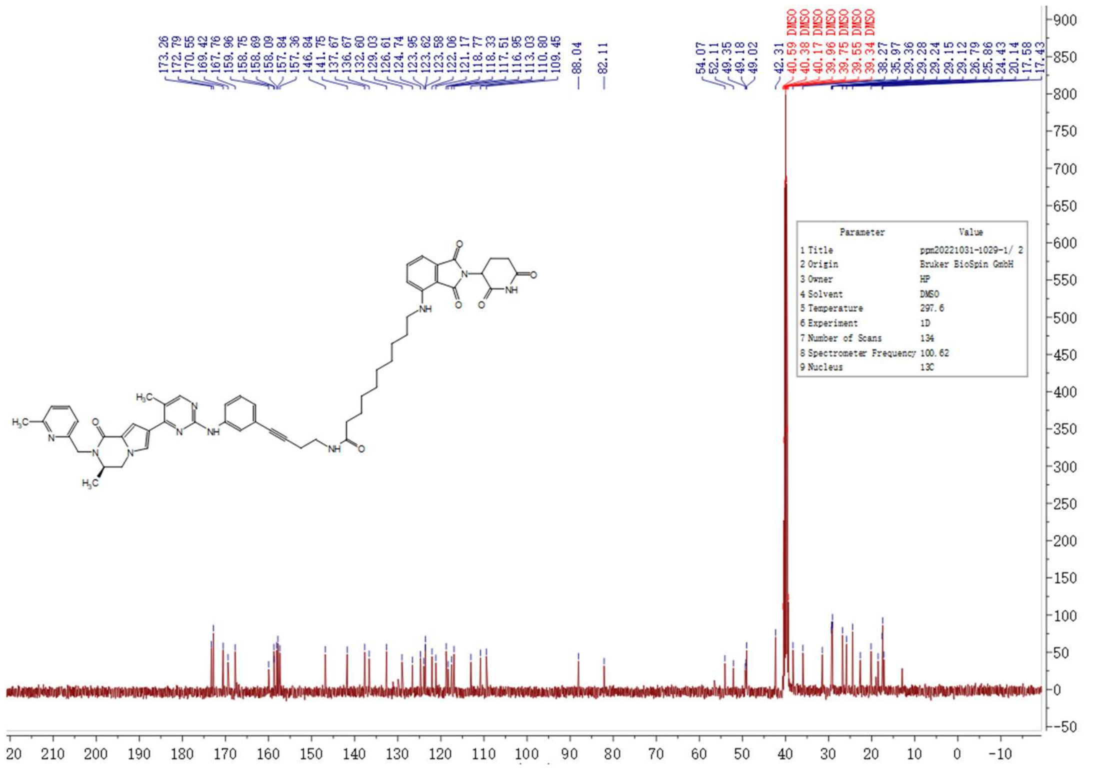

^{13}C NMR of **B1-10B**

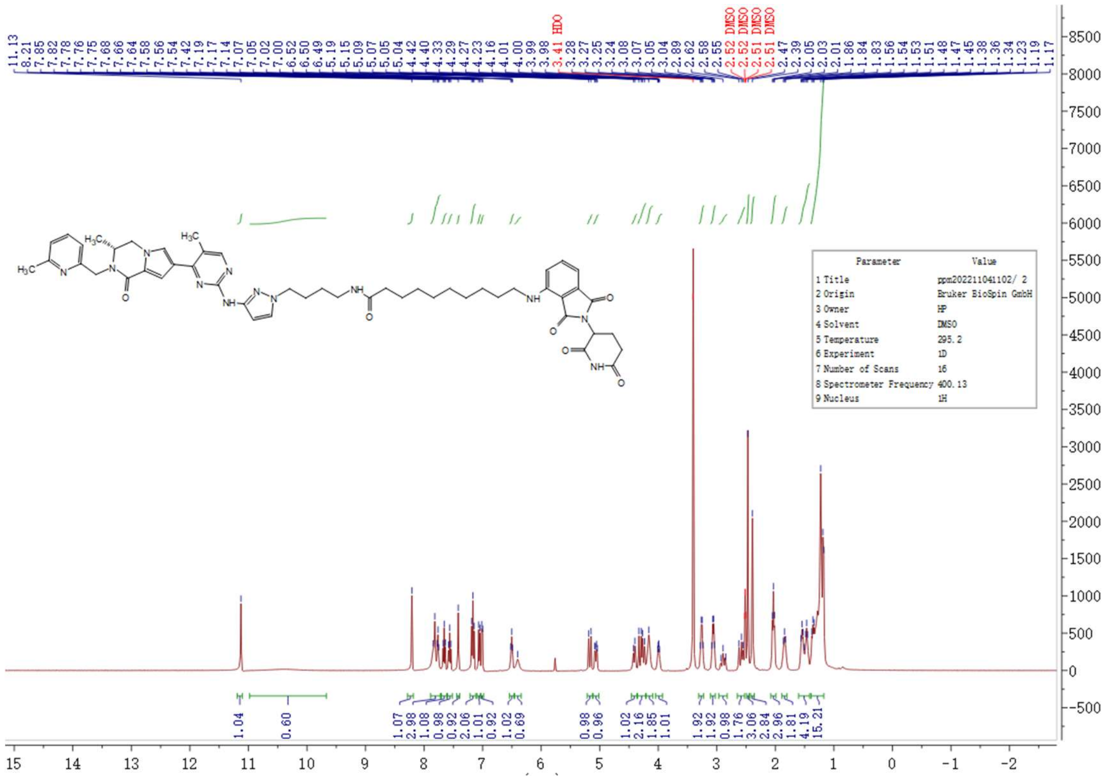

^{1}H NMR of **B-BZ**

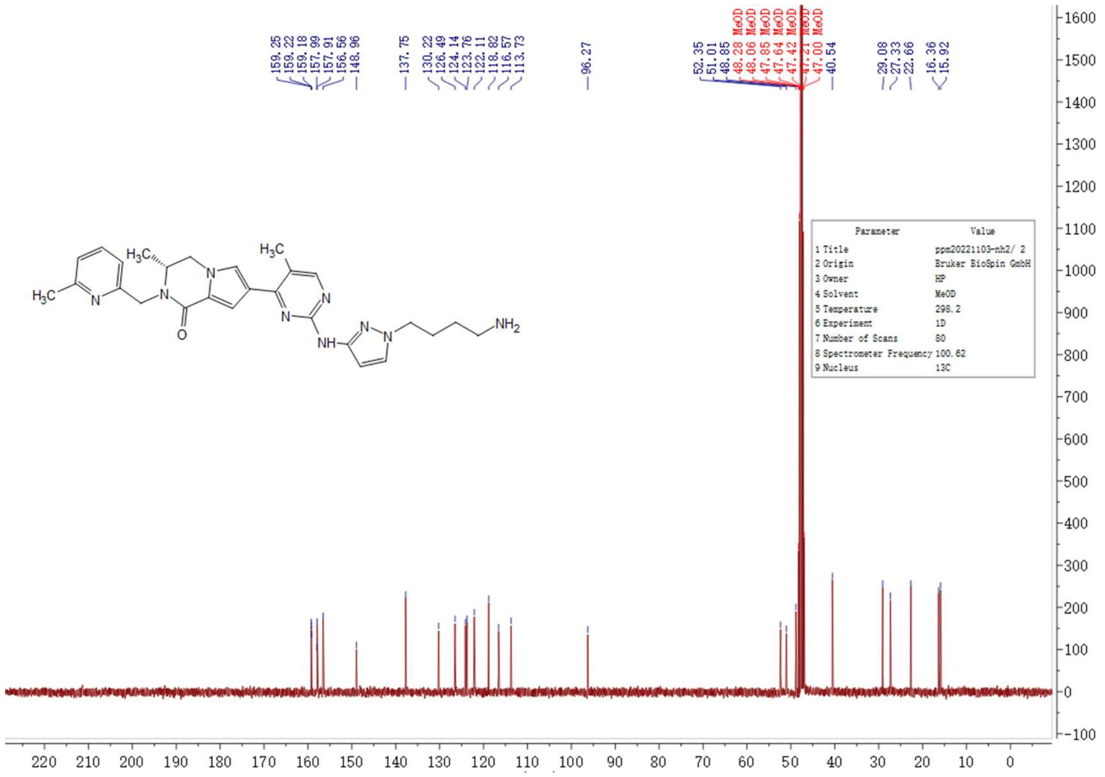

^{13}C NMR of **B-BZ**

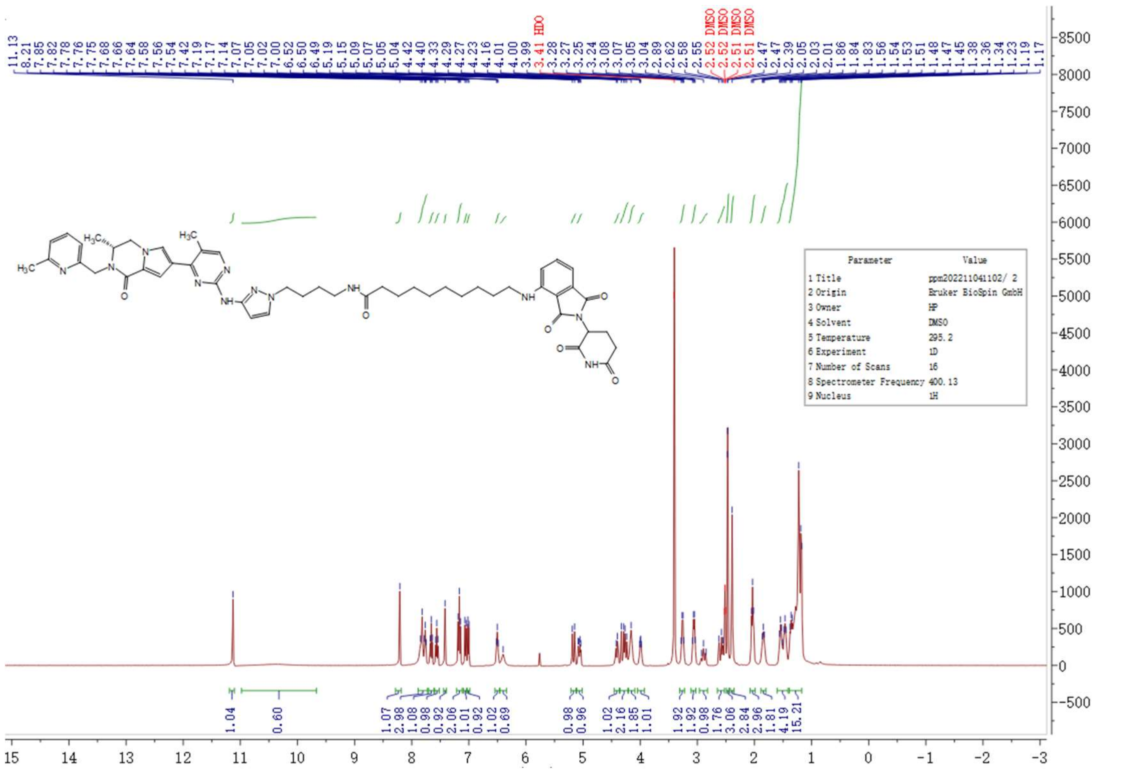

^{1}H NMR of **B1-10BZ**

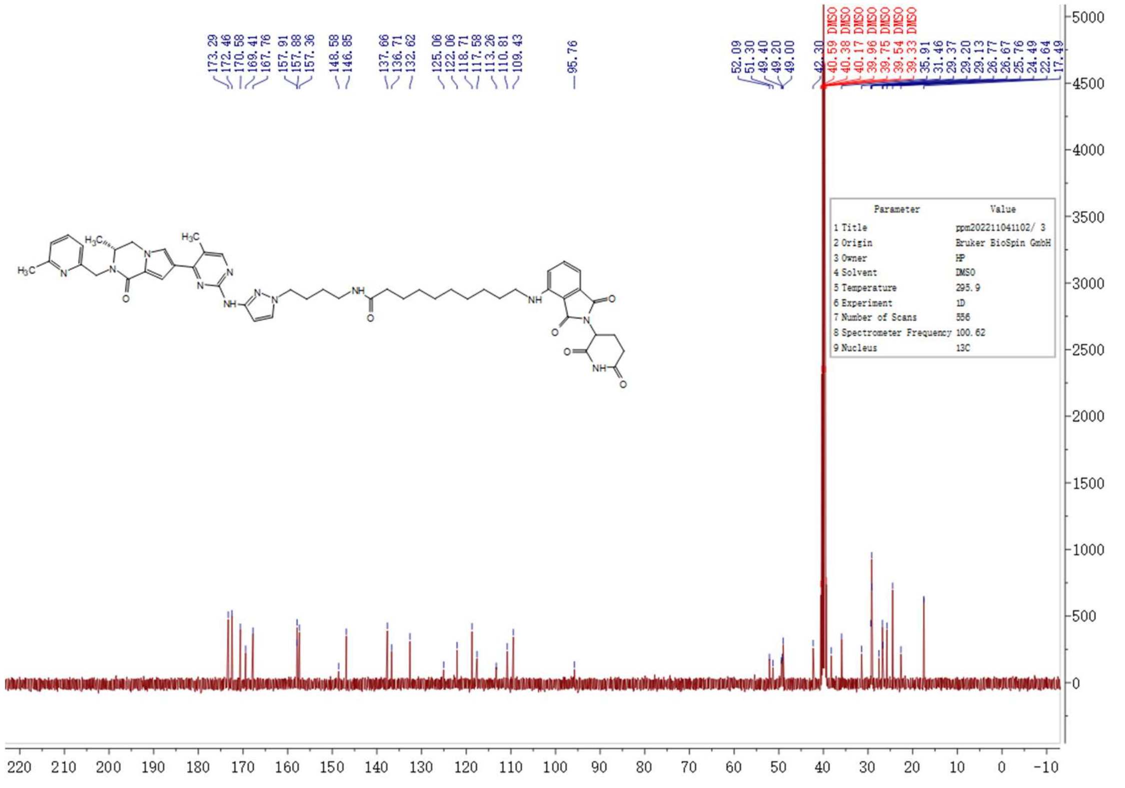

^{13}C NMR of **B1-10BZ**

6. HRMS spectrum

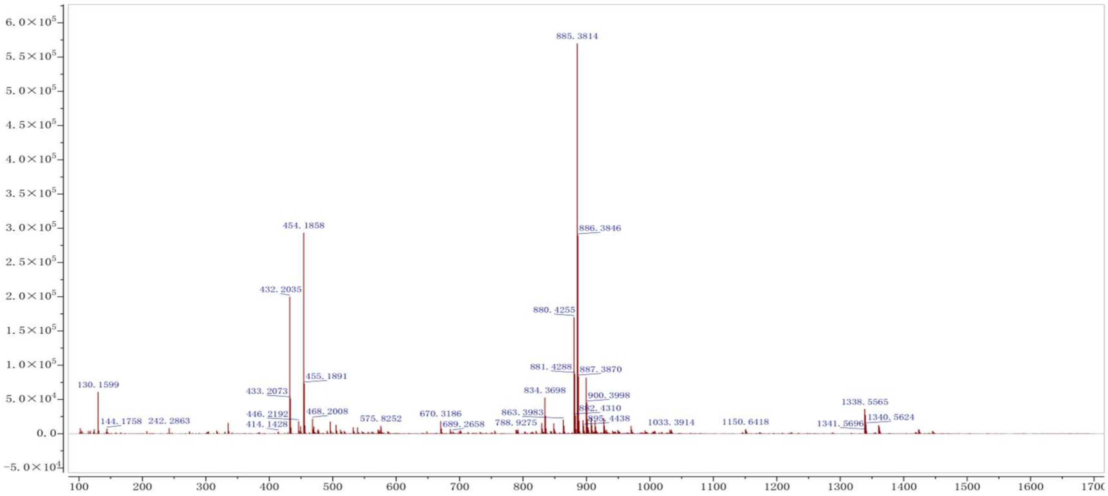

HRMS of (E)-7-(2,6-dimethoxy-4-(3-oxo-3-(6-oxo-3,6-dihydropyridin-1(2H)-yl)prop-1-en-1-yl)phenoxy)heptanoic acid (**7**)

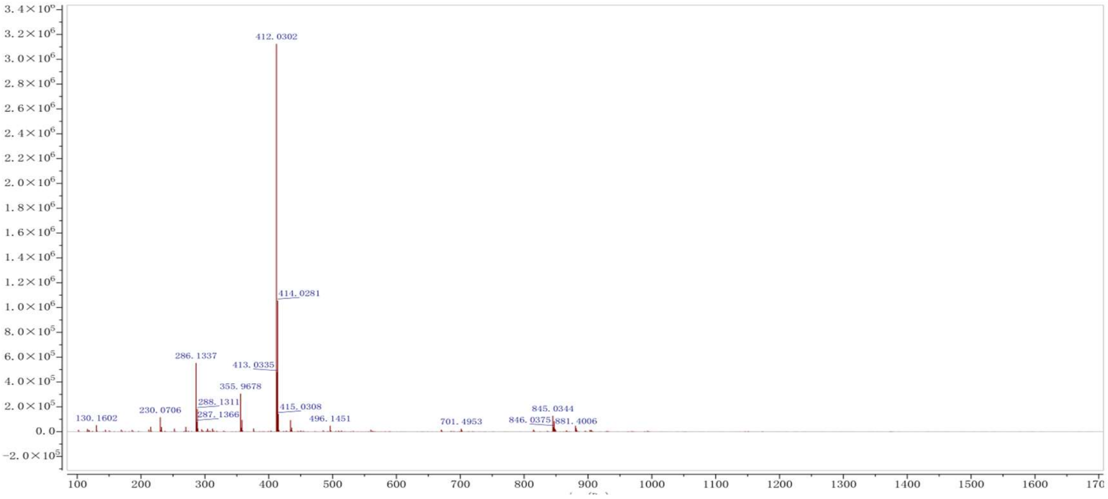

HRMS of tert-butyl (3-((5-chloro-4-iodopyridin-2-yl)amino)propyl)carbamate (**9**)

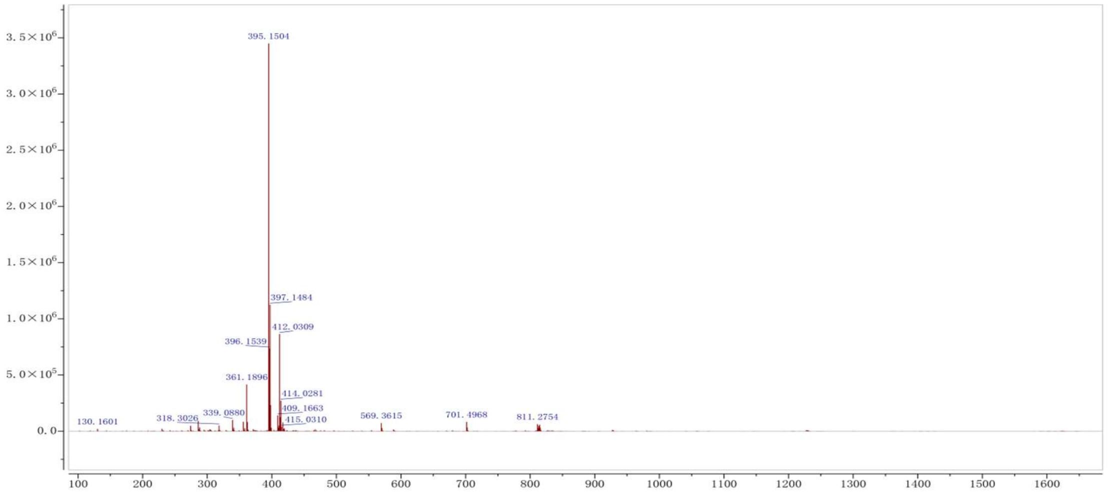

HRMS of 4-(2-((3-((tert-butoxycarbonyl)amino)propyl)amino)-5-chloropyridin-4-yl)-1H-pyrrole-2-carboxylic acid (**14**)

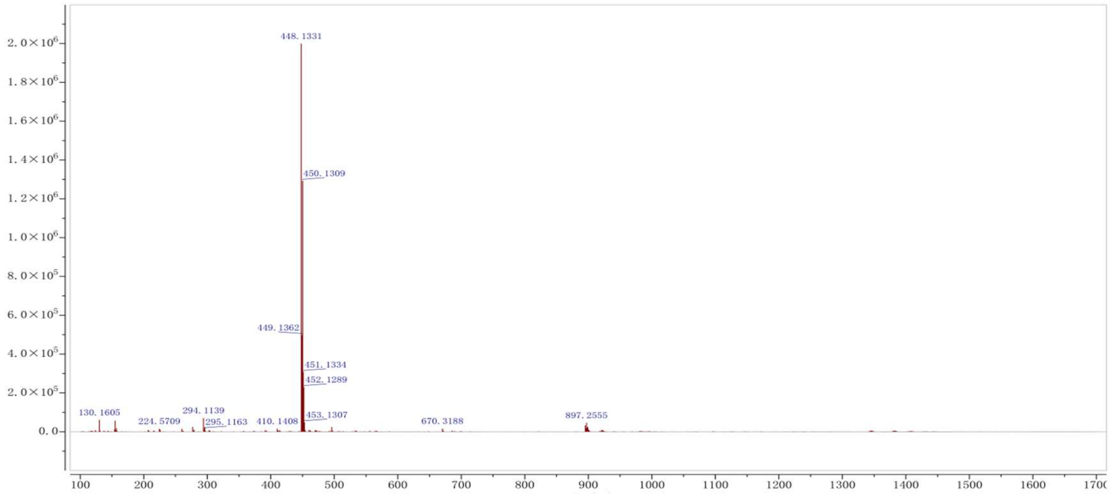

HRMS of **A**

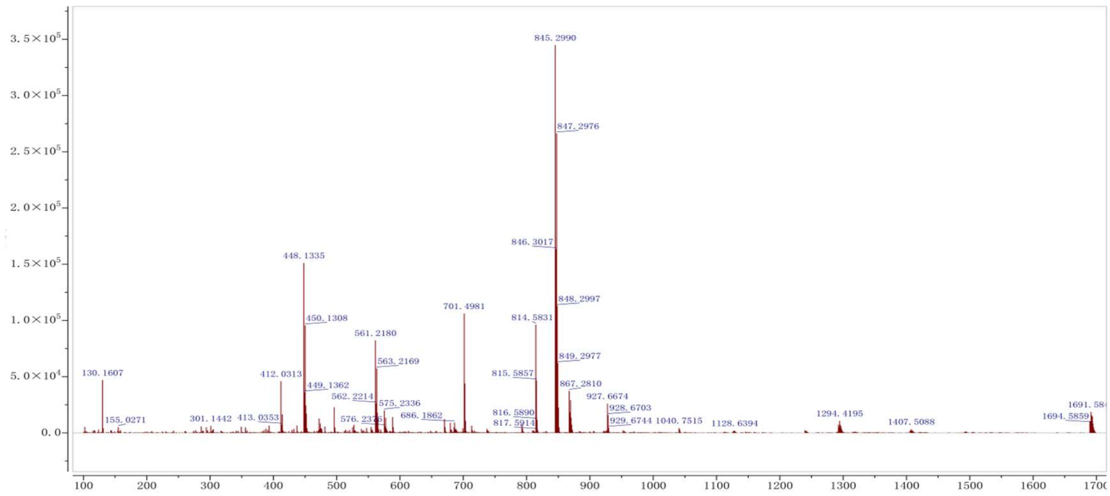

HRMS of **A1**

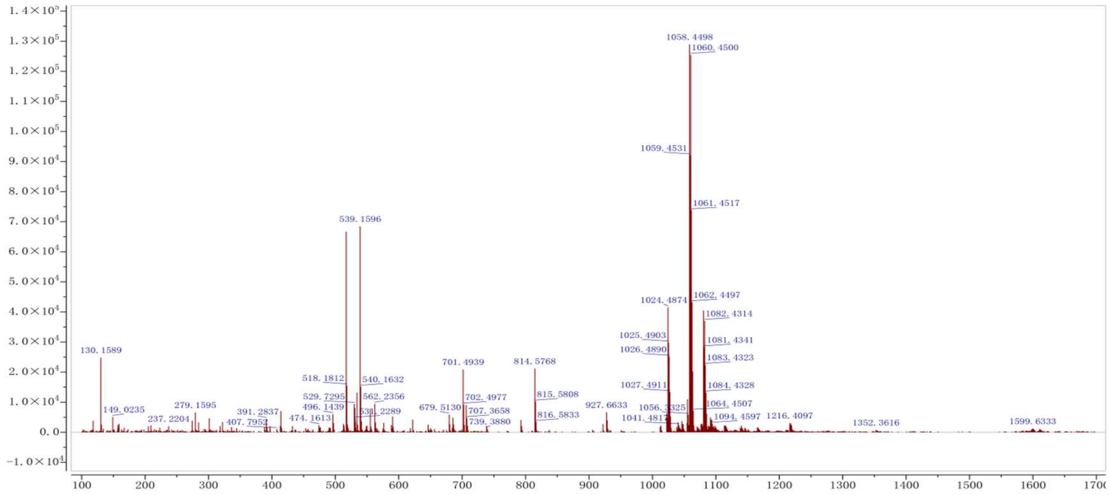

HRMS of **A2**

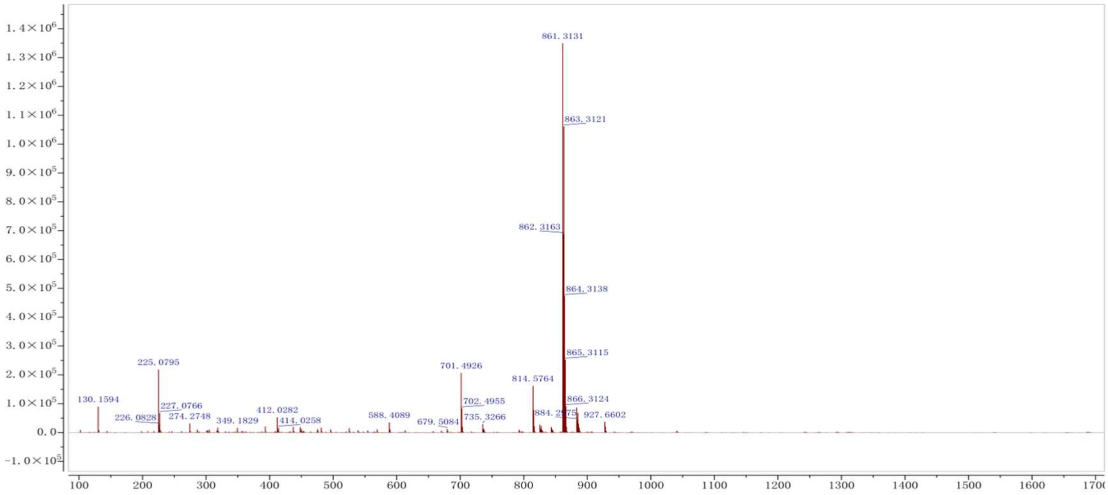

HRMS of **A3**

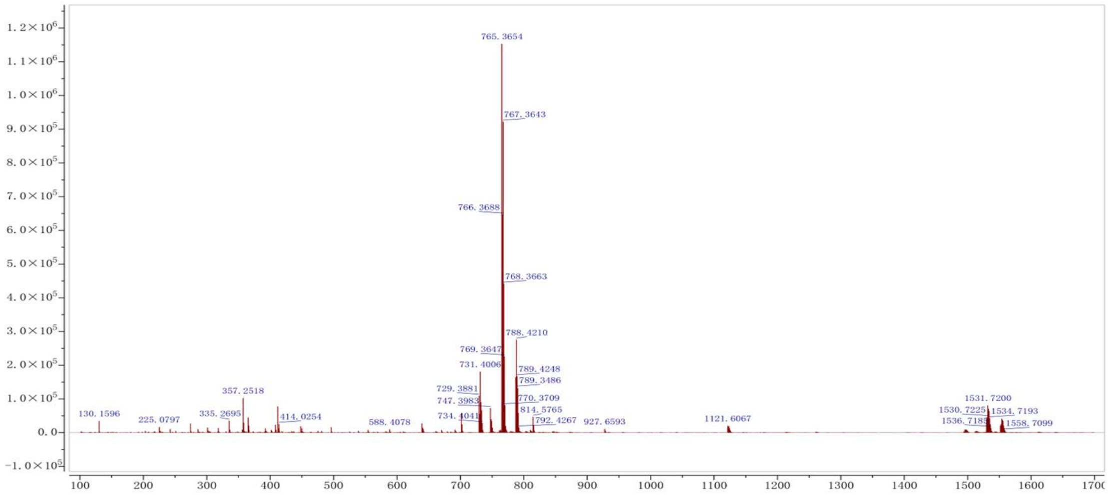

HRMS of **A4**

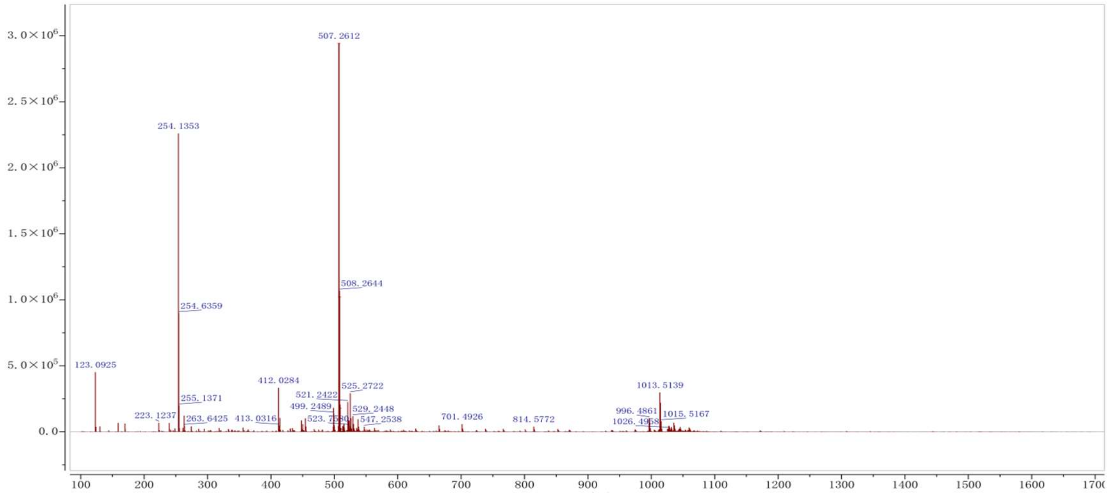

HRMS of **B**

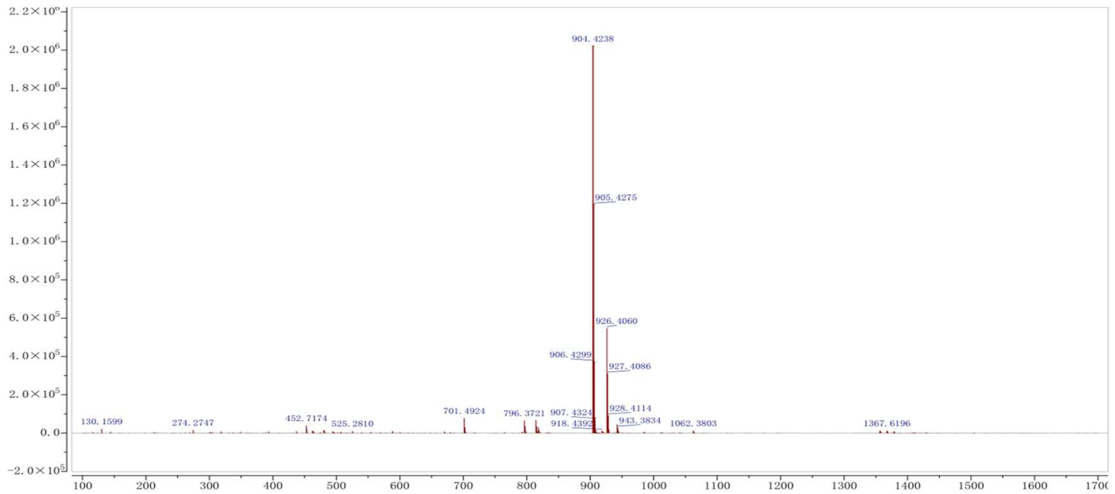

HRMS of **B1-8**

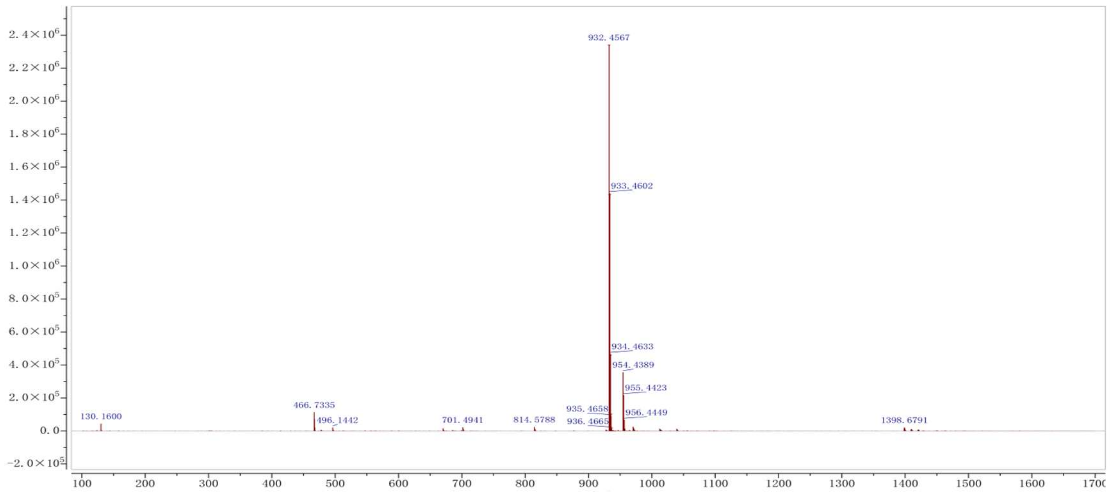

HRMS of **B2-10**

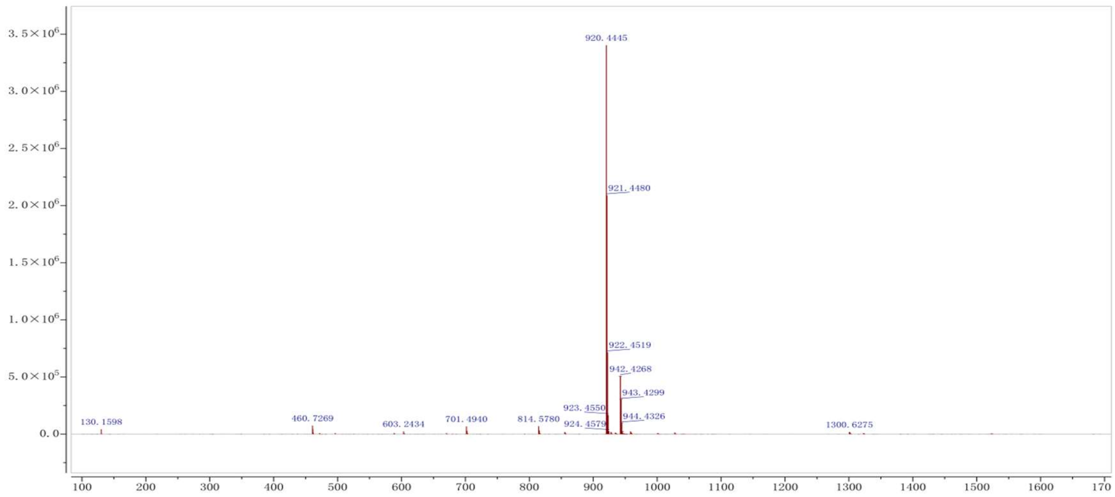

HRMS of **B3**

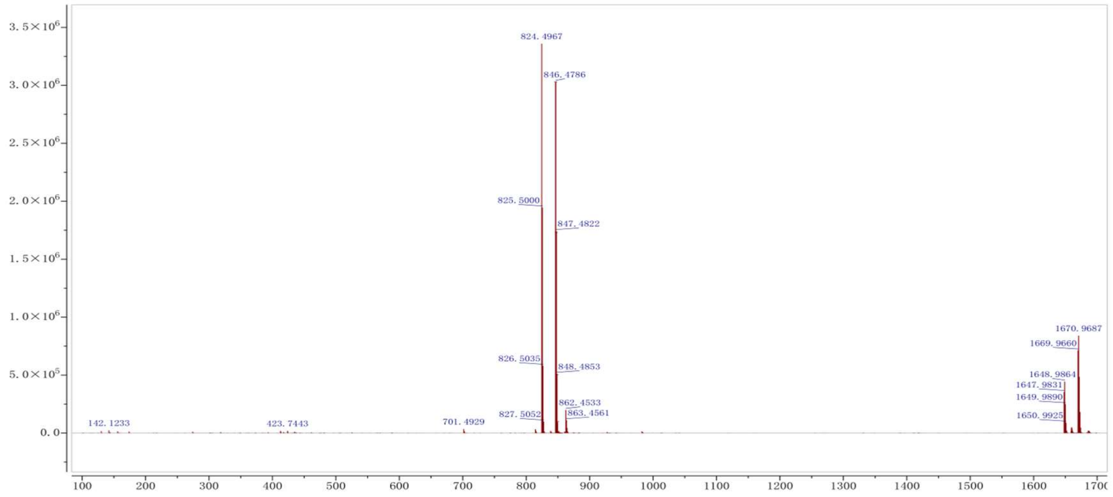

HRMS of **B4**

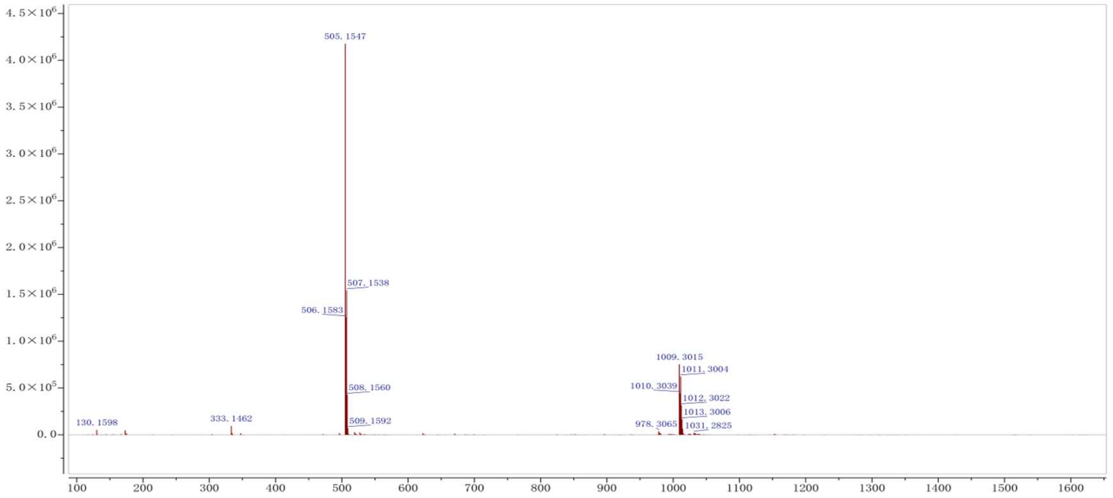

HRMS of **C**

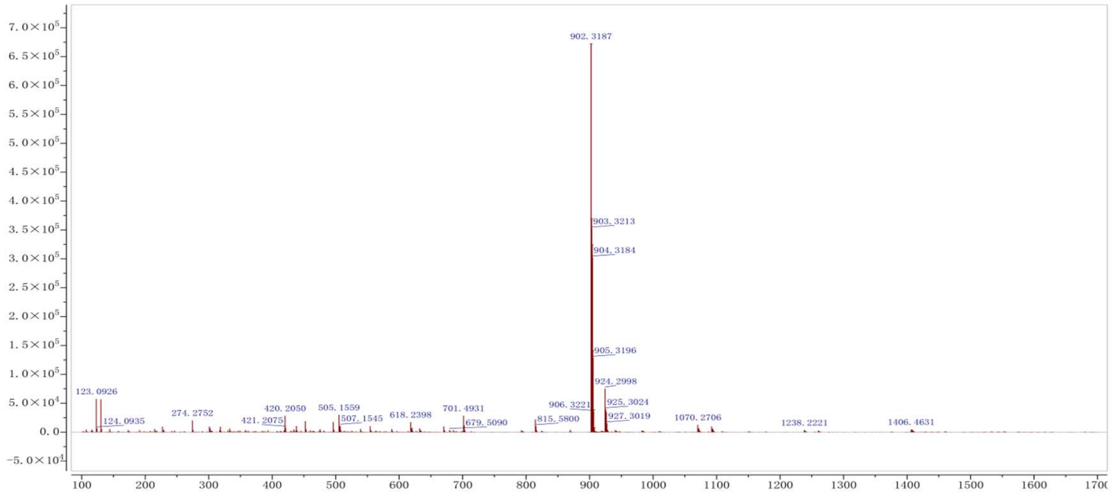

HRMS of **C1**

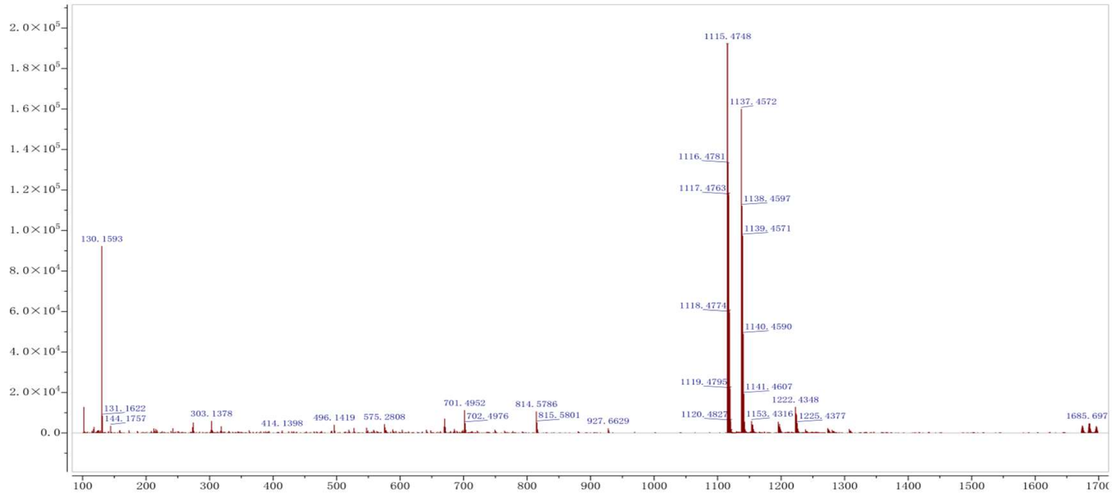

HRMS of **C2**

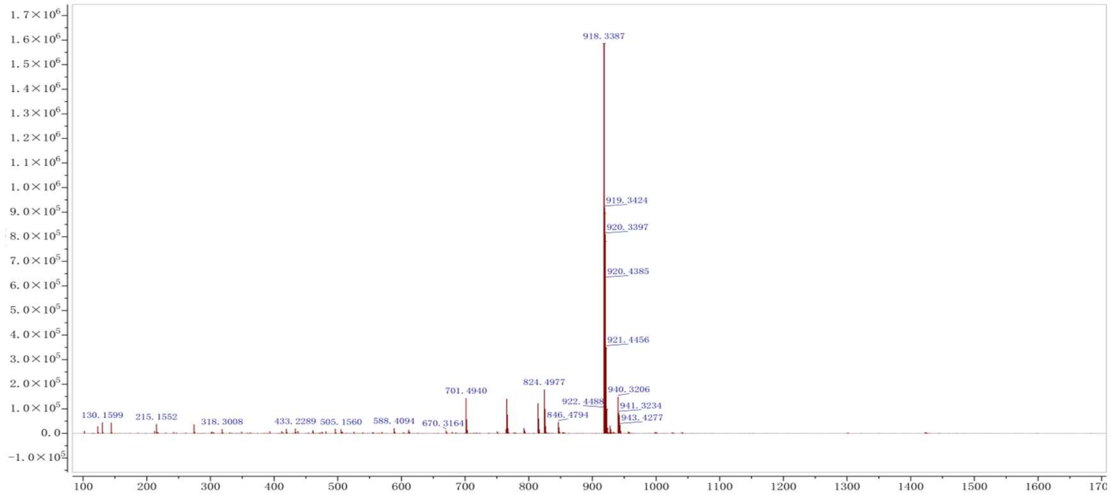

HRMS of **C3**

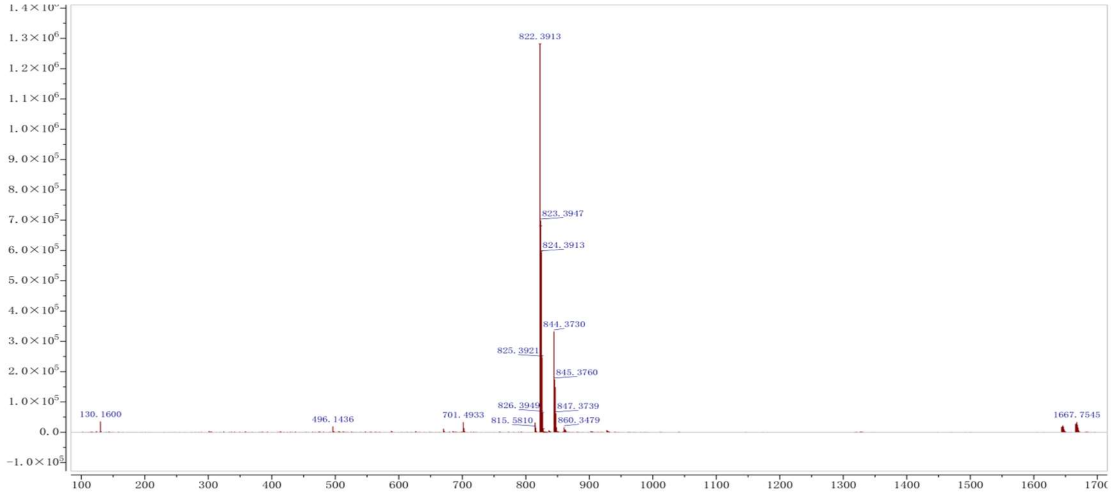

HRMS of **C4**

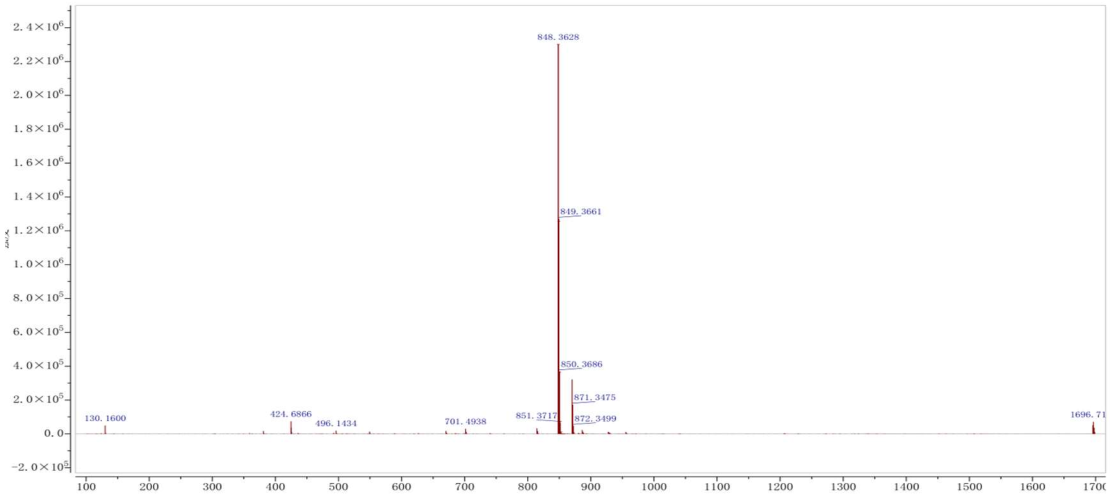

HRMS of **B1-4**

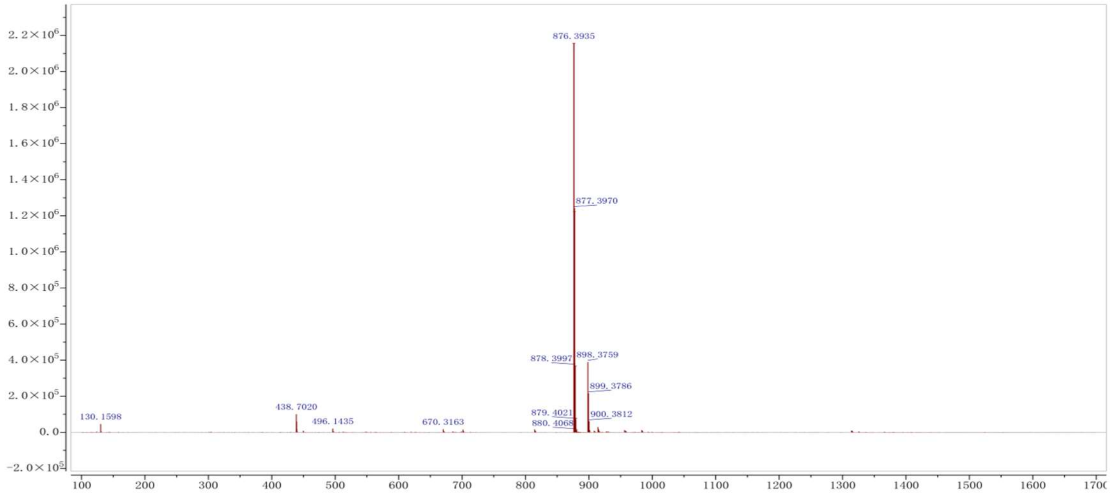

HRMS of **B1-6**

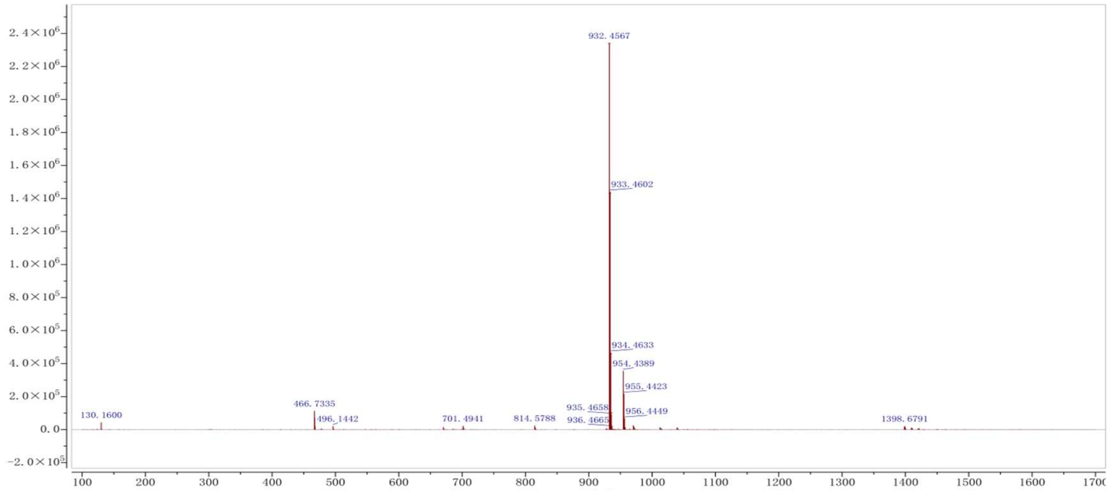

HRMS of **B1-10**

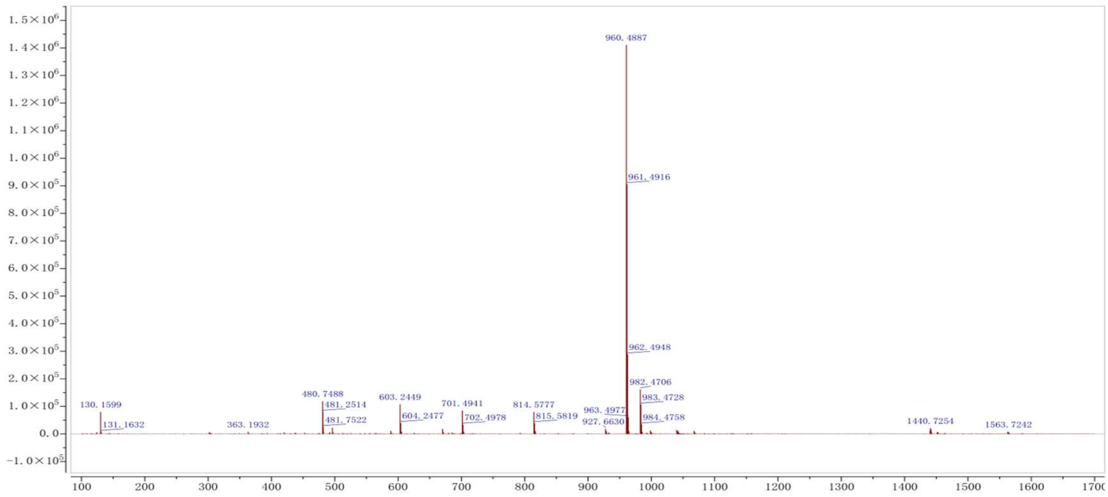

HRMS of **B1-12**

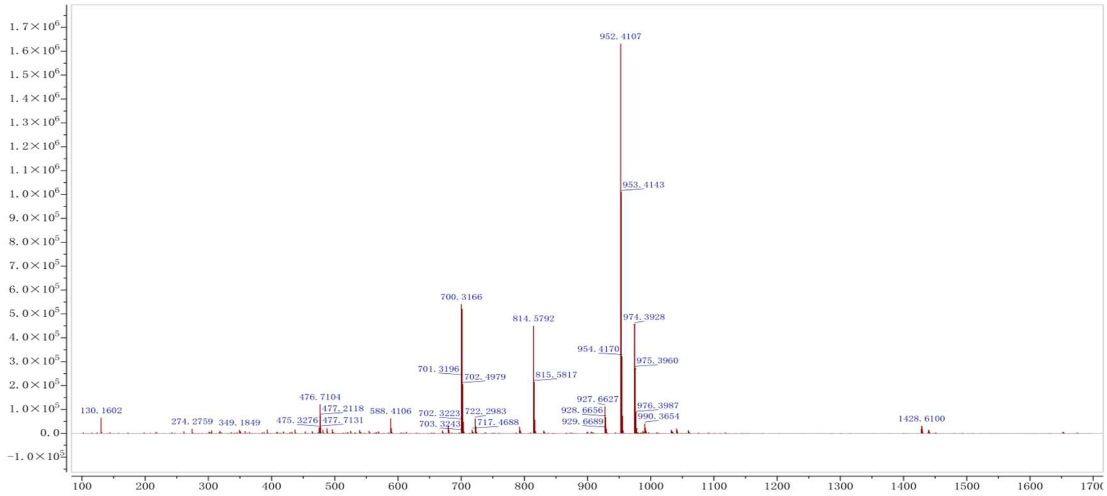

HRMS of **B1-3P**

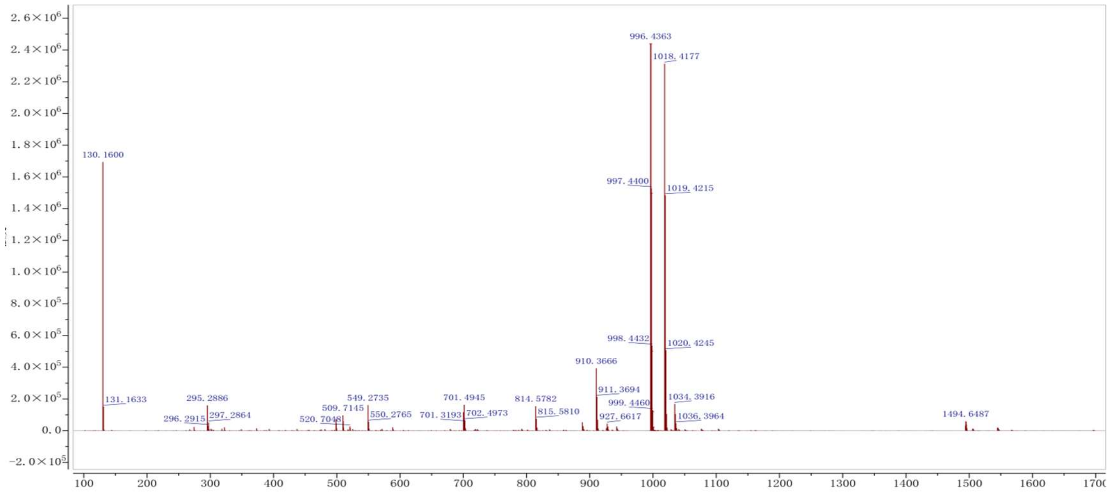

HRMS of **B1-4P**

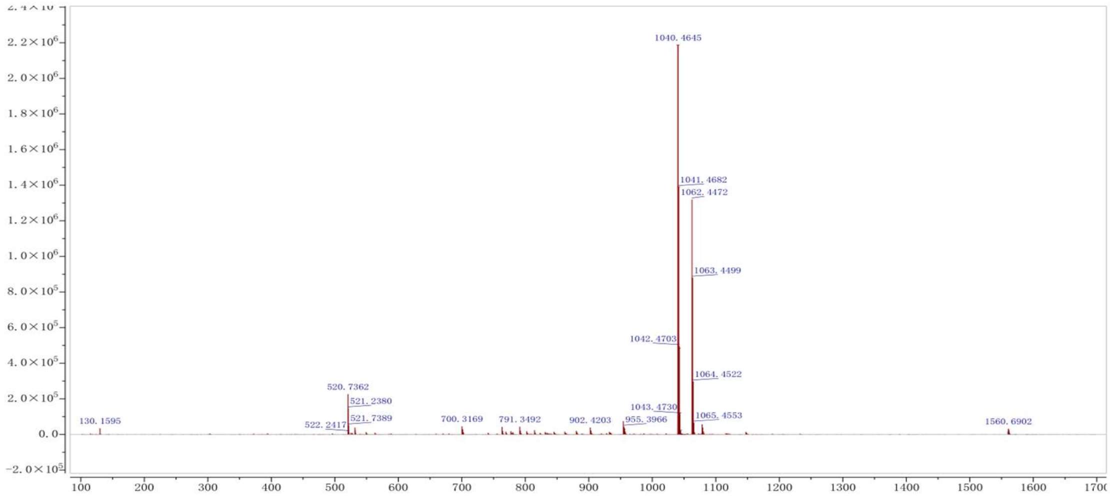

HRMS of **B1-5P**

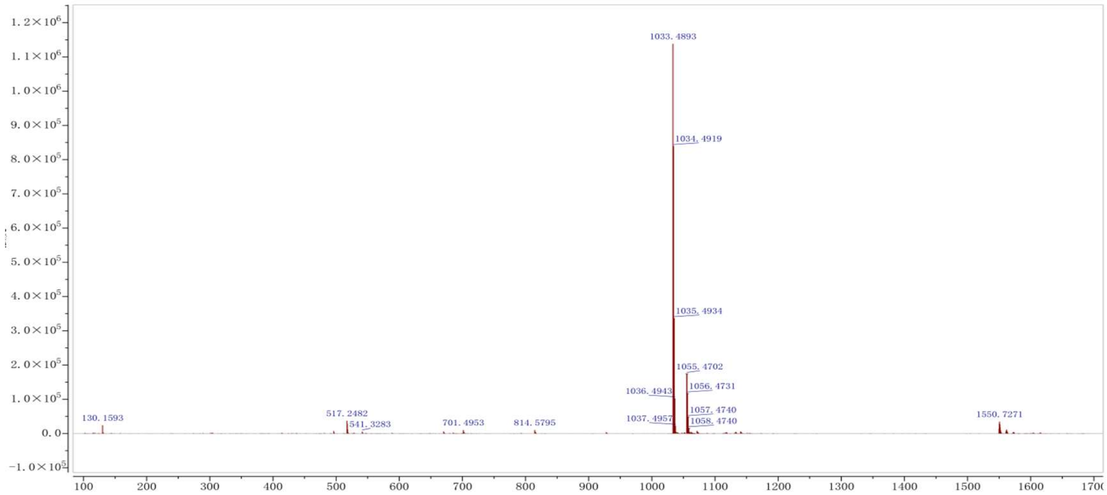

HRMS of **B2-4**

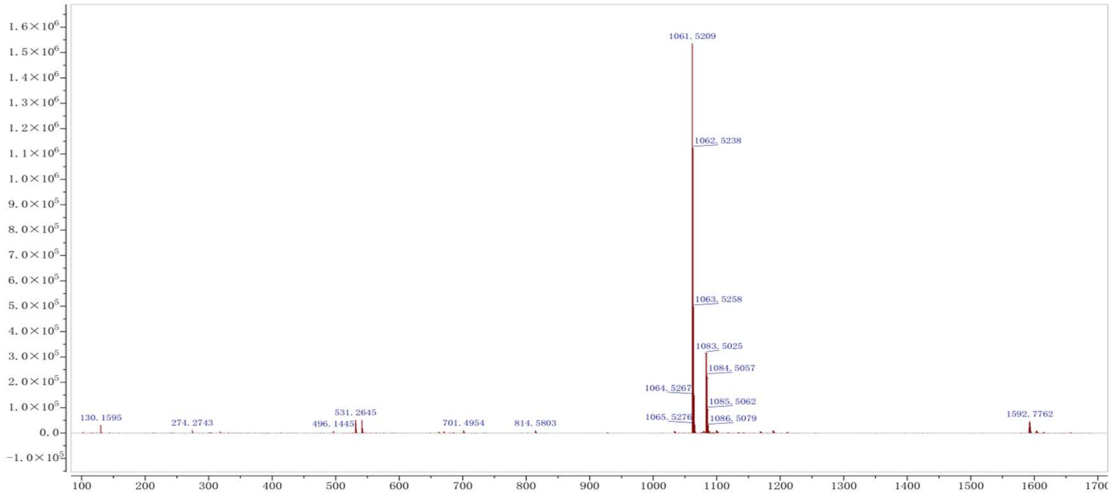

HRMS of **B2-6**

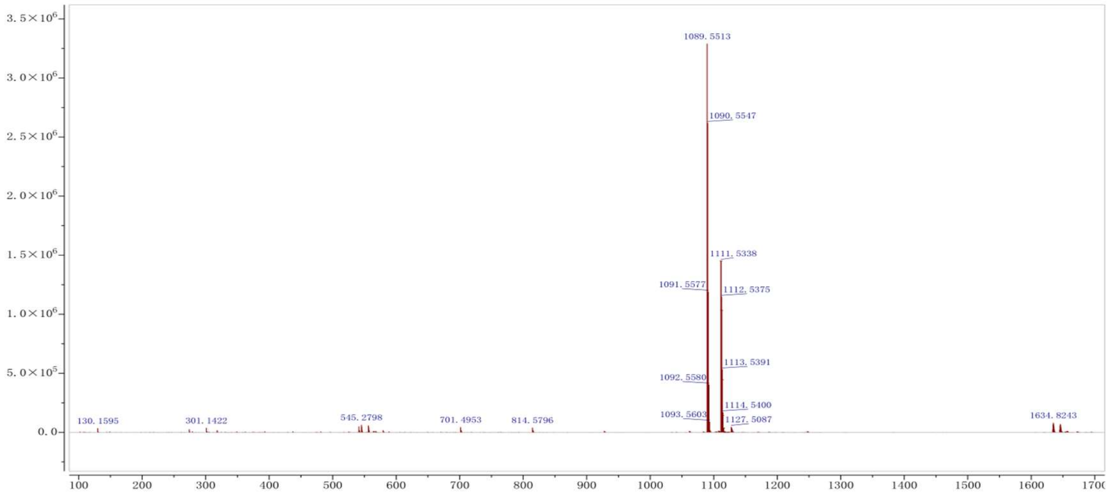

HRMS of **B2-8**

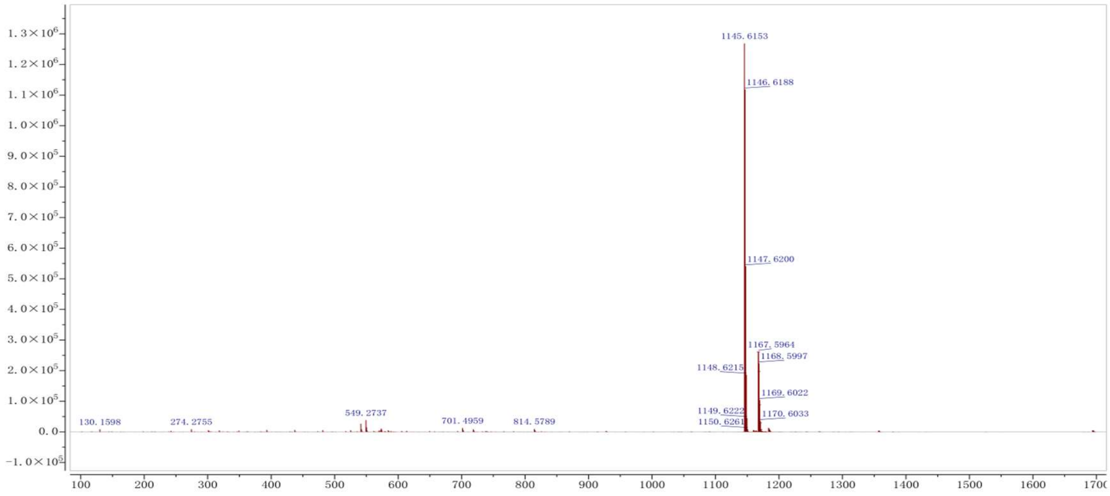

HRMS of **B2-12**

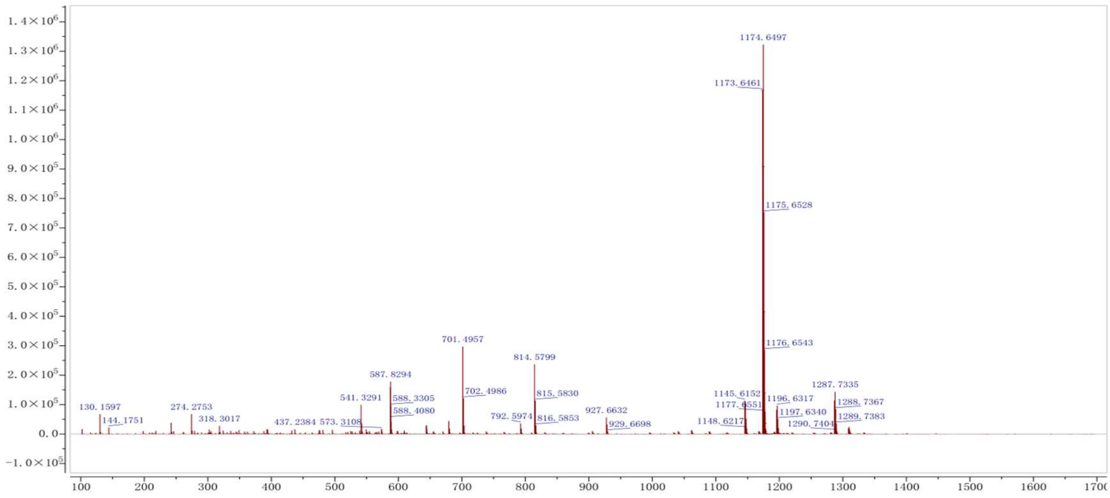

HRMS of **B2-14**

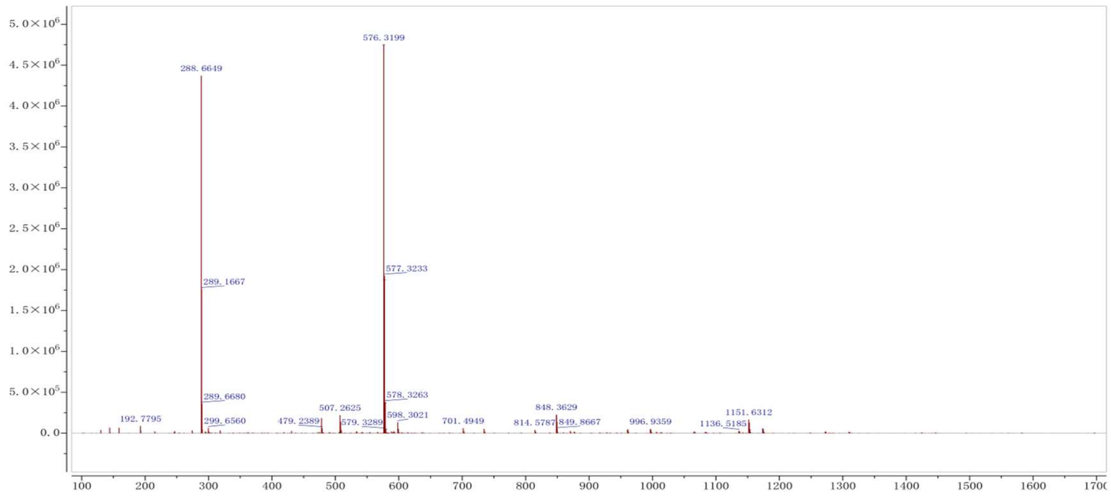

HRMS of **B-P**

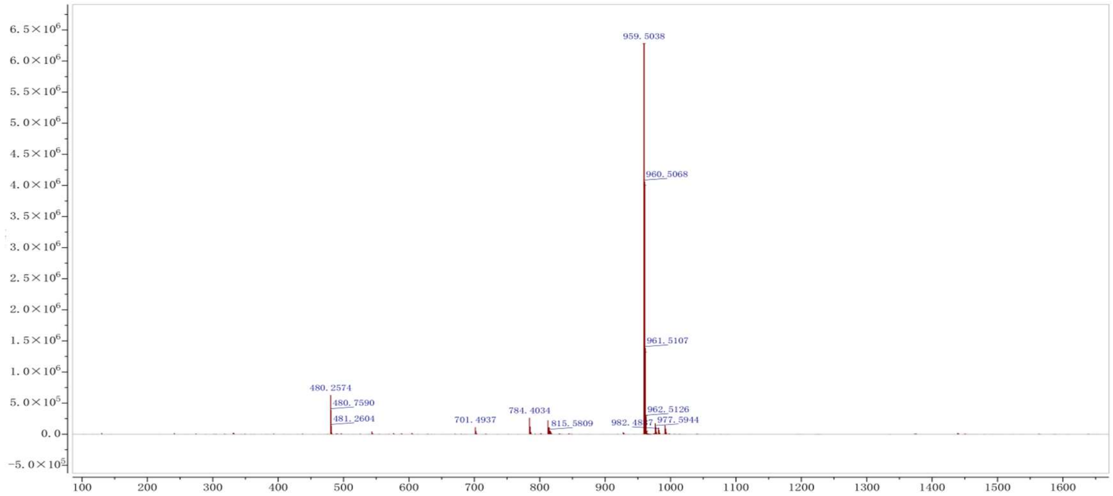

HRMS of **B1-10P**

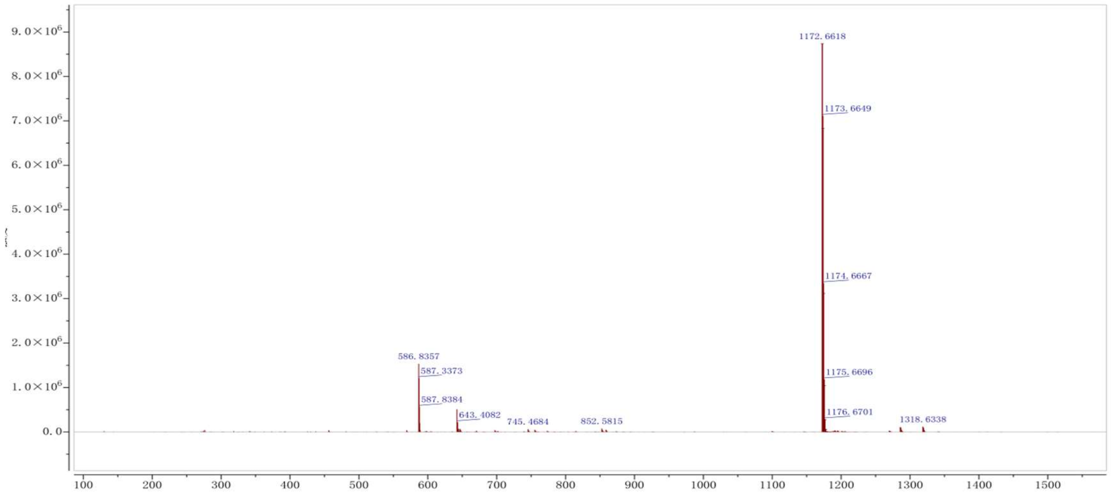

HRMS of **B2-12P**

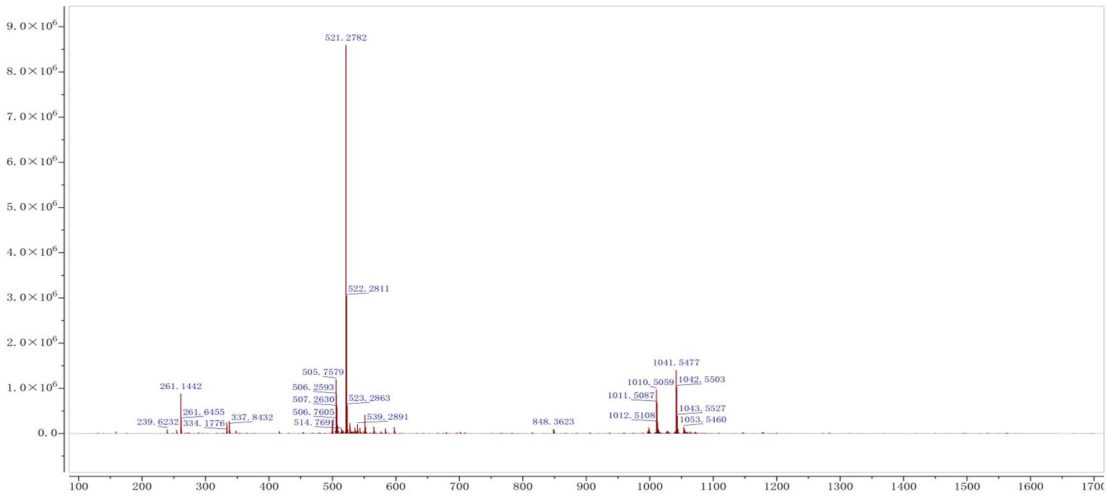

HRMS of **B-J**

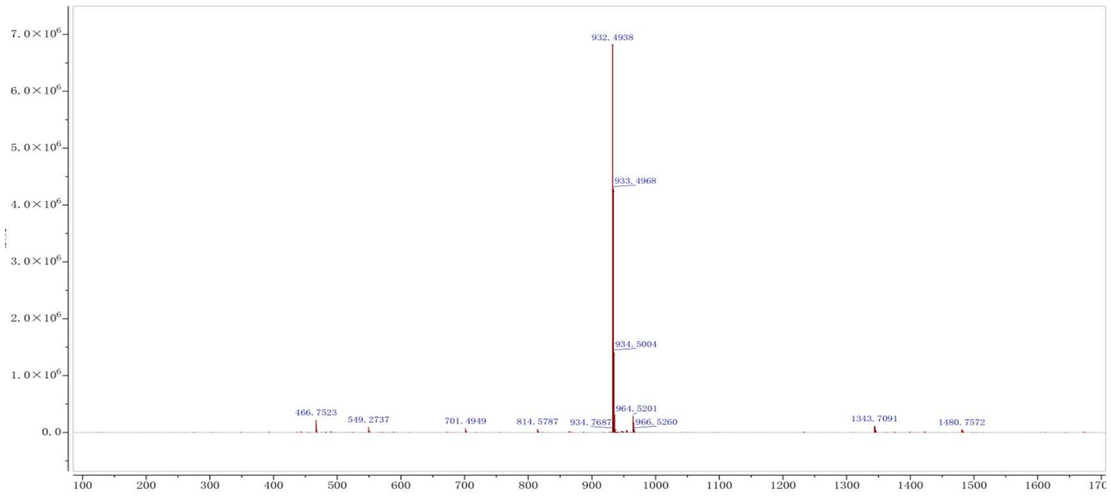

HRMS of **B1-10J**

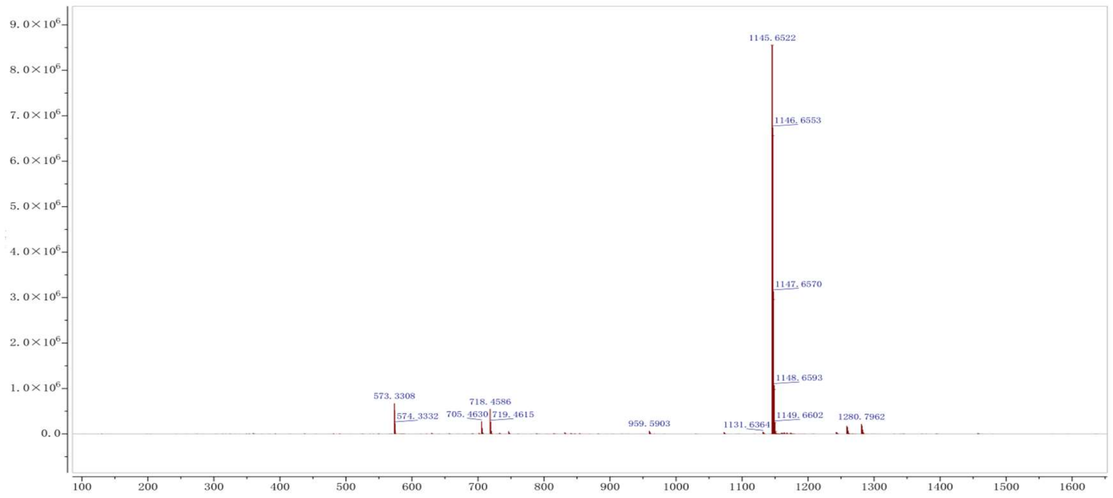

HRMS of **B2-12J**

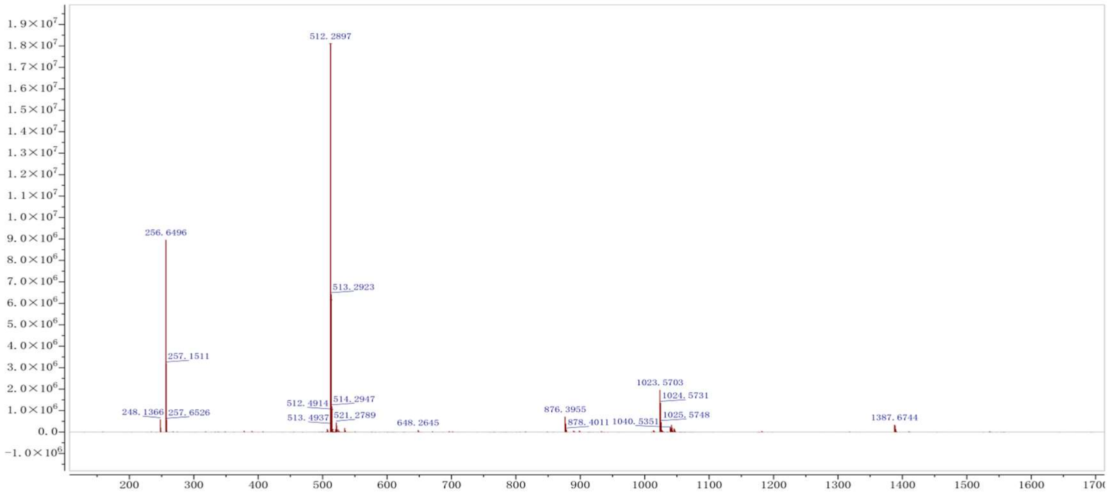

HRMS of **B-N**

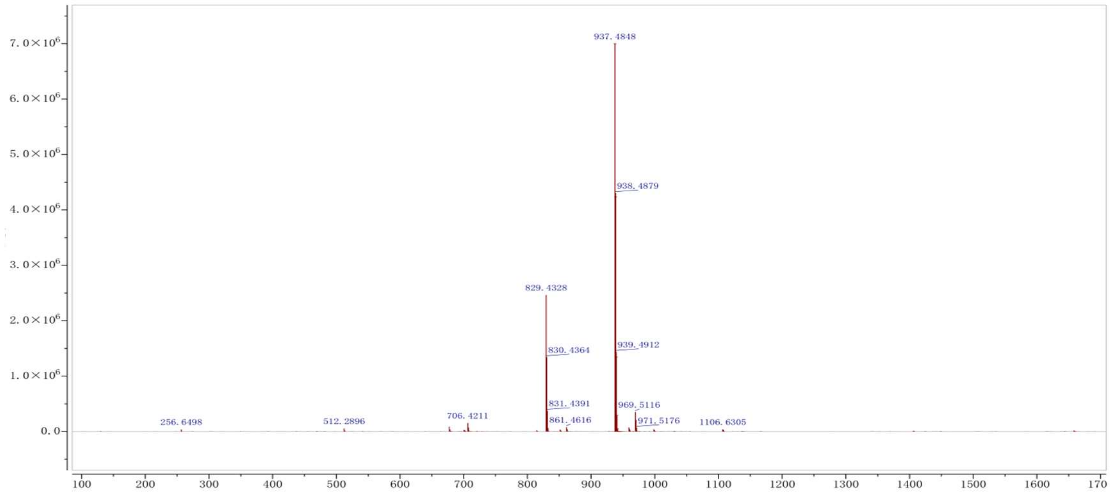

HRMS of **B1-10N**

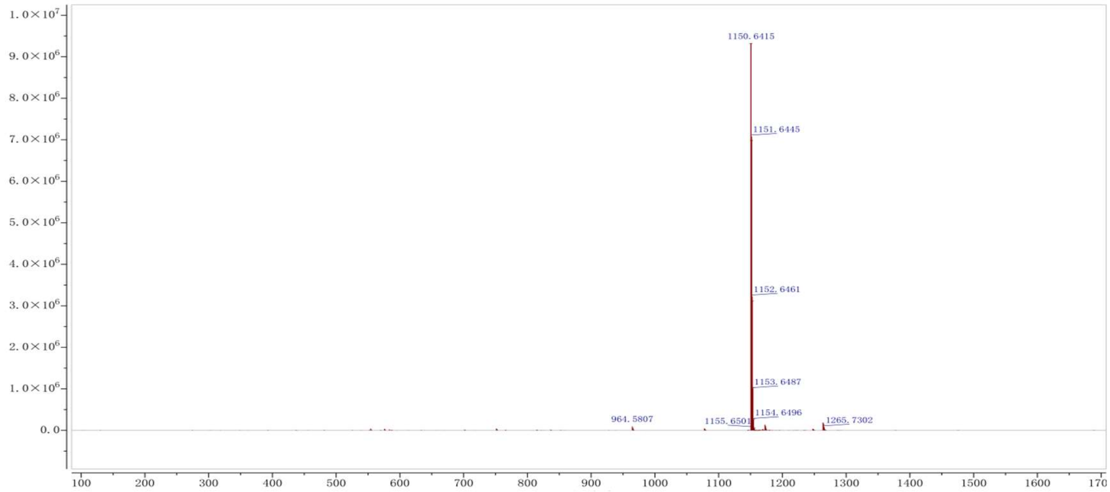

HRMS of **B2-12N**

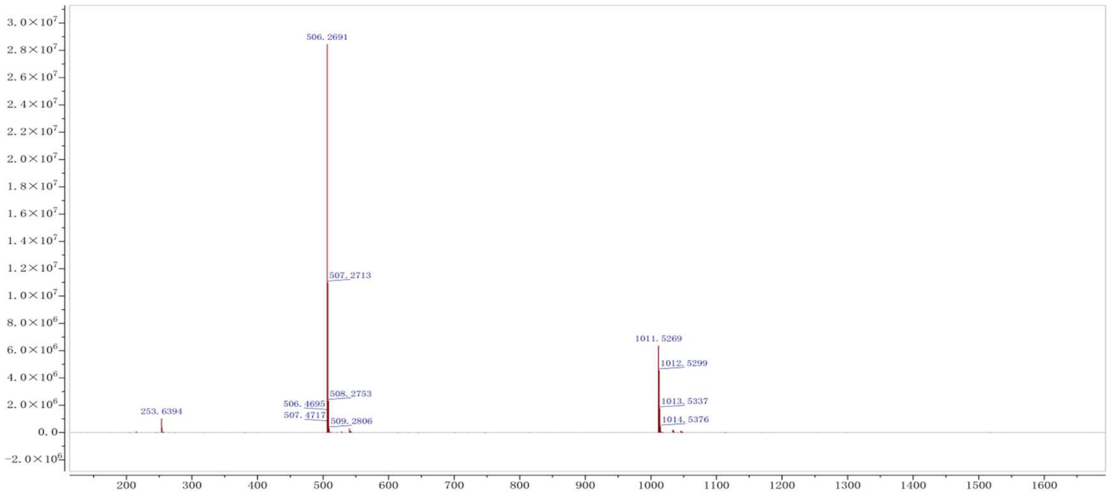

HRMS of **B-B**

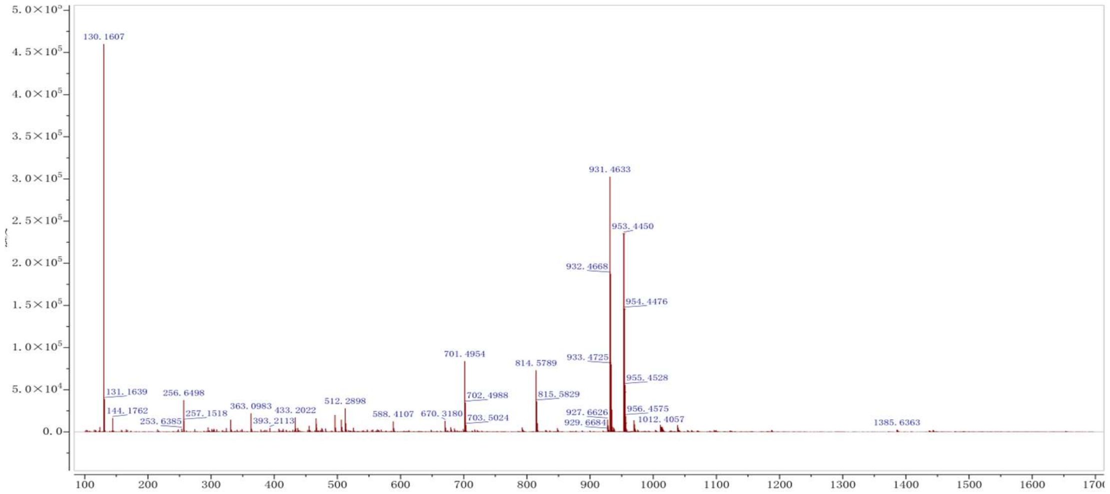

HRMS of **B1-10B**

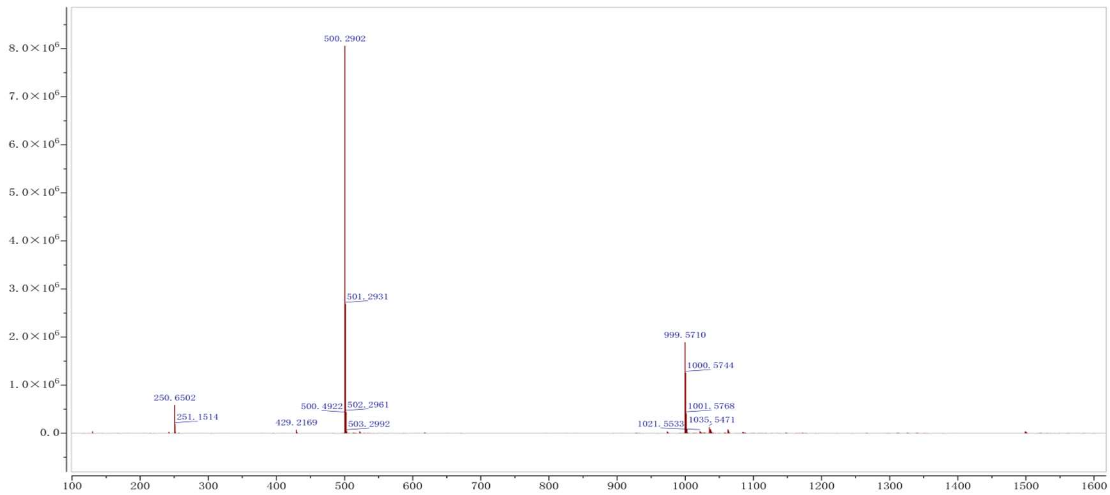

HRMS of **B-BZ**

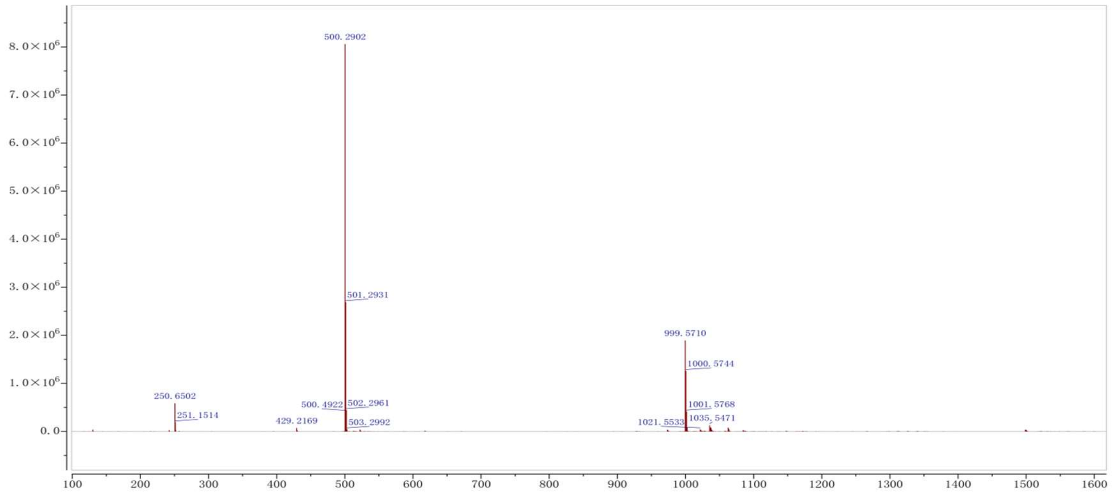

HRMS of **B1-10BZ**

7. HPLC

Gradient: 5% CH_3CN to 95% CH_3CN over 25 min at a flow rate of 1 mL/min.

Wavelength: 254 nm

No.	Ret. Time(min)	Area%
1	2.263	0.1894
2	17.536	2.4889
3	18.362	97.1827
4	19.073	0.1390

Purity: 97.1827%

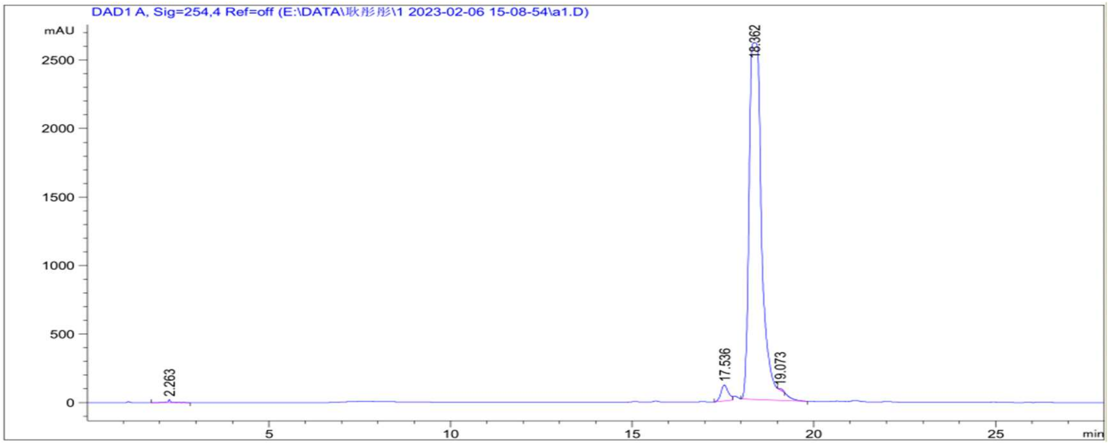

HPLC of **A1**

No.	Ret. Time(min)	Area%
1	2.245	0.1893
2	14.774	0.5346
3	18.288	99.2761

Purity: 99.2761%

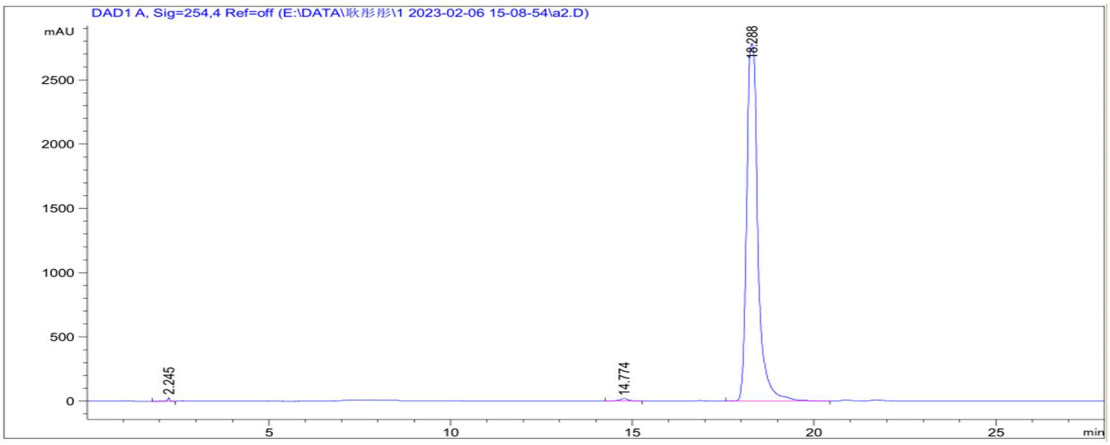

HPLC of **A2**

No.	Ret. Time(min)	Area%
1	17.884	0.5775
2	18.722	0.7747
3	19.289	98.0328
4	23.633	0.6150

Purity: 98.0328%

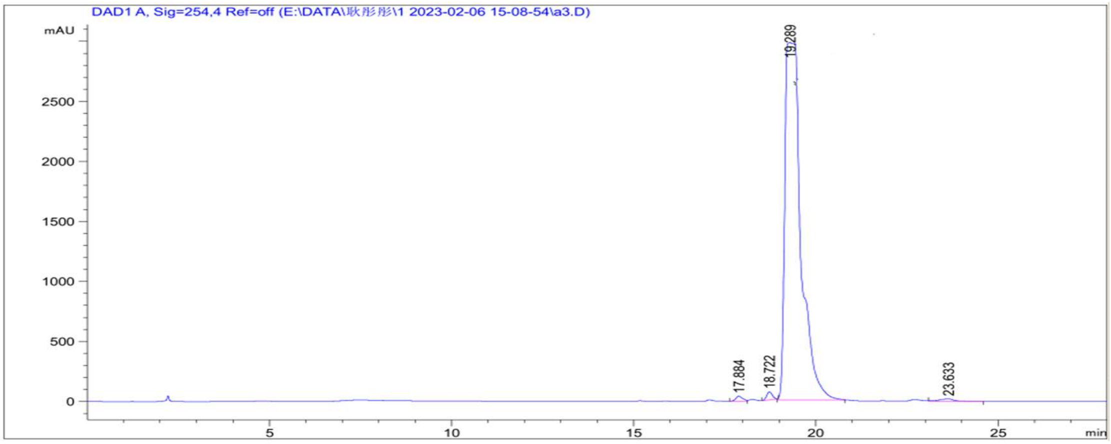

HPLC of **A3**

No.	Ret. Time(min)	Area%

1	18.873	0.2755
2	19.934	0.4753
3	21.350	99.2491

Purity: 99.2491%

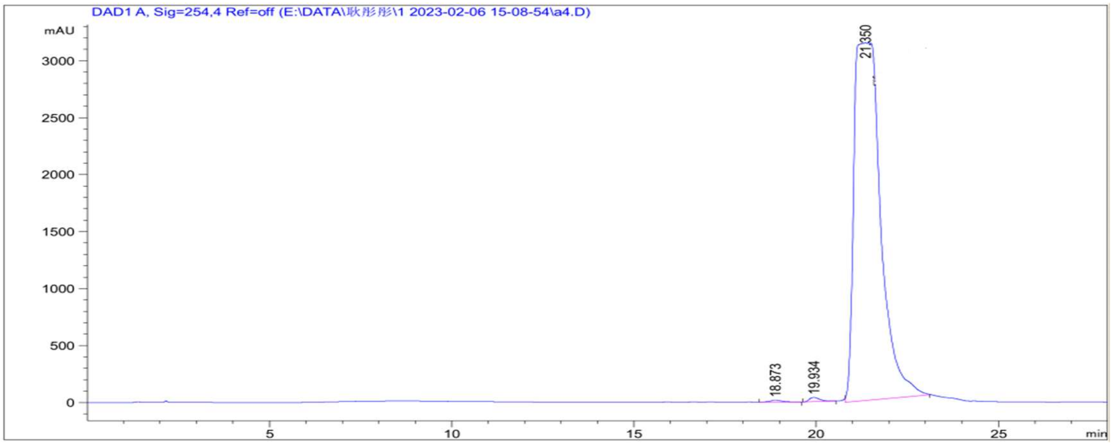

HPLC of **A4**

No.	Ret. Time(min)	Area%
1	2.181	0.2409
2	17.668	99.0219
3	21.176	0.7372

Purity: 99.0219%

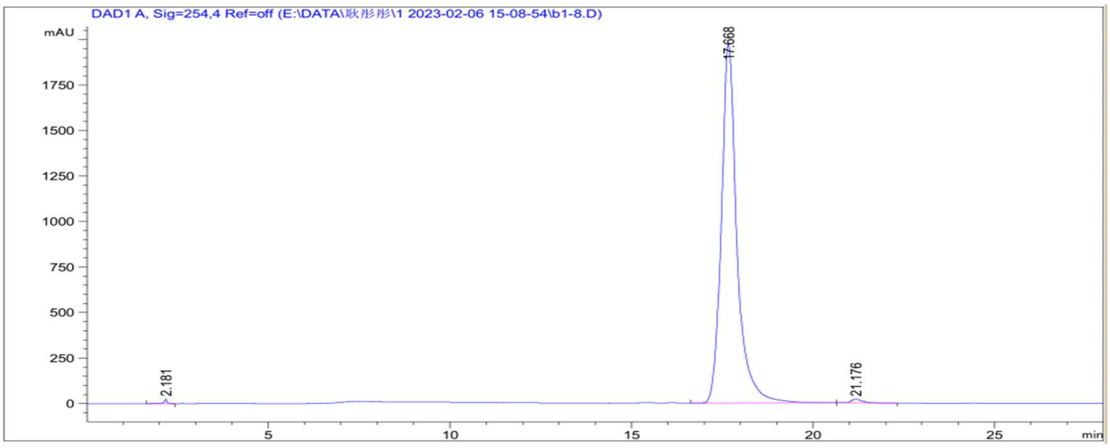

HPLC of **B1-8**

No.	Ret. Time(min)	Area%
1	2.180	0.1924
2	14.989	0.3818

3	18.010	99.4258

Purity: 99.4258%

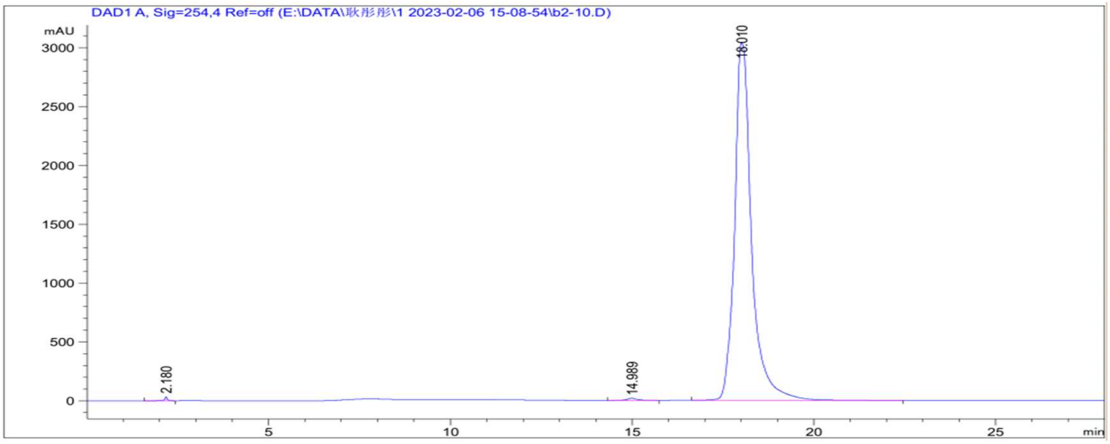

HPLC of **B2-10**

No.	Ret. Time(min)	Area%
1	15.486	0.2335
2	17.046	0.7765
3	18.487	98.0690
4	22.779	0.9211

Purity: 98.0690%

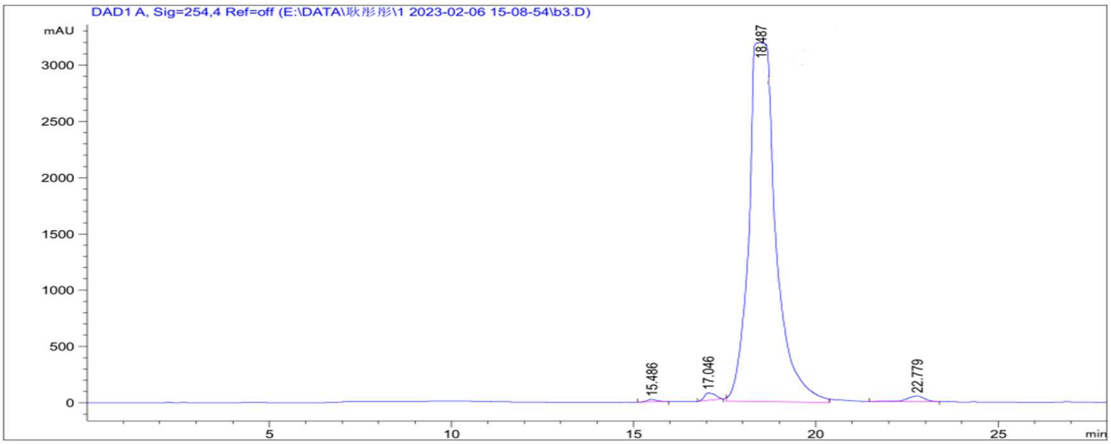

HPLC of **B3**

No.	Ret. Time(min)	Area%
1	2.221	0.1742
2	15.384	0.1648
3	17.996	0.2492

4	20.933	99.4118

Purity: 99.4118%

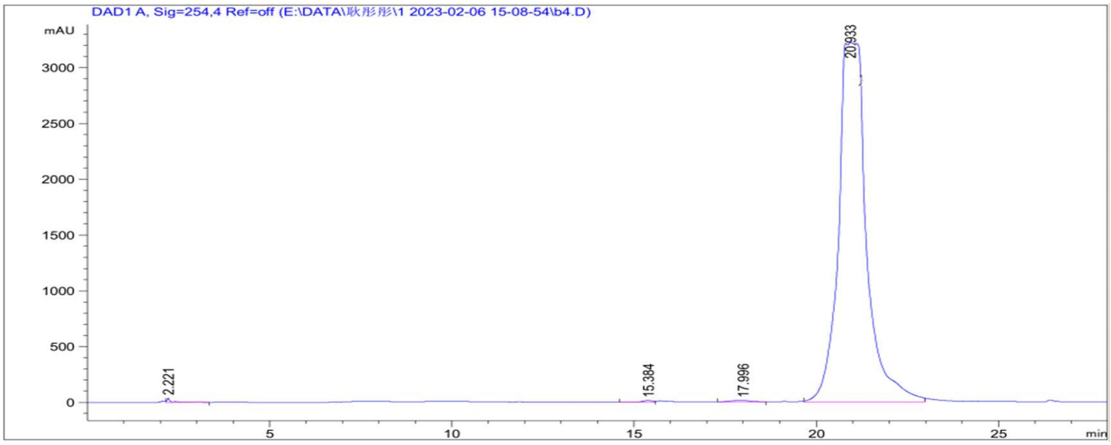

HPLC of **B4**

No.	Ret. Time(min)	Area%
1	2.221	100.0000

Purity: 100.0000%

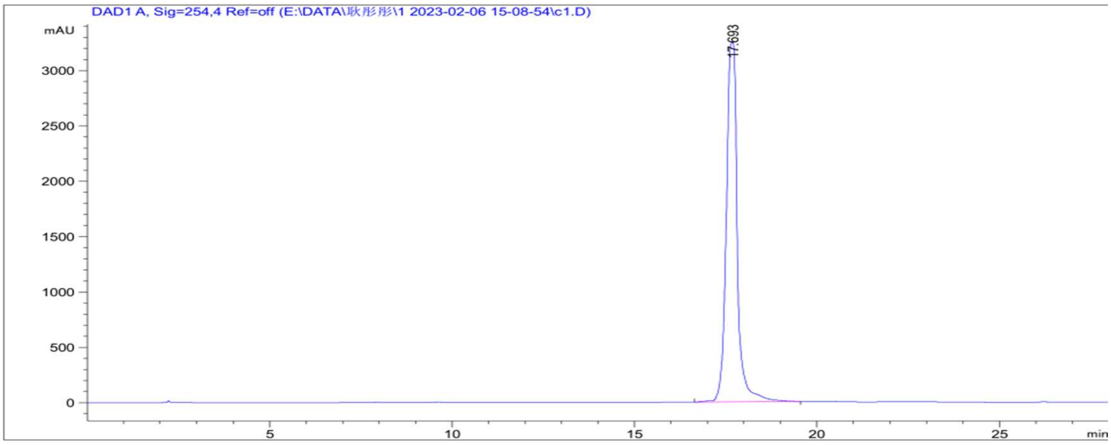

HPLC of **C1**

No.	Ret. Time(min)	Area%
1	14.891	0.2886
2	15.226	0.4499
3	17.984	99.2615

Purity: 99.2615%

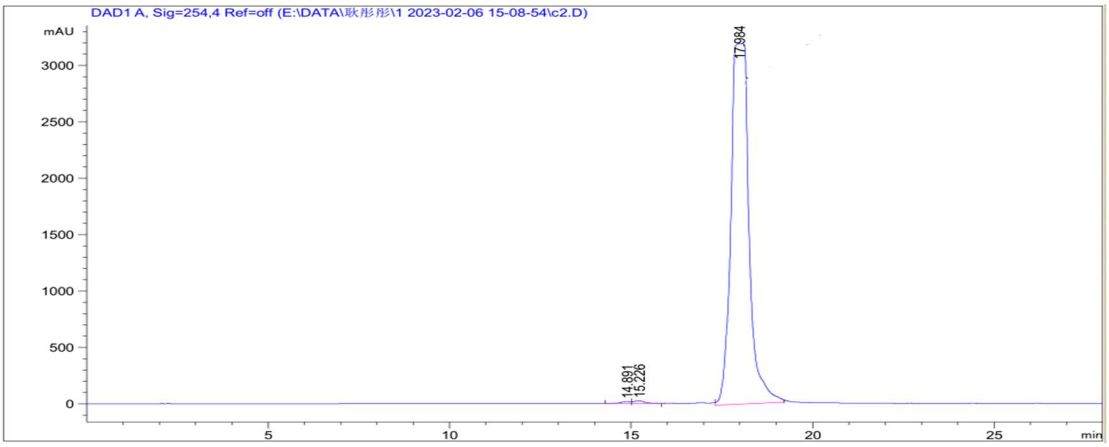

HPLC of **C2**

No.	Ret. Time(min)	Area%
1	16.352	0.1951
2	17.309	0.1237
3	18.594	99.6813

Purity: 99.6813%

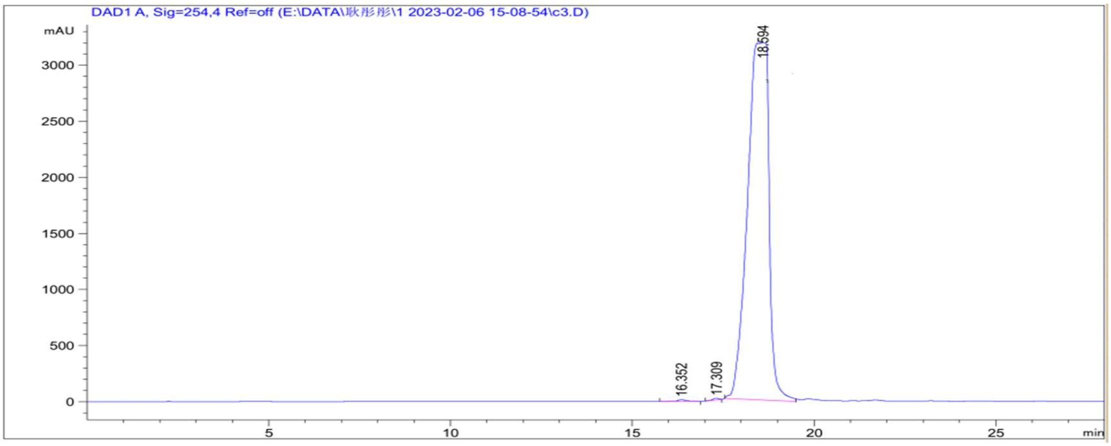

HPLC of **C3**

No.	Ret. Time(min)	Area%
1	17.422	0.2888
2	20.331	99.7112

Purity: 99.7112%

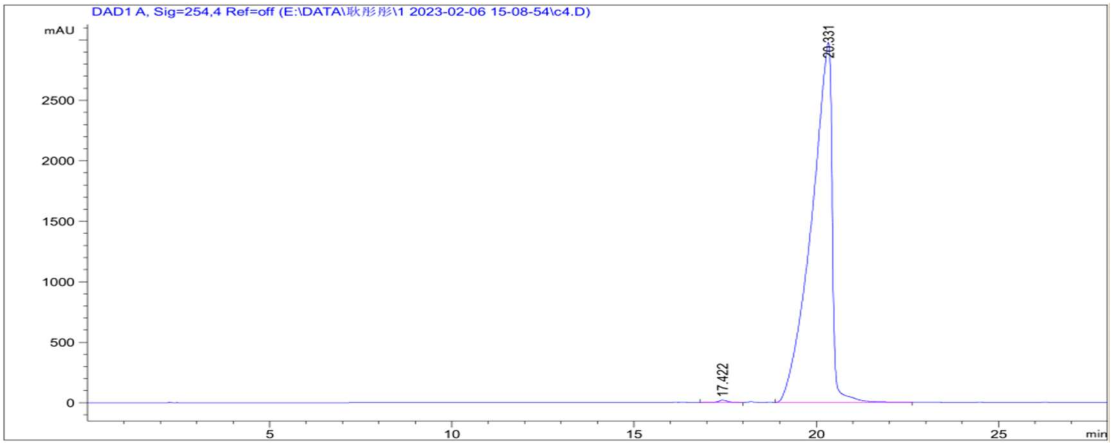

HPLC of **C4**

No.	Ret. Time(min)	Area%
1	12.217	95.7649
2	22.833	4.2351

Purity: 95.7649%

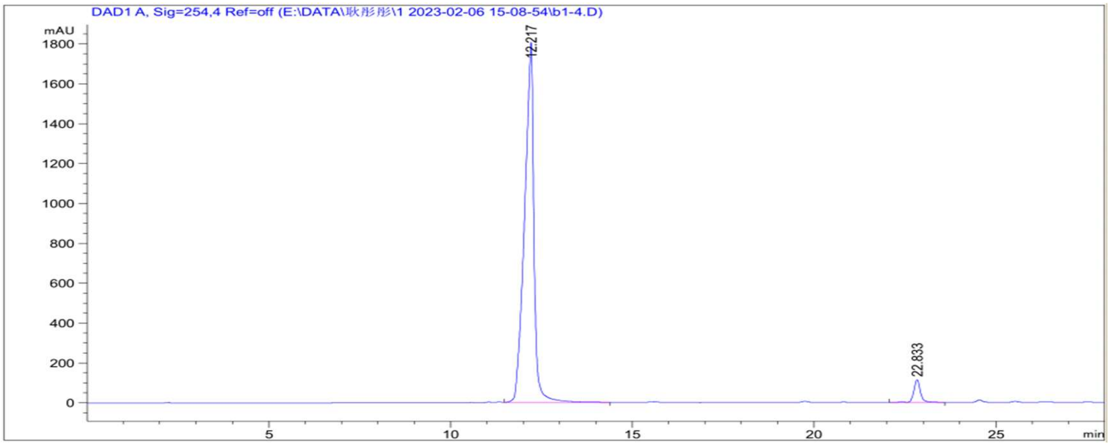

HPLC of **B1-4**

No.	Ret. Time(min)	Area%
1	12.848	95.3459
2	22.843	4.2385
3	24.561	0.4156

Purity: 95.3459%

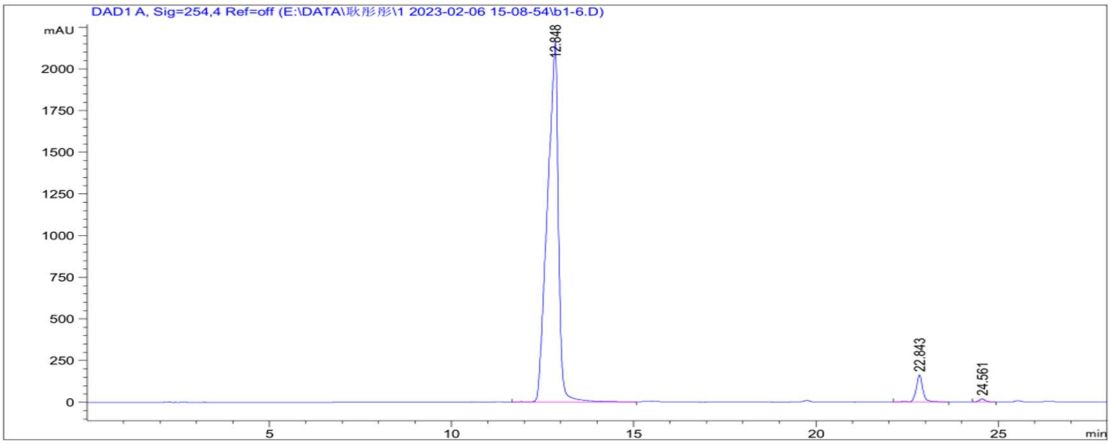

HPLC of **B1-6**

No.	Ret. Time(min)	Area%
1	15.044	95.1976
2	22.856	4.8024

Purity: 95.1976%

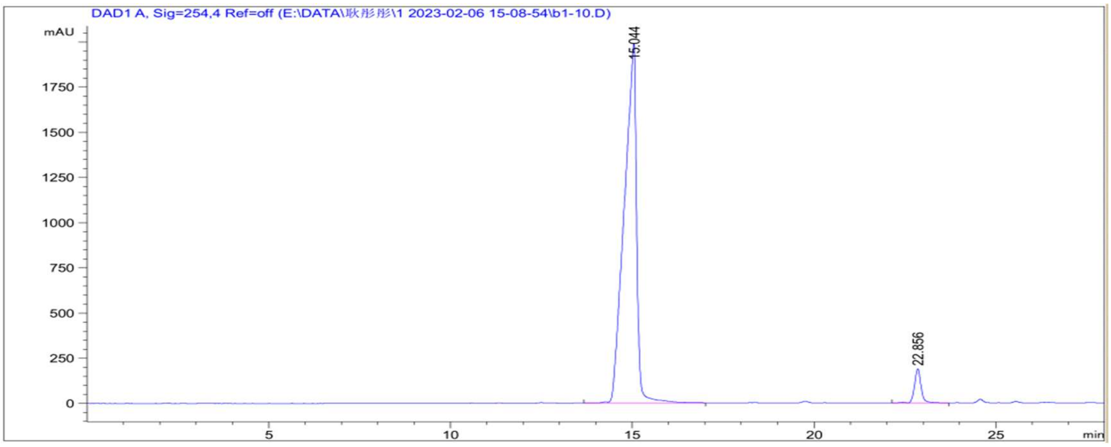

HPLC of **B1-10**

No.	Ret. Time(min)	Area%
1	16.121	95.0166
2	22.841	4.9834

Purity: 95.0166%

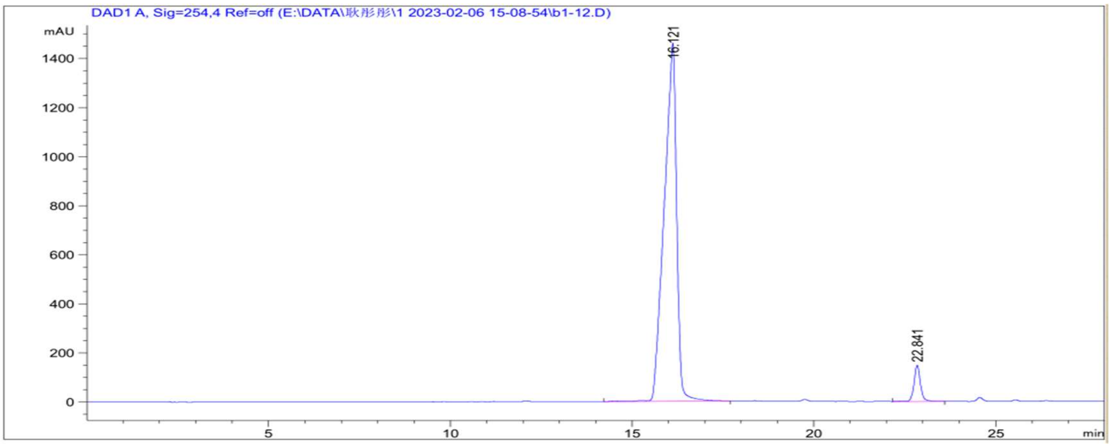

HPLC of **B1-12**

No.	Ret. Time(min)	Area%
1	10.846	3.361
2	11.192	95.950
3	21.645	0.689

Purity: 95.950%

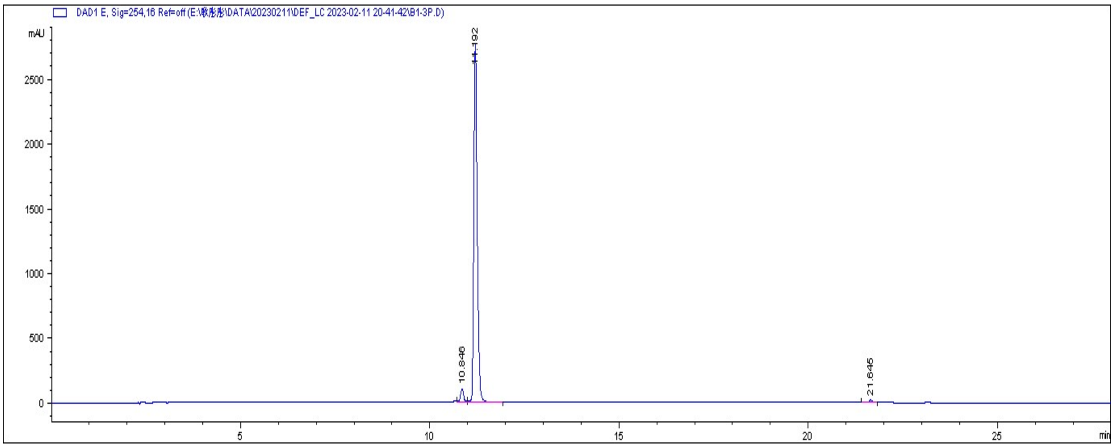

HPLC of **B1-3P**

No.	Ret. Time(min)	Area%
1	10.844	1.030
2	11.396	98.219
3	21.639	0.751

Purity: 98.219%

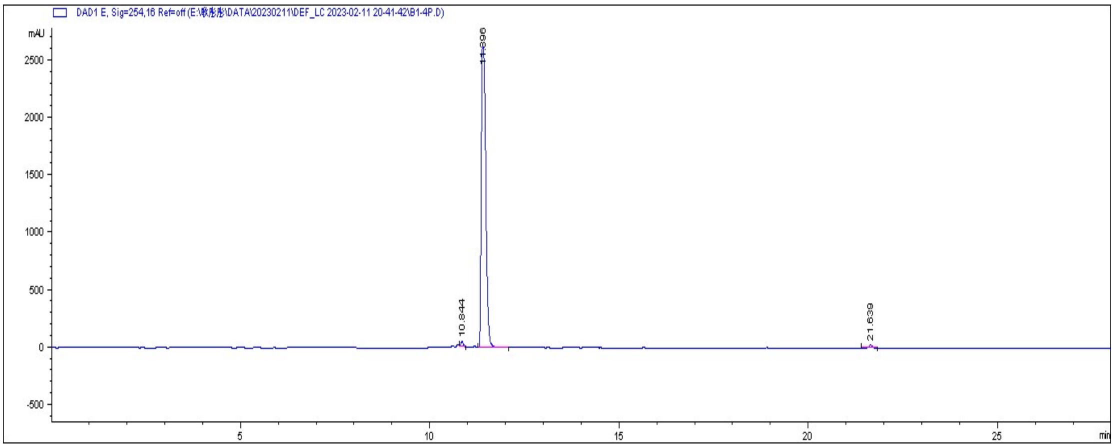

HPLC of **B1-4P**

No.	Ret. Time(min)	Area%
1	10.950	1.2848
2	11.840	2.1462
3	12.556	96.5690

Purity: 96.5690%

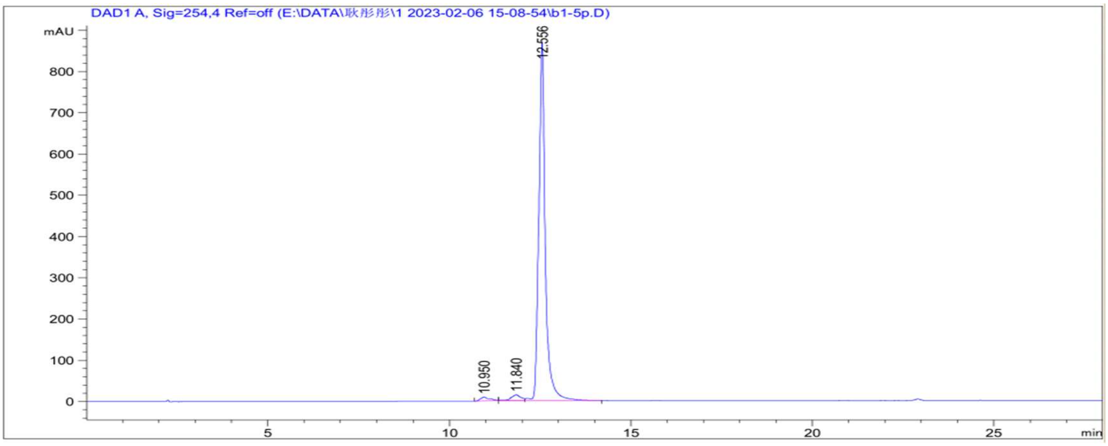

HPLC of **B1-5P**

No.	Ret. Time(min)	Area%
1	13.492	100.0000

Purity: 100.0000%

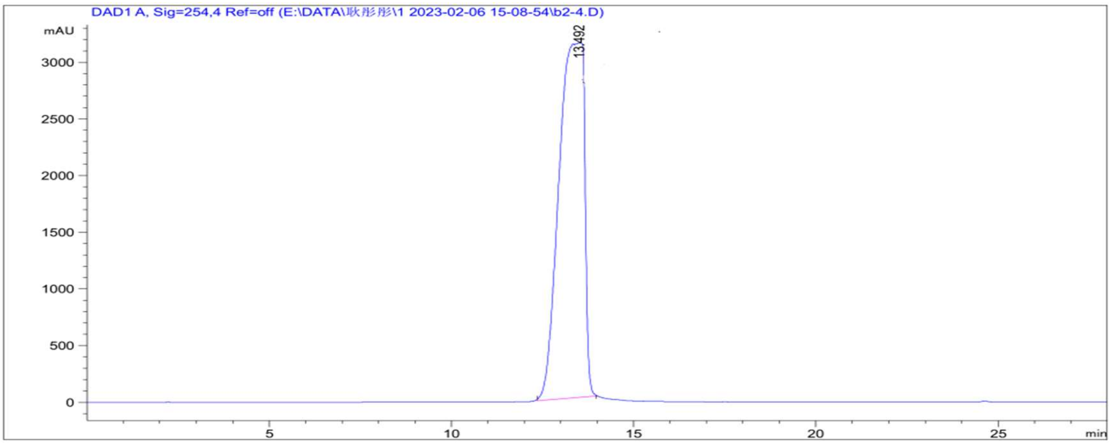

HPLC of **B2-4**

No.	Ret. Time(min)	Area%
1	13.462	100.0000

Purity: 100.0000%

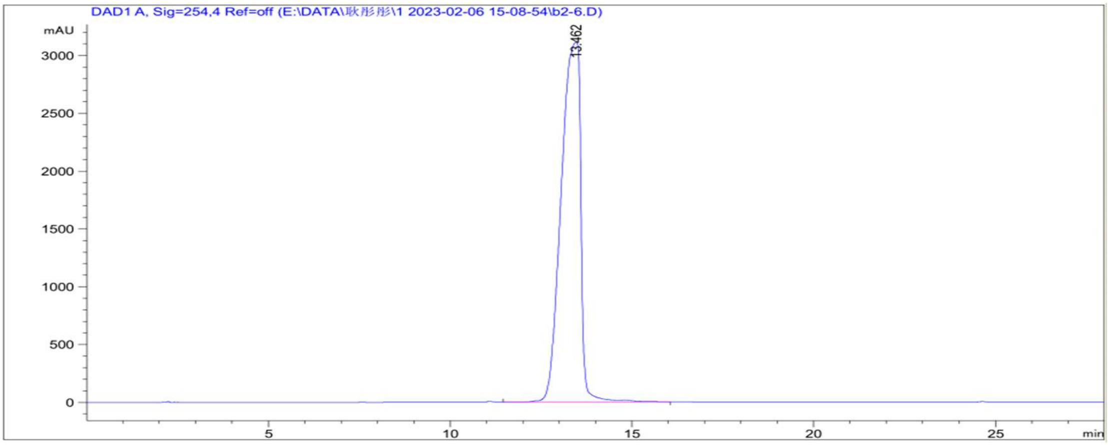

HPLC of **B2-6**

No.	Ret. Time(min)	Area%
1	13.4983	99.2071
2	15.660	0.7929

Purity: 99.2071%

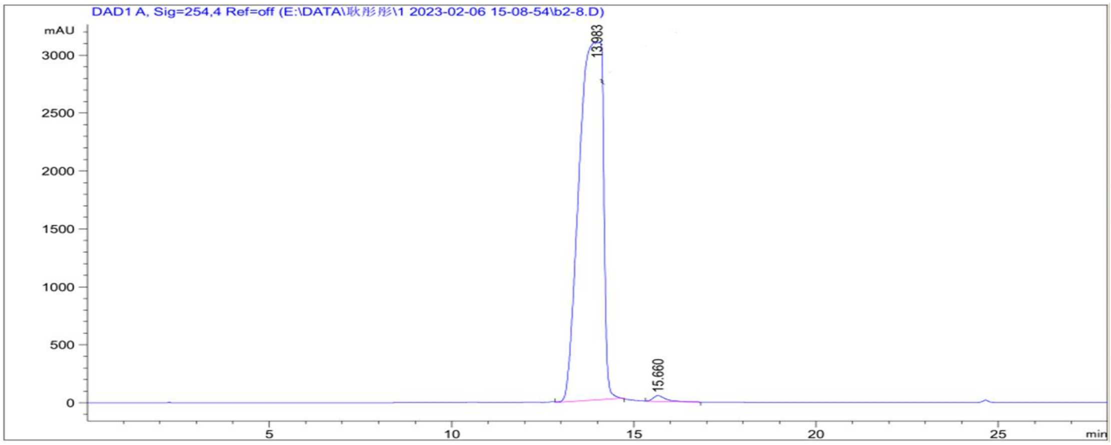

HPLC of **B2-8**

No.	Ret. Time(min)	Area%
1	10.825	1.5374
2	14.917	96.8397
3	16.181	0.5372
4	18.026	1.0858

Purity: 96.8397%

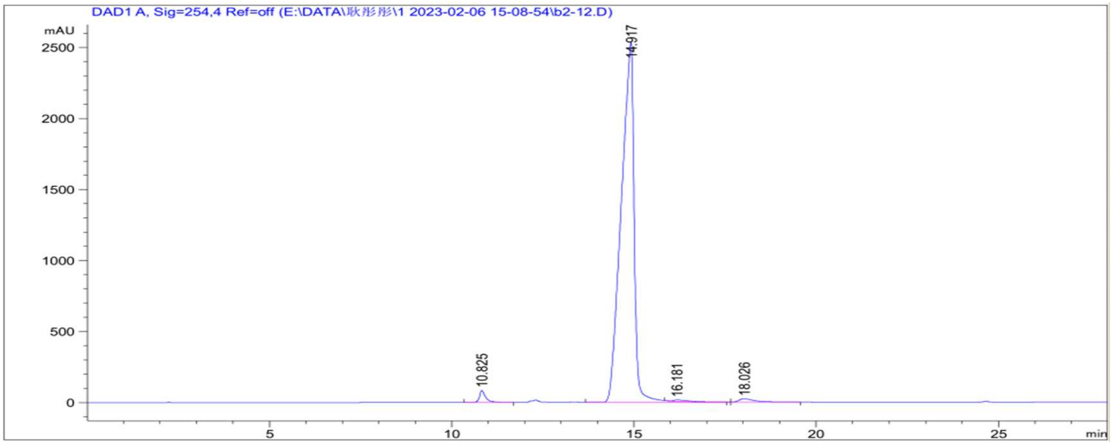

HPLC of **B2-12**

No.	Ret. Time(min)	Area%
1	10.820	1.6046
2	12.291	0.3296
3	15.056	96.2597
4	16.187	0.6004
5	18.017	1.2057

Purity: 96.2597%

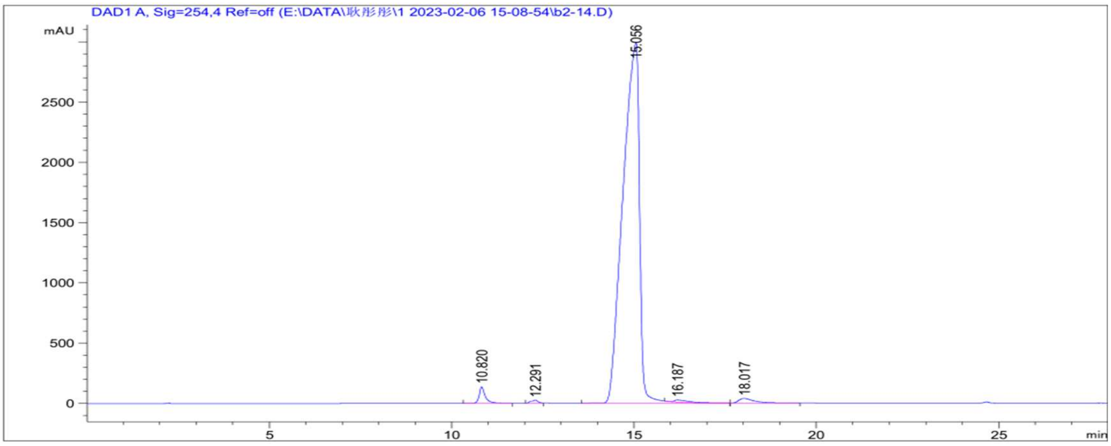

HPLC of **B2-14**

No.	Ret. Time(min)	Area%
1	10.204	0.2198
2	12.652	98.8697
3	14.717	0.9105

Purity: 98.8697%

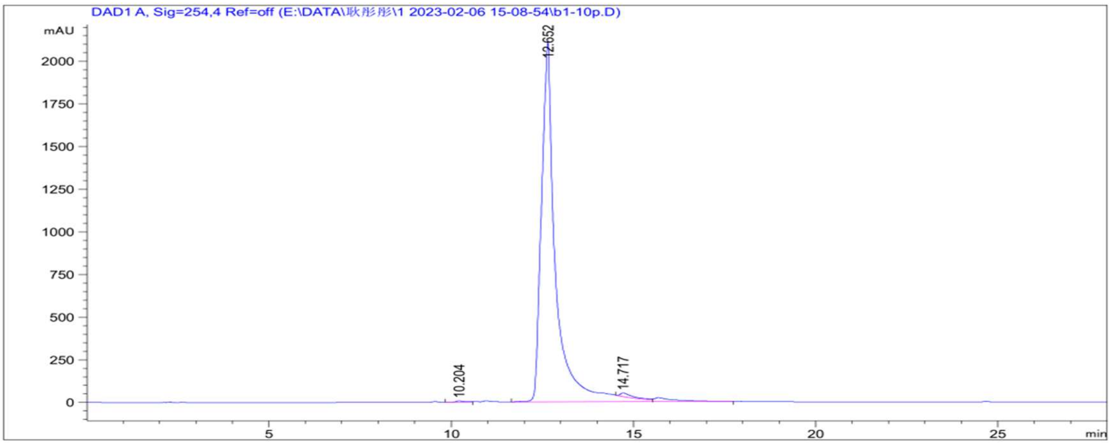

HPLC of **B1-10P**

No.	Ret. Time(min)	Area%
1	9.429	0.6530
2	9.596	0.9906
3	12.382	95.3404
4	13.451	3.0160

Purity: 95.3404%

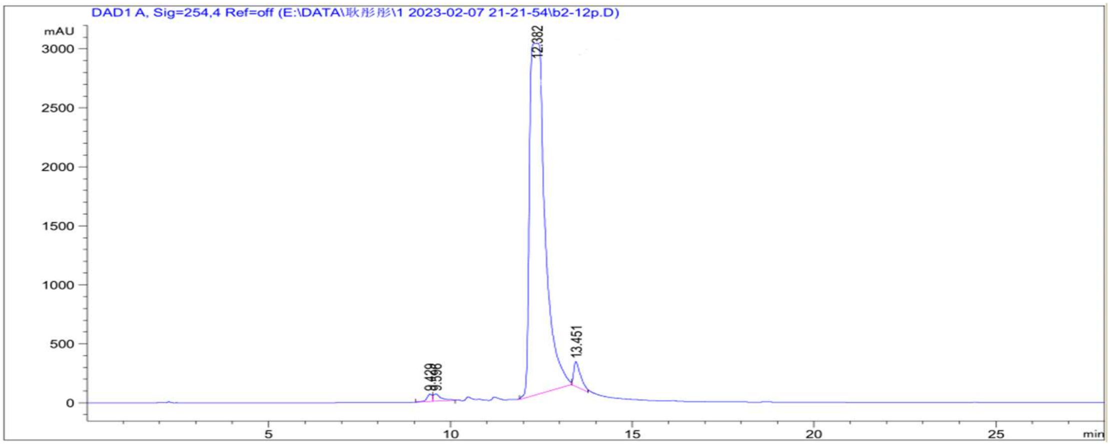

HPLC of **B2-12P**

No.	Ret. Time(min)	Area%
1	13.622	99.5943
2	22.939	0.4057

Purity: 99.5943%

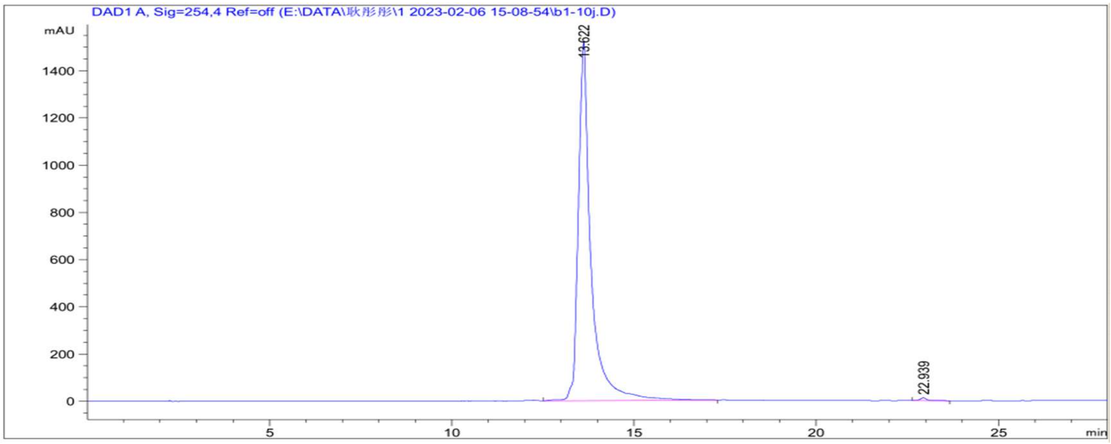

HPLC of **B1-10J**

No.	Ret. Time(min)	Area%
1	11.368	1.544
2	12.359	97.989
3	15.382	0.467

Purity: 97.989%

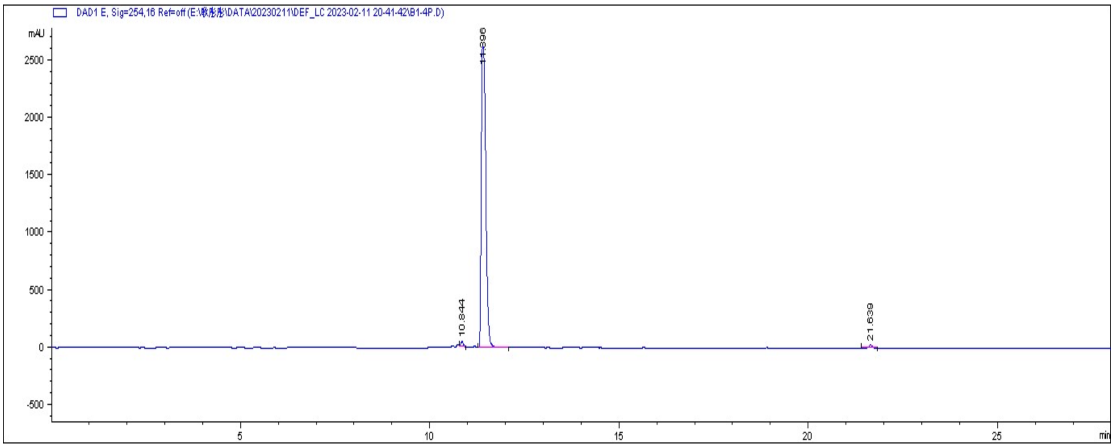

HPLC of **B2-12J**

No.	Ret. Time(min)	Area%
1	12.169	0.2445
2	12.754	0.6603
3	13.604	99.0951

Purity: 99.0951%

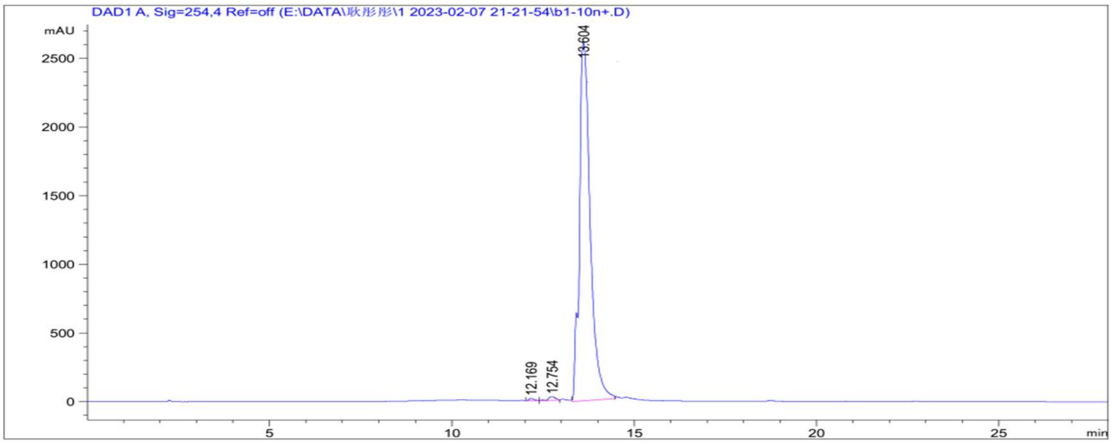

HPLC of **B1-10N**

No.	Ret. Time(min)	Area%
1	10.297	0.6646
2	12.545	0.1969
3	13.747	99.1385

Purity: 99.1385%

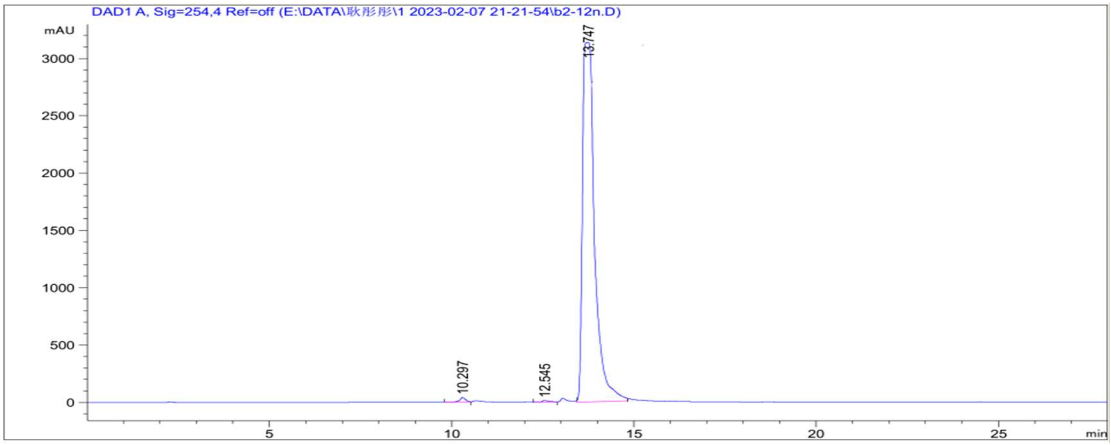

HPLC of **B2-12N**

No.	Ret. Time(min)	Area%
1	12.438	0.5648
2	14.096	0.9196
3	19.152	97.7393
4	19.762	0.7762

Purity: 97.7393%

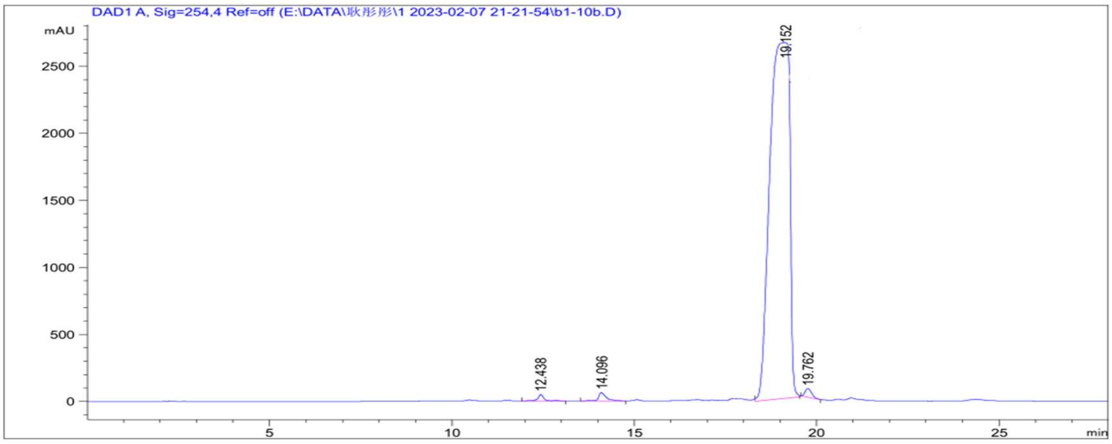

HPLC of **B1-10B**

No.	Ret. Time(min)	Area%
1	11.288	0.6866
2	15.535	99.3134

Purity: 99.3134%

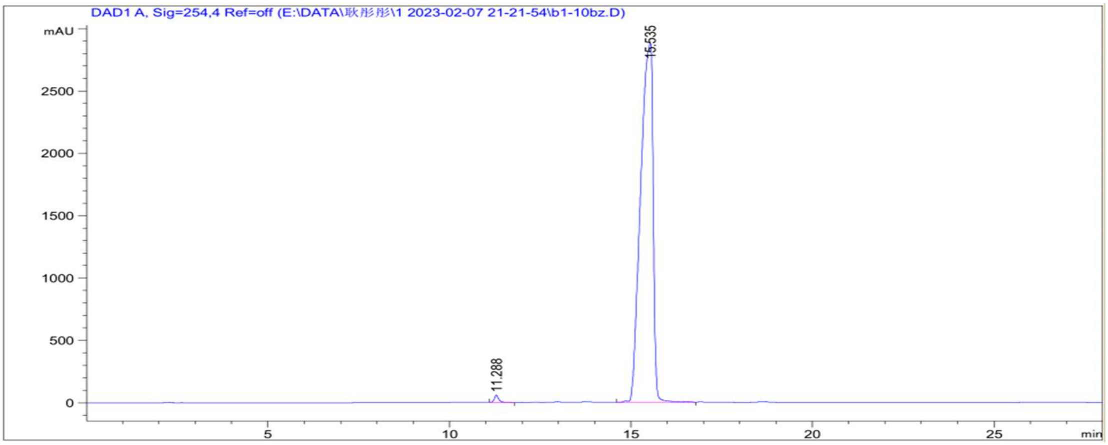

HPLC of **B1-10BZ**
